# Photoredox-enabled 1,2-dialkylation of α-substituted acrylates *via* Ireland–Claisen rearrangement†

**DOI:** 10.1039/d0sc06385a

**Published:** 2021-01-07

**Authors:** Roman Kleinmans, Leon E. Will, J. Luca Schwarz, Frank Glorius

**Affiliations:** Organisch-Chemisches Institut, Westfälische Wilhelms-Universität Münster Corrensstraße 40 48149 Münster Germany glorius@uni-muenster.de

## Abstract

Herein, we report the 1,2-dialkylation of simple feedstock acrylates for the synthesis of valuable tertiary carboxylic acids by merging Giese-type radical addition with an Ireland–Claisen rearrangement. Key to success is the utilization of the reductive radical-polar crossover concept under photocatalytic reaction conditions to force the [3,3]-sigmatropic rearrangement after alkyl radical addition to allyl acrylates. Using readily available alkyl boronic acids as radical progenitors, this redox-neutral, transition-metal-free protocol allows the mild formation of two C(sp^3^)–C(sp^3^) bonds, thus providing rapid access to complex tertiary carboxylic acids in a single step. Moreover, this strategy enables the efficient synthesis of highly attractive α,α-dialkylated γ-amino butyric acids (GABAs) when α-silyl amines are used as radical precursors – a structural motif that was still inaccessible in related transformations. Depending on the nature of the radical precursors and their inherent oxidation potentials, either a photoredox-induced radical chain or a solely photoredox mechanism is proposed to be operative.

## Abstract 2

A photocatalytic 1,2-dialkylation of α-substituted acrylates is enabled by a reaction cascade combining reductive radical-polar crossover with the established Ireland–Claisen rearrangement for the synthesis of valuable tertiary carboxylic acids.

## Graphical abstract

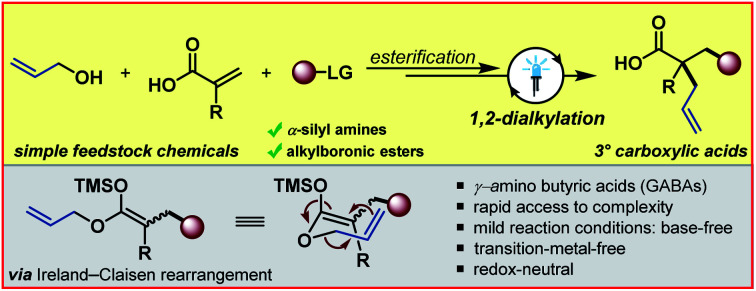

## Introduction

Carboxylic acids and their derivatives are among the most common structural motifs in natural products, biologically active compounds and materials science.^1^ In addition, carboxylic acids play an important role in organic synthesis owing to their high diversifiability.^1,2^ Nowadays, especially decarboxylative approaches for functional group interconversion or cross-coupling reactions are of great importance.^3^ The coupling of tertiary carboxylic acids to obtain otherwise difficult-to-access quaternary centers has recently gained significant attention.^4^ The synthesis of tertiary carboxylic acids, however, is still challenging and often requires harsh reaction conditions.^5^ An easy and mild access to complex tertiary carboxylic acids from readily available and simple starting materials is therefore highly desirable.

**Fig. 1:**
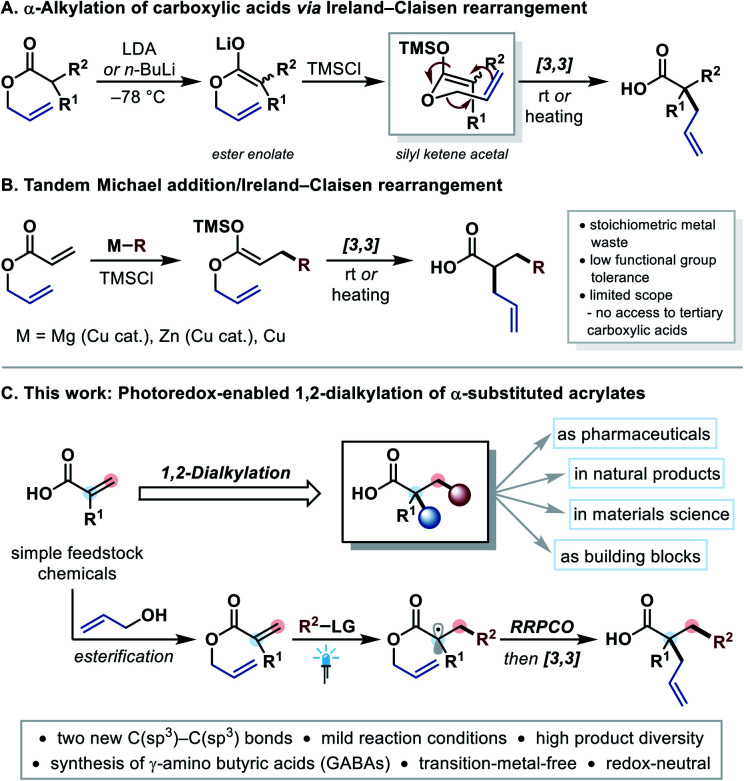
Background, motivation, and features of the current work. LG = leaving group, RRPCO = reductive radical-polar crossover.

A classical method is the α-alkylation of secondary carboxylic acids *via* Ireland–Claisen rearrangement (Fig. 1A).^6,7^ In this reaction, allyl esters are treated with strong bases such as LDA (lithium diisopropylamide) or *n*-BuLi under cryogenic conditions to form an ester enolate. This enolate is converted to the respective silyl ketene acetal, which can undergo a [3,3]-sigmatropic rearrangement at ambient temperature. Although this method is highly valuable from a synthetic point of view,^8^ the incompatibility with base-sensitive functional groups is adversely.^9^ Moreover, the main skeleton of the carboxylic acid has to be preset. Alternatively, the tandem Michael addition of carbon-nucleophiles to allyl acrylates with a subsequent Ireland–Claisen rearrangement forms two new C–C bonds at once, which generates more complexity in one step (Fig. 1B).^10^ However, these reactions often suffer from low functional group tolerance, limited substrate scope and produce stoichiometric quantities of metal waste (Mg, Zn, Cu). Furthermore, none of these methods could be applied to synthesize tertiary carboxylic acids.^10^

In contrast, a modern strategy for forming C–C bonds in the β-position of electron withdrawing groups is the photoredox-mediated Giese-type radical addition to Michael acceptors.^11,12^ The product radical can further be reduced to the corresponding carbanion, which is known as reductive radical-polar crossover (RRPCO).^13^ In most cases these carbanions are simply protonated,^14^ and there are only a few examples, in which carbon-electrophiles were employed to trap the anion, resulting in the formation of two new C–C bonds.^15,16^ With that in mind, we questioned whether it would be possible to merge photoredox-mediated RRPCO with a subsequent Ireland–Claisen rearrangement using allyl acrylates as Michael acceptors (Fig. 1C). This strategy would lead to an underexplored but highly desirable 1,2-dialkylation of α-substituted acrylates. Owing to the typically mild nature of photoredox catalysis,^11^ this approach was expected to tolerate a wide range of functional groups allowing an efficient synthesis of valuable tertiary carboxylic acids.

## Results and discussion

We began our investigation with the evaluation of a suitable alkyl radical precursor using allyl methacrylate (**2a**) as standard substrate under visible-light-mediated photocatalytic reaction conditions in the presence of trimethylsilyl chloride (TMSCl). Unfortunately, most of the common radical precursors^11*d*^ did not provide the desired product. Carboxylates and alkyl trifluoroborates, for example, were incompatible under the reaction conditions because of side reactions with TMSCl, while halogen atom abstraction (XAT)^17^ approaches or the use of Hantzsch esters almost exclusively resulted in the formation of the respective Giese-type side product (see ESI†). Pleasingly, utilization of the recently introduced, easily oxidizable arylboronates^15*d*,18^ yielded the carboxylic acid product **3a** when using cyclohexylboronic ester (**1a**) as radical progenitor and 4CzIPN as photocatalyst (Table 1).^19^ The corresponding arylboronates were preformed *in situ* by treating the readily available boronic esters^20^ with phenyllithium (PhLi). Extensive optimization led to the conditions shown in entry 1, which afforded the desired product in 76% ^1^H NMR yield. Interestingly, performing the reaction in solely THF significantly diminished the product yield (entry 2). The use of Ir-based photocatalysts instead of 4CzIPN decreased the yield of the desired product (entries 3 and 4).^19^ In contrast to other protocols employing arylboronate complexes as radical precursors,^15*d*,18^ excess PhLi was adversely (entry 5). The addition of TMSCl is crucial for this reaction (entry 6), and even the change to the sterically more hindered *tert*-butyldimethylsilyl chloride (TBSCl) inhibited the reaction entirely (entry 7). Control experiments revealed that the reaction does not proceed in the absence of light, photocatalyst or PhLi (entry 8). For a convenient and simple applicability of this transformation even on a larger scale, conditions were sought where no solvent has to be removed *in vacuo* during the reaction set-up. Nevertheless, changing the solvent to exclusively MeCN gave the product in 83% ^1^H NMR and 77% isolated yield (entry 9). Furthermore, a reaction-condition-based sensitivity screen was applied to facilitate high reproducibility (Table 1, see ESI for more details†).^21^ Apart from the expected moisture and air sensitivity, other parameters (concentration, light intensity, temperature, scale) had a negligible effect on the reaction outcome.

**Table 1:** Deviation from standard reaction conditions^a^

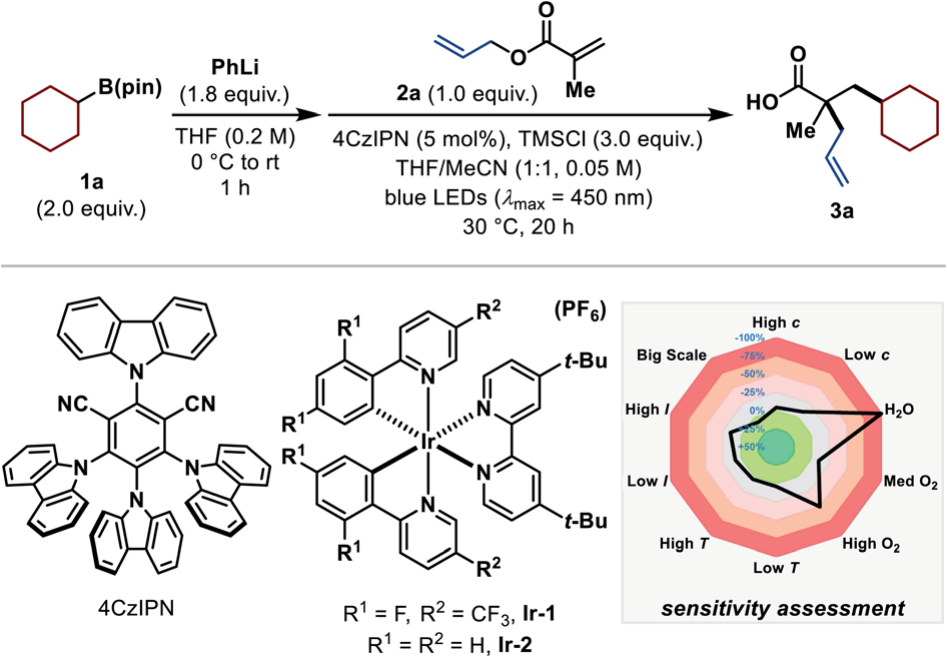		
Entry	Deviation from standard conditions	% yield **3a**
1	None	76 (74)
2	THF instead of MeCN/THF (1:1)	57
3	(**Ir-1**) (2 mol%) instead of 4CzIPN	68
4	(**Ir-2**) (2 mol%) instead of 4CzIPN	59
5	2.2 equiv. PhLi	51
6	No TMSCl	n.d.
7	TBSCl instead of TMSCl	n.d.
8	No light (40 °C), no PC or no PhLi	n.d.
**9**	**MeCN instead of MeCN/THF (1:1)**	**83 (77)**

a Reactions were performed on a 0.20 mmol scale. Yields were determined by ^1^H NMR spectroscopy using 1,3,5-trimethoxybenzene as internal standard. Isolated yields are given in parentheses. n.d. = not detected. See ESI for specified reaction conditions regarding the sensitivity assessment.

With these optimized reaction conditions in hand, we proceeded to investigate the substrate scope of this methodology (Table 2), starting with the boronic esters. Besides the cyclohexane moiety (**3a**), smaller and larger carbocycles could be installed in moderate to excellent yields (**3b** and **3c**). Up-scaling of the reaction by a factor of 20 had only a minor effect on the product yield (**3a**, and compare sensitivity assessment). Functional groups, such as protected secondary amines (**3d**), ethers (**3e**), and difluoromethylene (**3f**) groups were well tolerated. In addition, a boronic ester with an indane scaffold was successfully tested (**3g**), albeit giving a lower yield. Acyclic secondary boronic esters are also competent as demonstrated by product **3h**. Tertiary boronic esters such as 1-adamantyl and a gemfibrozil (Gevilon) derivative reacted in good yields to the products **3i** and **3j**, respectively. Notably, this leads to two all-carbon quaternary centers in a 1,3-distance. Primary boronic esters are currently a limitation, presumably owing to faster decomposition of the arylboronate complex with TMSCl (see ESI†).

**Table 2:** Scope of the 1,2-dialkylation of acrylates^a^

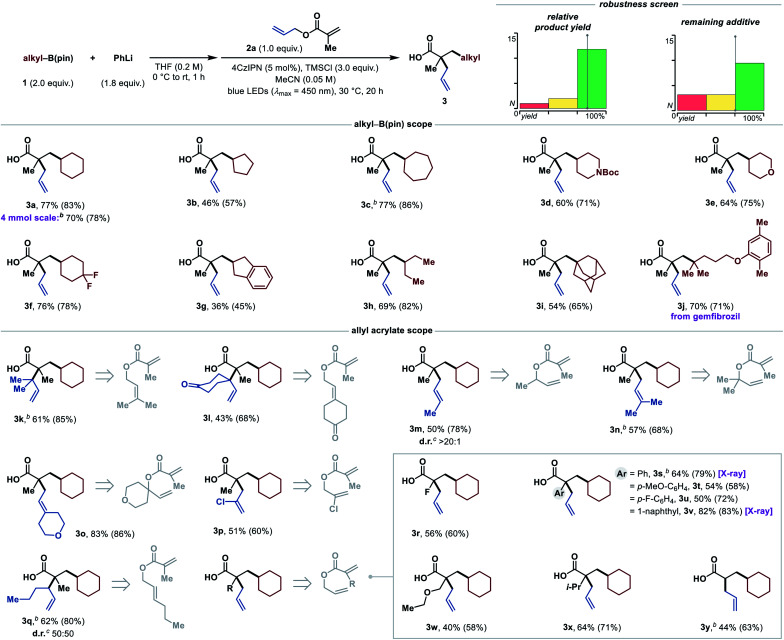

a Standard reaction conditions: **1** (2.0 equiv.), PhLi (1.9 M in dibutyl ether, 1.8 equiv.), THF (0.2 M) at 0 °C to rt for 1 h, then solvent exchange to MeCN (0.05 M), **2** (0.20 mmol, 1.0 equiv.), 4CzIPN (5 mol%), TMSCl (3.0 equiv.) at rt for 20 h under irradiation with blue LEDs (*λ*_max_ = 450 nm). Isolated yields are given. ^1^H NMR yields were determined with 1,3,5-trimethoxybenzene as internal standard and are given in parentheses. See ESI for full experimental details.

b Performed in THF/MeCN (1:1, 0.05 M).

c Diastereomeric ratio (d.r.) was determined by ^1^H NMR spectroscopy of the crude reaction mixture. Alkyl-B(pin) = alkylboronic acid pinacol ester, X-ray = see ESI for the crystal structure.

Next, we examined the allyl acrylate scope. This method not only allows the setting of quaternary centers in a 1,3-distance, but also in a vicinal fashion in good yields (**3k** and **3l**). The construction of contiguous, all-carbon quaternary centers still remains extremely challenging, and the occurrence of this structural motif in various natural products underpins the synthetic value of this transformation.^22^ In addition, **3l** shows that ketones, as an example of base-sensitive functional groups which pose a challenge in traditional Ireland–Claisen rearrangements, are well tolerated. Furthermore, this transformation offers highly diastereoselective access to *E*-olefins (**3m**) in high yield when secondary allylic alcohols are employed. This stereocontrol arises from the chair-like transition state of the Ireland–Claisen rearrangement, in which the substituent prefers the pseudo-equatorial position.^7^ Another advantage of this protocol is the rapid access to highly substituted olefins (**3n** and **3o**) from simple tertiary allylic alcohol-derived acrylate esters. Even an electron-withdrawing chloro-substituent in the 2-position of the allyl alcohol was tolerated to give the desired product **3p** in moderate yield. Additionally, an *E*-substituted allylic alcohol-derived acrylate ester was tested. The respective product **3q** was afforded in good yield, but unfortunately no diastereoselectivity was observed, which indicates that there is no geometry control during the silyl ketene acetal formation.^7^ As the next part of the allyl acrylate scope, different substituents in the α-position of the acrylate were tested. Pleasingly, a fluoro-substituent was tolerated, allowing the synthesis of α-fluoro tertiary carboxylic acid **3r** in a synthetically useful yield. These motifs are highly attractive, because they can be easily converted into a variety of valuable, fluorinated building blocks.^23^ Aryl substituents were also tolerated and the products (**3s–3v**) were obtained in good to high yields. Moreover, acyclic ethers (**3w**), sterically demanding alkyl groups (**3x**) and also an unsubstituted α-position (**3y**) were tolerated in moderate to good yields. The functional group tolerance of this methodology was additionally demonstrated by an additive-based robustness screen (Table 2).^24^ The results show a generally high tolerance towards many functional groups (see ESI for more details†).

To further illustrate the potential of this reaction concept, we investigated the use of α-silyl amines^25^ as radical precursors for the synthesis of tertiary γ-amino butyric acids (GABAs).^26^ GABAs are of broad interest because of their biological relevance as neuromodulators and their physicochemical properties.^27^ Importantly, these structural motifs would be extremely difficult to access *via* the tandem Michael addition/Ireland–Claisen strategy, owing to the challenging generation of unstabilized α-amino carbanions.^28^ After optimizing the reaction conditions with α-silyl amine **4a** and allyl methacrylate (**2a**) as standard substrates (see ESI†), we continued with the exploration of the α-silyl amine scope (Table 3). In addition to morpholine (**5a**), other important N-heterocycles such as thiomorpholine (**5b**), and various N-substituted piperazines (**5c**, **5d** and **5g**) were successfully tested in good to excellent yields. Besides N-heterocycles, acyclic α-silyl amines also reacted in good yields to the desired products **5e** and **5f**. A complex fluoxetine (Prozac) derivative could be installed as well (**5h**), highlighting the potential of this method. Even a *N*-[α-(silyl)ethyl]-substituted piperidine was suitable in this transformation and gave the branched product **5i** in moderate yield. Overall, many functional groups including ethers (**5a**, **5g** and **5h**), thioethers (**5b**), tertiary amines (**5c**, **5d** and **5g**), bromo-substituted pyrimidines (**5d**) and amides (**5g**) were tolerated, making this method applicable for the synthesis of a wide range of diverse α,α-dialkylated GABAs. It is worth noting that most other methods for the synthesis of GABAs by radical addition to acrylates are either limited to anilines or protected amines.^29^

**Table 3:** α-Silyl amine scope for the synthesis of γ-amino butyric acids (GABAs)^a^

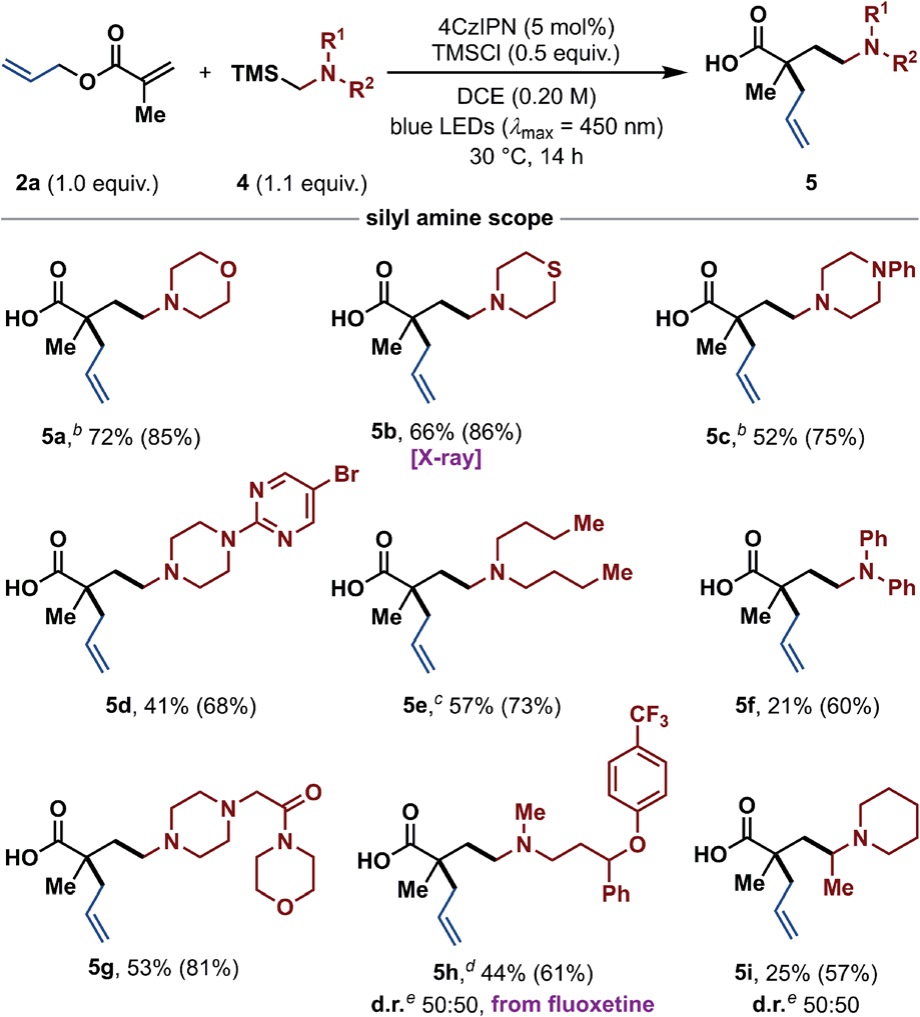

a Standard reaction conditions: **2** (0.30 mmol, 1.0 equiv.), **4** (1.1 equiv.), 4CzIPN (5 mol%), TMSCl (0.5 equiv.), DCE (0.20 M) at rt for 14 h under irradiation with blue LEDs (*λ*_max_ = 450 nm). Isolated yields are given. ^1^H NMR yields were determined with 1,3,5-trimethoxybenzene or mesitylene as internal standard and are given in parentheses.

b Isolated as methyl ester (see ESI).

c Performed in THF.

d Performed on a 0.10 mmol scale with 1.7 equiv. of α-silyl amine **4h**.

e Diastereomeric ratio was determined by ^1^H NMR spectroscopy of the isolated product. X-ray = see ESI for the crystal structure.

### Mechanistic studies

**Fig. 2:**
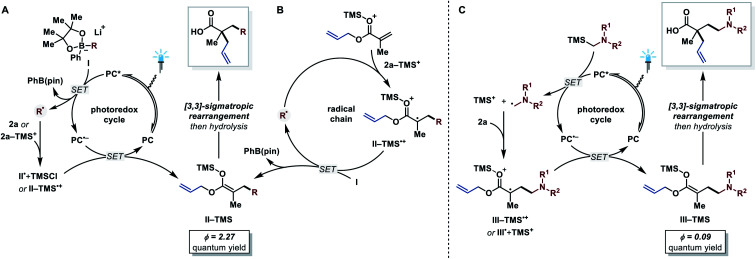
Proposed reaction mechanisms for the two reactions systems. (A) Photoredox mechanism and (B) radical chain mechanism with arylboronates as radical precursors. (C) Photoredox mechanism with α-silyl amines as radical precursors.

After demonstrating the substrate scope of this protocol, we turned our attention towards the reaction mechanism (Fig. 2). Based on previous reports,^15*d*,18^ the following photoredox mechanism was proposed for the transformation with arylboronates as radical precursors (Fig. 2A). Reductive quenching of the excited photocatalyst (*E*_1/2_(PC*/PC˙^−^) = +1.35 V *vs.* SCE in MeCN)^30^ by arylboronate complex **I** (for R = cyclohexyl, *E*_1/2_(**I˙+**/**I**) = +0.31 V *vs.* SCE in MeCN)^15*d*^ generates an alkyl radical and PhB(pin). The alkyl radical then adds to the allyl acrylate and reduction of the formed α-acyl radical **II˙** regenerates the photocatalyst (*E*_1/2_(PC/PC˙^−^) = −1.21 V *vs.* SCE in MeCN).^30^ Trapping of the ester enolate by TMSCl affords the silyl ketene acetal **II-TMS**, which finally undergoes the desired [3,3]-sigmatropic rearrangement. To test this hypothesis, various experiments were conducted. First, Stern–Volmer luminescence quenching studies revealed that only arylboronate complex **I** quenches the excited photocatalyst, which is consistent with the proposed reductive quenching pathway. The quantum yield of this transformation, however, was determined to be *Φ* = 2.27,^31^ which is not explained by a solely photoredox mechanism and hints towards a radical chain mechanism. Comparison of the redox potentials shows that a direct oxidation of arylboronate complex **I** by the respective α-acyl radical **II˙** is thermodynamically unfavorable (*E*_1/2_(**II˙**/**II−**) = approx. −0.6 V *vs.* SCE in MeCN).^32^ However, Lewis-acid activation of the α-acyl radical **II˙** with a TMS^+^ species has a dramatic effect on its oxidation potential (see species **II-TMS˙+**, *E*_1/2_(**II-TMS˙+**/**II-TMS**) = +0.46 V *vs.* SCE in MeCN).^33^ This opens up the possibility of direct oxidation of the arylboronate complex **I** by the activated α-acyl radical **II-TMS˙+** (Fig. 2B). We propose that in this reaction system both, the photoredox and the radical chain mechanism, are simultaneously operative (see ESI for more details†). Considering the higher oxidation potentials of α-silyl amines (*E*_1/2_(**4˙+**/**4**) = approx. +0.7 V *vs.* SCE in MeCN)^25*c*^ compared to arylboronates,^15*d*^ a radical chain mechanism is thermodynamically not feasible for these. The quantum yield of this reaction variant was determined to be *Φ* = 0.09,^31^ suggesting that a photo-independent radical chain process is negligible, although an inefficient chain cannot be excluded in principle. Furthermore, different behaviors of the two reaction systems were observed in TEMPO trapping and light on/off experiments, hinting towards a difference in the reaction mechanism (see ESI†). Based on these results, a solely photoredox mechanism is proposed (Fig. 2C). The α-silyl amine quenches the excited photocatalyst reductively (see Stern–Volmer quenching studies in the ESI†) and the resulting radical cation furnishes TMS^+^ and an α-amino alkyl radical after fragmentation.^25*c*^ This nucleophilic radical adds to the allyl acrylate in the presence of TMS^+^ to generate the first C(sp^3^)–C(sp^3^) bond. The formed α-acyl radical is reduced by the reduced state of the photocatalyst to afford the silyl ketene acetal intermediate. Subsequent [3,3]-sigmatropic Ireland–Claisen rearrangement gives the desired α,α-dialkylated γ-amino butyric acid product.

## Conclusions

We have developed a formal 1,2-dialkylation of α,β-unsaturated carboxylic acids, by combining modern photocatalytic-enabled RRPCO with the established [3,3]-sigmatropic Ireland-Claisen rearrangement. This transition-metal-free and redox-neutral protocol is especially efficient for the construction of tertiary carboxylic acids. Owing to the mild generation of the silyl ketene acetal intermediate, this protocol outperforms the classical Ireland–Claisen rearrangement in functional group tolerance. In addition to alkylboronic esters as readily available radical progenitors, α-silyl amines were also compatible, which led to the formation of valuable α,α-disubstituted γ-amino butyric acids (GABAs). Although, both systems rely on the same reaction design, they seem to proceed *via* different reaction mechanisms. While the alkylboronic acid variant primarily proceeds *via* a photoredox-induced radical chain mechanism, the α-silyl amine variant is proposed to proceed *via* a solely photoredox mechanism. Ultimately, we believe that this work showcases the still underexplored potential of the RRPCO concept as a mild alternative for the generation of carbanions, which should be employed in further transformations.

## Conflicts of interest

There are no conflicts to declare.

## Supplementary Material

SC-012-D0SC06385A-s001

SC-012-D0SC06385A-s002
