# Supplementary material for: Photoredox-enabled 1,2-dialkylation of α-substituted acrylates via Ireland–Claisen rearrangement
Source: Chem Sci. 2021 Jan 7;12(8):2816–22. doi: 10.1039/d0sc06385a (PMC8179405; doi:10.1039/d0sc06385a)

Electronic Supplementary Material (ESI) for Chemical Science.
This journal is © The Royal Society of Chemistry 2021

Photoredox-enabled 1,2-dialkylation of α-substituted acrylates *via* Ireland–Claisen rearrangement

Supporting Information

Roman Kleinmans, Leon E. Will, J. Luca Schwarz, and Frank Glorius*
*glorius@uni-muenster.de

Organisch-Chemisches Institut, Westfälische Wilhelms-Universität Münster, Corrensstraße 40, 48149 Münster, Germany

Table of Contents

1 General Information S2
2 Experimental Procedures and Characterization Data S4
2.1 Synthesis of Alkylboronic Acid Esters S4
2.2 Synthesis of Allyl Acrylates S6
2.3 Synthesis of *α*-Silyl Amines S20
2.4 Optimization Studies S23
2.5 Substrate Scope S28
2.6 Additive-Based Robustness Screen S46
2.7 Sensitivity Assessment S48
2.8 Crystal Structures S51
3 Mechanistic Analysis S55
3.1 UV/Vis Absorption Spectroscopy S55
3.2 Stern–Volmer Luminescence Quenching Analysis S56
3.3 Light On/Off Experiment S58
3.4 Quantum Yield Analysis S60
3.5 TEMPO Trapping Experiment S64
3.6 Decomposition Experiment: Primary *vs.* Secondary Alkylboronates S66
4 References S68
5 Spectra S71

1 General Information

Unless otherwise noted, reactions were carried out in cabinet-dry glassware under air. Reactions performed under an argon atmosphere were carried out in oven-dried glassware with oven-dried Teflon-coated magnetic stir bars. Solvents were purified by distillation over standard drying agents, and stored over molecular sieves under an argon atmosphere. Starting materials which were not synthesized in our laboratory, were obtained from commercial suppliers and used as received, unless otherwise noted. TMSCl was purchased from Sigma-Aldrich and was distilled over CaH_2 under an argon atmosphere. PhLi (1.9 M in dibutly ether, the concentration was confirmed by titration with diphenylacetic acid) was purchased from Sigma-Aldrich and used as received. The employed photocatalysts 4CzIPN,[1] $[Ir(dF(CF_3)ppy)_2(dtbbpy)](PF_6)$,[2] and $[Ir(ppy)_2(dtbbpy)](PF_6)$[3] were prepared following literature procedures.

Products were purified by column chromatography on Acros Organics silica gel (35–70 mesh) or by preparative thin-layer chromatography (PTLC) using PTLC-plates purchased from Analtech (L × W = 20 × 20 cm, layer thickness 1,000 µm). Suitable solvent mixtures for separation were identified by thin-layer chromatography (TLC) analysis on silica gel 60 F254 aluminum plates from Merck. Spots were visualized by irradiation with UV light (254 nm) or by staining in an alkaline $KMnO_4$ solution (+ heat). Carboxylic acid were also identified by a bromocresol green stain.

NMR spectra were recorded on a Bruker Avance II 300, Bruker Avance II 400, Agilent DD2 500 or on an Agilent DD2 600 spectrometer. Chemicals shifts (δ) are quoted in ppm downfield of tetramethylsilane. The residual solvent signals were used as references for ^{1}H and ^{13}C NMR spectra ($CDCl_3$: δ_H = 7.26 ppm, δ_C = 77.16 ppm). ^{19}F NMR spectra are referenced according to the proton resonance of tetramethylsilane as the primary reference for the unified chemical shift scale (IUPAC recommendation 2001). The multiplicity of all signals was described with standard abbreviations. Coupling constants (J) are quoted in Hz.

Samples for GC were filtered over a pad of silica with EtOAc before analysis if not stated otherwise (GC samples of the crude catalysis products were filtered over Celite®). GC-MS spectra were recorded on an Agilent Technologies 7890A GC-system (HP-5MS column: 0.25 mm × 30 m, film: 0.25 µm) with an Agilent 5975C VL MSD or an Agilent 5975 inert Mass Selective Detector (EI). The method indicated as '50_40' starts with an injection temperature T_0 (50 °C).After holding this temperature for 3 min, the column is heated by 40 °C/min to temperature T_1 (290 °C) and this temperature is held for an additional time. GC-FID analysis

was undertaken on an Agilent Technologies 6890A equipped with a HP-5 quartz column (0.32 mm × 30 m, film: 0.25 μm) using flame ionization detection (FID).

All photochemical reaction were performed in 10 mL Schlenk tubes (unless noted otherwise) under an argon atmosphere at room temperature. The reactions were carried out in a commercial EvoluChem™ PhotoRedOx Duo photobox with irradiation by EvoluChem HCK1021-01-008 blue LEDs (30 W, λ_{max} = 450 nm). The reaction temperature in this set-up was approx. 30 °C. Photochemical reactions which were performed in the commercial setup were irradiated with blue LEDs (5 W, λ_{max} = 455 nm), if not stated otherwise. Degassing of reactions was achieved by three freeze-pump-thaw cycles.

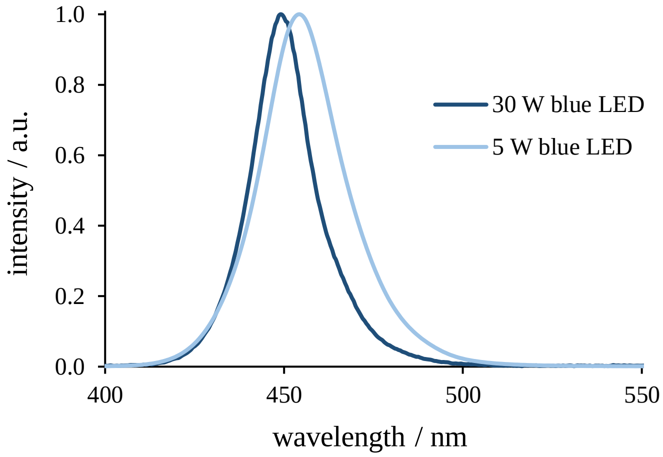

Figure S1. Emission spectra of EvoluChem 30 W blue LED and 5 W blue LED.

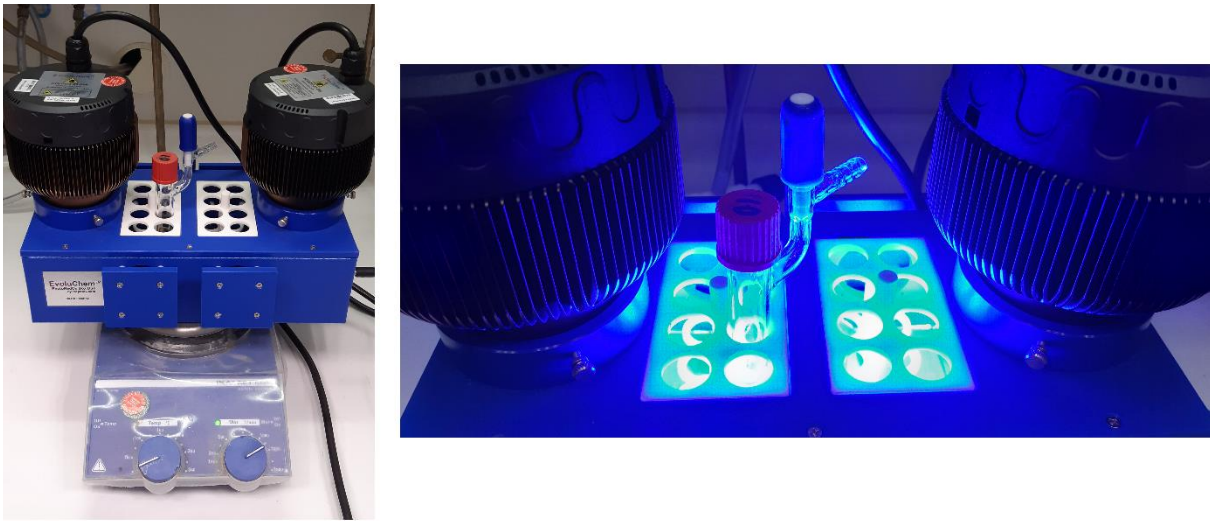

Figure S2. Photoreaction set-up. Commercial EvoluChem™ PhotoRedOx Duo photobox with irradiation by EvoluChem HCK1021-01-008 blue LEDs (30 W, λ_{max} = 450 nm).

2 Experimental Procedures and Characterization Data

2.1 Synthesis of Alkylboronic Acid Esters

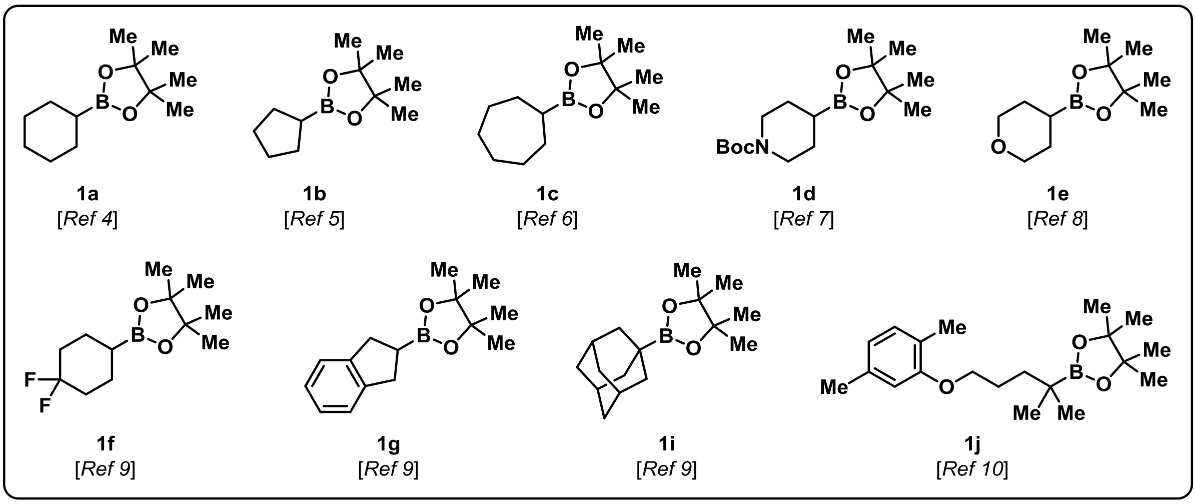

Alkylboronic esters **1a–1j** are known compounds and were prepared following literature procedures.[4–10]

1,3-Dioxoisoindolin-2-yl 2-ethylbutanoate (**S1h**): The title compound was prepared according to a literature procedure.[11] To a round bottom flask equipped with a Teflon-coated magnetic stir bar were added *N,N'*-dicyclohexylmethanediimine (DCC, 1.44 g, 7.0 mmol, 1.0 equiv), *N*-hydroxyphthalimide (NHPI, 1.14 g, 7.0 mmol, 1.0 equiv) and EtOAc (120 mL). The mixture was stirred at room temperature and 2-ethylbutanoic acid (0.88 mL, 7.0 mmol, 1.0 equiv) was added in one portion. After 2.5 h reaction time, the reaction mixture was filtered through a pad of Celite®, the pad was washed with EtOAc (2×), and the combined filtrates were concentrated *in vacuo*. Purification by silica gel column chromatography (5% EtOAc in pentane) gave the product (1.53 g, 84%) as a colorless solid.

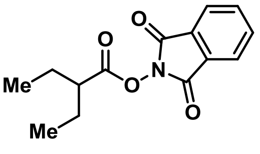

S1h

^{1}H NMR (400 MHz, $CDCl_3$) δ 7.83 – 7.76 (m, 2H), 7.75 – 7.68 (m, 2H), 2.54 (tt, J = 8.8, 5.4 Hz, 1H), 1.80 – 1.59 (m, 4H), 1.02 (t, J = 7.5 Hz, 6H); **^{13}C NMR** (101 MHz, $CDCl_3$) δ 172.2, 162.0, 134.7, 128.9, 123.8, 46.5, 25.2, 11.5; **HRMS** (ESI$^+$) calc'd for $[M+Na]^+$ 284.0893, found 284.0892; **R$_f$** (5% EtOAc in pentane) = 0.50.

4,4,5,5-Tetramethyl-2-(pentan-3-yl)-1,3,2-dioxaborolane (**1h**): The title compound was prepared according to a literature procedure.[8] To an oven-dried Schlenk flask equipped with a Teflon-coated magnetic stir bar were added 1,3-dioxoisoindolin-2-yl 2-ethylbutanoate (**S1h**, 1.31 g, 5.0 mmol, 1.0 equiv), $B_2(pin)_2$ (1.90 g, 7.5 mmol, 1.5 equiv), $Cu(acac)_2$ (261 mg, 1.0 mmol, 0.20 equiv), grinded $LiOH{\cdot}H_2O$ (3.15 g, 75.0 mmol, 15.0 equiv) and $MgCl_2$ (713 mg, 7.5 mmol, 1.5 equiv). The flask was evacuated and backfilled with argon three times and degassed dioxane/DMF (1/2, 40 mL) was added. The reaction was stirred at room temperature until the reaction turned dark brown (~15 min). The mixture was diluted with Et_2O, transferred into a separation funnel, and sat. aq. NH_4Cl was added. The organic layer was separated and the aq. layer was washed with Et_2O (2×). The combined org. layers were dried over Na_2SO_4 and concentrated *in vacuo*. Purification by silica gel chromatography (0.5% Et_2O in pentane) gave the product (371 mg, 37%) as a colorless liquid.

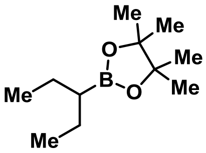

1h

^{1}H NMR (400 MHz, $CDCl_3$) δ 1.49 – 1.31 (m, 4H), 1.24 (s, 12H), 0.89 (t, *J* = 7.4 Hz, 6H), 0.86 – 0.79 (m, 1H); **^{13}C NMR** (101 MHz, $CDCl_3$) δ 82.9, 25.0, 24.1, 13.8; **^{11}B NMR** (128 MHz, $CDCl_3$) δ 34.7; **R_f** (0.5% Et_2O in pentane) = 0.29.

The carbon directly attached to the boron atom was not detected due to quadrupolar broadening. The spectral data are in agreement with literature data.[12]

2.2 Synthesis of Allyl Acrylates

Most of the synthesized allyl acrylates are volatile and should be handled carefully due to their toxicity.[13]

2.2.1 General Procedure 1

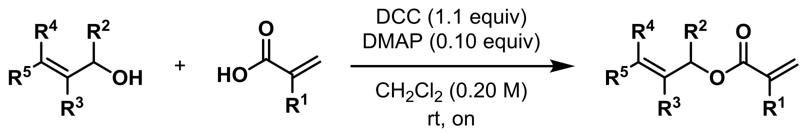

Allyl acrylates prepared by *General Procedure 1* were synthesized according to a modified literature procedure.[14] To a round bottom flask equipped with a Teflon-coated magnetic stir bar were added the appropriate acrylic acid (1.0 equiv), the appropriate allylic alcohol (1.1 equiv), DCC (1.1 equiv), DMAP (0.10 equiv), and CH_2Cl_2 (0.20 M). The reaction was stirred at room temperature overnight. The resulting suspension was filtered through a pad of Celite®, the pad was washed with CH_2Cl_2 (2×), the combined filtrates were washed with aq. HCl (2×, 1 M) and brine. The org. layer was concentrated *in vacuo*, and purification by silica gel chromatography (Et_2O/pentane) gave the product.

2.2.2 General Procedure 2

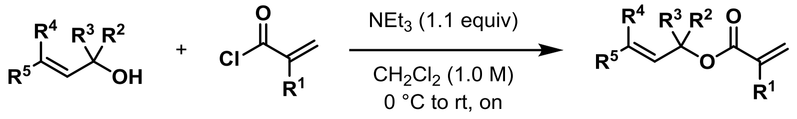

Allyl acrylates prepared by *General Procedure 2* were synthesized according to a modified literature procedure.[15] To an oven-dried Schlenk flask equipped with a Teflon-coated magnetic stir bar were added the appropriate allylic alcohol (1.0 equiv), triethylamine (1.1 equiv), and dry CH_2Cl_2 (1.0 M) under an argon atmosphere. The mixture was cooled to 0 °C, and the appropriate acryloyl chloride (1.2 equiv) was added dropwise. The reaction was allowed to warm up to room temperature and stirred overnight. Water was added to quench the reaction. The org. layer was washed with sat. aq. NH_4Cl, brine and concentrated *in vacuo*. The residue was purified by silica gel column chromatography (Et_2O/pentane) to afford the product.

2.2.3 General Procedure 3

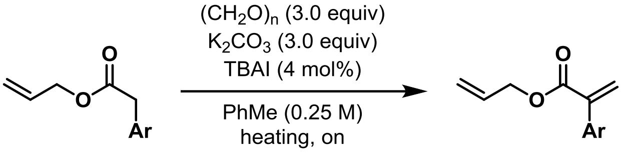

Allyl acrylates prepared by *General Procedure 3* were synthesized according to a modified literature procedure.[16] To an oven-dried Schlenk tube equipped with a Teflon-coated magnetic stir were added the appropriate allyl ester (1.0 equiv), paraformaldehyde (3.0 equiv), K_2CO_3 (3.0 equiv), tetrabutylammonium iodide (4 mol%), and dry toluene (0.25 M) under an argon atmosphere. The reaction mixture was stirred and heated overnight. The reaction was quenched with water and extracted with EtOAc. The combined org. layers were washed with brine and concentrated *in vacuo*. The residue was purified by silica gel column chromatography (Et_2O/pentane) to afford the desired product.

3-Methylbut-2-en-1-yl methacrylate (2b): The title compound was prepared from methacrylic acid (0.84 mL, 10 mmol, 1.0 equiv), prenol (1.1 mL, 11 mmol, 1.1 equiv), DCC (2.27 g, 11 mmol, 1.1 equiv), DMAP (122 mg, 1.0 mmol, 0.10 equiv), and CH_2Cl_2 (50 mL) according to *General Procedure 1*. Purification by silica gel column chromatography (1% to 3% Et_2O in pentane) gave the product (1.16 g, 75%) as a colorless liquid.

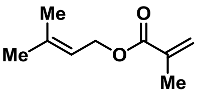

2b

^{1}H NMR (400 MHz, $CDCl_3$) δ 6.09 (dq, J = 2.0, 1.0 Hz, 1H), 5.56 – 5.49 (m, 1H), 5.42 – 5.31 (m, 1H), 4.64 (d, J = 7.1 Hz, 2H), 1.95 – 1.92 (m, 3H), 1.76 (s, 3H), 1.72 (s, 3H); **^{13}C NMR** (101 MHz, $CDCl_3$) δ 167.6, 138.9, 136.7, 125.3, 118.9, 61.8, 25.9, 18.5, 18.2; **HRMS** (ESI$^+$) calc'd for $[M+Na]^+$ 177.0886, found 177.0884; **R_f** (3% Et_2O in pentane) = 0.46.

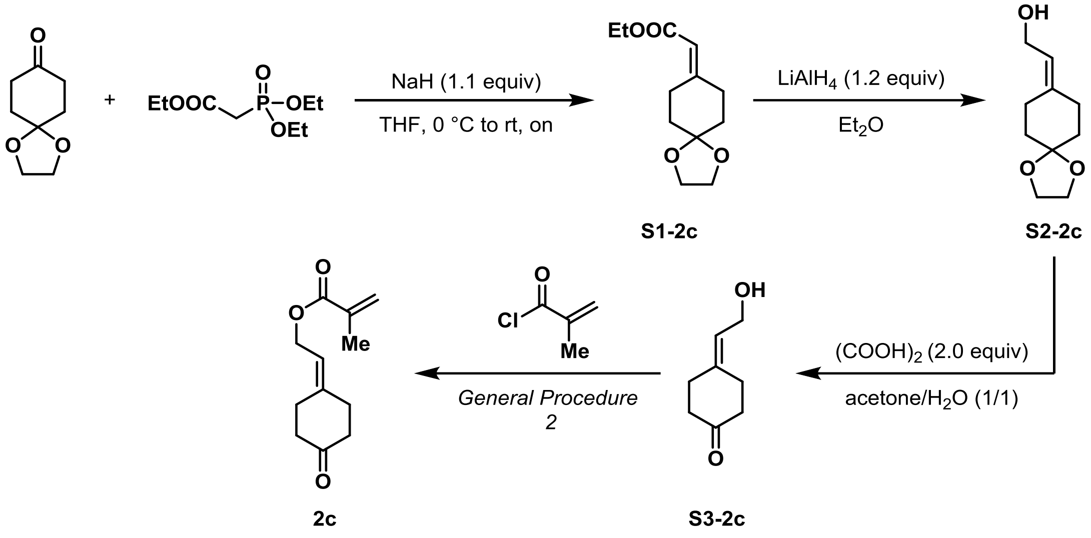

Ethyl 2-(1,4-dioxaspiro[4.5]decan-8-ylidene)acetate (**S1-2c**)**:** The title compound was prepared according to a modified literature procedure.[17] To an oven-dried Schlenk flask equipped with a Teflon-coated magnetic stir bar were added NaH (880 mg, 22.0 mmol, 1.1 equiv), and dry THF (80 mL) under an argon atmosphere. The suspension was cooled to 0 °C, and ethyl 2-(diethoxyphosphoryl)acetate (4.4 mL, 22 mmol, 1.1 equiv) was added dropwise. The mixture was allowed to warm up to room temperature, and stirred for 30 min at this temperature. Subsequently, 1,4-dioxaspiro[4.5]decan-8-one (3.12 g, 20.0 mmol, 1.0 equiv) was added, and the reaction mixture was stirred overnight. The reaction was quenched by addition of sat. aq. $NaHCO_3$, the layers were separated, and the aq. layer was extracted with EtOAc (3×). The combined org. layers were washed with brine, dried over Na_2SO_4, and concentrated *in vacuo*. Purification by silica gel column chromatography (10% EtOAc in pentane) gave the product (4.22 g, 94%) as a colorless oil.

^{1}H NMR (400 MHz, $CDCl_3$) δ 5.65 (t, *J* = 1.2 Hz, 1H), 4.13 (q, *J* = 7.1 Hz, 2H), 3.98 – 3.94 (m, 4H), 2.99 (ddd, *J* = 7.9, 5.0, 1.2 Hz, 2H), 2.36 (ddd, *J* = 8.0, 5.2, 1.2 Hz, 2H), 1.81 – 1.70 (m, 4H), 1.26 (t, *J* = 7.1 Hz, 3H); **^{13}C NMR** (101 MHz, $CDCl_3$) δ 166.7, 160.3, 114.4, 108.1, 64.6, 59.8, 35.9, 35.1, 34.7, 26.2, 14.4 – one signal is missing due to overlap; **R_f** (10% EtOAc in pentane) = 0.40.

The spectral data are in agreement with literature data.[18]

2-(1,4-Dioxaspiro[4.5]decan-8-ylidene)ethan-1-ol (**S2-2c**)**:** The title compound was prepared according to a modified literature procedure.[17] To an oven-dried Schlenk flask equipped with a Teflon-coated magnetic stir bar were added $LiAlH_4$ (240 mg, 6.0 mmol, 0.6 equiv) and dry

Et_2O under an argon atmosphere. The suspension was cooled to −78 °C, and ethyl 2-(1,4-dioxaspiro[4.5]decan-8-ylidene)acetate (**S1-2c**, 2.26 g,10.0 mmol, 1.0 equiv, as a solution in dry Et_2O) was added dropwise. The reaction was allowed to warm up to room temperature, and after 2 h another portion of $LiAlH_4$ (240 mg, 6.0 mmol, 0.6 equiv) was added. The mixture was stirred overnight, cooled to 0 °C, and carefully quenched with H_2O. The layers were separated, and the aq. layer was extracted with EtOAc (3×), washed with brine, and concentrated *in vacuo*. Purification by silica gel column chromatography (60% EtOAc in pentane) gave the product (1.40 g, 76%) as a colorless oil.

^{1}H NMR (400 MHz, $CDCl_3$) δ 5.40 (t, J = 7.0 Hz, 1H), 4.12 (d, J = 7.0 Hz, 2H), 3.97 – 3.92 (m, 4H), 2.35 – 2.21 (m, 4H), 1.72 – 1.63 (m, 4H); **^{13}C NMR** (101 MHz, $CDCl_3$) δ 141.3, 122.0, 108.8, 64.5, 58.8, 36.0, 35.5, 33.6, 25.3 – one signal is missing due to overlap; **R_f** (60% EtOAc in pentane) = 0.39.

The spectral data are in agreement with literature data.[18]

4-(2-Hydroxyethylidene)cyclohexan-1-one (**S3-2c**)**:** The title compound was prepared according to a literature procedure.[19] To a round bottom flask were added 2-(1,4-dioxaspiro[4.5]decan-8-ylidene)ethan-1-ol (**S2-2c**, 1.40 g, 7.6 mmol, 1.0 equiv), oxalic acid (1.37 g, 15.2 mmol, 2.0 equiv), and acetone/H_2O (1/1, 20 mL). The reaction was stirred overnight at room temperature and excess oxalic acid was consumed by addition of solid $NaHCO_3$. The mixture was filtered, and the residue was washed with Et_2O. The layers were separated, and the aq. layer was extracted with Et_2O (2×). The combined org. layers were dried over Na_2SO_4 and concentrated *in vacuo*. Purification by silica gel column chromatography (60% EtOAc in pentane) gave the product (673 mg, 63%) as a colorless oil.

^{1}H NMR (400 MHz, $CDCl_3$) δ 5.61 (t, J = 6.9 Hz, 1H), 4.21 (d, J = 6.9 Hz, 2H), 2.59 – 2.49 (m, 4H), 2.47 – 2.39 (m, 4H); **^{13}C NMR** (101 MHz, $CDCl_3$) δ 211.2, 138.1, 124.3, 58.9, 41.4, 40.7, 34.1, 26.3; **R_f** (60% EtOAc in pentane) = 0.35.

The spectral data are in agreement with literature data.[19]

2-(4-Oxocyclohexylidene)ethyl methacrylate (**2c**)**:** The title compound was prepared from 4-(2-hydroxyethylidene)cyclohexan-1-one (**S3-2c**, 660 mg, 4.7 mmol, 1.0 equiv), methacryloyl chloride (0.54 mL, 5.6 mmol, 1.2 equiv), triethylamine (0.72 mL, 5.2 mmol, 1.1 equiv), and dry

CH_2Cl_2 (5.0 mL) according to *General Procedure 2*. Purification by silica gel column chromatography (20% Et_2O in pentane) gave the product (504 mg, 51%) as colorless liquid.

^{1}H NMR (400 MHz, $CDCl_3$) δ 6.13 – 6.07 (m, 1H), 5.61 – 5.53 (m, 2H), 4.70 (d, J = 7.1 Hz, 2H), 2.62 (t, J = 7.4, 6.0, 1.4 Hz, 2H), 2.53 (t, J = 6.9 Hz, 2H), 2.48 – 2.40 (m, 4H), 1.94 (t, J = 1.3 Hz, 3H); **^{13}C NMR** (101 MHz, $CDCl_3$) δ 210.8, 167.4, 140.7, 136.5, 125.7, 119.5, 60.9, 41.2, 40.5, 34.0, 26.4, 18.5; **HRMS** (ESI$^+$) calc'd for $[M+Na]^+$ 231.0992, found 231.0991; **R_f** (20% Et_2O in pentane) = 0.25.

But-3-en-2-yl methacrylate (**2d**)**:** The title compound was prepared from methacrylic acid (0.84 mL, 10 mmol, 1.0 equiv), but-3-en-2-ol (0.96 mL, 11 mmol, 1.1 equiv), DCC (2.27 g, 11.0 mmol, 1.1 equiv), DMAP (122 mg, 1.0 mmol, 0.10 equiv), and CH_2Cl_2 (50 mL) according to *General Procedure 1*. Purification by silica gel column chromatography (2% Et_2O in pentane) gave the product (0.423 g, 31%) as a colorless liquid.

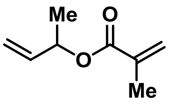

2d

^{1}H NMR (400 MHz, $CDCl_3$) δ 6.14 – 6.09 (m, 1H), 5.87 (ddd, J = 17.3, 10.5, 5.7 Hz, 1H), 5.55 (p, J = 1.6 Hz, 1H), 5.44 – 5.36 (m, 1H), 5.26 (dt, J = 17.3, 1.4 Hz, 1H), 5.14 (dt, J = 10.6, 1.3 Hz, 1H), 1.94 (dd, J = 1.6, 1.0 Hz, 3H), 1.35 (d, J = 6.5 Hz, 3H); **^{13}C NMR** (101 MHz, $CDCl_3$) δ 166.8, 137.9, 136.8, 125.4, 115.7, 71.3, 20.1, 18.5; **HRMS** (ESI$^+$) calc'd for $[M+Na]^+$ 163.0730, found 163.0729; **R_f** (2% Et_2O in pentane) = 0.70.

The spectral data are in agreement with literature data.[20]

2-Methylbut-3-en-2-yl methacrylate (**2e**)**:** The title compound was prepared from 2-methylbut-3-en-2-ol (1.0 mL, 10 mmol, 1.0 equiv), methacryloyl chloride (1.2 mL, 12 mmol, 1.2 equiv), triethylamine (1.5 mL, 11 mmol, 1.1 equiv), and dry CH_2Cl_2 (10 mL) according to *General Procedure 2*. Purification by silica gel column chromatography (1% Et_2O in pentane) gave the product (570 mg, 37%) as colorless liquid.

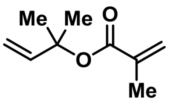

2e

^{1}H NMR (400 MHz, $CDCl_3$) δ 6.10 (dd, J = 17.5, 10.9 Hz, 1H), 6.03 (dt, J = 2.0, 1.0 Hz, 1H), 5.52 – 5.48 (m, 1H), 5.19 (dd, J = 17.5, 0.8 Hz, 1H), 5.08 (dd, J = 10.8, 0.8 Hz, 1H), 1.91 (t, J = 1.3 Hz, 3H), 1.56 (s, 6H); **^{13}C NMR** (101 MHz, $CDCl_3$) δ 166.5, 142.8, 137.8, 124.8, 112.7, 80.9, 26.6, 18.5; **GC-MS: t_R** (50_40): 5.2 min; **EI-MS: m/z** (%): 111.1 (4), 70.0 (7), 69.0 (100),

68.0 (20), 67.0 (12), 53.0 (8), 43.1 (7), 41.1 (62), 40.1 (5), 39.0 (21); **R$_f$** (1% Et_2O in pentane) = 0.21.

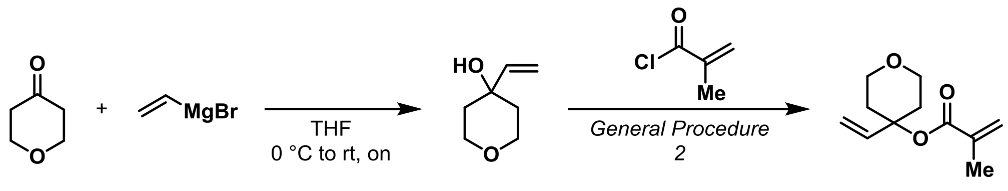

S2f

2f

4-Vinyltetrahydro-2*H*-pyran-4-ol (**S2f**)**:** The title compound was prepared according to a literature procedure.[21] To an oven-dried Schlenk flask equipped with a Teflon-coated magnetic stir bar were added tetrahydro-4*H*-pyran-4-one (0.74 mL, 8.0 mmol, 1.0 equiv), and THF (8.0 mL) under an argon atmosphere. The solution was cooled to 0 °C, and vinyl magnesium bromide (1.0 M in THF, 9.6 mL, 9.6 mmol, 1.2 equiv) was added dropwise. The reaction was allowed to warm up to room temperature and stirred overnight. Sat. aq. NH_4Cl was added to quench the reaction, and the resulting mixture was diluted with Et_2O. The layers were separated, and the aq. layer was extracted with Et_2O (2×). The combined org. layers were dried over Na_2SO_4 and concentrated *in vacuo*. Purification by silica gel column chromatography (30% EtOAc in pentane) gave the product (678 mg, 66%) as a colorless liquid.

H NMR (400 MHz, $CDCl_3$) δ 5.95 (dd, J = 17.4, 10.7 Hz, 1H), 5.26 (dd, J = 17.4, 1.0 Hz, 1H), 5.09 (dd, J = 10.7, 1.0 Hz, 1H), 3.81 (td, J = 11.2, 2.6 Hz, 2H), 3.77 – 3.69 (m, 2H), 1.81 (ddd, J = 14.8, 10.9, 5.0 Hz, 2H), 1.65 (s, 1H), 1.50 (dq, J = 14.0, 2.6 Hz, 2H); **^{13}C NMR** (101 MHz, $CDCl_3$) δ 145.2, 112.5, 69.4, 63.8, 37.6; **HRMS** (ESI^+) calc'd for $[M+Na]^+$ 151.0730, found 151.0730; **R$_f$** (30% EtOAc in pentane) = 0.23.

4-Vinyltetrahydro-2*H*-pyran-4-yl methacrylate (**2f**)**:** The title compound was prepared from 4-vinyltetrahydro-2*H*-pyran-4-ol (**S2f** ,576 mg, 4.5 mmol, 1.0 equiv), methacryloyl chloride (0.53 mL, 5.4 mmol, 1.2 equiv), triethylamine (0.69 mL, 5.0 mmol, 1.1 equiv), and dry CH_2Cl_2 (4.5 mL) according to *General Procedure 2*. Purification by silica gel column chromatography (10% Et_2O in pentane) gave the product (375 mg, 43%) as a colorless liquid.

^{1}H NMR (400 MHz, $CDCl_3$) δ 6.19 – 6.06 (m, 2H), 5.57 – 5.52 (m, 1H), 5.23 (d, J = 5.4 Hz, 1H), 5.20 (s, 1H), 3.79 (dt, J = 11.8, 4.1 Hz, 2H), 3.70 (td, J = 11.2, 2.4 Hz, 2H), 2.23 (dq, J = 14.4, 2.7 Hz, 2H), 1.96 – 1.85 (m, 5H); **^{13}C NMR** (101 MHz, $CDCl_3$) δ 166.1, 140.7, 137.3,

125.3, 114.8, 79.0, 63.8, 35.2, 18.5; **HRMS** (ESI$^+$) calc'd for $[M+Na]^+$ 219.0992, found 219.0992; **R$_f$** (10% Et_2O in pentane) = 0.28.

2-Chloroallyl methacrylate (2g): The title compound was prepared from methacrylic acid (0.84 mL, 10 mmol, 1.0 equiv), 2-chloroprop-2-en-1-ol (0.88 mL, 11 mmol, 1.1 equiv), DCC (2.27 g, 11.0 mmol, 1.1 equiv), DMAP (122 mg, 1.0 mmol, 0.10 equiv), and CH_2Cl_2 (50 mL) according to *General Procedure 1*. Purification by silica gel column chromatography (2% Et_2O in pentane) gave the product (1.34 g, 83%) as a colorless liquid.

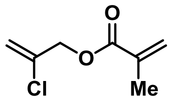

2g

^{1}H NMR (400 MHz, $CDCl_3$) δ 6.19 – 6.16 (m, 1H), 5.64 – 5.60 (m, 1H), 5.48 – 5.45 (m, 1H), 5.41 – 5.37 (m, 1H), 4.72 – 4.70 (m, 2H), 1.96 (dd, J = 1.7, 1.0 Hz, 3H); **^{13}C NMR** (101 MHz, $CDCl_3$) δ 166.5, 136.1, 135.8, 126.5, 114.7, 66.2, 18.3; **HRMS** (ESI$^+$) calc'd for $[M+Na]^+$ 183.0183, found 183.0183; **R$_f$** (2% Et_2O in pentane) = 0.50.

(*E*)-Hex-2-en-1-yl methacrylate (2h): The title compound was prepared from methacrylic acid (0.67 mL, 8.0 mmol, 1.0 equiv), (*E*)-hex-2-en-1-ol (881 mg, 8.8 mmol, 1.1 equiv), DCC (1.81 g, 8.8 mmol, 1.1 equiv), DMAP (98 mg, 1.0 mmol, 0.10 equiv), and CH_2Cl_2 (40 mL) according to *General Procedure 1*. Purification by silica gel column chromatography (1% Et_2O in pentane) gave the product (471 mg, 35%) as a colorless liquid.

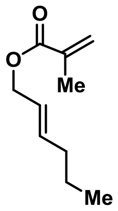

2h

^{1}H NMR (400 MHz, $CDCl_3$) δ 6.11 (dq, J = 1.9, 1.0 Hz, 1H), 5.78 (td, J = 14.6, 6.7, 1.2 Hz, 1H), 5.65 – 5.52 (m, 2H), 4.62 – 4.55 (m, 2H), 2.04 (q, J = 7.8 Hz, 2H), 1.94 (dd, J = 1.6, 1.0 Hz, 3H), 1.41 (h, J = 7.4 Hz, 2H), 0.90 (t, J = 7.4 Hz, 3H); **^{13}C NMR** (101 MHz, $CDCl_3$) δ 167.4, 136.6, 136.3, 125.5, 124.1, 65.6, 34.5, 22.2, 18.5, 13.8; **HRMS** (ESI$^+$) calc'd for $[M+Na]^+$ 191.1043, found 191.1041; **R$_f$** (1% Et_2O in pentane) = 0.37.

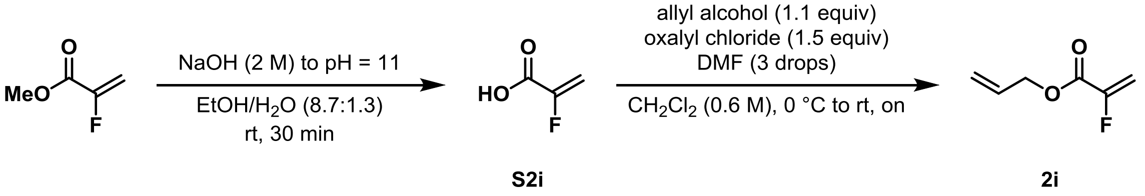

2-Fluoroacrylic acid (S2i): The title compound was prepared according to a literature procedure.[22] In a round bottom flask equipped with a Teflon-coated magnetic stir bar was dissolved 2-fluoroacrylic acid methyl ester (1.81 mL, 20.0 mmol, 1.0 equiv) in EtOH/H_2O (8.7/1.3, 20 mL), and the pH was adjusted with aq. NaOH (2 M) to be pH = 11. The mixture was stirred for 30 min at room temperature. The solvent was removed and the residue was treated with Et_2O (40 mL). Aq. HCl (6 M) was added dropwise until the solid was dissolved. The layers were separated, the aq. layer was extracted with Et_2O (2×), and the combined org. layers were dried over Na_2SO_4. The solvent was removed *in vacuo* to afford the product (1.17 g, 66%) as a colorless solid.

^{1}H NMR (400 MHz, $CDCl_3$) δ 7.62 (bs, 1H), 5.84 (dd, *J* = 42.7, 3.4 Hz, 1H), 5.48 (dd, *J* = 12.6, 3.4 Hz, 1H), **^{13}C NMR** (101 MHz, $CDCl_3$) δ 165.0 (d, *J* = 37.5 Hz), 152.6 (d, *J* = 260.3 Hz), 105.2 (d, *J* = 14.7 Hz); **^{19}F NMR** (377 MHz, $CDCl_3$) δ −118.2.

The spectral data are in agreement with literature data.[22]

Allyl 2-fluoroacrylate (2i): The title compound was prepared according to a modified literature procedure.[23] To an oven-dried Schlenk flask equipped with a Teflon-coated magnetic stir bar were added 2-fluoroacrylic acid (**S2i**, 0.54 g, 6.0 mmol, 1.0 equiv), DMF (3 drops), and CH_2Cl_2 (10 mL) under an argon atmosphere. The mixture was cooled to 0 °C, and oxalyl chloride (0.77 mL, 9.0 mmol, 1.5 equiv, attention: gas evolution) was added dropwise. The solution was allowed to warm up to room temperature. After 45 min, the reaction was cooled to 0 °C, and allyl alcohol (0.45 mL, 6.6 mmol, 1.1 equiv), and triethylamine (1.25 mL, 9.0 mmol, 1.5 equiv) were added. The reaction was allowed to warm up to room temperature and stirred overnight. Water was then added to quench the reaction. The org. layer was washed with sat. aq. NH_4Cl, brine and concentrated *in vacuo*. The residue was purified by silica gel column chromatography (3% Et_2O in pentane) to afford the product (357 mg, 46%) as a colorless liquid.

^{1}H NMR (400 MHz, $CDCl_3$) δ 5.96 (ddt, *J* = 17.1, 10.4, 5.8 Hz, 1H), 5.71 (dd, *J* = 43.3, 3.3 Hz, 1H), 5.43 – 5.26 (m, 3H), 4.74 (dt, *J* = 5.8, 1.4 Hz, 2H); **^{13}C NMR** (101 MHz, $CDCl_3$) δ 160.2 (d, *J* = 36.8 Hz), 153.4 (d, *J* = 262.3 Hz), 131.3, 119.4, 103.0 (d, *J* = 15.0 Hz), 66.5; **^{19}F NMR** (377 MHz, $CDCl_3$) δ −117.1; **GC-MS: t_R** (50_40): 3.7 min; **EI-MS: m/z** (%): 73.0 (100), 57.0 (25), 45.0 (37), 41.1 (32), 39.0 (27); **R_f** (3% Et_2O in pentane) = 0.60.

Allyl 2-phenylacrylate (**2j**)**:** The title compound was prepared from atropic acid (1.78 g, 10.0 mmol, 1.0 equiv), allyl alcohol (0.75 mL, 11 mmol, 1.1 equiv), DCC (2.27 g, 11 mmol, 1.1 equiv), DMAP (122 mg, 1.0 mmol, 0.10 equiv), and CH_2Cl_2 (50 mL) according to *General Procedure 1*. Purification by silica gel column chromatography (3% Et_2O in pentane) gave the product (1.26 g, 67%) as a colorless liquid.

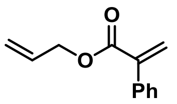

2j

^{1}H NMR (400 MHz, $CDCl_3$) δ 7.47 – 7.40 (m, 2H), 7.39 – 7.30 (m, 3H), 6.40 (d, J = 1.2 Hz, 1H), 6.04 – 5.93 (m, 1H), 5.92 (d, J = 1.2 Hz, 1H), 5.40 – 5.31 (m, 1H), 5.29 – 5.23 (m, 1H), 4.74 (ddd, J = 5.6, 1.5 Hz, 2H); **^{13}C NMR** (101 MHz, $CDCl_3$) δ 166.5, 141.4, 136.8, 132.1, 128.4, 128.3, 128.2, 127.1, 118.4, 65.8; **R_f** (3% Et_2O in pentane) = 0.50.

The spectral data are in agreement with literature data.[24]

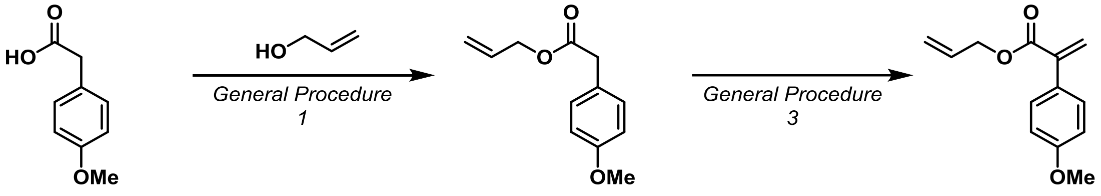

S2k **2k**

Allyl 2-(4-methoxyphenyl)acetate (**S2k**)**:** The title compound was prepared from 2-(4-methoxyphenyl)acetic acid (1.66 g, 10.0 mmol, 1.0 equiv), allyl alcohol (0.75 mL, 11 mmol, 1.1 equiv), DCC (2.27 g, 11 mmol, 1.1 equiv), DMAP (122 mg, 1.0 mmol, 0.10 equiv), and CH_2Cl_2 (50 mL) according to *General Procedure 1*. Purification by silica gel column chromatography (7% Et_2O in pentane) gave the product (1.23 g, 60%) as a colorless liquid.

^{1}H NMR (400 MHz, $CDCl_3$) δ 7.24 – 7.18 (m, 2H), 6.89 – 6.83 (m, 2H), 5.90 (ddt, J = 17.2, 10.4, 5.7 Hz, 1H), 5.33 – 5.17 (m, 2H), 4.59 (dt, J = 5.6, 1.4 Hz, 2H), 3.80 (s, 3H), 3.59 (s, 2H); **^{13}C NMR** (101 MHz, $CDCl_3$) δ 171.7, 158.9, 132.2, 130.4, 126.2, 118.3, 114.1, 65.5, 55.4, 40.6; **HRMS** (ESI^+) calc'd for $[M+Na]^+$ 229.0835, found 229.0833; **R_f** (5% Et_2O in pentane) = 0.39.

Allyl 2-(4-methoxyphenyl)acrylate (**2k**)**:** The title compound was prepared from allyl 2-(4-methoxyphenyl)acetate (**S2k**, 1.03 g, 5.0 mmol, 1.0 equiv), paraformaldehyde (450 mg, 15 mmol, 3.0 equiv), K_2CO_3 (2.07 g, 15 mmol, 3.0 equiv), tetrabutylammonium iodide (74 mg,

0.20 mmol, 4 mol%), and dry toluene (20 mL) according to *General Procedure 3*. The mixture was stirred at 70 °C overnight. Purification by silica gel column chromatography (6% Et_2O in pentane) gave the product (0.600 g, 55%) as a colorless liquid.

^{1}H NMR (400 MHz, $CDCl_3$) δ 7.40 – 7.35 (m, 2H), 6.91 – 6.86 (m, 2H), 6.30 (d, J = 1.2 Hz, 1H), 5.98 (ddt, J = 17.2, 10.4, 5.6 Hz, 1H), 5.86 (d, J = 1.2 Hz, 1H), 5.39 – 5.23 (m, 2H), 4.73 (dt, J = 5.7, 1.4 Hz, 2H), 3.82 (s, 3H); **^{13}C NMR** (101 MHz, $CDCl_3$) δ 166.7, 159.6, 140.7, 132.1, 129.5, 129.1, 125.4, 118.3, 113.5, 65.7, 55.3; **HRMS** (ESI$^+$) calc'd for $[M+Na]^+$ 241.0835, found 241.0833, **R_f** (6% Et_2O in pentane) = 0.51.

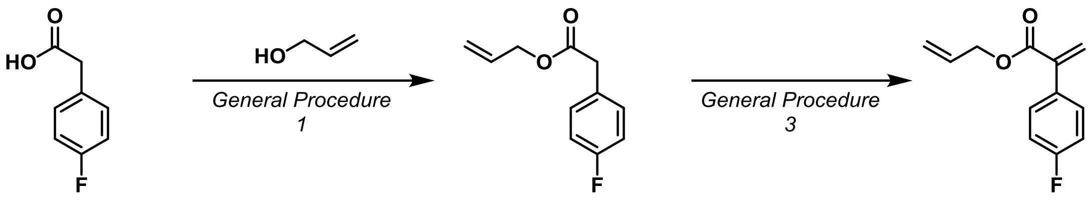

S2l **2l**

Allyl 2-(4-fluorophenyl)acetate (**S2l**)**:** The title compound was prepared from 2-(4-fluorophenyl)acetic acid (1.54 g, 10.0 mmol, 1.0 equiv), allyl alcohol (0.75 mL, 11 mmol, 1.1 equiv), DCC (2.27 g, 11 mmol, 1.1 equiv), DMAP (122 mg, 1.0 mmol, 0.10 equiv), and CH_2Cl_2 (50 mL) according to *General Procedure 1*. Purification by silica gel column chromatography (5% Et_2O in pentane) gave the product (1.09 g, 56%) as a colorless liquid.

^{1}H NMR (400 MHz, $CDCl_3$) δ 7.29 – 7.22 (m, 2H), 7.05 – 6.98 (m, 2H), 5.90 (ddt, J = 17.2, 10.5, 5.7 Hz, 1H), 5.32 – 5.19 (m, 2H), 4.63 – 4.57 (m, 2H), 3.62 (s, 2H); **^{13}C NMR** (101 MHz, $CDCl_3$) δ 171.2, 162.2 (d, J = 245.5 Hz), 132.1, 131.0 (d, J = 8.1 Hz), 129.8 (d, J = 3.1 Hz), 118.5, 115.6 (d, J = 21.4 Hz), 65.7, 40.6; **^{19}F NMR** (377 MHz, $CDCl_3$) δ −115.7; **HRMS** (ESI$^+$) calc'd for $[M+Na]^+$ 217.0635, found 217.0634; **R_f** (5% Et_2O in pentane) = 0.40.

Allyl 2-(4-fluorophenyl)acrylate (**2l**)**:** The title compound was prepared from allyl 2-(4-fluorophenyl)acetate (**S2l** ,971 mg, 5.0 mmol, 1.0 equiv), paraformaldehyde (0.45 g, 15 mmol, 3.0 equiv), K_2CO_3 (2.07 g, 15 mmol, 3.0 equiv), tetrabutylammonium iodide (74 mg, 0.20 mmol, 4 mol%), and dry toluene (20 mL) according to *General Procedure 3*. The mixture was stirred at 70 °C overnight. Purification by silica gel column chromatography (3% Et_2O in pentane) gave the product (433 mg, 42%) as a colorless liquid.

^{1}H NMR (400 MHz, $CDCl_3$) δ 7.44 – 7.37 (m, 2H), 7.08 – 7.01 (m, 2H), 6.42 – 6.37 (m, 1H), 6.05 – 5.92 (m, 1H), 5.92 – 5.88 (m, 1H), 5.41 – 5.24 (m, 2H), 4.73 (dt, J = 5.6, 1.4 Hz, 2H); **^{13}C NMR** (101 MHz, $CDCl_3$) δ 166.3, 162.9 (d, J = 247.6 Hz), 140.4, 132.8 (d, J = 3.4 Hz), 132.1, 130.3 (d, J = 8.1 Hz), 127.2 (d, J = 1.1 Hz), 118.6, 115.2 (d, J = 21.5 Hz), 65.9; **^{19}F NMR** (377 MHz, $CDCl_3$) δ −113.8; **HRMS** (ESI$^+$) calc'd for $[2M+Na]^+$ 435.1378, found 435.1374, **R$_f$** (4% Et_2O in pentane) = 0.58.

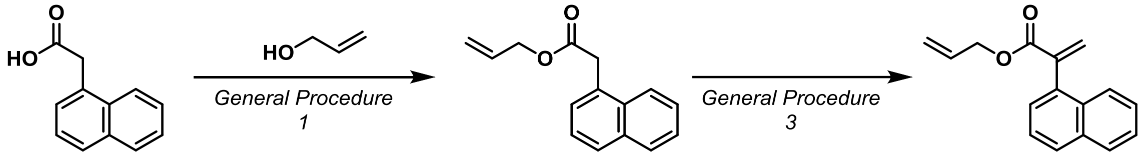

Allyl 2-(naphthalen-1-yl)acetate (S2m): The title compound was prepared from 2-(naphthalen-1-yl)acetic acid (1.86 g, 10.0 mmol, 1.0 equiv), allyl alcohol (0.75 mL, 11 mmol, 1.1 equiv), DCC (2.27 g, 11 mmol, 1.1 equiv), DMAP (122 mg, 1.0 mmol, 0.10 equiv), and CH_2Cl_2 (50 mL) according to *General Procedure 1*. Purification by silica gel column chromatography (4% Et_2O in pentane) gave the product (1.84 g, 82%) as a colorless liquid.

^{1}H NMR (300 MHz, $CDCl_3$) δ 8.07 – 7.99 (m, 1H), 7.93 – 7.86 (m, 1H), 7.86 – 7.78 (m, 1H), 7.60 – 7.48 (m, 2H), 7.47 – 7.42 (m, 2H), 5.89 (ddt, J = 17.2, 10.4, 5.6 Hz, 1H), 5.30 – 5.16 (m, 2H), 4.62 (dt, J = 5.6, 1.5 Hz, 2H), 4.12 (s, 2H); **^{13}C NMR** (76 MHz, $CDCl_3$) δ 171.3, 133.9, 132.2, 132.1, 130.6, 128.8, 128.2, 128.1, 126.5, 125.9, 125.6, 123.9, 118.3, 65.6, 39.2; **HRMS** (ESI$^+$) calc'd for $[M+Na]^+$ 249.0886, found 249.0884; **R$_f$** (4% Et_2O in pentane) = 0.35.

Allyl 2-(naphthalen-1-yl)acrylate (2m): The title compound was prepared from allyl 2-(naphthalen-1-yl)acetate (**S2m**, 1.66 g, 7.3 mmol, 1.0 equiv), paraformaldehyde (0.72 g, 24 mmol, 3.3 equiv), K_2CO_3 (3.32 g, 24.0 mmol, 3.3 equiv), tetrabutylammonium iodide (118 mg, 0.32 mmol, 4 mol%), and dry toluene (20 mL). The reaction mixture was stirred at 50 °C overnight. Because of incomplete conversion, the mixture was further heated to 70 °C for 3 h. Purification by silica gel column chromatography (4% Et_2O in pentane) gave the product (800 mg, 46%) as a colorless liquid.

^{1}H NMR (400 MHz, $CDCl_3$) δ 7.90 – 7.83 (m, 2H), 7.80 – 7.72 (m, 1H), 7.52 – 7.44 (m, 3H), 7.41 – 7.35 (m, 1H), 6.77 – 6.74 (m, 1H), 5.94 – 5.91 (m, 1H), 5.91 – 5.80 (m, 1H), 5.21 – 5.12

(m, 2H), 4.70 – 4.64 (m, 2H); **^{13}C NMR** (101 MHz, $CDCl_3$) δ 166.7, 140.9, 135.3, 133.5, 132.0, 131.9, 130.1, 128.7, 128.4, 127.1, 126.3, 126.0, 125.5, 125.3, 118.1, 65.7; **HRMS** (ESI^+) calc'd for $[M+Na]^+$ 261.0886, found 261.0883; **R$_f$** (4% Et_2O in pentane) = 0.55.

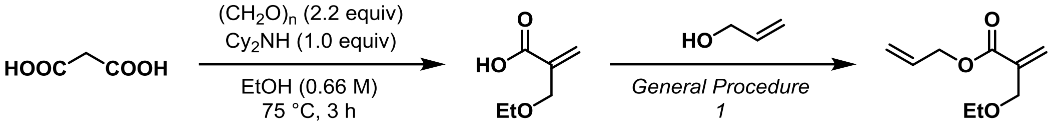

S2n **2n**

2-(Ethoxymethyl)acrylic acid (**S2n**): The title compound was prepared according to a literature procedure.[25] To a Schlenk tube equipped with a Teflon-coated magnetic stir bar were added malonic acid (1.04 g, 10.0 mmol, 1.0 equiv), para formaldehyde (660 mg, 22.0 mmol, 2.2 equiv), and EtOH (15 mL). To the stirred mixture was added dicyclohexylamine (2.0 mL, 10 mmol, 1.0 equiv), and the reaction was subsequently heated to 75 °C for 3 h. The solvent was removed *in vacuo*, and purification by acid/base work-up with subsequent silica gel column chromatography (1% HCOOH/20% EtOAc in pentane) afforded the product (936 mg, 72%) as a colorless solid. The product was directly used in the next step.

Allyl 2-(ethoxymethyl)acrylate (**2n**): The title compound was prepared from 2-(ethoxymethyl)acrylic acid (**S2n**, 650 mg, 5.0 mmol, 1.0 equiv), allyl alcohol (0.38 mL, 5.5 mmol, 1.1 equiv), DCC (1.13 g, 5.5 mmol, 1.1 equiv), DMAP (61 mg, 0.50 mmol, 0.10 equiv), and CH_2Cl_2 (25 mL) according to *General Procedure 1*. Purification by silica gel column chromatography (3% Et_2O in pentane) gave the product (479 mg, 56%) as a colorless liquid.

^{1}H NMR (400 MHz, $CDCl_3$) δ 6.35 – 6.29 (m, 1H), 6.00 – 5.86 (m, 2H), 5.38 – 5.20 (m, 2H), 4.66 (dt, J = 5.6, 1.5 Hz, 2H), 4.19 (t, J = 1.6 Hz, 2H), 3.55 (q, J = 7.0 Hz, 2H), 1.22 (t, J = 7.0 Hz, 3H); **^{13}C NMR** (101 MHz, $CDCl_3$) δ 165.7, 137.5, 132.2, 125.9, 118.2, 68.8, 66.4, 65.4, 15.3; **HRMS** (ESI^+) calc'd for $[M+Na]^+$ 193.0835, found 193.0833; **R$_f$** (5% Et_2O in pentane) = 0.37.

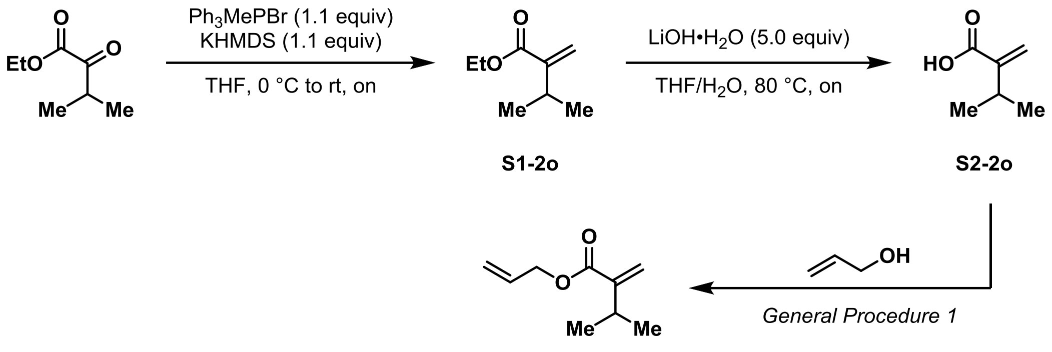

Ethyl 3-methyl-2-methylenebutanoate (S1-2o): The title compound was prepared according to a modified literature procedure.[26] To an oven-dried Schlenk flask equipped with a Teflon-coated magnetic stir bar were added Ph_3MePBr (3.93 g, 11.0 mmol, 1.1 equiv), and dry THF (20 mL) under an argon atmosphere. The mixture was cooled to −78 °C, KHMDS (1.0 M in dry THF, 2.19 g, 11 mmol, 1.1 equiv) was added dropwise and was then stirred for 15 min at this temperature. The mixture was allowed to warm up to room temperature and stirred for 1 h. After re-cooling to −78 °C ethyl 3-methyl-2-methylenebutanoate (1.5 mL, 10 mmol, 1.0 equiv) was added dropwise. The reaction was again allowed to warm up to room temperature and was stirred overnight. Aq. HCl (2 M) was added to quench the reaction, and the mixture was extracted with EtOAc. The combined org. layers were dried over Na_2SO_4 and concentrated *in vacuo*. Purification by silica gel column chromatography (0.5% Et_2O in pentane) gave the product (1.15 g, 80%) as a colorless liquid.

^{1}H NMR (400 MHz, $CDCl_3$) δ 6.10 (s, 1H), 5.50 (t, J = 1.3 Hz, 1H), 4.21 (q, J = 7.1 Hz, 2H), 2.81 (heptd, J = 6.9, 1.3 Hz, 1H), 1.30 (t, J = 7.1 Hz, 3H), 1.08 (d, J = 6.8 Hz, 6H).

The spectral data are in agreement with literature data.[27]

3-Methyl-2-methylenebutanoic acid (S2-2o): The title compound was prepared according to a modified literature procedure.[26] To a Schlenk flask equipped with a Teflon-coated magnetic stir bar were added ethyl 3-methyl-2-methylenebutanoate (**S1-2o**, 1.15 g, 8.0 mmol, 1.0 equiv), $LiOH•H_2O$ (1.68 g, 40 mmol, 5.0 equiv), and THF/H_2O (1/1, 32 mL). The reaction mixture stirred at 80 °C overnight. The reaction was allowed to cool to room temperature and was washed with Et_2O (3×). The aq. layer was acidified with aq. HCl (2 M) and extracted with EtOAc (4×). The combined org. layers were dried over Na_2SO_4 and concentrated *in vacuo* to

afford the product (664 mg, 73%) as a slightly orange liquid. The product was used in the next step without further purification.

Allyl 3-methyl-2-methylenebutanoate (**2o**)**:** The title compound was prepared from 3-methyl-2-methylenebutanoic acid (**S2-2o**, 570 mg, 5.0 mmol, 1.0 equiv), allyl alcohol (0.38 mL, 5.5 mmol, 1.1 equiv), DCC (1.13 g, 5.5 mmol, 1.1 equiv), DMAP (61 mg, 0.50 mmol, 0.10 equiv), and CH_2Cl_2 (25 mL) according to *General Procedure 1*. Purification by silica gel column chromatography (1% Et_2O in pentane) gave the product (586 mg, 76%) as a colorless liquid.

^{1}H NMR (400 MHz, $CDCl_3$) δ 6.15 (s, 1H), 6.02 – 5.89 (m, 1H), 5.54 (s, 1H), 5.38 – 5.20 (m, 2H), 4.66 (d, J = 5.6 Hz, 2H), 2.82 (hept, J = 6.7 Hz, 1H), 1.09 (d, J = 6.9 Hz, 6H); **^{13}C NMR** (101 MHz, $CDCl_3$) δ 167.2, 147.2, 132.5, 122.1, 118.1, 65.3, 29.5, 22.0; **R_f** (2% Et_2O in pentane) = 0.50.

The spectral data are in agreement with literature data.[28]

Allyl acrylate (**2p**)**:** The title compound was prepared from allyl alcohol (0.89 mL, 13 mmol, 1.3 equiv), methacryloyl chloride (0.82 mL, 10 mmol, 1.0 equiv), triethylamine (7.0 mL, 50 mmol, 5.0 equiv), and dry CH_2Cl_2 (20 mL) according to *General Procedure 2*. Purification by silica gel column chromatography (2% Et_2O in pentane) gave the product (340 mg, 30%) as a colorless liquid.

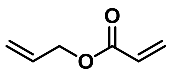

2p

^{1}H NMR (400 MHz, $CDCl_3$) δ 6.43 (dd, J = 17.3, 1.5 Hz, 1H), 6.14 (dd, J = 17.4, 10.4 Hz, 1H), 5.95 (ddt, J = 17.2, 10.4, 5.7 Hz, 1H), 5.84 (dd, J = 10.4, 1.4 Hz, 1H), 5.37 – 5.22 (m, 2H), 4.66 (dt, J = 5.7, 1.4 Hz, 2H); **^{13}C NMR** (101 MHz, $CDCl_3$) δ 166.0, 132.2, 131.1, 128.4, 118.4, 65.3; **R_f** (2% Et_2O in pentane) = 0.31.

The spectral data are in agreement with literature data.[29]

2.3 Synthesis of *α*-Silyl Amines

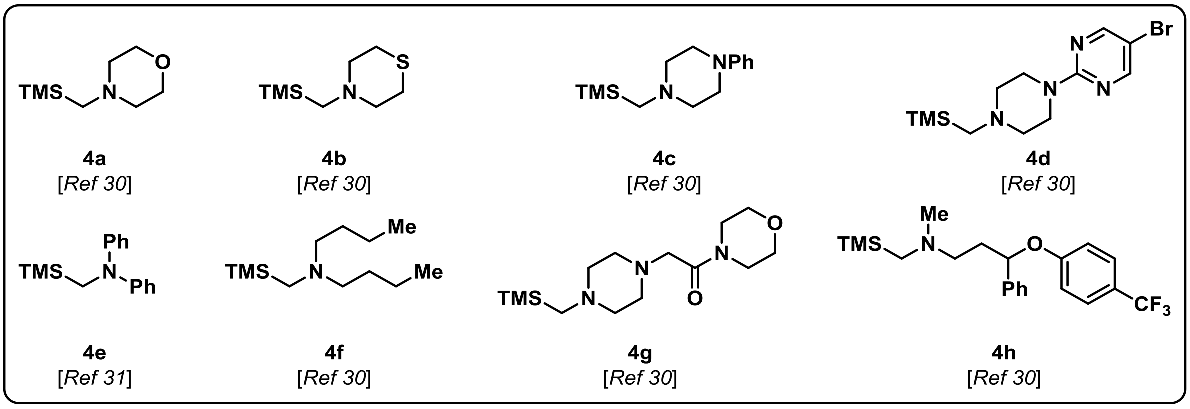

α-Silyl amines were prepared according to literature procedures.[30,31]

1-Morpholino-2-(4-((trimethylsilyl)methyl)piperazin-1-yl)ethan-1-one (**4g**): The title compound was prepared according to a literature procedure.[30] To an oven-dried Schlenk tube equipped with a Teflon-coated stir bar were added (chloromethyl)trimethylsilane (0.35 mL, 2.5 mmol, 1.0 equiv), dry DMF (2.5 mL), and 1-morpholino-2-(piperazin-1-yl)ethan-1-one (1.07 g, 5.0 mmol, 2.0 equiv) under an argon atmosphere. The reaction mixture was heated to 90 °C and stirred overnight. The reaction was quenched with brine, and the aq. layer was extracted with Et_2O (3×). The combined org. layers were washed with water (2×) and brine. The organic layer was dried over Na_2SO_4, and the solvent was removed *in vacuo* to afford the product (277 mg, 37%) as a colorless solid.

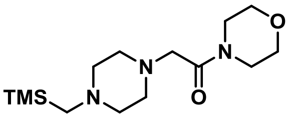

4g

^{1}H NMR (300 MHz, $CDCl_3$) δ 3.74 – 3.53 (m, 8H), 3.14 (s, 2H), 2.62 – 2.25 (m, 8H), 1.91 (s, 2H), 0.05 (s, 9H); **^{13}C NMR** (75 MHz, $CDCl_3$) δ 168.4, 67.2, 67.2, 61.5, 57.0, 53.3, 50.8, 46.4, 42.3, −1.0; **HRMS** (ESI$^+$) calc'd for $[M+H]^+$ 300.2102, found 300.2118.

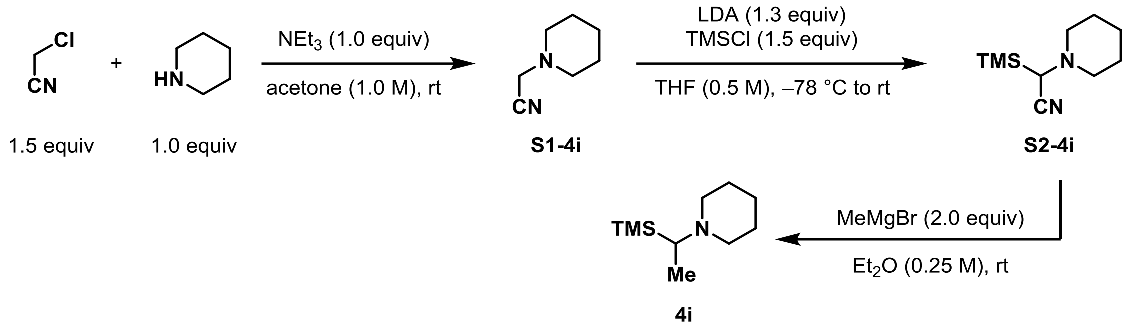

2-(Piperidin-1-yl)acetonitrile (**S1-4i**)**:** The title compound was prepared following a literature procedure.[30] To a solution of piperidine (3.95 mL, 40.0 mmol, 1.0 equiv) in acetone (40 mL) was added Et_3N (5.60 mL, 40.0 mmol, 1.0 equiv) at room temperature. The mixture was stirred for 10 min before chloroacetonitrile (3.80 mL, 60.0 mmol, 1.5 equiv) was added. The reaction mixture was stirred at room temperature overnight, quenched with sat. aq. NH_4Cl and extracted with Et_2O. The combined organics were dried over $MgSO_4$, and concentrated *in vacuo* to afford the product (4.28 g, 34.5 mmol, 86%) as an orange solid.

^{1}H NMR (400 MHz, $CDCl_3$) δ 3.51 (s, 2H), 2.54 (t, J = 5.4 Hz, 4H), 1.64 (p, J = 5.7 Hz, 4H), 1.51 – 1.41 (m, 2H); **^{13}C NMR** (101 MHz, $CDCl_3$) δ 114.9, 53.2, 47.0, 25.7, 23.3; **HRMS** (ESI^+) calc'd for $[M+Na]^+$ 147.0893, found 147.0892.

2-(Piperidin-1-yl)-2-(trimethylsilyl)acetonitrile (**S2-4i**): The title compound was prepared following a modified literature procedure.[32] *n*-BuLi (1.33 M in hexane, 9.78 mL, 13.0 mmol, 1.3 equiv) was added dropwise to a solution of diisopropylamine (1.83 mL, 13.0 mmol, 1.3 equiv) in dry THF (10 mL) at −78 °C. This LDA solution was stirred for 30 min at the same temperature and was then transferred to a pre-cooled solution of 2-(piperidin-1-yl)acetonitrile (1.24 g, 10.0 mmol, 1.0 equiv) and chlorotrimethylsilane (1.91 mL, 15.0 mmol, 1.5 equiv) in dry THF (10 mL) at −78 °C. The reaction mixture was stirred for 3 h at −78 °C, then slowly warmed to room temperature and stirred overnight. The reaction was quenched with sat. aq. NH_4Cl and extracted with Et_2O. The combined organics were washed with brine, dried over $MgSO_4$ and concentrated under reduced pressure to afford the title compound (1.96 g, 10.0 mmol, >99%) as an orange oil.

^{1}H NMR (400 MHz, $CDCl_3$) δ 3.02 (s, 1H), 2.61 – 2.49 (m, 2H), 2.46 – 2.36 (m, 2H), 1.68 – 1.50 (m, 4H), 1.50 – 1.34 (m, 2H), 0.22 (s, 9H); **^{13}C NMR** (101 MHz, $CDCl_3$) δ 117.5, 54.3, 50.7, 26.3, 23.5, −2.2; **HRMS** (ESI^+) calc'd for $[M+Na]^+$ 219.1288, found 219.1293.

1-(1-(Trimethylsilyl)ethyl)piperidine (4i): The title compound was prepared following a modified literature procedure.[30] To a solution of 2-(piperidin-1-yl)-2-(trimethylsilyl)acetonitrile (981 mg, 5.0 mmol, 1.0 equiv) in dry Et_2O (20 mL) was added methylmagnesium bromide (3.0 M in Et_2O, 3.33 mL, 10.0 mmol, 2.0 equiv) dropwise at room temperature. The reaction mixture was stirred for 1 h, quenched with sat. aq. NH_4Cl and extracted with Et_2O. The combined organics were dried over $MgSO_4$, concentrated under reduced pressure and purified by silica gel column chromatography (50% Et_2O in pentane) to afford the product (581 mg, 62%) as a slightly brown liquid.

^{1}H NMR (400 MHz, $CDCl_3$) δ 2.60 – 2.50 (m, 2H), 2.49 – 2.35 (m, 2H), 2.16 – 2.10 (m, 1H), 1.61 – 1.49 (m, 4H), 1.44 – 1.32 (m, 2H), 1.04 (d, J = 7.4 Hz, 3H), 0.04 (s, 9H); **^{13}C NMR** (101 MHz, $CDCl_3$) δ 52.6, 52.5, 26.8, 24.5, 9.0, −1.5; **HRMS** (ESI$^+$) calc'd for $[M+H]^+$ 186.1673, found 186.1672; **R_f** (Et_2O) 0.25.

2.4 Optimization Studies

2.4.1 Optimization – Alkyl–B(pin) Variant

To an oven-dried 10 mL Schlenk tube equipped with a Teflon-coated magnetic stir bar were added 2-cyclohexyl-4,4,5,5-tetramethyl-1,3,2-dioxaborolane (**1a**), and dry THF (2.0 mL) under an argon atmosphere. The solution was cooled to 0 °C, and a PhLi solution (1.9 M in dibutyl ether) was added dropwise while stirring. The mixture was stirred for 45 min at 0 °C and was then allowed to warm up to room temperature for further 15 min. If a THF/MeCN solvent mixture (0.05 M) was employed, dry MeCN (2.0 mL) was directly added. Otherwise, THF was removed *in vacuo* (*via* Schlenk line), and the resulting colorless solid was dissolved in the respective solvent or solvent mixture. To this solution were added allyl methacrylate (**2a**, 27.1 µL, 0.20 mmol, 1.0 equiv), 4CzIPN (7.9 mg, 0.010 mmol, 5 mol%), and TMSCl. The reaction mixture was degassed by three freeze-pump-thaw cycles. Subsequently, the reaction was stirred and irradiated with blue LEDs (30 W, λ_{max} = 450 nm) for 20 h. The solvent was removed through a stream of argon, 1,3,5-trimethoxybenzene (33.6 mg, 0.20 mmol, 1.0 equiv) was added as internal standard, and the ^{1}H NMR yield was determined (the resulting suspension after addition of $CDCl_3$ was filtered over a pad of Celite® directly into the NMR tube).

Table S1. Optimization studies – alkyl–B(pin) variant.

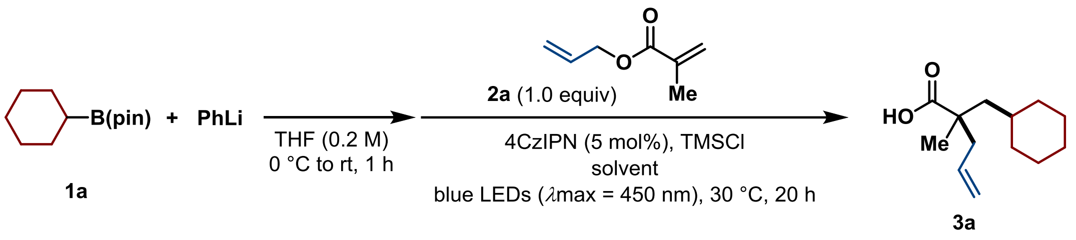

entry	equiv **1a**	equiv PhLi	equiv TMSCl	solvent (M)	comment	% yield **3a**
1	1.1	1.2	2.0	THF (0.2)		50
2	1.1	1.2	2.0	DCE (0.13)		42
3	1.1	1.2	2.0	DMA (0.13)		55
4	1.1	1.2	2.0	MeCN (0.13)		50
5	1.1	1.2	3.0	MeCN (0.05)		56
6	1.1	1.2	3.0	1,4-dioxane (0.05)		n.d.
7	1.5	1.65	3.0	THF (0.67)	0.30 mmol scale	55
8	1.5	1.65	3.0	MeCN (0.05)		70

9	1.5	1.65	3.0	MeCN (0.05)	50 °C no ventilation	65
10	1.5	1.65	3.0	THF/MeCN (0.05)		73
11	1.5	1.65	3.0	THF/MeCN (0.05)	10 mol% PC	~75
12	1.5	1.65	3.0	THF/MeCN (0.1)		63
13	1.5	1.65	2.0	THF/MeCN (0.05)		~45
14	1.5	1.65	5.0	THF/MeCN (0.05)		66
15	1.5	1.65		THF/MeCN (0.05)	3.0 equiv TMSOTf	n.d.
16	1.5	1.65		THF/MeCN (0.05)	BSA (3.0 equiv)/ TMSCl (0.5 equiv)	n.d.
17	1.5	1.65	3.0	THF/MeCN (0.05)	$[Ru(bpy)_3](BF_4)_2$ (2 mol%)	35
18	1.5	1.65	3.0	THF/MeCN (0.05)	**Ir-1** (2 mol%)	63
19	**2.0**	**1.8**	**3.0**	**THF/MeCN (0.05)**		**78**
20	2.0	1.8	3.0	THF/MeCN (0.05)	3/1 solvent mixture	71

Ir-1: $[Ir(dF(CF_3)ppy)_2(dtbbpy)](PF_6)$. If not stated otherwise, THF/MeCN was a 1/1 solvent mixture. BSA = bis(trimethylsilyl)acetamide. n.d. = not detected.

Table S2. Deviation of the optimized reaction conditions – alkyl–B(pin) variant

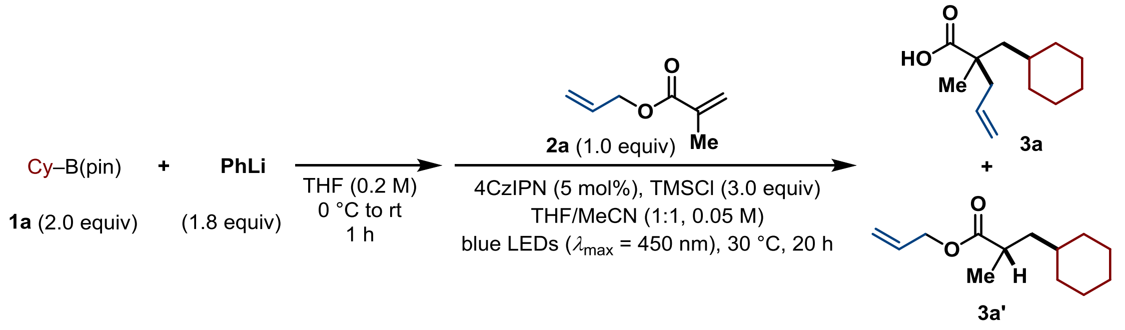

entry	deviation from standard conditions	% yield **3a**	% yield **3a'**
1	none	76 (74)	14
2	THF instead of MeCN/THF (1:1)	57	-
3	$[Ir(dF\text{-}CF_3(ppy))_2dtbbpy](PF_6)$ (**Ir-1**) (2 mol%) instead of 4CzIPN	68	18
4	$[Ir(ppy)_2dtbbpy](PF_6)$ (**Ir-2**) (2 mol%) instead of 4CzIPN	59	18
5	2.2 equiv PhLi	51	20
6	TBSCl instead TMSCl	n.d.	-
7	no TMSCl	n.d.	-
8	higher concentration (0.1 M)	76	10
9	10 mol% photocatalyst	79	9
**10**	**MeCN** instead of MeCN/THF (1:1)	**83** (77)	**17**
11	no light (40 °C), no photocatalyst *or* no PhLi	n.d.	n.d.

Giese-type product **3a'** was not characterized, but due to full conversion of starting material **2a** (by GC-MS) the ^1H NMR signals of the typical chemical shift of the allyl ester ($COO–\mathbf{CH_2}–(\mathbf{CH}CH_2)$) were utilized for quantification. Isolated yield is given in parentheses.

2.4.2 *Optimization – α-Silyl Amine Variant*

To an oven-dried 10 mL Schlenk tube equipped with a Teflon-coated magnetic stir was added 4CzIPN (3.9 mg, 0.005 mmol, 5 mol%), and the tube was evacuated and backfilled with argon three times. Subsequently, dry DCE (0.5 mL), 4-((trimethylsilyl)methyl)morpholine (**4a**), allyl methacrylate (**2a**, 13.4 µL, 0.10 mmol, 1.0 equiv), and if stated the respective additive were added under an argon atmosphere. If specified, the reactions were degassed by carrying out three freeze-pump-thaw cycles. The reaction mixture was stirred and irradiated with blue LEDs (30 W, λ_{max} = 450 nm) for 14 h at room temperature. The solvent was removed through a stream of argon, mesitylene (14.0 µL, 0.10 mmol, 1.0 equiv) was added as internal standard, and the ^{1}H NMR yield was determined.

Table S3. Optimization studies – *α*-silyl amine variant.

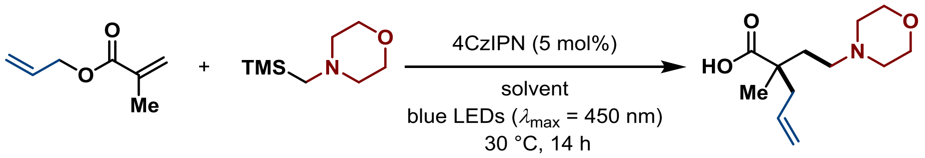

entry	equiv **4a**	solvent (M)	comment	% yield **5a**
1	2.0	THF (0.2 M)		55
2	2.0	CH_2Cl_2 (0.2 M)		67
3	2.0	DCE (0.2 M)		70
4	2.0	MeCN (0.2 M)		12
5	2.0	CH_2Cl_2 (0.2 M)	**Ir-1** (2 mol%)	26
6	2.0	CH_2Cl_2 (0.2 M)	**Ir-2** (2 mol%)	39
7	2.0	DCE (0.2 M)	+1.0 equiv K_3PO_4	72
8	2.0	DCE (0.2 M)	+3.0 equiv K_3PO_4	66
9	2.0	DCE (0.2 M)	+1.0 equiv K_2HPO_4	71
10	2.0	DCE (0.2 M)	+1.0 equiv $NaHCO_3$	61
11	2.0	DCE (0.2 M)	+1.0 equiv Na_2CO_3	72
12	2.0	DCE (0.2 M)	+2.0 equiv Na_2CO_3	64
13	2.0	DCE (0.2 M)	+1.0 equiv K_2CO_3	67
14	2.0	DCE (0.2 M)	+1.0 equiv Cs_2CO_3	34
15	1.5	DCE (0.2 M)		63
16	1.0	DCE (0.2 M)		72
17	1.1	DCE (0.2 M)	+0.25 equiv TMSCl	73

18	1.1	DCE (0.2 M)	+0.5 equiv TMSCl	76
19	1.1	DCE (0.2 M)	+1.0 equiv TMSCl	70
20	1.1	DCE (0.2 M)	10 mol% PC	70
21	1.1	DMA (0.2 M)	degassed	81
22	1.1	DCE (0.2 M)	degassed	82
**23[a]**	**1.1**	**DCE (0.2 M)**	**+0.5 equiv TMSCl, degassed**	**85**
24	1.1	DCE (0.2 M)	no light	n.d.
25	1.1	DCE (0.2 M)	40 °C	n.d.

Ir-1: [Ir(dF(CF_3)ppy)$_2$dtbbpy](PF_6); **Ir-2**: [Ir(ppy)$_2$dtbbpy](PF_6). [a] Performed on a 0.30 mmol scale.

Table S4. Deviation of the optimized reaction conditions – *α*-silyl amine variant.

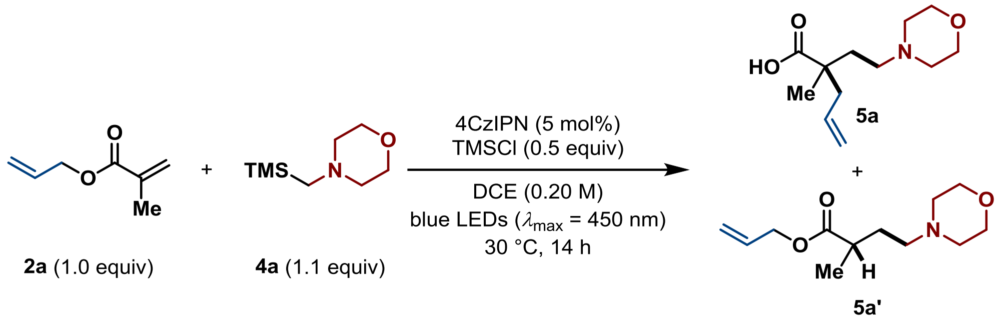

entry	deviation from standard conditions	% yield **5a**	% yield **5a'**
1[a]	none	85 (73)	15
2	THF instead of DCE	79	21
3	MeCN instead of DCE	47	39
4	[Ir(dF(CF_3)ppy)$_2$dtbbpy](PF_6) (**Ir-1**) (2 mol%) instead of 4CzIPN	51	39
5	[Ir(ppy)$_2$dtbbpy](PF_6) (**Ir-2**) (2 mol%) instead of 4CzIPN	66	28
6	2.0 equiv silyl amine **4a**	71	22
7[a]	no TMSCl	71	19
8	lower concentration (0.1 M)	74	18
9	10 mol% photocatalyst	76	17
10	no light (40 °C) *or* no photocatalyst	n.d.	n.d.

All reactions were degassed by three freeze-pump-thaw cycles. Giese-type product **5a'** was not characterized, but due to full conversion of starting material **2a** (by GC-MS) the ^{1}H NMR signals of the typical chemical shift of the allyl ester (COO–**CH**$_2$–(**CHCH**$_2$) were utilized for quantification. [a] Performed on a 0.30 mmol scale.

2.4.3 Unsuccessful Radical Precursors

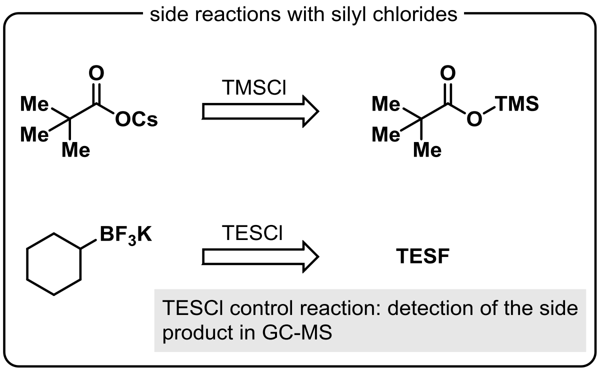

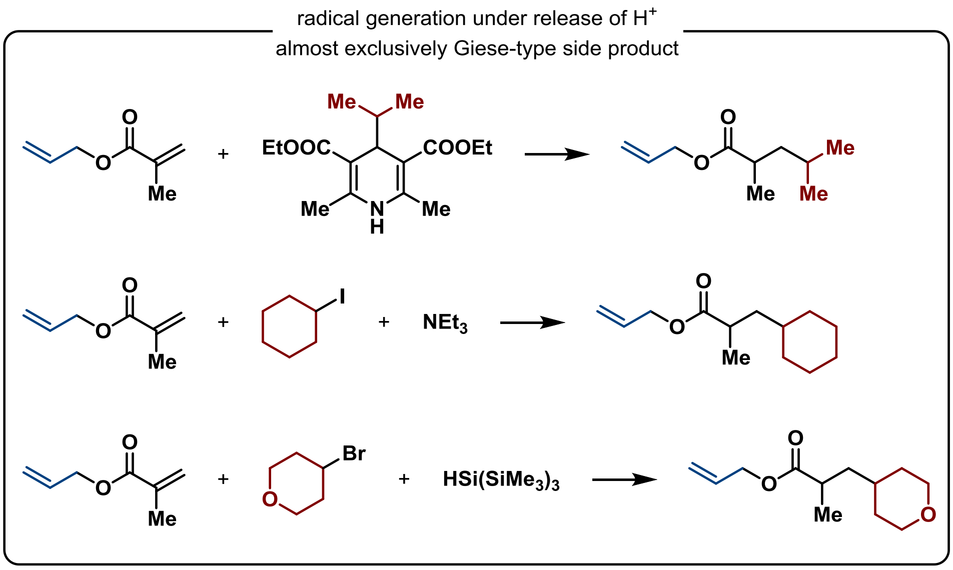

2.5 Substrate Scope

2.5.1 Photoredox-enabled 1,2-dialkylation of α-substituted acrylates – Alkyl–B(pin) Variant (General Procedure 4)

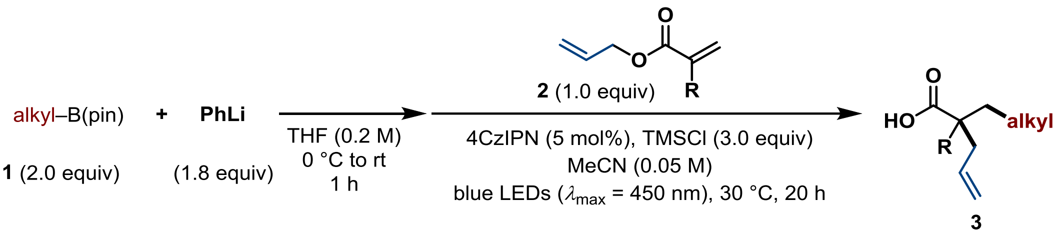

To an oven-dried 10 mL Schlenk tube equipped with a Teflon-coated magnetic stir bar were added the appropriate boronic ester (0.40 mmol, 2.0 equiv), and dry THF (2.0 mL, 0.2 M) under an argon atmosphere. The solution was cooled to 0 °C, and a PhLi solution (1.9 M in dibutyl ether, 0.19 mL, 0.36 mmol, 1.8 equiv) was added dropwise while stirring. The mixture was stirred for 45 min at 0 °C and was then allowed to warm up to room temperature for further 15 min. After that, the solvent was removed *in vacuo* (*via* Schlenk line), and the resulting colorless solid was dissolved in dry MeCN (4.0 mL, 0.05 M). To this solution were added the appropriate allyl acrylate (0.20 mmol, 1.0 equiv), 4CzIPN (7.9 mg, 0.010 mmol, 5 mol%), and TMSCl (80 µL, 0.60 mmol, 3.0 equiv). The reaction mixture was degassed by three freeze-pump-thaw cycles and subsequently stirred and irradiated with blue LEDs (30 W, λ_{max} = 450 nm) for 20 h. The solvent was removed through a stream of argon, 1,3,5-trimethoxybenzene (33.6 mg, 0.20 mmol, 1.0 equiv) was added as internal standard, and the ^{1}H NMR yield was determined (the resulting suspension after addition of $CDCl_3$ was filtered over a pad of Celite® directly into the NMR tube). The NMR tube, used pipettes, and the Schlenk tube were carefully rinsed with CH_2Cl_2 into a round bottom flask.

work-up 1: MeOH (1.0 mL) and HCOOH (0.1 mL) were added, and the mixture was stirred for at least 1 h at room temperature.

work-up 2: The mixture was transferred into a separation funnel containing aq. HCl (1 M). The aq. layer was separated, extracted with EtOAc (5×10 mL), and the org. layers were combined.

In both cases the solvent was removed *in vacuo*, and the residue was purified by silica gel column chromatography and/or PTLC to afford the product.

2-(Cyclohexylmethyl)-2-methylpent-4-enoic acid (**3a**)**:** The title compound was prepared from 2-cyclohexyl-4,4,5,5-tetramethyl-1,3,2-dioxaborolane (**1a**, 84.0 mg, 0.40 mmol, 2.0 equiv) and allyl methacrylate (**2a**, 27.1 µL, 0.20 mmol, 1.0 equiv) according to *General Procedure 4*. After *work-up 2*, purification by silica gel column chromatography (CH_2Cl_2 to 0.5% HOAc/1% EtOAc in CH_2Cl_2) gave the product (32.4 mg, 77%) as colorless oil.

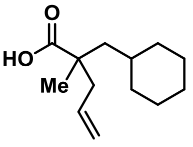

3a

^{1}H NMR (400 MHz, $CDCl_3$) δ 5.81 – 5.66 (m, 1H), 5.08 (s, 1H), 5.06 – 5.02 (m, 1H), 2.41 (ddt, J = 13.7, 7.1, 1.2 Hz, 1H), 2.15 (ddt, J = 13.7, 7.7, 1.1 Hz, 1H), 1.67 – 1.60 (m, 5H), 1.41 – 1.33 (m, 2H), 1.30 – 1.04 (m, 7H), 1.00 – 0.89 (m, 2H); **^{13}C NMR** (126 MHz, $CDCl_3$) δ 184.4, 133.8, 118.4, 46.6, 45.4, 44.5, 35.0, 34.5, 33.9, 26.6, 26.5, 26.4, 21.1; **HRMS** (ESI^{-}) calc'd for $[M-H]^{-}$ 237.1860, found 237.1857; **R_f** (0.5% HOAc / 3% EtOAc in CH_2Cl_2) = 0.47.

2-(Cyclopentylmethyl)-2-methylpent-4-enoic acid (**3b**)**:** The title compound was prepared from 2-cyclopentyl-4,4,5,5-tetramethyl-1,3,2-dioxaborolane (**1b**, 78.4 mg, 0.40 mmol, 2.0 equiv) and allyl methacrylate (**2a**, 27.1 µL, 0.20 mmol, 1.0 equiv) according to *General Procedure 4*. After *work-up 1*, purification by PTLC (1st run: CH_2Cl_2, 2nd run 1% HCOOH/5% EtOAc in pentane; 2nd PTLC: 1% HCOOH/10% EtOAc in pentane) gave the product (17.9 mg, 46%) as a colorless oil.

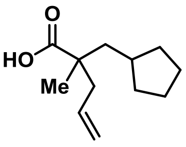

3b

^{1}H NMR (400 MHz, $CDCl_3$) δ 5.83 – 5.68 (m, 1H), 5.10 – 5.07 (m, 1H), 5.07 – 5.03 (m, 1H), 2.45 (ddt, J = 13.6, 7.0, 1.3 Hz, 1H), 2.17 (ddt, J = 13.6, 7.8, 1.1 Hz, 1H), 1.85 – 1.72 (m, 4H), 1.64 – 1.55 (m, 3H), 1.52 – 1.42 (m, 2H), 1.16 (s, 3H), 1.13 – 1.01 (m, 2H); **^{13}C NMR** (101 MHz, $CDCl_3$) δ 184.1, 134.0, 118.4, 46.0, 45.6, 44.2, 37.0, 34.3, 33.5, 25.2, 25.0, 21.3; **HRMS** (ESI^{-}) calc'd for $[M-H]^{-}$ 195.1391, found 195.1388; **R_f** (1% HCOOH/10% EtOAc in pentane) = 0.56.

2-(Cycloheptylmethyl)-2-methylpent-4-enoic acid (**3c**)**:** The title compound was prepared from 2-cycloheptyl-4,4,5,5-tetramethyl-1,3,2-dioxaborolane (**1c**, 89.6 mg, 0.40 mmol, 2.0 equiv) and allyl methacrylate (**2a**, 27.1 µL, 0.20 mmol, 1.0 equiv) according to *General Procedure 4*. The reaction was performed in a THF/MeCN (1/1, 0.05 M) solvent mixture. After *work-up 1*, purification by silica gel column chromatography (CH_2Cl_2 to 1% HCOOH/1% EtOAc in

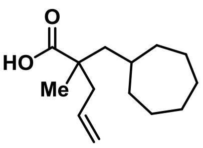

3c

CH_2Cl_2) and subsequent PTLC (1% HCOOH/10% EtOAc in pentane) gave the product (34.4 mg, 77%) as a colorless oil.

^{1}H NMR (400 MHz, $CDCl_3$) *δ* 5.81 – 5.67 (m, 1H), 5.11 – 5.07 (m, 1H), 5.07 – 5.03 (m, 1H), 2.42 (ddt, *J* = 13.6, 7.0, 1.2 Hz, 1H), 2.14 (ddt, *J* = 13.6, 7.7, 1.1 Hz, 1H), 1.74 – 1.51 (m, 8H), 1.49 – 1.32 (m, 5H), 1.28 – 1.17 (m, 2H), 1.14 (s, 3H); **^{13}C NMR** (101 MHz, $CDCl_3$) *δ* 184.3, 133.9, 118.4, 47.3, 45.8, 44.5, 36.5, 36.1, 35.5, 28.6, 28.5, 26.3, 26.3, 21.0; **HRMS** (ESI^-) calc'd for $[M-H]^-$ 223.1693, found 223.1699; **R_f** (1% HCOOH/1% EtOAc in CH_2Cl_2) = 0.40.

2-((1-(*tert*-Butoxycarbonyl)piperidin-4-yl)methyl)-2-methylpent-4-enoic acid (3d): The title compound was prepared from *tert*-butyl 4-(4,4,5,5-tetramethyl-1,3,2-dioxaborolan-2-yl)piperidine-1-carboxylate (**1d**, 124.4 mg, 0.40 mmol, 2.0 equiv) and allyl methacrylate (**2a**, 27.1 µL, 0.20 mmol, 1.0 equiv) according to *General Procedure 4*. After *work-up 1* (without the addition of the acid), purification by PTLC (1% HCOOH/30% EtOAc in pentane) gave the product (37.3 mg, 60%) as a slightly brown solid.

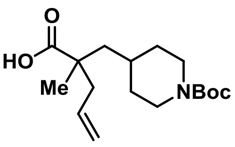

3d

^{1}H NMR (400 MHz, $CDCl_3$) *δ* 5.72 (ddt, *J* = 16.0, 10.8, 7.4 Hz, 1H), 5.14 – 5.01 (m, 2H), 4.13 – 3.90 (m, 2H), 2.77 – 2.54 (m, 2H), 2.41 (dd, *J* = 13.7, 7.1 Hz, 1H), 2.16 (dd, *J* = 13.7, 7.7 Hz, 1H), 1.68 (dd, *J* = 13.9, 6.6 Hz, 1H), 1.63 – 1.46 (m, 4H), 1.45 (s, 9H), 1.42 – 1.37 (m, 1H), 1.18 – 1.12 (m, 4H); **^{13}C NMR** (101 MHz, $CDCl_3$) *δ* 182.9, 155.0, 133.5, 118.6, 79.6, 45.4, 45.3, 44.5, 44.0 (b), 33.7, 33.6, 32.9, 32.7, 28.6, 21.2; **HRMS** (ESI^+) calc'd for $[M+Na]^+$ 334.1989, found 334.1987;**R_f** (1% HCOOH/30% EtOAc in pentane) = 0.45.

2-Methyl-2-((tetrahydro-2*H*-pyran-4-yl)methyl)pent-4-enoic acid (3e): The title compound was prepared from 4,4,5,5-tetramethyl-2-(tetrahydro-2*H*-pyran-4-yl)-1,3,2-dioxaborolane (**1e**, 84.9 mg, 0.40 mmol, 2.0 equiv) and allyl methacrylate (**2a**, 27.1 µL, 0.20 mmol, 1.0 equiv) according to *General Procedure 4*. After *work-up 2*, purification by silica gel column chromatography (CH_2Cl_2 to 0.5% HCOOH/10% EtOAc in CH_2Cl_2) and subsequent PTLC (2% HCOOH/40% EtOAc in pentane) gave the product (27.2 mg, 64%) as a colorless oil.

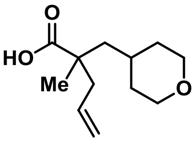

3e

^{1}H NMR (400 MHz, $CDCl_3$) *δ* 5.73 (ddt, *J* = 15.9, 11.2, 7.4 Hz, 1H), 5.09 (s, 1H), 5.08 – 5.04 (m, 1H), 3.95 – 3.85 (m, 2H), 3.35 (tdd, *J* = 12.0, 10.2, 2.1 Hz, 2H), 2.41 (dd, *J* = 13.7, 7.1 Hz,

1H), 2.17 (dd, J = 13.7, 7.7 Hz, 1H), 1.70 (dd, J = 13.6, 6.8 Hz, 1H), 1.66 – 1.58 (m, 1H), 1.58 – 1.49 (m, 2H), 1.42 (dd, J = 13.7, 4.9 Hz, 1H), 1.38 – 1.28 (m, 2H), 1.16 (s, 3H); **^{13}C NMR** (101 MHz, $CDCl_3$) δ 183.4, 133.5, 118.7, 68.1, 68.0, 45.9, 45.2, 44.5, 34.5, 33.6, 31.9, 21.2; **HRMS** (ESI$^-$) calc'd for $[M-H]^-$ 211.1339, found 211.1337; **R_f** (2% HCOOH/40% EtOAc in pentane) = 0.59.

2-((4,4-Difluorocyclohexyl)methyl)-2-methylpent-4-enoic acid (3f): The title compound was prepared from 2-(4,4-difluorocyclohexyl)-4,4,5,5-tetramethyl-1,3,2-dioxaborolane (**1f**, 98.4 mg, 0.40 mmol, 2.0 equiv) and allyl methacrylate (**2a**, 27.1 µL, 0.20 mmol, 1.0 equiv) according to *General Procedure 4*. After *work-up 1*, purification by silica gel column chromatography (1st column: CH_2Cl_2 to 0.5% HOAc/2% EtOAc in CH_2Cl_2, 2nd column: 0.5% HCOOH/5% EtOAc in pentane) and gave the product (37.6 mg, 76%) as a colorless oil.

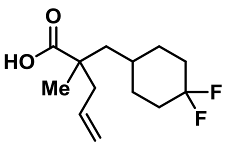

3f

^{1}H NMR (400 MHz, $CDCl_3$) δ 5.73 (ddt, J = 16.4, 10.7, 7.4 Hz, 1H), 5.12 – 5.04 (m, 2H), 2.42 (dd, J = 13.7, 7.1 Hz, 1H), 2.18 (dd, J = 13.7, 7.7 Hz, 1H), 2.07 – 1.98 (m, 2H), 1.75 – 1.67 (m, 4H), 1.66 – 1.58 (m, 1H), 1.45 – 1.41 (m, 1H), 1.40 – 1.24 (m, 3H), 1.17 (s, 3H); **^{13}C NMR** (101 MHz, $CDCl_3$) δ 183.7, 133.3, 123.4 (dd, J = 241.9, 239.4 Hz), 118.9, 45.5, 44.5, 44.5, 33.6 (dd, J = 2.9, 2.9 Hz), 33.6 (dd, J = 48.0, 3.5 Hz), 32.6, 30.1 (dd, J = 110.3, 9.4 Hz), 21.1 – one signal is missing due to overlap; **^{19}F NMR** (376 MHz, $CDCl_3$) δ −92.2 (d, J = 235.0 Hz), −101.9 (d, J = 235.1 Hz); **R_f** (1% HCOOH/10% EtOAc in pentane) = 0.38.

2-((2,3-Dihydro-1*H*-inden-2-yl)methyl)-2-methylpent-4-enoic acid (3g): The title compound was prepared from 2-(2,3-dihydro-1*H*-inden-2-yl)-4,4,5,5-tetramethyl-1,3,2-dioxaborolane (**1g**, 97.6 mg, 0.40 mmol, 2.0 equiv) and allyl methacrylate (**2a**, 27.1 µL, 0.20 mmol, 1.0 equiv) according to *General Procedure 4*. After *work-up 1,* purification by PTLC (1st PTLC: 1% HCOOH/3% EtOAc in CH_2Cl_2, 2nd PTLC: 1% HCOOH/5% EtOAc in pentane) gave the product (17.5 mg, 36%) as a colorless solid.

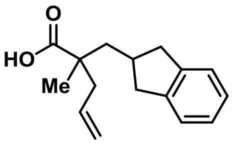

3g

^{1}H NMR (400 MHz, $CDCl_3$) δ 7.18 – 7.06 (m, 4H), 5.87 – 5.73 (m, 1H), 5.16 – 5.08 (m, 2H), 3.10 – 2.97 (m, 2H), 2.69 – 2.46 (m, 4H), 2.27 (ddt, J = 13.6, 7.7, 1.1 Hz, 1H), 2.06 (dd, J = 14.0, 6.4 Hz, 1H), 1.82 (dd, J = 14.0, 5.9 Hz, 1H), 1.25 (s, 3H); **^{13}C NMR** (101 MHz, $CDCl_3$) δ 183.8, 143.4, 143.3, 133.7, 126.3, 126.3, 124.3, 124.2, 118.7, 46.1, 44.8, 44.1, 40.8, 40.1,

37.8, 21.4; **HRMS** (ESI$^-$) calc'd for [M−H]$^-$ 243.1391, found 243.1387; **R$_f$** (1% HCOOH/3% EtOAc in CH_2Cl_2) = 0.66.

2-Allyl-4-ethyl-2-methylhexanoic acid (**3h**)**:** The title compound was prepared from 4,4,5,5-tetramethyl-2-(pentan-3-yl)-1,3,2-dioxaborolane (**1h**, 79.2 mg, 0.40 mmol, 2.0 equiv) and allyl methacrylate (**2a**, 27.1 µL, 0.20 mmol, 1.0 equiv) according to *General Procedure 4*. After *work-up 1*, purification by PTLC (1st PTLC: 1% HCOOH/2% EtOAc in CH_2Cl_2, 2nd PTLC: 1% HCOOH/10% Et_2O in pentane) gave the product (27.4 mg, 69%) as a colorless oil.

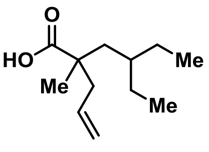

3h

^{1}H NMR (300 MHz, $CDCl_3$) δ 5.84 – 5.66 (m, 1H), 5.12 – 5.07 (m, 1H), 5.07 – 5.02 (m, 1H), 2.46 (ddt, J = 13.6, 6.9, 1.3 Hz, 1H), 2.12 (ddt, J = 13.6, 7.8, 1.1 Hz, 1H), 1.65 (dd, J = 13.9, 5.0 Hz, 1H), 1.41 (dd, J = 14.0, 4.3 Hz, 1H), 1.34 – 1.22 (m, 5H), 1.14 (s, 3H), 0.82 (td, J = 7.2, 5.3 Hz, 6H); **^{13}C NMR** (76 MHz, $CDCl_3$) δ 184.4, 134.0, 118.4, 45.7, 44.4, 43.2, 36.7, 26.7, 26.1, 21.0, 10.8, 10.6; **HRMS** (ESI$^-$) calc'd for [M−H]$^-$ 197.1536, found 197.1544; **R$_f$** (1% HCOOH/2% EtOAc in CH_2Cl_2) = 0.45.

2-(((1*s*,3*s*)-Adamantan-1-yl)methyl)-2-methylpent-4-enoic acid (**3i**)**:** The title compound was prepared from 2-((3*r*,5*r*,7*r*)-adamantan-1-yl)-4,4,5,5-tetramethyl-1,3,2-dioxaborolane (**1i**, 104.8 mg, 0.40 mmol, 2.0 equiv) and allyl methacrylate (**2a**, 27.1 µL, 0.20 mmol, 1.0 equiv) according to *General Procedure 4*. After *work-up 2*, purification by silica gel column chromatography (1% HCOOH/5% Et_2O in pentane) and subsequent PTLC (0.5% HCOOH/1% EtOAc in CH_2Cl_2) gave the product (28.3 mg, 54%) as a colorless solid.

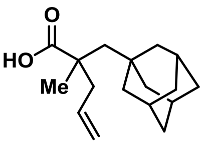

3i

^{1}H NMR (400 MHz, $CDCl_3$) δ 5.72 (ddt, J = 16.9, 10.5, 7.3 Hz, 1H), 5.13 – 5.00 (m, 2H), 2.41 (dd, J = 13.5, 7.0 Hz, 1H), 2.13 (dd, J = 13.5, 7.8 Hz, 1H), 1.96 – 1.84 (m, 3H), 1.73 (d, J = 14.6 Hz, 1H), 1.70 – 1.57 (m, 9H), 1.57 – 1.49 (m, 3H), 1.28 (d, J = 14.6 Hz, 1H), 1.23 (s, 3H); **^{13}C NMR** (76 MHz, $CDCl_3$) δ 184.8, 133.5, 118.7, 53.7, 47.0, 45.4, 43.3, 37.0, 34.1, 28.9, 22.3; **HRMS** (ESI$^-$) calc'd for [M−H]$^-$ 261.1849, found 261.1853; **R$_f$** (1% HCOOH/10% Et_2O in pentane) =0.26.

2-Allyl-7-(2,5-dimethylphenoxy)-2,4,4-trimethylheptanoic acid (3j): The title compound was prepared from 2-(5-(2,5-dimethylphenoxy)-2-methylpentan-2-yl)-4,4,5,5-tetramethyl-1,3,2-dioxaborolane (**1j**, 132.9 mg, 0.40 mmol, 2.0 equiv) and allyl methacrylate (**2a**, 27.1 µL, 0.20 mmol, 1.0 equiv) according to *General Procedure 4*. After *work-up 1*, purification by silica gel column chromatography (CH_2Cl_2 to 1% HCOOH/2% EtOAc in CH_2Cl_2) and subsequent PTLC (1% HCOOH/10% EtOAc in pentane) gave the product (46.3 mg, 70%) as a colorless oil.

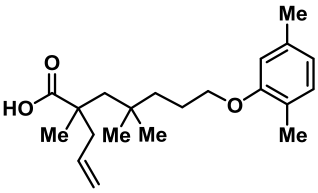

3j

^{1}H NMR (400 MHz, $CDCl_3$) δ 7.02 (d, J = 7.5 Hz, 1H), 6.67 (d, J = 7.5 Hz, 1H), 6.64 (s, 1H), 5.85 – 5.67 (m, 1H), 5.14 – 5.04 (m, 2H), 3.91 (td, J = 6.5, 2.0 Hz, 2H), 2.45 (dd, J = 13.5, 6.9 Hz, 1H), 2.33 (s, 3H), 2.20 (s, 3H), 2.16 (dd, J = 13.6, 7.9 Hz, 1H), 1.95 (d, J = 14.6 Hz, 1H), 1.83 – 1.74 (m, 2H), 1.50 (d, J = 14.6 Hz, 1H), 1.47 – 1.36 (m, 2H), 1.26 (s, 3H), 1.01 (s, 3H), 0.98 (s, 3H); **^{13}C NMR** (101 MHz, $CDCl_3$) δ 184.8, 157.2, 136.5, 133.4, 130.4, 123.7, 120.7, 118.9, 112.1, 68.6, 50.1, 47.1, 45.9, 41.0, 34.2, 28.1, 27.5, 24.4, 21.6, 21.5, 16.0; **HRMS** (ESI$^+$) calc'd for $[M+Na]^+$ 355.2244, found 355.2242; **R_f** (1% HCOOH/10% EtOAc in pentane) = 0.78.

2-(Cyclohexylmethyl)-2,3,3-trimethylpent-4-enoic acid (3k): The title compound was prepared from 2-cyclohexyl-4,4,5,5-tetramethyl-1,3,2-dioxaborolane (**1a**, 84.0 mg, 0.40 mmol, 2.0 equiv) and 3-methylbut-2-en-1-yl methacrylate (**2b**, 30.9 mg, 0.20 mmol, 1.0 equiv) according to *General Procedure 4*. The reaction was performed in a THF/MeCN (1/1, 0.05 M) solvent mixture. After *work-up 1*, purification by PTLC (1st PTLC: 0.5% HCOOH/1% EtOAc in CH_2Cl_2, 2nd PTLC: 1% HCOOH/5% EtOAc in pentane) gave the product (29.3 mg, 61%) as a colorless oil.

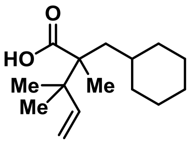

3k

^{1}H NMR (400 MHz, $CDCl_3$) δ 5.95 (dd, J = 17.4, 10.8 Hz, 1H), 5.07 – 4.92 (m, 2H), 1.86 – 1.75 (m, 1H), 1.69 – 1.55 (m, 5H), 1.29 – 1.12 (m, 5H), 1.09 (s, 3H), 1.05 (s, 6H), 0.98 – 0.83 (m, 2H); **^{13}C NMR** (101 MHz, $CDCl_3$) δ 183.8, 144.6, 112.7, 50.5, 42.0, 40.9, 35.7, 35.1, 32.9, 26.7, 26.6, 26.5, 23.0, 22.7, 17.3; **HRMS** (ESI$^-$) calc'd for $[M-H]^-$ 237.1849, found 237.1857; **R_f** (1% HCOOH/5% EtOAc in pentane) = 0.50.

3-Cyclohexyl-2-methyl-2-(4-oxo-1-vinylcyclohexyl)propanoic acid (3l): The title compound was prepared from 2-cyclohexyl-4,4,5,5-tetramethyl-1,3,2-dioxaborolane (**1a**, 84.0 mg, 0.40 mmol, 2.0 equiv) and 2-(4-oxocyclohexylidene)ethyl methacrylate (**2c**, 41.7 mg, 0.20 mmol, 1.0 equiv) according to *General Procedure 4*. After *work-up 1*, purification by silica gel column chromatography (CH_2Cl_2 to 1% HCOOH/20% EtOAc in pentane) and subsequent PTLC (1% HCOOH/25% EtOAc in CH_2Cl_2) gave the product (25.0 mg, 43%) as a colorless solid.

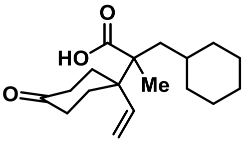

3l

^{1}H NMR (400 MHz, $CDCl_3$) δ 5.71 (dd, J = 17.8, 11.0 Hz, 1H), 5.49 (d, J = 11.0 Hz, 1H), 5.19 (d, J = 17.7 Hz, 1H), 2.43 – 2.31 (m, 2H), 2.31 – 2.18 (m, 3H), 2.15 – 2.01 (m, 2H), 1.86 – 1.73 (m, 2H), 1.71 – 1.55 (m, 5H), 1.35 – 1.14 (m, 4H), 1.13 (s, 3H), 1.11 – 1.06 (m, 1H), 1.03 – 0.93 (m, 1H), 0.92 – 0.82 (m, 1H); **^{13}C NMR** (101 MHz, $CDCl_3$) δ 212.3, 183.0, 138.7, 118.7, 51.5, 45.0, 40.4, 38.2, 38.1, 35.7, 34.9, 32.8, 30.3, 28.9, 26.6, 26.5, 26.4, 17.1; **HRMS** (ESI$^-$) calc'd for $[M{-}H]^-$ 291.1966, found 291.1961; **R$_f$** (1% HCOOH/20% EtOAc in pentane) = 0.30.

(*E*)-2-(Cyclohexylmethyl)-2-methylhex-4-enoic acid (3m): The title compound was prepared from 2-cyclohexyl-4,4,5,5-tetramethyl-1,3,2-dioxaborolane (**1a**, 84.0 mg, 0.40 mmol, 2.0 equiv) and but-3-en-2-yl methacrylate (**2d**, 28.0 mg, 0.20 mmol, 1.0 equiv) according to *General Procedure 4*. After *work-up 1*, purification by silica gel column chromatography (CH_2Cl_2 to 1% HCOOH/1.5% EtOAc in CH_2Cl_2) and subsequent PTLC (1% HCOOH/1% EtOAc in pentane) gave the product (22.3 mg, 50%) as a colorless oil.

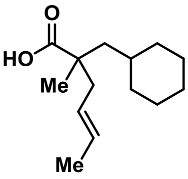

3m

^{1}H NMR (400 MHz, $CDCl_3$) δ 5.53 – 5.42 (m, 1H), 5.40 – 5.28 (m, 1H), 2.33 (dd, J = 13.7, 6.9 Hz, 1H), 2.07 (dd, J = 13.6, 7.4 Hz, 1H), 1.69 – 1.58 (m, 9H), 1.36 – 1.31 (m, 2H), 1.29 – 1.13 (m, 3H), 1.11 (s, 3H), 0.99 – 0.88 (m, 2H); **^{13}C NMR** (101 MHz, $CDCl_3$) δ 184.5, 129.0, 126.1, 46.6, 45.6, 43.5, 35.0, 34.5, 33.8, 26.6, 26.5, 26.4, 20.9, 18.2; **HRMS** (ESI$^-$) calc'd for $[M{-}H]^-$ 223.1704, found 223.1700; **R$_f$** (1% HCOOH/1.5% EtOAc in CH_2Cl_2) = 0.50.

2-(Cyclohexylmethyl)-2,5-dimethylhex-4-enoic acid (3n): The title compound was prepared from 2-cyclohexyl-4,4,5,5-tetramethyl-1,3,2-dioxaborolane (**1a**, 84.0 mg, 0.40 mmol, 2.0 equiv) and 2-methylbut-3-en-2-yl methacrylate (**2e**, 30.8 mg, 0.20 mmol, 1.0 equiv) according to *General Procedure 4*. The reaction was performed in a THF/MeCN (1/1, 0.05 M) solvent mixture. After *work-up 2*,

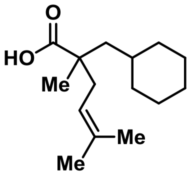

3n

purification by silica gel column chromatography (0.5% HOAc/3% EtOAc in CH_2Cl_2) gave the product (27.4 mg, 57%) as a colorless oil.

^{1}H NMR (300 MHz, $CDCl_3$) δ 5.09 (t, J = 7.6 Hz, 1H), 2.34 (dd, J = 14.3, 7.3 Hz, 1H), 2.11 (dd, J = 14.2, 7.9 Hz, 1H), 1.70 (s, 3H), 1.69 – 1.53 (m, 9H), 1.41 – 1.30 (m, 2H), 1.30 – 1.13 (m, 3H), 1.11 (s, 3H), 1.00 – 0.84 (m, 2H); **^{13}C NMR** (75 MHz, $CDCl_3$) δ 185.1, 134.7, 119.4, 46.6, 45.9, 38.8, 35.1, 34.6, 33.8, 26.6, 26.5, 26.4, 26.2, 20.8, 18.1; **HRMS** (ESI^-) calc'd for $[M-H]^-$ 237.1860, found 237.1857; **R_f** (0.5% HOAc/3% EtOAc in CH_2Cl_2) = 0.50.

2-(Cyclohexylmethyl)-2-methyl-4-(tetrahydro-4*H*-pyran-4-ylidene)butanoic acid (3o):

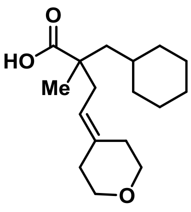

3o

The title compound was prepared from 2-cyclohexyl-4,4,5,5-tetramethyl-1,3,2-dioxaborolane (**1a**, 84.0 mg, 0.40 mmol, 2.0 equiv) and 4-vinyltetrahydro-2*H*-pyran-4-yl methacrylate (**2f**, 39.3 mg, 0.20 mmol, 1.0 equiv) according to *General Procedure 4*. After *work-up 1*, purification by silica gel column chromatography (CH_2Cl_2 to 1% HCOOH/10% EtOAc in CH_2Cl_2) gave the product (46.8 mg, 83%) as a colorless solid.

^{1}H NMR (400 MHz, $CDCl_3$) δ 5.16 (t, J = 7.7 Hz, 1H), 3.71 – 3.57 (m, 4H), 2.37 (dd, J = 14.1, 7.6 Hz, 1H), 2.26 (q, J = 4.8 Hz, 2H), 2.21 (t, J = 5.2 Hz, 2H), 2.11 (dd, J = 14.1, 7.9 Hz, 1H), 1.69 – 1.58 (m, 6H), 1.40 – 1.32 (m, 2H), 1.29 – 1.14 (m, 3H), 1.13 (s, 3H), 1.00 – 0.85 (m, 2H); **^{13}C NMR** (101 MHz, $CDCl_3$) δ 184.3, 137.4, 118.2, 69.8, 68.8, 46.7, 45.8, 37.5, 37.4, 35.1, 34.5, 33.8, 30.1, 26.5, 26.5, 26.4, 20.9; **HRMS** (ESI^+) calc'd for $[M+Na]^+$ 303.1931, found 303.1929; **R_f** (1% HCOOH/10 EtOAc in CH_2Cl_2) = 0.29.

4-Chloro-2-(cyclohexylmethyl)-2-methylpent-4-enoic acid (3p): The title compound was

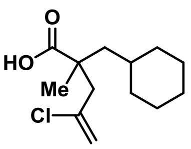

3p

prepared from 2-cyclohexyl-4,4,5,5-tetramethyl-1,3,2-dioxaborolane (**1a**, 84.0 mg, 0.40 mmol, 2.0 equiv) and 2-chloroallyl methacrylate (**2g**, 32.1 mg, 0.20 mmol, 1.0 equiv) according to *General Procedure 4*. After *work-up 1*, purification by silica gel column chromatography (CH_2Cl_2 to 1% HCOOH/1% EtOAc in CH_2Cl_2) and subsequent PTLC (1% HCOOH/10% EtOAc in pentane) gave the product (25.1 mg, 51%) as a colorless solid.

^{1}H NMR (400 MHz, $CDCl_3$) δ 5.27 (d, J = 1.2 Hz, 1H), 5.15 (s, 1H), 2.87 (d, J = 14.4 Hz, 1H), 2.40 (d, J = 14.4 Hz, 1H), 1.76 – 1.60 (m, 6H), 1.41 – 1.32 (m, 2H), 1.25 (s, 3H), 1.23 – 1.07

(m, 3H), 1.01 – 0.89 (m, 2H); **^{13}C NMR** (101 MHz, $CDCl_3$) δ 183.6, 138.7, 116.3, 49.2, 47.5, 45.6, 35.3, 34.3, 34.0, 26.5, 26.5, 26.3, 20.3; **HRMS** (ESI$^-$) calc'd for $[M-H]^-$ 243.1146, found 243.1155; **R$_f$** (1% HCOOH/3% EtOAc in CH_2Cl_2) = 0.43.

2-(Cyclohexylmethyl)-2-methyl-3-vinylhexanoic acid (**3q**)**:** The title compound was prepared from 2-cyclohexyl-4,4,5,5-tetramethyl-1,3,2-dioxaborolane (**1a**, 84.0 mg, 0.40 mmol, 2.0 equiv) and (*E*)-hex-2-en-1-yl methacrylate (**2h**, 33.6 mg, 0.20 mmol, 1.0 equiv) according to *General Procedure 4*. The reaction was performed in a THF/MeCN (1/1, 0.05 M) solvent mixture. After *work-up 1*, purification by silica gel column chromatography (1% HCOOH/2% EtOAc to 5% EtOAc in pentane) and subsequent PTLC (3% MeOH in CH_2Cl_2) gave the product (31.5 mg, 62%) as a colorless oil.

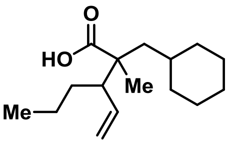

3q

^{1}H NMR (400 MHz, $CDCl_3$, both diastereomers) δ 5.66 – 5.37 (m, 1H), 5.16 – 4.92 (m, 2H), 2.25 – 1.98 (m, 1H), 1.70 – 1.54 (m, 6H), 1.47 – 1.11 (m, 9H), 1.09 – 1.06 (m, 3H), 0.98 – 0.82 (m, 5H); **^{13}C NMR** (101 MHz, $CDCl_3$, both diastereomers) δ 184.6, 184.0, 138.5, 138.0, 118.5, 117.5, 53.2, 52.4, 49.1, 48.8, 46.0, 44.5, 35.7, 35.6, 34.8, 34.3, 33.7, 33.5, 32.1, 30.6, 26.6, 26.6, 26.6, 26.4, 26.4, 21.1, 21.0, 17.3, 16.0, 14.1, 14.0 – one carbon is missing due to overlap; **HRMS** (ESI$^-$) calc'd for $[M-H]^-$ 251.2017, found 251.2014; **R$_f$** (1% HCOOH/1% EtOAc in CH_2Cl_2) = 0.66.

2-(Cyclohexylmethyl)-2-fluoropent-4-enoic acid (**3r**)**:** The title compound was prepared from 2-cyclohexyl-4,4,5,5-tetramethyl-1,3,2-dioxaborolane (**1a**, 84.0 mg, 0.40 mmol, 2.0 equiv) and allyl 2-fluoroacrylate (**2i**, 26.0 mg, 0.20 mmol, 1.0 equiv) according to *General Procedure 4*. After *work-up 1*, purification by silica gel column chromatography (1st column: 1% HCOOH/5% EtOAc in pentane, 2nd column: CH_2Cl_2 to 1% HCOOH/3%EtOAc in CH_2Cl_2) gave the product (24.1 mg, 56%) as a colorless oil.

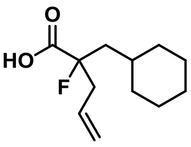

3r

^{1}H NMR (400 MHz, $CDCl_3$) δ 8.63 (bs, 1H), 5.80 (ddt, J = 18.6, 9.5, 7.2 Hz, 1H), 5.19 (s, 1H), 5.18 – 5.13 (m, 1H), 2.70 – 2.55 (m, 2H), 1.95 – 1.75 (m, 3H), 1.73 – 1.60 (m, 4H), 1.57 – 1.47 (m, 1H), 1.27 – 1.20 (m, 2H), 1.18 – 1.09 (m, 1H), 1.03 – 0.90 (m, 2H); **^{13}C NMR** (101 MHz, $CDCl_3$) δ 177.0 (d, J = 26.9 Hz), 130.5 (d, J = 3.9 Hz), 120.2, 97.3 (d, J = 190.2 Hz), 44.1 (d, J = 20.8 Hz), 42.6 (d, J = 22.1 Hz), 34.2 (d, J = 2.0 Hz), 33.9, 33.7, 26.3, 26.2 – one signal is

missing due to overlap; **^{19}F NMR** (377 MHz, $CDCl_3$) δ −164.2; **HRMS** (ESI$^-$) calc'd for $[M-H]^-$ 213.1285, found 213.1294; **R$_f$** (1% HCOOH/10% EtOAc in pentane) = 0.32.

2-(Cyclohexylmethyl)-2-phenylpent-4-enoic acid (**3s**)**:** The title compound was prepared from 2-cyclohexyl-4,4,5,5-tetramethyl-1,3,2-dioxaborolane (**1a**, 84.0 mg, 0.40 mmol, 2.0 equiv) and allyl 2-phenylacrylate (**2j**, 37.6 mg, 0.20 mmol, 1.0 equiv) according to *General Procedure 4*. The reaction was performed in a THF/MeCN (1/1, 0.05 M) solvent mixture. After *work-up 2*, purification by silica gel column chromatography (CH_2Cl_2 to 0.5% HOAc/3% EtOAc in CH_2Cl_2) gave the product (35.0 mg, 64%) as a colorless solid.

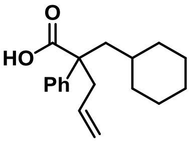

3s

^{1}H NMR (400 MHz, $CDCl_3$) δ 7.28 – 7.22 (m, 4H), 7.20 – 7.15 (m, 1H), 5.44 (ddt, *J* = 17.2, 10.2, 7.1 Hz, 1H), 5.01 – 4.89 (m, 2H), 2.83 – 2.71 (m, 2H), 1.96 – 1.81 (m, 2H), 1.58 – 1.45 (m, 4H), 1.34 – 1.28 (m, 1H), 1.26 – 1.17 (m, 1H), 1.11 – 0.98 (m, 3H), 0.96 – 0.87 (m, 1H), 0.85 – 0.77 (m, 1H); **^{13}C NMR** (101 MHz, $CDCl_3$) δ 182.1, 142.1, 133.7, 128.4, 127.1, 126.8, 118.6, 53.3, 41.7, 39.5, 35.1, 34.3, 34.0, 26.6, 26.6, 26.4; **HRMS** (ESI$^-$) calc'd for $[M-(HCO_2)]^-$ 227.1805, found 227.1801; **R$_f$** (0.5% HOAc/3% EtOAc in CH_2Cl_2) = 0.23; **X-ray** (single-crystal) A colorless prism-like specimen of X-ray diffraction quality was obtained by slow evaporation of a solution of **3s** in $CDCl_3$ (CCDC 2033245).

2-(Cyclohexylmethyl)-2-(4-methoxyphenyl)pent-4-enoic acid (**3t**)**:** The title compound was prepared from 2-cyclohexyl-4,4,5,5-tetramethyl-1,3,2-dioxaborolane (**1a**, 84.0 mg, 0.40 mmol, 2.0 equiv) and allyl 2-(4-methoxyphenyl)acrylate (**2k**, 43.7 mg, 0.20 mmol, 1.0 equiv) according to *General Procedure 4*. After *work-up 1*, purification by silica gel column chromatography (CH_2Cl_2 to 1% HCOOH/3% EtOAc in pentane) and subsequent PTLC (1st PTLC: 1% HCOOH/5% EtOAc in CH_2Cl_2, 2nd PTLC: 7% MeOH in CH_2Cl_2) gave the product (32.8 mg, 54%) as a colorless solid.

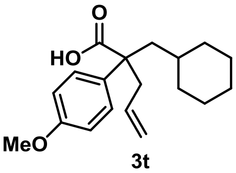

3t

^{1}H NMR (400 MHz, $CDCl_3$) δ 7.24 (d, *J* = 8.9 Hz, 2H), 6.85 (d, *J* = 8.9 Hz, 2H), 5.51 (ddt, *J* = 17.2, 10.2, 7.1 Hz, 1H), 5.10 – 4.95 (m, 2H), 3.80 (s, 3H), 2.82 (d, *J* = 7.1 Hz, 2H), 1.98 (dd, *J* = 14.1, 6.1 Hz, 1H), 1.88 (dd, *J* = 14.1, 5.1 Hz, 1H), 1.66 – 1.52 (m, 4H), 1.43 – 1.25 (m, 2H), 1.19 – 1.05 (m, 3H), 1.03 – 0.93 (m, 1H), 0.93 – 0.82 (m, 1H); **^{13}C NMR** (101 MHz, $CDCl_3$) δ 182.3, 158.4, 134.2, 133.9, 127.8, 118.5, 113.8, 55.3, 52.5, 41.8, 39.4, 35.1, 34.2, 34.0, 26.6,

26.6, 26.4; **HRMS** (ESI$^-$) calc'd for [M−H]$^-$ 301.1809, found 301.1806; **R$_f$** (1% HCOOH/5% EtOAc in CH_2Cl_2) = 0.58.

2-(Cyclohexylmethyl)-2-(4-fluorophenyl)pent-4-enoic acid (**3u**)**:** The title compound was prepared from 2-cyclohexyl-4,4,5,5-tetramethyl-1,3,2-dioxaborolane (**1a**, 84.0 mg, 0.40 mmol, 2.0 equiv) and allyl 2-(4-fluorophenyl)acrylate (**2l**, 41.2 mg, 0.20 mmol, 1.0 equiv) according to *General Procedure 4*. After *work-up 1*, purification by silica gel column chromatography (CH_2Cl_2 to 1% HCOOH/1% EtOAc in CH_2Cl_2) and subsequent PTLC (1st PTLC: 1% HCOOH/2% EtOAc in pentane, 2nd PTLC: 7% MeOH in CH_2Cl_2) gave the product (28.9 mg, 50%) as a colorless solid.

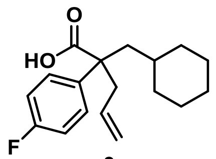

3u

^{1}H NMR (400 MHz, $CDCl_3$) δ 7.33 – 7.27 (m, 2H), 7.01 (t, J = 8.7 Hz, 2H), 5.49 (ddt, J = 17.2, 10.2, 7.1 Hz, 1H), 5.09 – 4.97 (m, 2H), 2.82 (d, J = 7.2 Hz, 2H), 1.98 (dd, J = 14.2, 5.9 Hz, 1H), 1.89 (dd, J = 14.2, 5.2 Hz, 1H), 1.66 – 1.54 (m, 4H), 1.41 – 1.25 (m, 2H), 1.18 – 1.06 (m, 3H), 1.02 – 0.93 (m, 1H), 0.93 – 0.84 (m, 1H); **^{13}C NMR** (101 MHz, $CDCl_3$) δ 182.0, 161.8 (d, J = 246.1 Hz), 137.8 (d, J = 3.4 Hz), 133.4, 128.5 (d, J = 8.0 Hz), 118.9, 115.3 (d, J = 21.2 Hz), 52.9, 41.9, 39.6, 35.1, 34.2, 34.0, 26.6, 26.5, 26.3; **^{19}F NMR** (376 MHz, $CDCl_3$) δ −115.9; **HRMS** (ESI$^-$) calc'd for [M−H]$^-$ 289.1609, found 289.1608; **R$_f$** (1% HCOOH/2% EtOAc in CH_2Cl_2) = 0.28.

2-(Cyclohexylmethyl)-2-(naphthalen-1-yl)pent-4-enoic acid (**3v**)**:** The title compound was prepared from 2-cyclohexyl-4,4,5,5-tetramethyl-1,3,2-dioxaborolane (**1a**, 84.0 mg, 0.40 mmol, 2.0 equiv) and allyl 2-(naphthalen-1-yl)acrylate (**2m**, 47.7 mg, 0.20 mmol, 1.0 equiv) according to *General Procedure 4*. After *work-up 1*, purification by silica gel column chromatography (1st column: 1% HCOOH/1% EtOAc in CH_2Cl_2, 2nd column: 1% HCOOH/5% EtOAc in pentane) gave the product (53.2 mg, 82%) as a colorless oil.

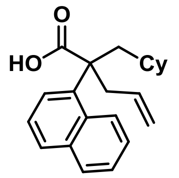

3v

^{1}H NMR (400 MHz, $CDCl_3$) δ 7.98 (d, J = 8.6 Hz, 1H), 7.87 (dd, J = 8.1, 1.5 Hz, 1H), 7.79 (d, J = 7.8 Hz, 1H), 7.52 – 7.35 (m, 4H), 5.57 – 5.42 (m, 1H), 5.08 – 4.96 (m, 2H), 3.13 (dd, J = 13.8, 6.4 Hz, 1H), 2.94 (dd, J = 13.8, 8.1 Hz, 1H), 2.19 (dd, J = 14.2, 5.3 Hz, 1H), 2.06 (dd, J = 14.2, 4.3 Hz, 1H), 1.71 – 1.64 (m, 1H), 1.63 – 1.55 (m, 1H), 1.53 – 1.45 (m, 1H), 1.43 – 1.34 (m, 1H), 1.32 – 1.24 (m, 1H), 1.22 – 1.10 (m, 1H), 1.09 – 0.89 (m, 4H), 0.70 – 0.56 (m, 1H); **^{13}C NMR** (101 MHz, $CDCl_3$) δ 183.2, 137.8, 134.4, 133.5, 131.7, 129.6, 128.6, 126.1, 125.3,

125.0, 124.7, 124.0, 118.8, 53.0, 41.4, 39.9, 35.4, 34.9, 33.3, 26.6, 26.4, 26.2; **HRMS** (ESI⁻) calc'd for [M−(HCO_2)]⁻ 277.1962, found 277.1957; **R_f** (1% HCOOH/1% EtOAc in CH_2Cl_2) = 0.50; **X-ray** (single-crystal) A colorless plate-like specimen of X-ray diffraction quality was obtained by slow evaporation of a solution of **3v** in $CDCl_3$ (CCDC 2033246).

2-(Cyclohexylmethyl)-2-(ethoxymethyl)pent-4-enoic acid (3w): The title compound was prepared from 2-cyclohexyl-4,4,5,5-tetramethyl-1,3,2-dioxaborolane (**1a**, 84.0 mg, 0.40 mmol, 2.0 equiv) and allyl 2-(ethoxymethyl)acrylate (**2n**, 34.0 mg, 0.20 mmol, 1.0 equiv) according to *General Procedure 4*. After *work-up 1*, purification by silica gel column chromatography (CH_2Cl_2 to 1% HCOOH/3% EtOAc in CH_2Cl_2) and subsequent PTLC (1% HCOOH/7% EtOAc in CH_2Cl_2) gave the product (20.1 mg, 40%) as a colorless oil.

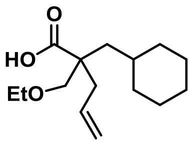

3w

^{1}H NMR (400 MHz, $CDCl_3$) δ 5.72 (ddt, J = 17.5, 10.3, 7.4 Hz, 1H), 5.15 – 5.04 (m, 2H), 3.55 – 3.37 (m, 4H), 2.48 (dd, J = 13.9, 7.3 Hz, 1H), 2.37 (dd, J = 13.7, 7.4 Hz, 1H), 1.69 – 1.58 (m, 5H), 1.56 – 1.43 (m, 2H), 1.41 – 1.33 (m, 1H), 1.26 – 1.16 (m, 5H), 1.15 – 1.07 (m, 1H), 0.99 – 0.87 (m, 2H); **^{13}C NMR** (101 MHz, $CDCl_3$) δ 180.9, 133.6, 118.7, 71.6, 67.0, 49.5, 41.1, 37.7, 34.6, 34.4, 33.8, 26.6, 26.5, 26.4, 15.1; **HRMS** (ESI⁻) calc'd for [M−H]⁻ 253.1809, found 253.1807; **R_f** (1% HCOOH/3% EtOAc in CH_2Cl_2) = 0.38.

2-(Cyclohexylmethyl)-2-isopropylpent-4-enoic acid (3x): The title compound was prepared from 2-cyclohexyl-4,4,5,5-tetramethyl-1,3,2-dioxaborolane (**1a**, 84.0 mg, 0.40 mmol, 2.0 equiv) and allyl 3-methyl-2-methylenebutanoate (**2o**, 30.8 mg, 0.20 mmol, 1.0 equiv) according to *General Procedure 4*. After *work-up 1*, purification by silica gel column chromatography (CH_2Cl_2 to 1% HCOOH/1% EtOAc in pentane) gave the product (30.7 mg, 64%) as a colorless oil.

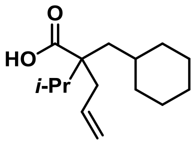

3x

^{1}H NMR (400 MHz, $CDCl_3$) δ 5.86 – 5.72 (m, 1H), 5.17 – 5.02 (m, 2H), 2.53 (dd, J = 15.0, 8.3 Hz, 1H), 2.43 (dd, J = 15.0, 6.1 Hz, 1H), 1.88 (hept, J = 6.9 Hz, 1H), 1.81 – 1.73 (m, 1H), 1.69 – 1.57 (m, 5H), 1.43 – 1.33 (m, 2H), 1.27 – 0.99 (m, 5H), 0.97 (d, J = 6.9 Hz, 3H), 0.90 (d, J = 6.9 Hz, 3H); **^{13}C NMR** (101 MHz, $CDCl_3$) δ 183.3, 135.0, 117.9, 50.8, 41.3, 35.5, 35.4, 34.5, 33.8, 33.3, 26.7, 26.6, 26.5, 18.0, 17.9; **HRMS** (ESI⁻) calc'd for [M−H]⁻ 237.1860, found 237.1857; **R_f** (1% HCOOH/1% EtOAc in CH_2Cl_2) = 0.49.

2-(Cyclohexylmethyl)pent-4-enoic acid (**3y**)**:** The title compound was prepared from 2-cyclohexyl-4,4,5,5-tetramethyl-1,3,2-dioxaborolane (**1a**, 84.0 mg, 0.40 mmol, 2.0 equiv) and allyl acrylate (**2p**, 22.4 mg, 0.20 mmol, 1.0 equiv) according to *General Procedure 4*. The reaction was performed in a THF/MeCN (1/1, 0.05 M) solvent mixture. After *work-up 2*, purification by silica gel column chromatography (CH_2Cl_2 to 1% HOAc in CH_2Cl_2) and subsequent PTLC (1% HCOOH/7% EtOAc in pentane) gave the product (17.3 mg, 44%) as a colorless oil.

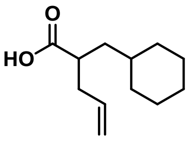

3y

^{1}H NMR (400 MHz, $CDCl_3$) δ 5.76 (ddt, J = 17.1, 10.2, 7.0 Hz, 1H), 5.12 – 4.99 (m, 2H), 2.62 – 2.52 (m, 1H), 2.36 (dt, J = 14.8, 7.5 Hz, 1H), 2.23 (dt, J = 14.1, 6.8 Hz, 1H), 1.83 – 1.75 (m, 1H), 1.72 – 1.55 (m, 5H), 1.37 – 1.10 (m, 6H), 0.97 – 0.78 (m, 2H); **^{13}C NMR** (101 MHz, $CDCl_3$) δ 182.2, 135.3, 117.1, 42.7, 39.5, 36.9, 35.6, 33.7, 33.0, 26.7, 26.4, 26.3; **HRMS** (ESI^-) calc'd for $[M-H]^-$ 195.1391, found 195.1388; **R_f** (0.5% HOAc/3% EtOAc in CH_2Cl_2) = 0.31.

2.5.2 Photoredox-enabled 1,2-dialkylation of α-substituted acrylates – α-Silyl Amine Variant (General Procedure 5)

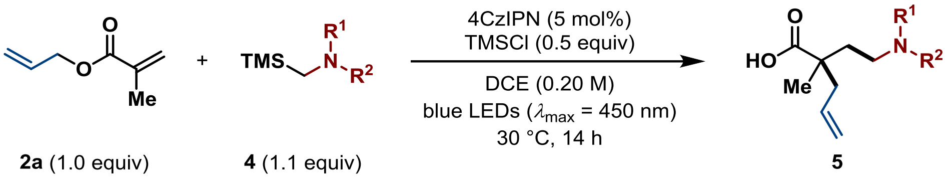

To an oven-dried 10 mL Schlenk tube equipped with a Teflon-coated magnetic stir were added 4CzIPN (11.7 mg, 0.015 mmol, 5 mol%), and the respective α-silyl amine (if solid, 0.33 mmol, 1.1 equiv). The tube was evacuated and backfilled with argon three times. Subsequently, dry DCE (1.5 mL), the respective α-silyl amine (if liquid, 0.33 mmol, 1.1 equiv), allyl methacrylate (**2a**, 40.2 µL, 0.30 mmol, 1.0 equiv), and TMSCl (19 µL, 0.15 mmol, 0.5 equiv) were added. The reaction mixture was degassed by three freeze-pump-thaw cycles and then stirred and irradiated with blue LEDs (30 W, λ_{max} = 450 nm) for 14 h. The solvent was removed through a stream of argon, 1,3,5-trimethoxybenzene (50.4 mg, 0.30 mmol, 1.0 equiv) or mesitylene (42.0 µL, 0.30 mmol, 1.0 equiv) was added as internal standard, and the ^{1}H NMR yield was determined. The NMR tube, used pipettes, and the Schlenk tube were carefully rinsed with CH_2Cl_2 into a round bottom flask. MeOH (1.0 mL) was added, and the mixture was stirred for at least 1 h at room temperature. The solvent was removed *in vacuo*, and the residue was purified by silica gel column chromatography and/or preparative thin-layer chromatography (PTLC) to afford the product.

esterification: To ease the purification, some products were converted into their corresponding methyl ester. After removing the solvent through a stream of argon, the Schlenk tube was evacuated and backfilled with argon three times. Dry MeOH (1.5 mL) and TMSCl (0.20 mL, 1.6 mmol, 5.3 equiv) were added under an argon atmosphere, and the mixture was stirred at 65 °C overnight. The reaction was quenched with sat. aq. $NaHCO_3$ and extracted with CH_2Cl_2 (5×). The combined org. layers were concentrated *in vacuo*, and purification by silica gel column chromatography afforded the product.[33]

Methyl 2-methyl-2-(2-morpholinoethyl)pent-4-enoate (5a): The title compound was prepared from 4-((trimethylsilyl)methyl)morpholine (**4a**, 57.2 mg, 0.33 mmol, 1.1 equiv) according to *General Procedure 5*. After esterification (see *esterification*), purification by silica gel column chromatography (8% EtOH/32% EtOAc in pentane) gave the product (52.4 mg, 72%) as a slightly yellow oil.

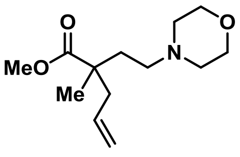

5a

^{1}H NMR (300 MHz, $CDCl_3$) δ 5.77 – 5.59 (m, 1H), 5.10 – 4.98 (m, 2H), 3.70 – 3.63 (m, 7H), 2.47 – 2.38 (m, 4H), 2.36 – 2.14 (m, 4H), 1.94 – 1.82 (m, 1H), 1.65 – 1.54 (m, 1H), 1.14 (s, 3H); **^{13}C NMR** (75 MHz, $CDCl_3$) δ 177.1, 133.7, 118.4, 67.0, 54.8, 54.0, 51.8, 44.9, 43.9, 35.2, 21.4; **HRMS** (ESI^+) calc'd for $[M+H]^+$ 242.1751, found 242.1751; **R_f** (12% EtOH/48% EtOAc/40% pentane) = 0.50.

2-Methyl-2-(2-thiomorpholinoethyl)pent-4-enoic acid (5b): The title compound was prepared from 4-((trimethylsilyl)methyl)thiomorpholine (**4b**, 62.4 mg, 0.33 mmol, 1.1 equiv) according to *General Procedure 5*. Purification by silica gel column chromatography (5% MeOH in CH_2Cl_2) gave the product (48.2 mg, 66%) as a slightly brown solid.

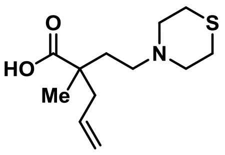

5b

^{1}H NMR (400 MHz, $CDCl_3$) δ 14.30 (bs, 1H), 5.80 – 5.64 (m, 1H), 5.07 – 4.98 (m, 2H), 3.01 – 2.85 (m, 4H), 2.74 (t, J = 5.1 Hz, 4H), 2.59 (t, J = 7.4 Hz, 2H), 2.31 (dd, J = 13.6, 7.2 Hz, 1H), 2.22 (dd, J = 13.7, 7.6 Hz, 1H), 1.82 (dt, J = 14.4, 7.3 Hz, 1H), 1.60 (dt, J = 14.3, 7.3 Hz, 1H), 1.09 (s, 3H); **^{13}C NMR** (101 MHz, $CDCl_3$) δ 179.4, 134.4, 118.1, 54.1, 54.0, 45.1, 43.9, 33.0, 26.4, 22.5; **HRMS** (ESI^-) calc'd for $[M-H]^-$ 242.1209, found 242.1218; **R_f** (10% MeOH in CH_2Cl_2) = 0.30; **X-ray** (single-crystal) A colorless needle-like specimen of X-ray diffraction quality was obtained by slow evaporation of a solution of **5b** in $CDCl_3$ (CCDC 2033247).

Methyl 2-methyl-2-(2-(4-phenylpiperazin-1-yl)ethyl)pent-4-enoate (5c): The title compound was prepared from 1-phenyl-4-((trimethylsilyl)methyl)piperazine (**4c**, 81.8 mg, 0.33 mmol, 1.1 equiv) according to *General Procedure 5*. After esterification (see *esterification*), purification by silica gel column chromatography (15% EtOAc in pentane) gave the product (49.3 mg, 52%) as a colorless oil.

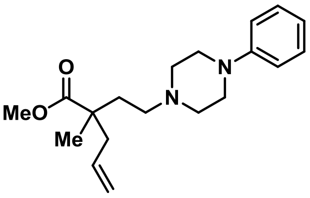

5c

^{1}H NMR (400 MHz, $CDCl_3$) δ 7.32 – 7.23 (m, 2H), 6.94 (d, J = 7.9 Hz, 2H), 6.87 (t, J = 7.3 Hz, 1H), 5.82 – 5.67 (m, 1H), 5.13 – 5.05 (m, 2H), 3.69 (s, 3H), 3.24 – 3.16 (m, 4H), 2.64 – 2.58 (m, 4H), 2.47 – 2.31 (m, 3H), 2.25 (dd, J = 13.7, 7.7 Hz, 1H), 1.97 (ddd, J = 13.4, 9.9, 5.9 Hz, 1H), 1.68 (ddd, J = 13.4, 9.8, 5.4 Hz, 1H), 1.20 (s, 3H); **^{13}C NMR** (101 MHz, $CDCl_3$) δ 177.1, 151.4, 133.8, 129.2, 119.7, 118.4, 116.1, 54.4, 53.5, 51.8, 49.2, 44.9, 43.8, 35.7, 21.4; **HRMS** (ESI$^+$) calc'd for $[M+H]^+$ 317.2224, found 317.2222; **R$_f$** (30% EtOAc in pentane) = 0.48.

2-(2-(4-(5-Bromopyrimidin-2-yl)piperazin-1-yl)ethyl)-2-methylpent-4-enoic acid (5d): The title compound was prepared from 5-bromo-2-(4-((trimethylsilyl)methyl)piperazin-1-yl)pyrimidine (**4d**, 108.6 mg, 0.33 mmol, 1.1 equiv) according to *General Procedure 5*. Purification by PTLC (1st PTLC: 3 runs 1% HOAc/10% EtOH/39% EtOAc in CH_2Cl_2, 2nd PTLC: 10% MeOH in CH_2Cl_2) gave the product (47.4 mg, 41%) as a colorless solid.

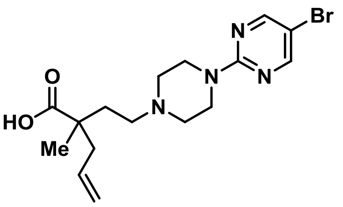

5d

^{1}H NMR (400 MHz, $CDCl_3$) δ 13.05 (bs, 1H), 8.27 (s, 2H), 5.75 (ddt, J = 17.4, 10.3, 7.3 Hz, 1H), 5.11 – 5.01 (m, 2H), 3.98 – 3.80 (m, 4H), 2.80 – 2.66 (m, 4H), 2.67 – 2.52 (m, 2H), 2.37 (dd, J = 13.7, 7.0 Hz, 1H), 2.26 (dd, J = 13.7, 7.6 Hz, 1H), 1.94 – 1.83 (m, 1H), 1.73 – 1.64 (m, 1H), 1.13 (s, 3H); **^{13}C NMR** (101 MHz, $CDCl_3$) δ 179.2, 159.7, 158.1, 134.3, 118.3, 106.5, 53.5, 52.0, 45.2, 43.8, 42.7, 33.7, 22.4; **HRMS** (ESI$^-$) calc'd for $[M-H]^-$ 381.0932, found 381.0927; **R$_f$** (10% MeOH in CH_2Cl_2) = 0.61.

2-(2-(Dibutylamino)ethyl)-2-methylpent-4-enoic acid (5e): The title compound was prepared from *N*-butyl-*N*-((trimethylsilyl)methyl)butan-1-amine (**4e**, 71.1 mg, 0.33 mmol, 1.1 equiv), and dry THF (1.5 mL) as solvent according to *General Procedure 5*. Purification by PTLC (1st run: 20% EtOH in EtOAc, 2nd run: 10% MeOH in CH_2Cl_2) gave the product (46.2 mg, 57%) as a slightly brown oil.

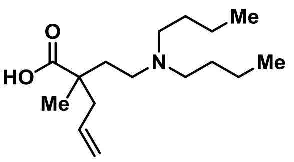

5e

^{1}H NMR (400 MHz, $CDCl_3$) δ 5.85 – 5.67 (m, 1H), 5.04 – 4.95 (m, 2H), 2.99 – 2.68 (m, 6H), 2.37 (dd, J = 13.9, 7.1 Hz, 1H), 2.24 (dd, J = 13.8, 7.4 Hz, 1H), 1.91 – 1.80 (m, 1H), 1.65 – 1.48 (m, 5H), 1.36 – 1.25 (m, 4H), 1.13 (s, 3H), 0.90 (t, J = 7.3 Hz, 6H); **^{13}C NMR** (101 MHz, $CDCl_3$) δ 180.6, 135.1, 117.5, 51.8, 49.7, 45.5, 44.5, 31.6, 25.7, 24.0, 20.3, 13.7; **HRMS** (ESI$^+$) calc'd for $[M+Na]^+$ 292.2247, found 292.2243; **R$_f$** (10% MeOH in CH_2Cl_2) = 0.69.

2-(2-(Diphenylamino)ethyl)-2-methylpent-4-enoic acid (5f): The title compound was prepared from *N*-phenyl-*N*-((trimethylsilyl)methyl)aniline (**4f**, 84.2 mg, 0.33 mmol, 1.1 equiv) according to *General Procedure 5*. Purification by silica gel column chromatography (2% EtOAc in CH_2Cl_2) gave the product (19.5 mg, 21%) as colorless oil.

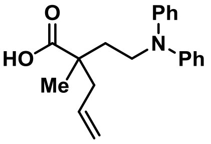

5f

^{1}H NMR (400 MHz, $CDCl_3$) δ 7.26 (t, J = 8.0 Hz, 4H), 7.03 – 6.97 (m, 4H), 6.94 (t, J = 7.3 Hz, 2H), 5.81 – 5.66 (m, 1H), 5.13 – 5.04 (m, 2H), 3.82 – 3.72 (m, 2H), 2.43 (dd, J = 13.8, 7.1 Hz, 1H), 2.29 (dd, J = 13.8, 7.7 Hz, 1H), 2.14 – 2.04 (m, 1H), 1.92 – 1.82 (m, 1H), 1.24 (s, 3H); **^{13}C NMR** (126 MHz, $CDCl_3$) δ 182.9, 147.9, 133.2, 129.4, 121.4, 120.9, 119.0, 48.2, 44.7, 43.3, 35.2, 21.6; **HRMS** (ESI$^-$) calc'd for $[M-H]^-$ 308.1656, found 308.1651; **R$_f$** (2% EtOAc in CH_2Cl_2) = 0.23.

2-Methyl-2-(2-(4-(2-morpholino-2-oxoethyl)piperazin-1-yl)ethyl)pent-4-enoic acid (5g): The title compound was prepared from 1-morpholino-2-(4-((trimethylsilyl)methyl)piperazin-1-yl)ethan-1-one (**4g**, 98.7 mg, 0.33 mmol, 1.1 equiv) according to *General Procedure 5*. Purification by PTLC (2 runs 15% MeOH in CH_2Cl_2) gave the product (56.2 mg, 53%) as a colorless solid.

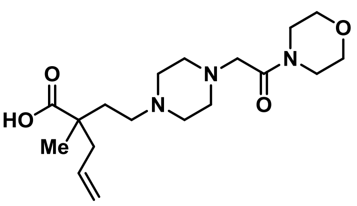

5g

^{1}H NMR (400 MHz, $CDCl_3$) δ 12.93 (bs, 1H), 5.74 (ddt, J = 19.0, 9.4, 7.3 Hz, 1H), 5.10 – 4.98 (m, 2H), 3.68 – 3.60 (m, 4H), 3.60 – 3.46 (m, 4H), 3.16 (d, J = 2.6 Hz, 2H), 3.10 – 2.36 (m, 10H), 2.36 – 2.23 (m, 2H), 1.82 (dt, J = 14.1, 7.0 Hz, 1H), 1.60 (dt, J = 14.1, 7.0 Hz, 1H), 1.11 (s, 3H); **^{13}C NMR** (101 MHz, $CDCl_3$) δ 179.3, 167.6, 134.6, 118.1, 67.0, 66.8, 59.9, 53.3, 52.0, 51.8, 46.0, 45.3, 44.1, 42.2, 33.3, 22.8; **HRMS** (ESI$^+$) calc'd for $[M+Na]^+$ 376.2207, found 376.2206; **R$_f$** (2 runs 15% MeOH in CH_2Cl_2) = 0.52.

2-Methyl-2-(2-(methyl(3-phenyl-3-(4-(trifluoromethyl)phenoxy)propyl)amino)ethyl)pent-4-enoic acid (5h): The title compound was prepared from *N*-methyl-3-phenyl-3-(4-(trifluoromethyl)phenoxy)-*N*-((trimethylsilyl)methyl)propan-1-amine (**4h**, 67.2 mg, 0.17 mmol, 1.7 equiv) according to *General Procedure 5* on

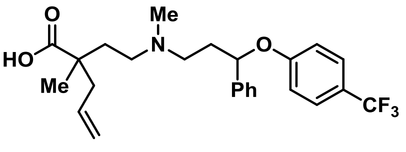

5h

a 0.10 mmol scale. Purification by PTLC (1st PTLC: 10% MeOH in CH_2Cl_2, 2nd PTLC: 15% MeOH in CH_2Cl_2) gave the product (19.8 mg, 44%) as colorless oil as a 50:50 mixture of diastereomers.

^{1}H NMR (400 MHz, $CDCl_3$, both diastereomers) δ 7.42 (d, J = 8.7 Hz, 2H), 7.36 – 7.32 (m, 4H), 7.30 – 7.26 (m, 1H), 6.88 (d, J = 8.5 Hz, 2H), 5.85 – 5.64 (m, 1H), 5.30 (dt, J = 8.7, 4.5 Hz, 1H), 5.08 – 4.94 (m, 2H), 2.97 – 2.81 (m, 2H), 2.79 – 2.68 (m, 2H), 2.44 (d, J = 1.9 Hz, 3H), 2.41 – 2.32 (m, 1H), 2.31 – 2.23 (m, 2H), 2.21 – 2.09 (m, 1H), 1.82 (dt, J = 13.9, 6.7 Hz, 1H), 1.59 (dt, J = 14.7, 6.1 Hz, 1H), 1.14 (d, J = 12.0 Hz, 3H); **^{13}C NMR** (101 MHz, $CDCl_3$, both diastereomers) δ 179.7, 160.1, 140.0, 140.0, 134.8, 134.7, 129.1, 128.4, 127.0 (q, J = 3.8 Hz), 125.9, 124.4 (q, J = 270.9 Hz), 123.3 (q, J = 32.6 Hz), 118.0, 118.0, 115.9, 78.0, 77.9, 53.3, 53.3, 53.0, 53.0, 45.6, 45.5, 44.5, 44.4, 40.6, 40.5, 34.5, 34.4, 32.7, 24.0, 23.9 – some signals are missing due to overlap; **^{19}F NMR** (376 MHz, $CDCl_3$) δ −61.6, −61.6; **HRMS** (ESI$^+$) calc'd for $[M+Na]^+$ 472.2070, found 472.2069; **R$_f$** (10% MeOH in CH_2Cl_2) = 0.24.

2-Methyl-2-(2-(piperidin-1-yl)propyl)pent-4-enoic acid (5i): The title compound was prepared from 1-(1-(trimethylsilyl)ethyl)piperidine (**4i**, 60.1 mg, 0.33 mmol, 1.1 equiv) according to *General Procedure 5*. Purification by PTLC (1st PTLC: 15% MeOH in CH_2Cl_2, 2nd PTLC: 2 runs 40% EtOH in EtOAc) gave the product (17.8 mg, 25%) as a brown oil as a 50:50 mixture of diastereomers.

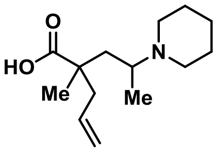

5i

^{1}H NMR (400 MHz, $CDCl_3$, both diastereomers) δ 5.87 – 5.71 (m, 1H), 5.12 – 4.97 (m, 2H), 3.22 – 2.87 (m, 3H), 2.87 – 2.15 (m, 4H), 2.15 – 1.67 (m, 6H), 1.29 – 1.00 (m, 8H); **^{13}C NMR** (101 MHz, $CDCl_3$, both diastereomers) δ 180.8, 180.5, 135.7, 134.4, 118.1, 117.6, 57.3, 57.1, 52.8, 47.2, 46.5, 46.4, 44.6, 42.5, 39.2, 38.7, 26.4, 24.9, 24.1, 23.4, 14.9, 14.5 – some signals are missing due to overlap; **HRMS** (ESI$^-$) calc'd for $[M-H]^-$ 238.1812, found 238.1810; **R$_f$** (20% MeOH in CH_2Cl_2) = 0.63.

2.6 Additive-Based Robustness Screen

First in 2013, our group developed an intermolecular additive-based robustness screen to evaluate the functional group preservation and the robustness of a chemical transformation.[34,35] Therefore, selected additives were added to a present reaction and the remaining additive (functional group tolerance) as well as the product yield (robustness) were determined.

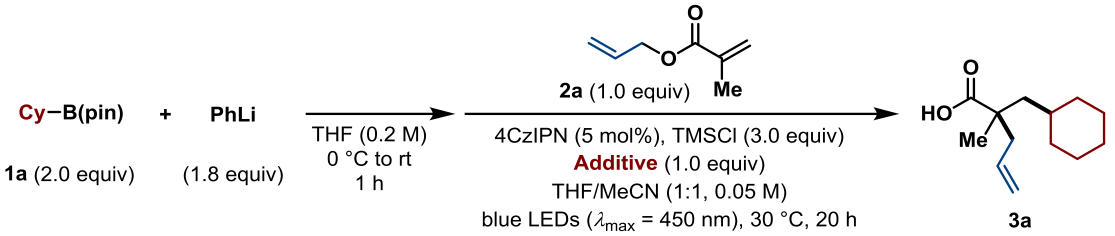

For the reaction set-up two stock solutions were prepared.

stock solution 1: To an oven-dried Schlenk flask equipped with a Teflon-coated magnetic stir bar were added 2-cyclohexyl-4,4,5,5-tetramethyl-1,3,2-dioxaborolane (**1a**, 756 mg, 3.6 mmol, 2.0 equiv), and dry THF (18 mL) under an argon atmosphere. The solution was cooled to 0 °C, and a PhLi solution (1.9 M in dibutyl ether, 1.7 mL, 3.2 mmol, 1.8 equiv) was added dropwise. The mixture was stirred for 30 min at 0 °C, allowed to warm up to room temperature and stirred for another 30 min at this temperature.

stock solution 2: To an oven-dried Schlenk flask equipped with a Teflon-coated magnetic stir bar were added 4CzIPN (71.1 mg, 0.09 mmol, 5 mol%), allyl methacrylate (**2a**, 242 µL, 1.8 mmol, 1.0 equiv), and dry MeCN (18 mL) under an argon atmosphere. The mixture was stirred until everything had dissolved.

Both stock solutions were degassed by three freeze-pump-thaw cycles.

To an oven-dried 10 mL Schlenk tube equipped with a Teflon-coated magnetic stir bar were added *stock solution 1* (1 mL), *stock solution 2* (1 mL), the respective additive (0.10 mmol, 1.0 equiv), and TMSCl (40 µL, 0.30 mmol, 3.0 equiv). The reaction mixture was stirred at room temperature and irradiated with blue LEDs (30 W, λ_{max} = 450 nm) for 20 h. Mesitylene (14 µL, 0.10 mmol, 1.0 equiv) and 1,3,5-trimethoxy benzene (16.8 mg, 0.10 mmol, 1.0 equiv) were added as internal standards. GC-FID samples were prepared for the analysis of the remaining additive (entry 11 was filtered over Celite® instead of silica). Subsequently, the solvent was removed through a stream of argon, and the ^{1}H NMR product yield was determined (the

resulting suspension after addition of $CDCl_3$ was filtered over a pad of Celite® directly into the NMR tube).

Table S5. Results of the additive-based robustness screen.

entry	additive	% remaining additive	% product yield
**A1**	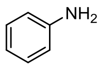	78	13
**A2**	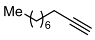	92	68
**A3**	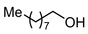	98	32
**A4**	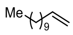	96	70
**A5**	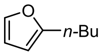	103	68
**A6**	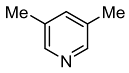	13	56
**A7**	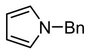	78	63
**A8**	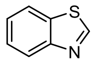	62	57
**A9**	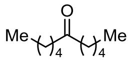	95	65
**A10**	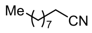	89	69
**A11**	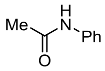	36	25
**A12**	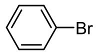	91	63
**A13**	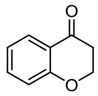	18	48
**A14**	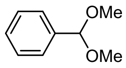	46	68
**A15**	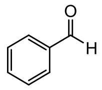	0	30
**16**	control		68
		**average**	**average**
		66	53

legend of the color code	
**additive:**	**product:**
<33	<23
33–66	23–45
>66	>45

2.7 Sensitivity Assessment

In 2019, a reaction-condition-based sensitivity assessment was published by our group to improve the reproducibility of chemical transformations.[36]

For the reaction set-up two stock solutions were prepared.

stock solution 1: To an oven-dried Schlenk flask equipped with a Teflon-coated magnetic stir bar were added 2-cyclohexyl-4,4,5,5-tetramethyl-1,3,2-dioxaborolane (**1a**, 1.01 g, 4.8 mmol, 2.0 equiv), and dry THF (21.6 mL, 0.22 M) under an argon atmosphere. The mixture was cooled to 0 °C, and a PhLi solution (1.9 M in dibutyl ether, 2.3 mL, 4.3 mmol, 1.8 equiv) was added dropwise. The mixture was stirred for 30 min at 0 °C and was then allowed to warm up to room temperature for further 30 min.

stock solution 2: To an oven-dried Schlenk flask equipped with a Teflon-coated magnetic stir bar were added 4CzIPN (94.8 mg, 0.20 mmol, 5 mol%), allyl methacrylate (**2a**, 322 µL, 2.4 mmol, 1.0 equiv), and dry MeCN (21.6 mL, 0.11 M) under an argon atmosphere. The mixture was stirred until everything had dissolved.

Standard conditions: Until otherwise stated, all reactions were carried out in oven-dried 10 mL Schlenk tubes. The Schlenk tubes were charged with 1.8 mL of each stock solution, and subsequently TMSCl (80 µL, 0.60 mmol, 3.0 equiv) was slowly added to the solution. The reaction conditions were adjusted as shown in table S6 before three freeze-pump-thaw cycles were carried out. The reactions were placed in photoreaction set-up (all in the same distance to the lamps) stirred and irradiated with blue LEDs (30 W, λ_{max} = 450 nm) for 20 h. The solvent was removed through a stream of argon, 1,3,5-trimethoxybenzene (33.6 mg, 0.20 mmol, 1.0 equiv) was added as internal standard and the ^{1}H NMR yield was determined (the resulting suspension after addition of $CDCl_3$ was filtered over a pad of Celite® directly into the NMR tube).

The reactions from entry 8 and 9 were carried out in cooling-jacketed Schlenk tubes. The control reaction (entry 9) was not cooled by water flow. To entries 3–11 were added dry THF (0.2 mL), and dry MeCN (0.2 mL) to reach the standard concentration (0.05 M).

Big scale: To an oven-dried 150 mL Schlenk tube equipped with a Teflon-coated magnetic stir bar were added 2-cyclohexyl-4,4,5,5-tetramethyl-1,3,2-dioxaborolane (**1a**, 1.68 g, 8.0 mmol, 2.0 equiv) and dry THF (40 mL) under an argon atmosphere. The reaction was cooled 0 °C and a PhLi solution (1.9 M in dibutyl ether, 3.8 mL, 7.2 mmol, 1.8 equiv) was added dropwise. The mixture was stirred for 30 min at 0 °C and then allowed to warm up to room temperature and stirred for further 30 min. Subsequently, dry MeCN (40 mL), 4CzIPN (158 mg, 0.2 mmol, 5 mol%), allyl methacrylate (**2a**, 536 µL, 4.0 mmol, 1.0 equiv) and TMSCl (1.6 mL, 12 mmol, 3.0 equiv) were added. The reaction mixture was degassed by three freeze-pump-thaw cycles and subsequently stirred and irradiated with blue LEDs (30 W, λ_{max} = 450 nm) for 20 h. The ^{1}H NMR yield was determined using 1,3,5-trimethoxybenzene. To the remaining reaction solution were added MeOH (20 mL) and HCOOH (2.0 mL) and the mixture was stirred overnight. The solvent was removed *in vacuo* and purification by silica gel column chromatography (CH_2Cl_2 to 1% HCOOH/1% EtOAc in CH_2Cl_2), acid/base work-up and another silica gel column chromatography (1% HCOOH/2% EtOAc in pentane) gave the product (585 mg, 70%) as a colorless oil.

Table S6. Sensitivity Screen reaction set-up and results.

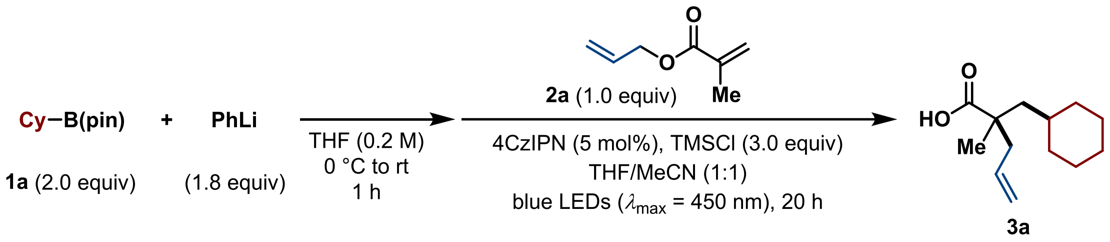

entry	modification	execution	% yield	% deviation
1	high *c*	no additional solvent	70	−4
2	low *c*	+0.4 mL THF/MeCN each	66	−10
3	high H_2O	+10 µL H_2O	0	−100
4	med. O_2	no degassing	66	−10
5	high O_2	+30 mL air	36	−51
6	control	---	73	---
7	high *T*	43 °C	70	−4
8	low *T*	18 °C	76	−4
9	low *T* (control)	(30 °C)	79	---
10	low *I*	*d* = 32 cm	67	−8
11	high *I*	*d* = 2 cm	62	−15
12	big scale	4.0 mmol scale	78	+7

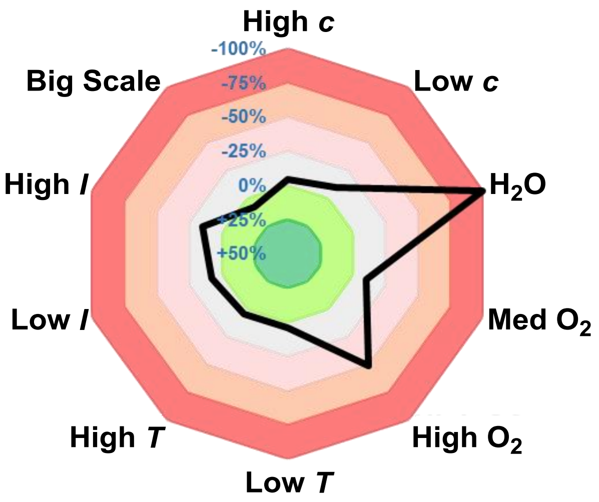

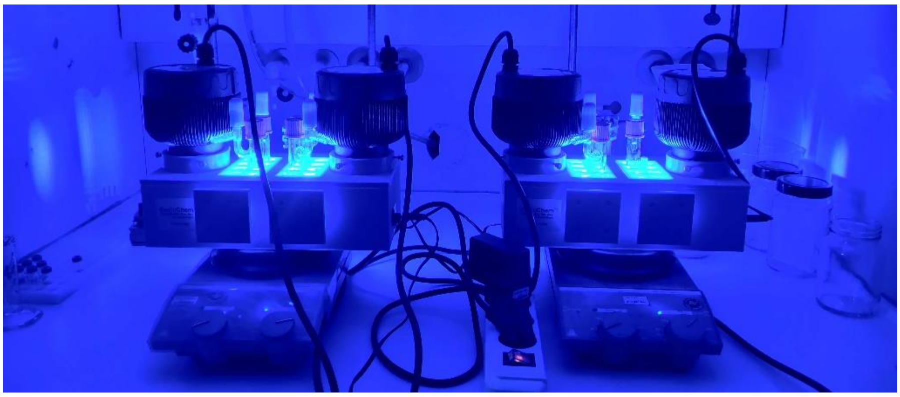

Figure S3. Photoreaction set-up for the sensitivity-assessment in a commercial EvoluChem™ PhotoRedOx Duo photobox (entries 1–6).

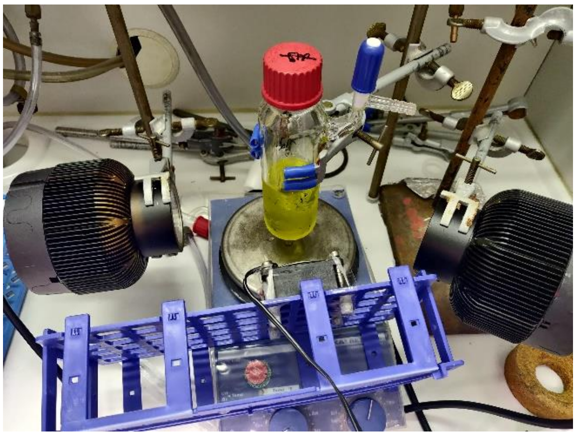

Figure S4. Big scale photoreaction set-up (d = 8 cm) with fan cooling (entry 12).

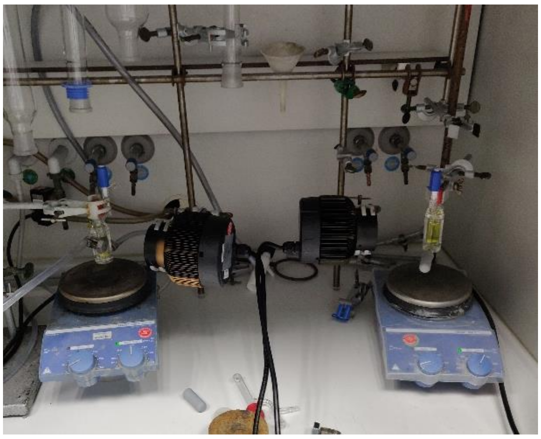

Figure S5. Low T photoreaction set-up with water cooling (entry 8) and control (entry 9, no water flow).

2.8 Crystal Structures

X-Ray diffraction: Data sets for compounds **3s**, **3v** and **5b** collected with a Bruker D8 Venture PHOTON III diffractometer. Programs used: data collection: APEX3 V2016.1-0[1] (Bruker AXS Inc., **2016**); cell refinement: SAINT V8.37A (Bruker AXS Inc., **2015**); data reduction: SAINT V8.37A (Bruker AXS Inc., **2015**); absorption correction, SADABS V2014/7 (Bruker AXS Inc., **2014**); structure solution *SHELXT-2015*;[2] structure refinement *SHELXL-2015*[3] and graphics, *XP*:[4] *R*-values are given for observed reflections, and wR^2 values are given for all reflections.

X-ray crystal structure analysis of 3s: A colorless prism-like specimen of $C_{18}H_{24}O_2$, approximate dimensions 0.054 mm × 0.067 mm × 0.161 mm, was used for the X-ray crystallographic analysis. The X-ray intensity data were measured on a Bruker D8 Venture PHOTON III Diffractometer system equipped with a micro focus tube Cu Ims (CuK_α, λ = 1.54178 Å) and a MX mirror monochromator. A total of 1300 frames were collected. The total exposure time was 19.07 h. The frames were integrated with the Bruker SAINT software package using a wide-frame algorithm. The integration of the data using a triclinic unit cell yielded a total of 12248 reflections to a maximum θ angle of 68.30° (0.83 Å resolution), of which 2832 were independent (average redundancy 4.325, completeness = 98.9%, R_{int} = 5.56%, R_{sig} = 4.36%) and 2262 (79.87%) were greater than $2\sigma(F^2)$. The final cell constants of a = 8.3649(2) Å, b = 10.0504(2) Å, c = 10.2831(3) Å, α = 73.682(2)°, β = 70.3480(10)°, γ = 86.6350(10)°, volume = 780.74(3) $Å^3$, are based upon the refinement of the XYZ-centroids of 5069 reflections above 20 σ(I) with 11.23° < 2θ < 136.5°. Data were corrected for absorption effects using the Multi-Scan method (SADABS). The ratio of minimum to maximum apparent transmission was 0.926. The calculated minimum and maximum transmission coefficients (based on crystal size) are 0.9130 and 0.9700. The structure was solved and refined using the Bruker SHELXTL Software Package, using the space group *P*-1, with Z = 2 for the formula unit, $C_{18}H_{24}O_2$. The final anisotropic full-matrix least-squares refinement on F^2 with 186 variables converged at R1 = 7.09%, for the observed data and wR2 = 18.95% for all data. The goodness-of-fit was 1.072. The largest peak in the final difference electron density synthesis was 0.645 $e^-/Å^3$ and the largest hole was −0.242 $e^-/Å^3$ with an RMS deviation of 0.055 $e^-/Å^3$. On the basis of the final model, the calculated density was 1.159 g/cm^3 and F(000), 296 e^-. The hydrogen at O2 atom was refined freely. **CCDC Nr.:** 2033245.

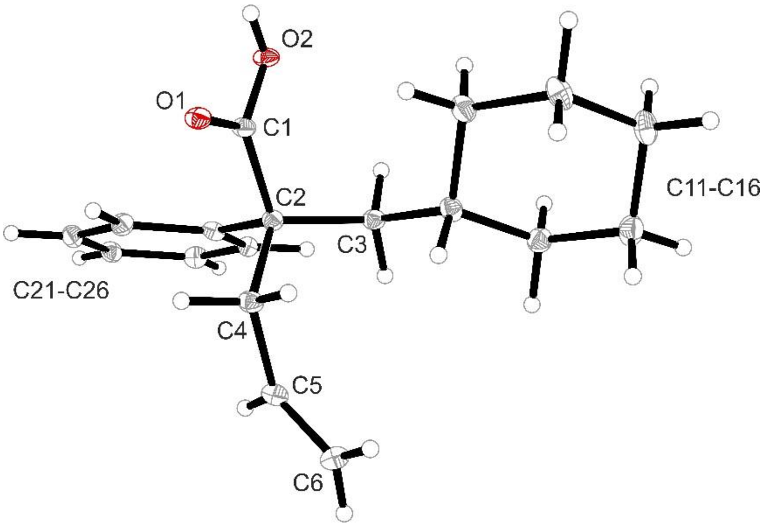

Figure S6. Crystal structure of compound **3s**. Thermal ellipsoids are shown at 15% probability.

X-ray crystal structure analysis of 3v: A colorless plate-like specimen of $C_{22}H_{26}O_2$, approximate dimensions 0.077 mm × 0.145 mm × 0.154 mm, was used for the X-ray crystallographic analysis. The X-ray intensity data were measured (λ = 1.54178 Å). A total of 1198 frames were collected. The total exposure time was 16.17 h. The frames were integrated with the Bruker SAINT software package using a wide-frame algorithm. The integration of the data using a monoclinic unit cell yielded a total of 26566 reflections to a maximum θ angle of 66.70° (0.84 Å resolution), of which 3174 were independent (average redundancy 8.370, completeness = 99.7%, R_{int} = 10.45%, R_{sig} = 4.81%) and 2277 (71.74%) were greater than $2\sigma(F^2)$. The final cell constants of a = 10.5885(4) Å, b = 8.3629(3) Å, c = 20.6387(7) Å, β = 99.927(2)°, volume = 1800.21(11) $Å^3$, are based upon the refinement of the XYZ-centroids of 6556 reflections above 20 σ(I) with 8.836° < 2θ < 133.1°. Data were corrected for absorption effects using the Multi-Scan method (SADABS). The ratio of minimum to maximum apparent transmission was 0.879. The calculated minimum and maximum transmission coefficients (based on crystal size) are 0.9160 and 0.9570. The structure was solved and refined using the Bruker SHELXTL Software Package, using the space group $P2_1/n$, with Z = 4 for the formula unit, $C_{22}H_{26}O_2$. The final anisotropic full-matrix least-squares refinement on F^2 with 221 variables converged at R1 = 4.69%, for the observed data and wR2 = 12.01% for all data. The goodness-of-fit was 1.040. The largest peak in the final difference electron density synthesis was 0.177 $e^-/Å^3$ and the largest hole was −0.214 $e^-/Å^3$ with an RMS deviation of 0.046 $e^-/Å^3$. On the basis of the final model, the calculated density was 1.190 g/cm^3 and F(000), 696 e^-. The hydrogen at O2 atom was refined freely. **CCDC Nr.:** 2033246.

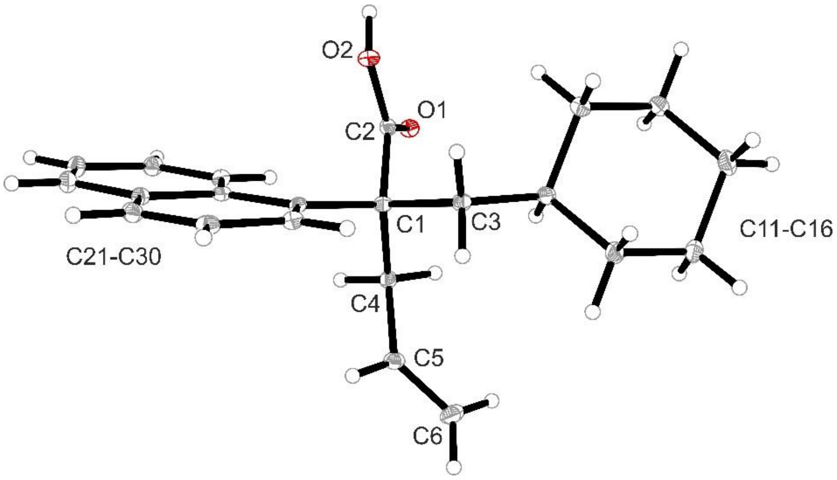

Figure S7. Crystal structure of compound **3v**. Thermal ellipsoids are shown at 15% probability.

X-ray crystal structure analysis of 5b: A colorless needle-like specimen of $C_{12}H_{21}NO_2S$, approximate dimensions 0.041 mm × 0.096 mm × 0.218 mm, was used for the X-ray crystallographic analysis. The X-ray intensity data were measured (λ = 1.54178 Å). A total of 1083 frames were collected. The total exposure time was 15.34 h. The frames were integrated with the Bruker SAINT software package using a wide-frame algorithm. The integration of the data using a triclinic unit cell yielded a total of 9493 reflections to a maximum θ angle of 66.63° (0.84 Å resolution), of which 2315 were independent (average redundancy 4.101, completeness = 99.5%, R_{int} = 9.27%, R_{sig} = 6.42%) and 1318 (56.93%) were greater than 2σ(F^2). The final cell constants of a = 6.3005(3) Å, b = 10.5228(5) Å, c = 10.9791(5) Å, α = 65.837(3)°, β = 82.374(3)°, γ = 88.124(4)°, volume = 658.07(6) $Å^3$, are based upon the refinement of the XYZ-centroids of 1477 reflections above 20 σ(I) with 8.904° < 2θ < 132.1°. Data were corrected for absorption effects using the Numerical Mu From Formula method (SADABS). The ratio of minimum to maximum apparent transmission was 0.829. The calculated minimum and maximum transmission coefficients (based on crystal size) are 0.6600 and 0.9200. The structure was solved and refined using the Bruker SHELXTL Software Package, using the space group *P*-1, with Z = 2 for the formula unit, $C_{12}H_{21}NO_2S$. The final anisotropic full-matrix least-squares refinement on F^2 with 266 variables converged at R1 = 6.43%, for the observed data and wR2 = 20.26% for all data. The goodness-of-fit was 1.035. The largest peak in the final difference electron density synthesis was 0.398 $e^-/Å^3$ and the largest hole was −0.198 $e^-/Å^3$ with an RMS deviation of 0.048 $e^-/Å^3$. On the basis of the final model, the calculated density was 1.228 g/cm^3 and F(000), 264 e^-. **CCDC Nr.:** 2033247.

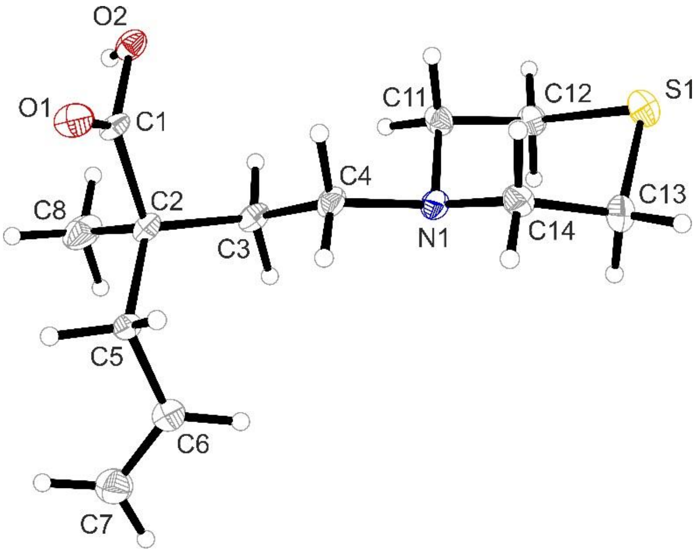

a)

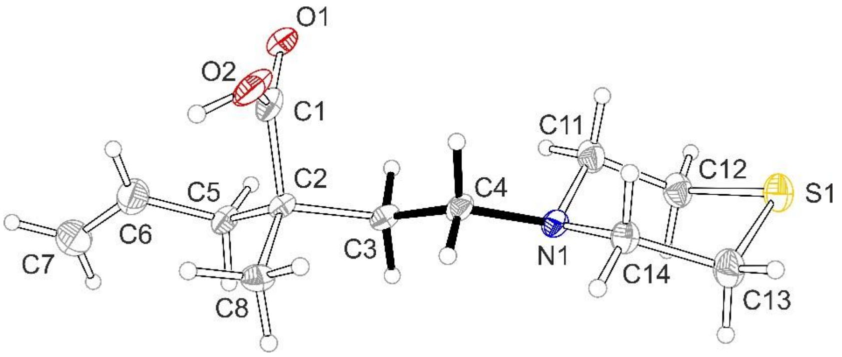

b)

Figure S8. Crystal structure of compound **5b**. A mixture (almost 50%) of two isomers was found in the asymmetric unit of compound **5b**.Thermal ellipsoids are shown at 15% probability.

References X-Ray Part:

[1] *APEX3* (**2016**), *SAINT* (**2015**) and *SADABS* (**2015**), Bruker AXS Inc., Madison, Wisconsin, USA.

[2] G. M. Sheldrick, *SHELXT – Integrated space-group and crystal-structure determination*, *Acta Cryst.* **2015**, *A71*, 3–8.

[3] G.M. Sheldrick, *Crystal structure refinement with SHELXL*, *Acta Cryst.* **2015**, *C71 (1)*, 3–8.

[4] *XP – Interactive molecular graphics, Version 5.1*, Bruker AXS Inc., Madison, Wisconsin, USA, **1998**.

3 Mechanistic Analysis

3.1 UV/Vis Absorption Spectroscopy

UV/Vis absorption spectra were recorded on a Jasco V-730 spectrophotometer, equipped with a temperature control unit at 25 °C. The samples were measured in Starna® fluorescence quartz cuvettes (type: 29-F, chamber volume = 1.400 mL, H × W × D = 48 mm × 12.5 mm × 12.5 mm, path length = 10 mm).

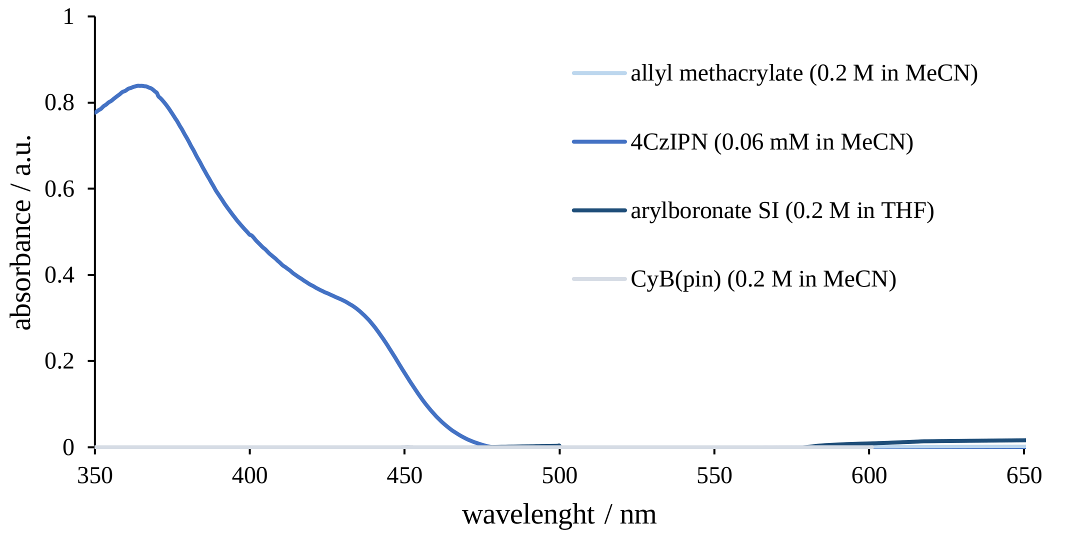

Figure S9. UV/Vis absorption spectra of the starting materials in isolation – alkyl–B(pin) variant.

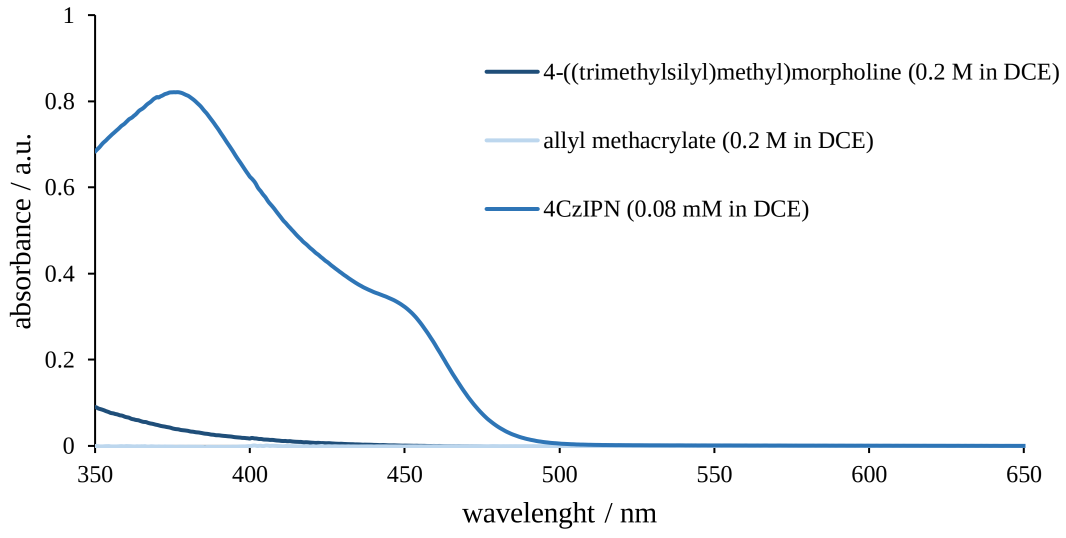

Figure S10. UV/Vis absorption spectra of the starting materials in isolation – α-silyl amine variant.

Figure S9 and S10 reveal that the photocatalyst (4CzIPN) is the only species, which absorbs light at around λ = 450 nm (intensity maximum of the used blue LEDs λ_{max} = 450 nm), implying that no other species is directly excited by the irradiation under the reaction conditions.

3.2 Stern–Volmer Luminescence Quenching Analysis

Stern–Volmer luminescence quenching analysis were carried out, to identify quenchers of the excited photoredox catalyst. Therefore the luminescence of the excited photocatalyst is measured in the presence of varying concentrations of potential quencher.

The quenching studies were carried out on a JASCO FP-8300 spectrofluorometer using Starna® fluorescence quartz cuvettes (type: 29-F, chamber volume = 1.400 mL, H × W × D = 48 mm × 12.5 mm × 12.5 mm, path length = 10 mm). The following parameters were set: data interval = 0.5 nm, scan-speed = 500 nm/min, excitation wavelength λ_{ex} = 420 nm, measured luminescence wavelength λ = 540 nm.

All samples were prepared in an argon-filled glovebox with degassed and dry solvents. The quenching studies were performed using a solution of 4CzIPN ($1\cdot10^{-5}$ M in the respective solvent). The varying concentrations of the potential quencher were achieved by diluting the respective stock solutions in the cuvettes. The samples were sealed with PTFE stoppers and removed from the glovebox for the measurement.

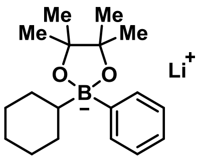

SI

Arylboronate complex (**SI**) was prepared as follows: To an oven-dried 10 mL Schlenk tube equipped with a Teflon-coated magnetic stir bar were added 2-cyclohexyl-4,4,5,5-tetramethyl-1,3,2-dioxaborolane (**1a**, 105 mg, 0.50 mmol, 1.0 equiv), and dry THF (3.0 mL) under an argon atmosphere. The solution was cooled to 0 °C, and a PhLi solution (1.9 M in dibutyl ether, 0.26 mL, 0.50 mmol, 1.0 equiv) was added dropwise while stirring. The mixture was stirred for 45 min at 0 °C and was then allowed to warm up to room temperature for further 15 min. After that, the solvent was removed *in vacuo* (*via* Schlenk line), and the resulting colorless solid was further dried overnight.

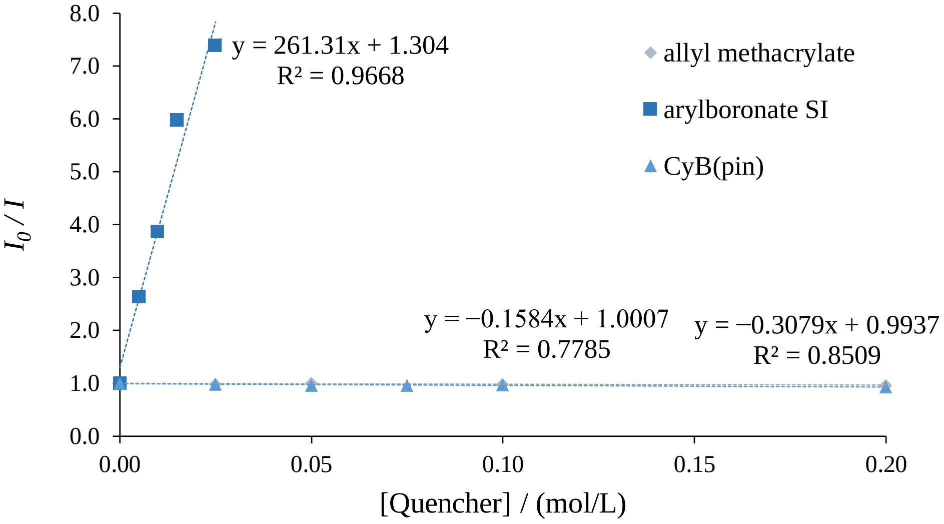

Figure S11. Stern–Volmer luminescence quenching – alkyl–B(pin) variant in MeCN.

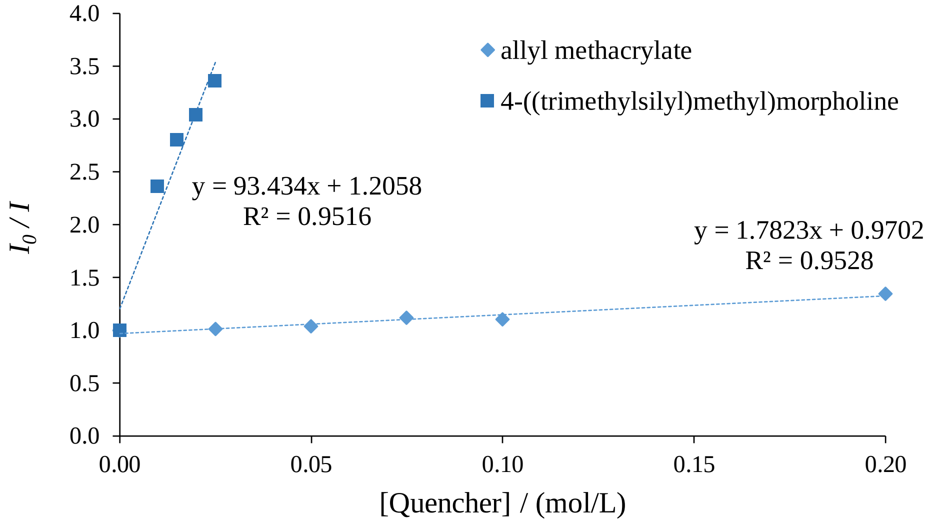

Figure S12. Stern–Volmer luminescence quenching – *α*-silyl amine variant in DCE.

The results for the alkyl–B(pin) variant demonstrate, that only the arylboronate complex (**SI**) quenches the excited photocatalyst.

In case of the *α*-silyl amines variant, both *α*-silyl amines and allyl methacrylate quench the photoredox catalyst. However, consideration of the Stern–Volmer constants suggests that quenching of the excited photocatalyst by the acrylate is negligible.

3.3 Light On/Off Experiment

To gain insights into how the product formation of the two transformation types depend on the irradiation, light on/off experiments were performed.

Light on/off experiment – alkyl–B(pin) variant

To an oven-dried 50 mL Schlenk tube equipped with a Teflon-coated magnetic stir bar were added 2-cyclohexyl-4,4,5,5-tetramethyl-1,3,2-dioxaborolane (**1a**, 0.45 mL, 2.0 mmol, 2.0 equiv) and dry THF (10 mL) under an argon atmosphere. The reaction was cooled 0 °C and a PhLi solution (1.9 M in dibutyl ether, 0.95 mL, 1.8 mmol, 1.8 equiv) was added dropwise. The mixture was stirred for 45 min at 0 °C and was then allowed to warm up to room temperature and stirred for 30 min. Subsequently, dry MeCN (10 mL), 4CzIPN (40 mg, 0.05 mmol, 5 mol%), 1,3,5-trimethoxybenzene as internal standard (168 mg, 1.0 mmol, 1.0 equiv), allyl methacrylate (**2a**, 134 µL, 1.0 mmol, 1.0 equiv) and TMSCl (0.40 mL, 3.0 mmol, 3.0 equiv) were added. The reaction mixture was degassed by three freeze-pump-thaw cycles. Subsequently, the reaction was stirred and alternately irradiated with blue LEDs (6×5 W, λ_{max} = 455 nm) or kept in the dark for the indicated time intervals (grey areas in figure S13). For each measuring point, 2.0 mL of the reaction solution were taken and quenched with 0.25 mL MeOH. The solvent was removed and the ^{1}H NMR yield was determined.

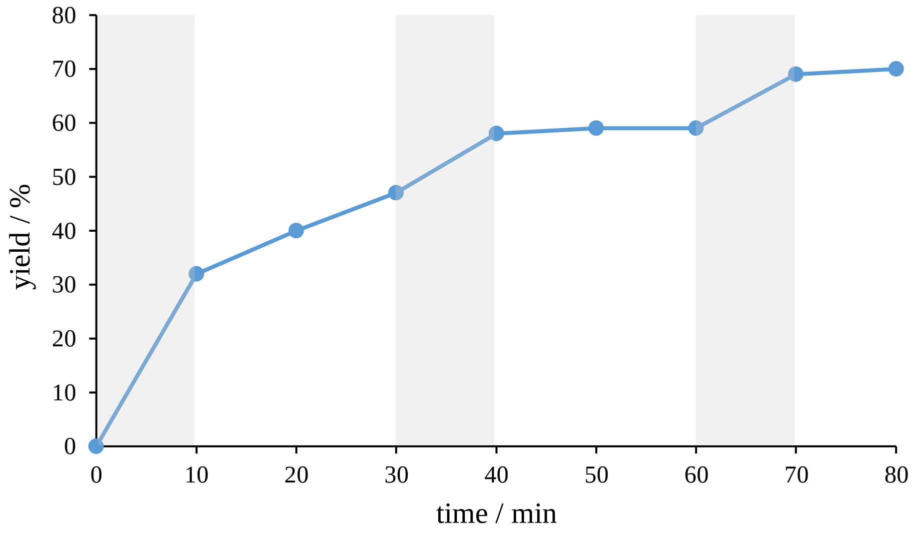

Figure S13. Results of the light on/off experiment – alkyl–B(pin) variant (grey areas indicate the irradiation intervals).

Following the first irradiation interval, product formation was still observed in the absence of light. After the second and third irradiation interval no further product formation was

determined in the dark. Although this experiment does not generally provide evidence for the existence of a radical chain mechanism, it should additionally be noted, that this transformation contains a thermal rearrangement step which could lead to product formation in the absence of light. Thus, conclusions regarding a radical chain mechanism cannot be drawn from this experiment.

Light on/off experiment – *α*-silyl amine variant

To an oven-dried 10 mL Schlenk tube equipped with a Teflon-coated magnetic stir were added 4CzIPN (40 mg, 0.05 mmol, 5 mol%) and 1,3,5-trimethoxybenzene as internal standard (168 mg, 1.0 mmol, 1.0 equiv). The tube was evacuated and backfilled with argon three times. Subsequently, dry DCE (5.0 mL), 4-((trimethylsilyl)methyl)morpholine (**4a**, 215 µL, 0.33 mmol, 1.1 equiv), allyl methacrylate (**2a**, 134 µL, 1.0 mmol, 1.0 equiv), and TMSCl (60 µL, 0.50 mmol, 0.5 equiv) were added and the reaction mixture was degassed by three freeze-pump-thaw cycles. The reaction was stirred and alternately irradiated with blue LEDs (30 W, λ_{max} = 450 nm) or kept in the dark for the indicated time intervals (grey areas in figure S14). For each measuring point, 0.5 mL of the reaction solution were taken and quenched with 0.25 mL MeOH. The solvent was removed and the ^{1}H NMR yield was determined.

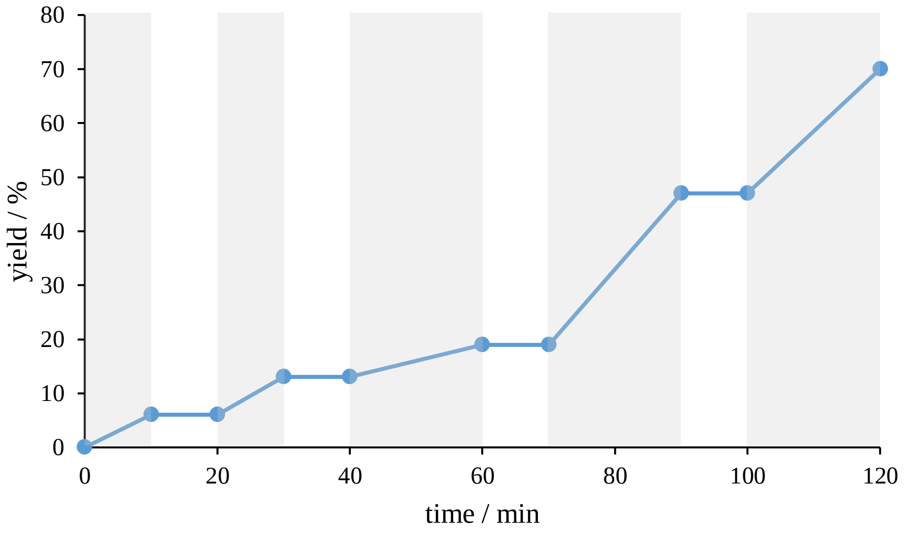

Figure S14. Results of the light on/off experiment – *α*-silyl amine variant (grey areas indicate the irradiation intervals).

No product formation was observed in the absence of light.

3.4 Quantum Yield Analysis

The photon flux of the blue LED (18 W, λ_{max} = 425 nm) was determined by standard ferrioxalate actinometry according to a modified literature procedure by Yoon and co-workers.[37] Therefore, two solutions were prepared and stored in the dark. All following steps were also carried out in a darkened lab to prevent undesired irradiation.

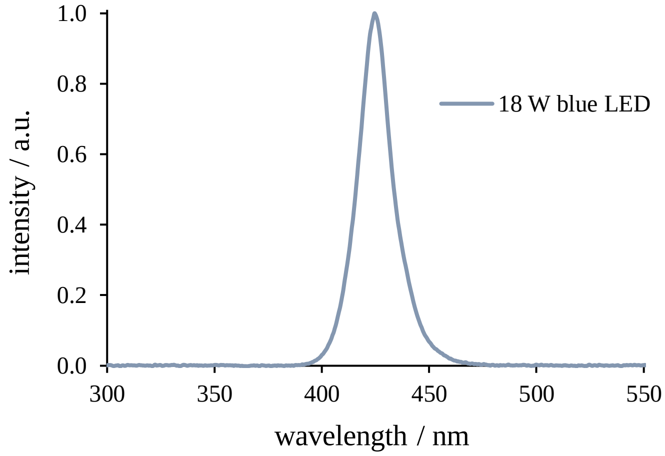

Figure S15. Emission spectrum of the blue LED (18 W) for quantum yield determination.

solution 1: Potassium ferrioxalate hydrate (737 mg, 1.50 mmol) was dissolve in aq. H_2SO_4 (0.05 M, 10 mL) to afford a 0.15 M ferrioxalate solution (attention: light sensitive!).

solution 2: 1,10-Phenanthroline monohydrate (25 mg, 0.13 mmol), NaOAc·3H_2O (5.63 g, 41.3 mmol) were dissolved in aq. H_2SO_4 (0.05 M, 25 mL).

To determine the photon flux the reduction of $[Fe(C_2O_4)_3]^{3-}$ into $[Fe(C_2O_4)_2]^{2-}$ by irradiation in a settled time is measured.[38,39] For that, *solution 1* (1 mL) was irradiated for 20 s at λ = 425 nm in a standard reaction Schlenk tube. Subsequently, s*olution 2* (175 µL) was added and the mixture was stirred for 1 h to ensure that all Fe(II)-ions were coordinated by phenanthroline. The solution was transferred into a quartz cuvette and the absorbance was measured at λ = 510 nm. The same procedure was repeated three times and also for a control sample, which has not been irradiated. The average absorbance of the three irradiated samples and the control sample were used to calculate the generated amount of Fe(II) ($n_{Fe(II)}$) according to the Lambert-Beer law (equation 1),

$$n_{Fe(II)} = \frac{V \cdot \Delta A_{510\,\mathrm{nm}}}{l \cdot \varepsilon} \qquad (1)$$

where V is the total Volume ($1.175 \cdot 10^{-3}$ L), $\Delta A_{510\,\mathrm{nm}}$ is the difference in absorbance of irradiated and non-irradiated (control) samples (at λ = 510 nm), l is the path length of the cuvette (10 mm), and ε is the molar attenuation coefficient of the ferrioxalate actinometer at λ = 510 nm (11100 $L \cdot mol^{-1} \cdot cm^{-1}$).[37,38] The photonflux (ϕ_q) can be calculated using equation 2,

$$\phi_q = \frac{n_{Fe(II)}}{\phi_F \cdot t \cdot f} \qquad (2)$$

where ϕ_F is the quantum yield of the ferrioxalate actinometer (1.12 at λ = 416 nm)[40] and t is the irradiation time (20 s). The fraction of light absorbed at λ = 425 nm by the actinometer (f) is calculated by using equation 3, where $A_{425\,\mathrm{nm}}$ is the absorbance of *solution 1* at λ = 425 nm.

$$f = 1 - 10^{-A_{425\,\mathrm{nm}}} \qquad (3)$$

In this case, the absorbance ($A_{425\,\mathrm{nm}}$) of *solution 1* was > 3, which indicates that > 99.9% of the photons are absorbed (f > 0.999).

After combining all equations, the photon flux was calculated to be $\phi_q = 1.17 \cdot 10^{-8}$ einsteins$\cdot s^{-1}$.

Determination of the standard reaction quantum yield – alkyl–B(pin) variant

For practical reasons, the reaction was performed in higher concentration (0.10 M) compared to the standard reaction conditions.

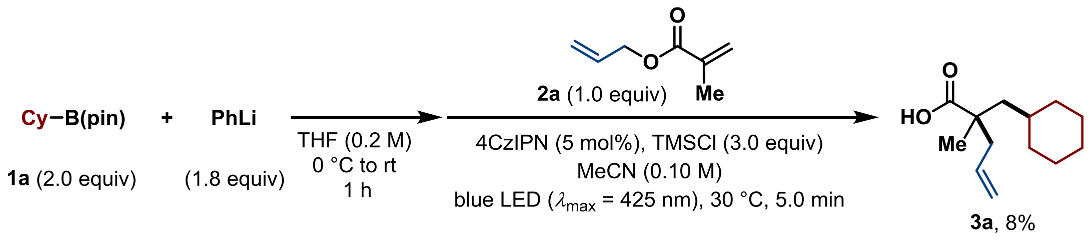

To an oven-dried 10 mL Schlenk tube equipped with a Teflon-coated magnetic stir bar were added 2-cyclohexyl-4,4,5,5-tetramethyl-1,3,2-dioxaborolane (**1a**, 42.0 mg, 0.20 mmol, 2.0 equiv), and dry THF (1.0 mL, 0.2 M) under an argon atmosphere. The solution was cooled to 0 °C, and a PhLi solution (1.9 M in dibutyl ether, 95 µL, 0.18 mmol, 1.8 equiv) was added dropwise while stirring. The mixture was stirred for 45 min at 0 °C and was then allowed to

warm up to room temperature for further 15 min. After that, the solvent was removed *in vacuo* (*via* Schlenk line), and the resulting colorless solid was dissolved in dry MeCN (1.0 mL). To this solution were added allyl methacrylate (**2a**, 13.6 µL, 0.10 mmol, 1.0 equiv), 4CzIPN (3.9 mg, 0.005 mmol, 5 mol%), and TMSCl (40 µL, 0.30 mmol, 3.0 equiv). The reaction mixture was degassed by three freeze-pump-thaw cycles and subsequently stirred and irradiated in the previously used setup (18 W, 425 nm, $\phi_q = 1.17\cdot10^{-8}$ einsteins·s^{-1}) for 5.0 min. The solvent was removed through a stream of argon, 1,3,5-trimethoxybenzene (16.8 mg, 0.10 mmol, 1.0 equiv) was added as internal standard, and the ^{1}H NMR yield was determined (the resulting suspension after addition of $CDCl_3$ was filtered over a pad of Celite® directly into the NMR tube).The yield was determined to be 8% ($n = 8\cdot10^{-6}$ mol). The quantum yield of the reaction can be calculated using equation (4),

$$\phi = \frac{n_{\text{product}}}{\phi_q \cdot t \cdot f_R} \tag{4}$$

where ϕ_q is the photon flux ($1.17\cdot10^{-8}$ einsteins·s^{-1}), t is the irradiation time (300 s). The fraction of absorbance (f_R) of the reaction was determined by measuring the absorbance of a control reaction, which has not been irradiated. The fraction of absorbance of this control reaction at λ = 425 nm gave a value > 3, which indicates that all photons were absorbed (f_R > 0.999).

The quantum yield for the reaction was calculated to be ϕ = 2.27.

The quantum yield ϕ > 1 suggests that a photo-independent radical chain process is operative in this mechanism.

Determination of the standard reaction quantum yield – *α*-silyl amine variant

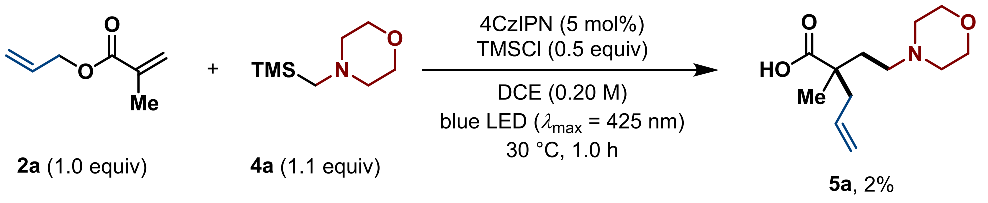

To an oven-dried 10 mL Schlenk tube equipped with a Teflon-coated magnetic stir bar were added 4CzIPN (7.9 mg, 0.01 mmol, 5 mol%) and the tube was evacuated and backfilled with argon three times. Subsequently, dry DCE (1.0 mL), 4-((trimethylsilyl)methyl)morpholine (**4a**, 43 µL, 0.22 mmol, 1.1 equiv), allyl methacrylate (**2a**, 26.8 µL, 0.20 mmol, 1.0 equiv), and

TMSCl (12.6 µL, 0.10 mmol, 0.5 equiv) were added. The reaction mixture was degassed by three freeze-pump-thaw cycles and then stirred and irradiated in the previously used setup (18 W, 425 nm, ϕ_q = 1.17·10^{-8} einsteins·s^{-1}) for 1 h. The solvent was removed through a stream of argon, 1,3,5-trimethoxybenzene (33.6 mg, 0.2 mmol, 1.0 equiv) was added as internal standard, and the ^{1}H NMR yield was determined. The yield was determined to be 2% (n = 4·10^{-6} mol). The fraction of absorbance of the control reaction was determined to be $f_R > 0.999$.

The quantum yield for the reaction was calculated to be $\phi = 0.09$.

3.5 TEMPO Trapping Experiment

Radical trapping experiments with 2,2,6,6-tetramethylpiperidinyloxyl (TEMPO, free radical) were performed for both reaction systems to obtain indications, whether radical species are involved in the reaction mechanism.

TEMPO trapping experiment – alkyl–B(pin) variant

To an oven-dried 10 mL Schlenk tube equipped with a Teflon-coated magnetic stir bar were added 2-cyclohexyl-4,4,5,5-tetramethyl-1,3,2-dioxaborolane (**1a**, 84.0 mg, 0.40 mmol, 2.0 equiv) and dry THF (2 mL) under an argon atmosphere. The reaction was cooled 0 °C and a PhLi solution (1.9 M in dibutyl ether, 0.19 mL, 0.36 mmol, 1.8 equiv) was added dropwise. The mixture was stirred for 45 min at 0 °C and was then allowed to warm up to room temperature for 15 min. Subsequently, dry MeCN (2 mL), 4CzIPN (7.9 mg, 0.01 mmol, 5 mol%), 2,2,6,6-tetramethylpiperidinyloxyl (TEMPO, 62.4 mg, 0.40 mmol, 2.0 equiv), allyl methacrylate (**2a**, 27.1 µL, 0.20 mmol, 1.0 equiv), and TMSCl (0.08 mL, 0.6 mmol, 3 equiv) were added. The reaction mixture was degassed by three freeze-pump-thaw cycles and subsequently stirred and irradiated with blue LEDs (30 W, λ_{max} = 450 nm) for 20 h. The crude reaction mixture was analyzed by GC-MS and ESI-MS. Then, the solvent was removed through a stream of argon, 1,3,5-trimethoxybenzene (33.6 mg, 0.20 mmol, 1.0 equiv) was added as internal standard, and the ^{1}H NMR yield was determined (the resulting suspension after addition of $CDCl_3$ was filtered over a pad of Celite® directly into the NMR tube).

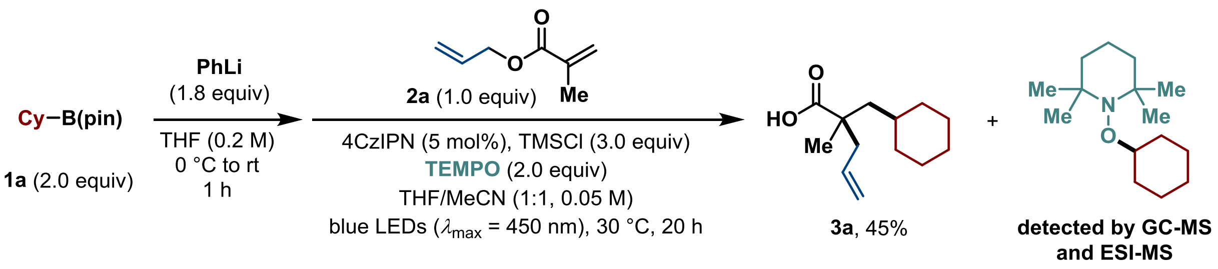

The product yield was decreased to 45% ^{1}H NMR yield and the expected TEMPO radical adduct was detected by GC-MS and ESI-MS. The decreased yield and the presence of the TEMPO radical adduct, suggest a radical reaction pathway. However, complete inhibition of the reaction would be expected as with the α-silyl amine variant, indicating that these two reaction systems proceed *via* different mechanisms.

TEMPO trapping experiment – α-silyl amine variant

To an oven-dried 10 mL Schlenk tube equipped with a Teflon-coated magnetic stir bar were added 4CzIPN (7.9 mg, 0.01 mmol, 5 mol%) and 2,2,6,6-tetramethylpiperidinyloxyl (TEMPO, 34.3 mg, 0.22 mmol, 1.1 equiv). The tube was evacuated and backfilled with argon three times. Subsequently, dry DCE (1.0 mL), 4-((trimethylsilyl)methyl)morpholine (**4a**, 38.1 mg, 0.22 mmol, 1.1 equiv), allyl methacrylate (**2a**, 27.1 µL, 0.20 mmol, 1.0 equiv), and TMSCl (13 µL, 0.10 mmol, 0.5 equiv) were added. The reaction mixture was degassed by three freeze-pump-thaw cycles, and subsequently stirred and irradiated with blue LEDs (30 W, λ_{max} = 450 nm) for 14 h. The crude reaction mixture was analyzed by GC-MS and ESI-MS. Then, the solvent was removed through a stream of argon, 1,3,5-trimethoxybenzene (33.6 mg, 0.20 mmol, 1.0 equiv) was added as internal standard, and the ^{1}H NMR yield was determined.

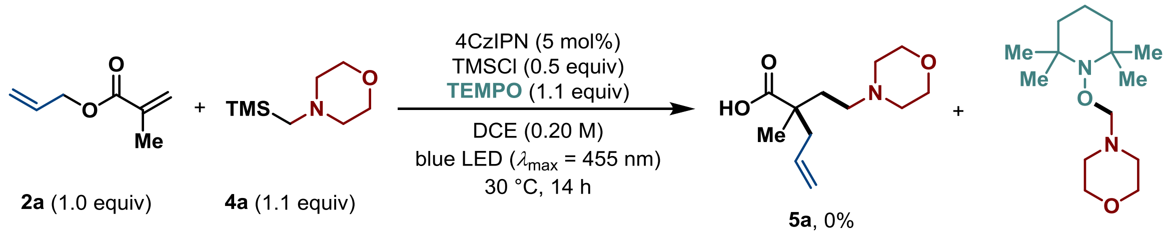

2a (1.0 equiv) **4a** (1.1 equiv) **5a**, 0%

<u>not</u>
detected by GC-MS or by ESI-MS

The reaction was completely inhibited, but the expected TEMPO radical adduct was not detected by GC-MS or ESI-MS. However, the complete inhibition of the reaction hints towards a radical reaction pathway.

3.6 Decomposition Experiment: Primary *vs.* Secondary Alkylboronates

To understand the limitation (low yielding) of primary alkylboronic acid pinacol esters in this protocol, we examined the decomposition of a primary and secondary alkylboronate complex upon treatment with TMSCl in relation to each other. The formation of 2,3-dimethyl-3-((trimethylsilyl)oxy)butan-2-ol (**SII**) under the reaction conditions is utilized as an indicator for the progress of the decomposition of the alkylboronate complexes.

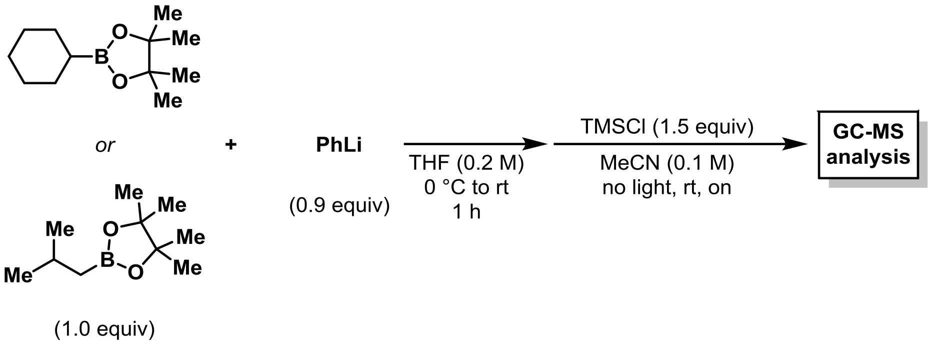

To an oven-dried 10 mL Schlenk tube equipped with a Teflon-coated magnetic stir bar were added 2-cyclohexyl-4,4,5,5-tetramethyl-1,3,2-dioxaborolane (**1a**, 84.0 mg, 0.40 mmol, 1.0 equiv) or 2-isobutyl-4,4,5,5-tetramethyl-1,3,2-dioxaborolane (73.6 mg, 0.40 mmol, 1.0 equiv), and dry THF (2.0 mL) under an argon atmosphere. The solution was cooled to 0 °C and a PhLi solution (1.9 M in dibutyl ether, 0.19 mL, 0.36 mmol, 0.9 equiv) was added dropwise while stirring. The mixture was stirred for 45 min at 0 °C and was then allowed to warm up to room temperature for further 15 min. After that, the solvent was removed *in vacuo* (*via* Schlenk line), and the resulting colorless solid was dissolved in dry MeCN (4.0 mL). To this solution was added TMSCl (80 µL, 0.60 mmol, 1.5 equiv), and the mixture was stirred in the absence of light at room temperature overnight. Mesitylene (28 µL, 0.20 mmol) was added as internal standard and GC-MS was measured.

As can be seen form figure S16, the relative formation of **SII** in case of the primary alkylboronate complex is much higher (~3.3 times) compared to the secondary alkylboronate complex. This result indicates a faster decomposition of the primary alkylboronate complex under the reaction conditions.

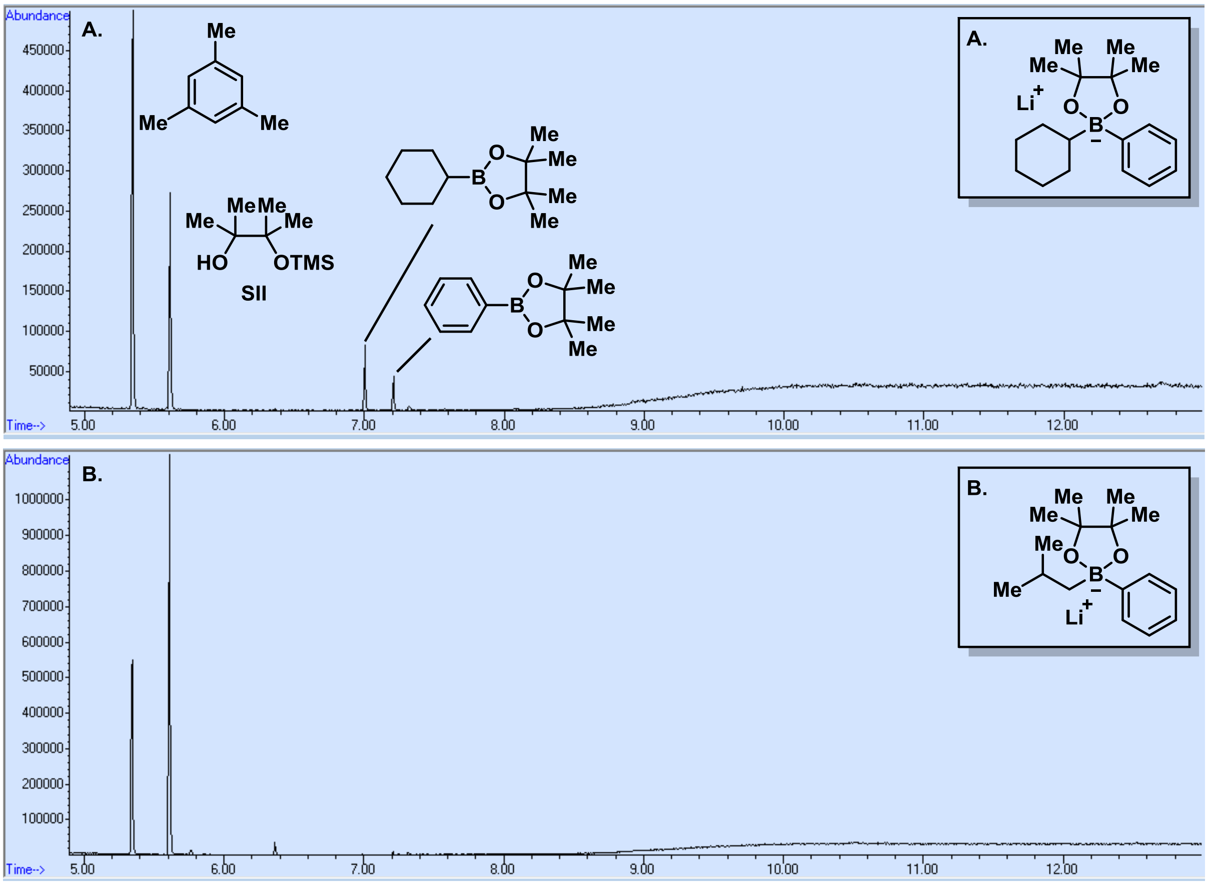

Figure S16. Comparison of relative 2,3-dimethyl-3-((trimethylsilyl)oxy)butan-2-ol (**SII**) formation as indicator for the decomposition of alkylboronate complexes upon TMSCl treatment. **A.** GC-MS results with a secondary alkylboronate complex. **B.** GC-MS results with a primary alkylboronate complex.

4 References

1 J. Luo and J. Zhang, *ACS Catal.*, 2016, **6**, 873–877.

2 D. Hanss, J. C. Freys, G. Bernardinelli and O. S. Wenger, *Eur. J. Inorg. Chem.*, 2009, 4850–4859.

3 J. D. Slinker, A. A. Gorodetsky, M. S. Lowry, J. Wang, S. Parker, R. Rohl, S. Bernhard and G. G. Malliaras, *J. Am. Chem. Soc.*, 2004, **126**, 2763–2767.

4 G. Casoni, E. Myers and V. K. Aggarwal, *Synthesis*, 2016, **48**, 3241–3253.

5 T. C. Atack, R. M. Lecker and S. P. Cook, *J. Am. Chem. Soc.*, 2014, **136**, 9521–9523.

6 F. Sandfort, F. Strieth-Kalthoff, F. J. R. Klauck, M. J. James and F. Glorius, *Chem. - Eur. J.*, 2018, **24**, 17210–17214.

7 M. Wollenburg, D. Moock and F. Glorius, *Angew. Chem., Int. Ed.*, 2019, **58**, 6549–6553.

8 J. Wang, M. Shang, H. Lundberg, K. S. Feu, S. J. Hecker, T. Qin, D. G. Blackmond and P. S. Baran, *ACS Catal.*, 2018, **8**, 9537–9542.

9 A. Fawcett, J. Pradeilles, Y. Wang, T. Mutsuga, E. L. Myers and V. K. Aggarwal, *Science*, 2017, **357**, 283–286.

10 C. Shu, A. Noble and V. K. Aggarwal, *Angew. Chem., Int. Ed.*, 2019, **58**, 3870–3874.

11 L. Candish, M. Teders and F. Glorius, *J. Am. Chem. Soc.*, 2017, **139**, 7440–7443.

12 J. W. Clary, T. J. Rettenmaier, R. Snelling, W. Bryks, J. Banwell, W. T. Wipke and B. Singaram, *J. Org. Chem.*, 2011, **76**, 9602–9610.

13 U. Blaschke, K. Eismann, A. Böhme, A. Paschke and G. Schüürmann, *Chem. Res. Toxicol.*, 2012, **25**, 170–180.

14 M. Sova, A. Perdih, M. Kotnik, K. Kristan, T. L. Rižner, T. Solmajer and S. Gobec, *Bioorg. Med. Chem.*, 2006, **14**, 7404–7418.

15 T. Miura, Y. Mikano and M. Murakami, *Org. Lett.*, 2011, **13**, 3560–3563.

16 W. Kong, Q. Wang and J. Zhu, *Angew. Chem., Int. Ed.*, 2017, **56**, 3987–3991.

17 M. Koy, P. Bellotti, F. Katzenburg, C. G. Daniliuc and F. Glorius, *Angew. Chem., Int.*

Ed., 2020, **59**, 2375–2379.

18 A. Srikrishna and P. Praveen Kumar, *Tetrahedron*, 2000, **56**, 8189–8195.

19 J. Li and T. L. Lowary, *Org. Lett.*, 2008, **10**, 881–884.

20 R. Riclea, J. Gleitzmann, H. Bruns, C. Junker, B. Schulz and J. S. Dickschat, *Beilstein J. Org. Chem.*, 2012, **8**, 941–950.

21 J. R. Sanzone and K. A. Woerpel, *Angew.Chem., Int. Ed.*, 2016, **55**,790 –793.

22 T. J. Cogswell, C. S. Donald, D.-L. Long and R. Marquez, *Org. Biomol. Chem.*, 2015, **13**, 717–728.

23 Z. Gonda, F. Béke, O. Tischler, M. Petró, Z. Novák and B. L. Tóth, *Eur. J. Org. Chem.*, 2017, 2112–2117.

24 J. H. Reed, P. A. Donets, S. Miaskiewicz and N. Cramer, *Angew. Chem., Int. Ed.*, 2019, **58**, 8893–8897.

25 B. Jiang, M. Zhao, S.-S. Li, Y.-H. Xu and T.-P. Loh, *Angew. Chem., Int. Ed.*, 2018, **57**, 555–559.

26 C. Feng and T.-P. Loh, *Angew. Chem., Int. Ed.*, 2013, **52**, 12414–12417.

27 X. Wang and S. L. Buchwald, *J. Am. Chem. Soc.*, 2011, **133**, 19080–19083.

28 J. Lauer-Fields, K. Brew, J. K. Whitehead, S. Li, R. P. Hammer and G. B. Fields, *J. Am. Chem. Soc.*, 2007, **129**, 10408–10417.

29 A. C. Serra, C. M. M. da Silva Corrêa and M. L. C. do Vale, *Tetrahedron*, 1991, **47**, 9463–9488.

30 J. L. Schwarz, R. Kleinmans, T. O. Paulisch and F. Glorius, *J. Am. Chem. Soc.*, 2020, **142**, 2168–2174.

31 C. Wang, Y. Zheng, H. Huo, P. Röse, L. Zhang, K. Harms, G. Hilt and E. Meggers, *Chem. - Eur. J.*, 2015, **21**, 7355–7359.

32 S. Okazaki and Y. Sato, *Synthesis*, 1990, 36–38.

33 J. Li and Y. Sha, *Molecules*, 2008, **13**, 1111–1119.

34 T. Gensch, M. Teders and F. Glorius, *J. Org. Chem.*, 2017, **82**, 9154–9159.

35 K. D. Collins and F. Glorius, *Nat. Chem.*, 2013, **5**, 597–601.

36 L. Pitzer, F. Schäfers and F. Glorius, *Angew. Chem., Int. Ed.*, 2019, **58**, 8572–8576.

37 M. A. Cismesia and T. P. Yoon, *Chem. Sci.*, 2015, **6**, 5426–5434.

38 H. Heath, *Proc. R. Soc. London. Ser. A. Math. Phys. Sci.*, 1956, **235**, 518–536.

39 I. P. Pozdnyakov, O. V. Kel, V. F. Plyusnin, V. P. Grivin and N. M. Bazhin, *J. Phys. Chem. A*, 2008, **112**, 8316–8322.

40 E. E. Wegner and A. W. Adamson, *J. Am. Chem. Soc.*, 1966, **88**, 394–404.

5 Spectra

S1h, ^{1}H NMR (400 MHz, $CDCl_3$)

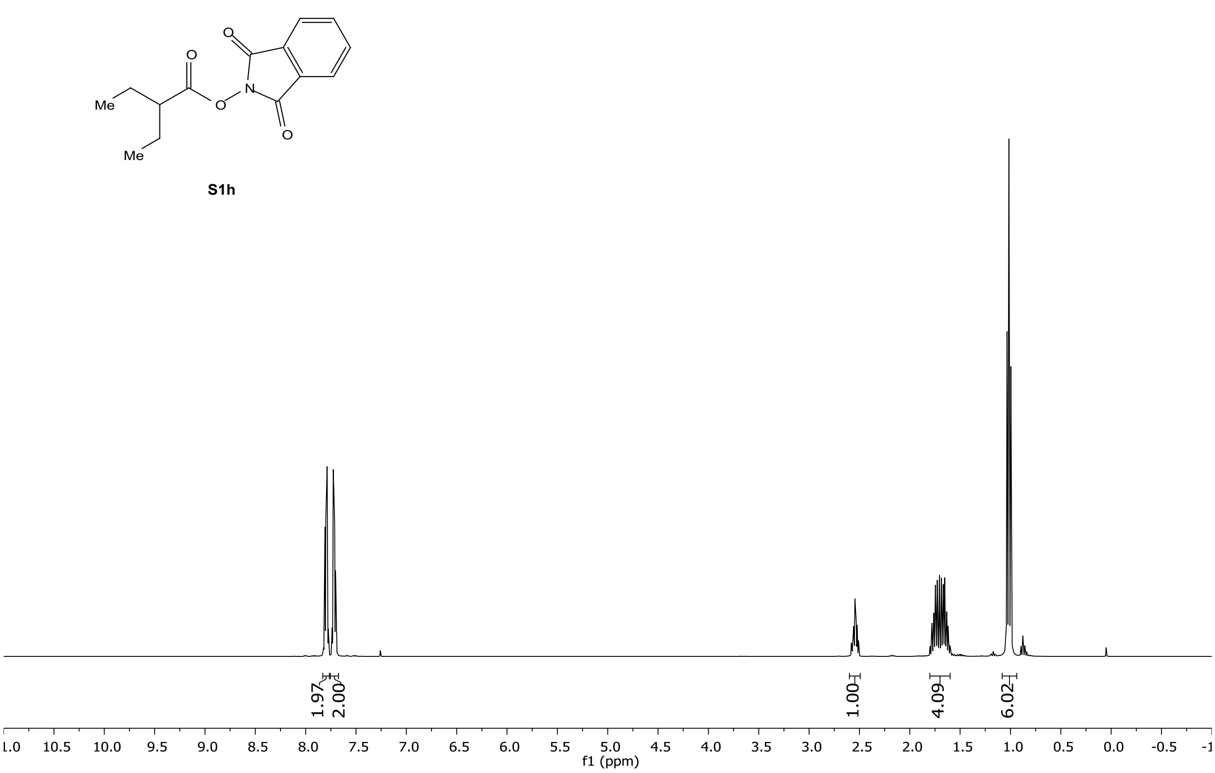

S1h, ^{13}C NMR (101 MHz, $CDCl_3$)

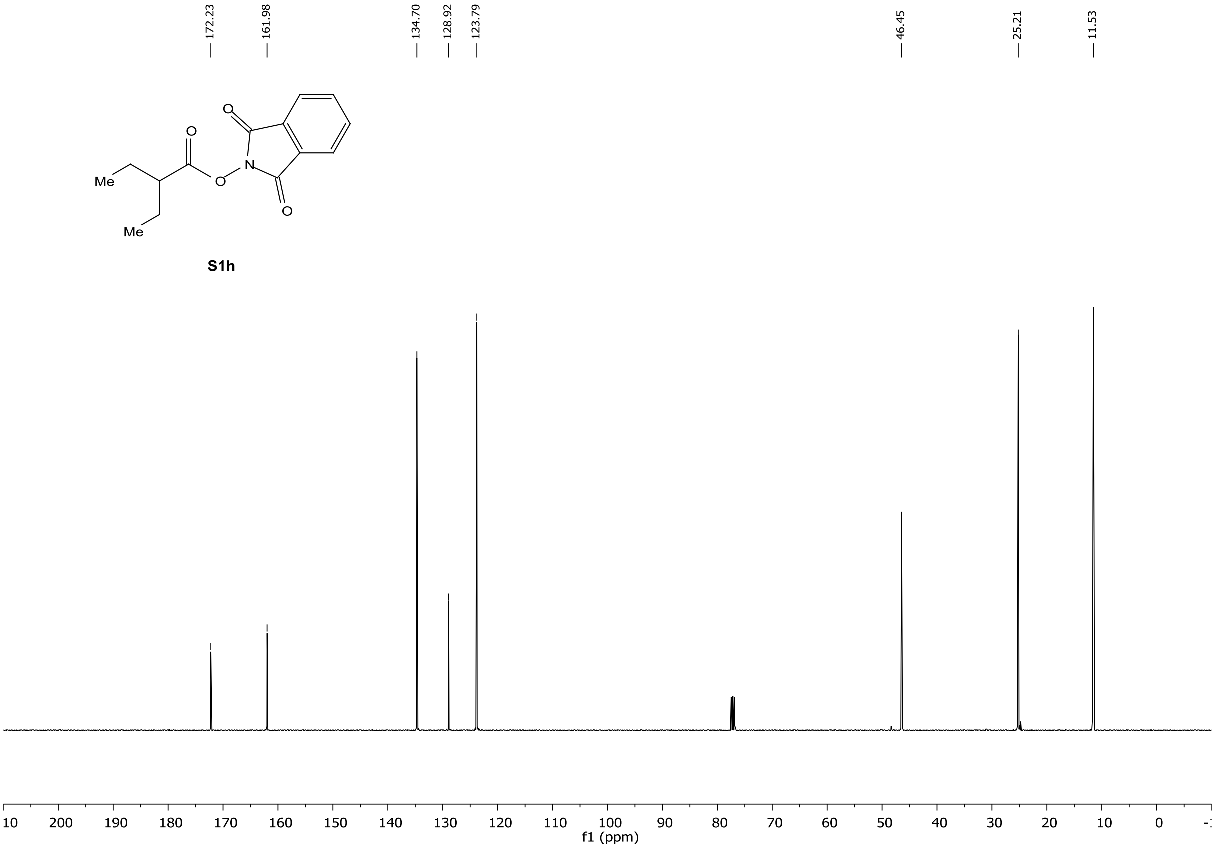

1h, ^{1}H NMR (400 MHz, $CDCl_3$)

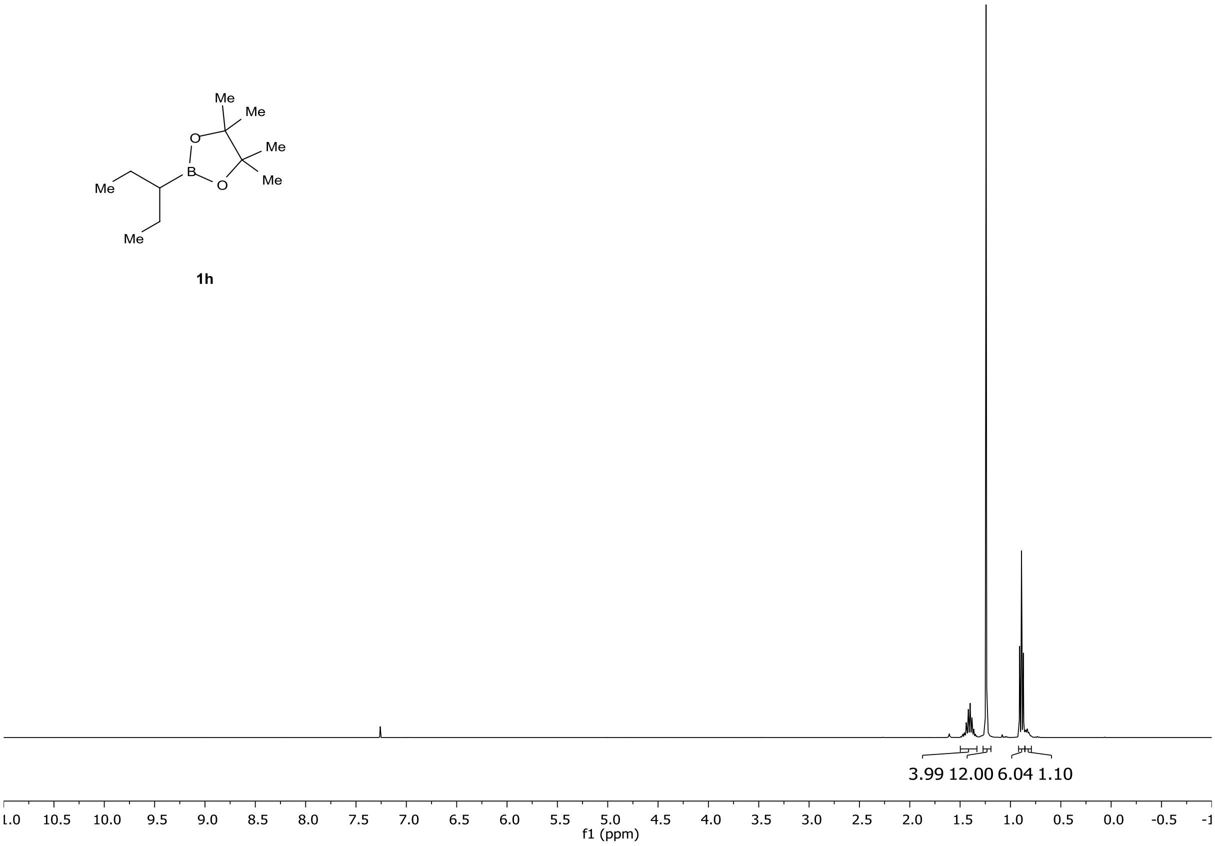

1h, ^{13}C NMR (101 MHz, $CDCl_3$)

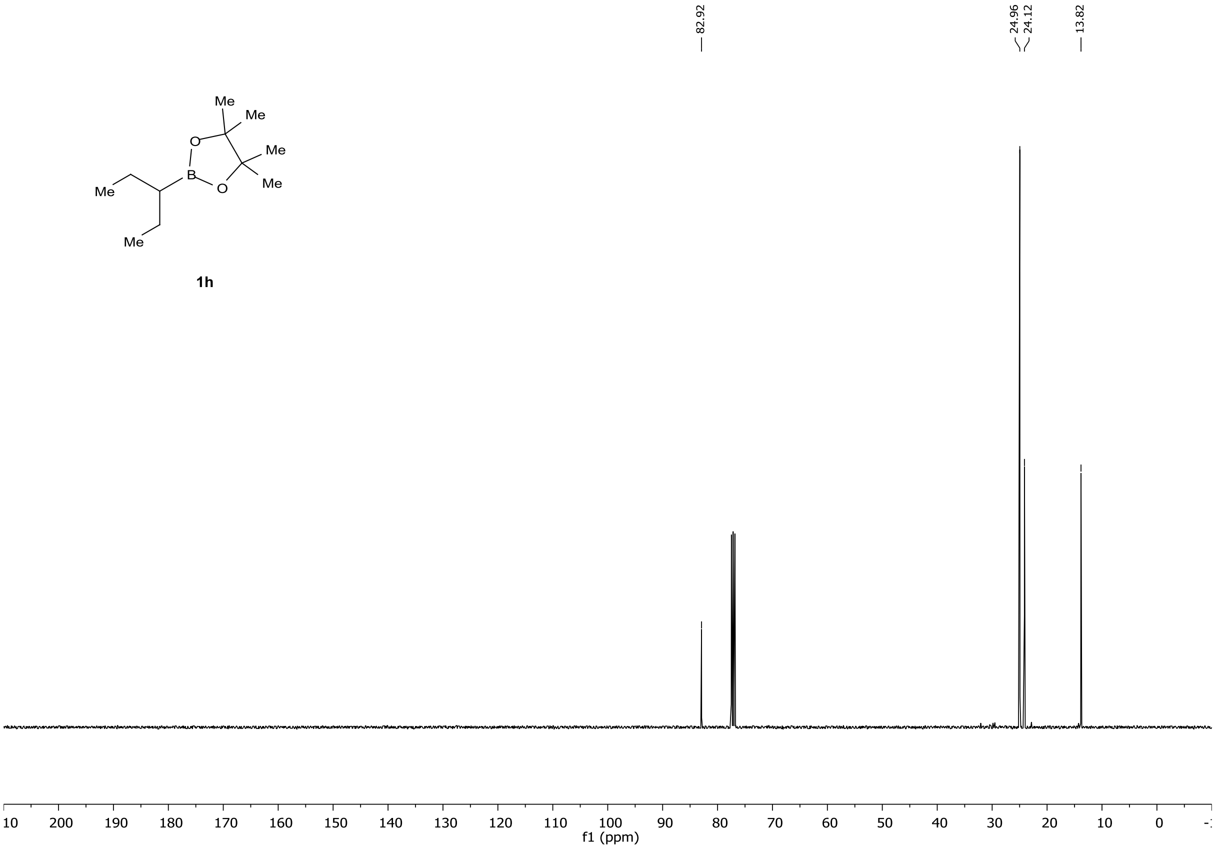

1h, ^{11}B NMR (128 MHz, $CDCl_3$)

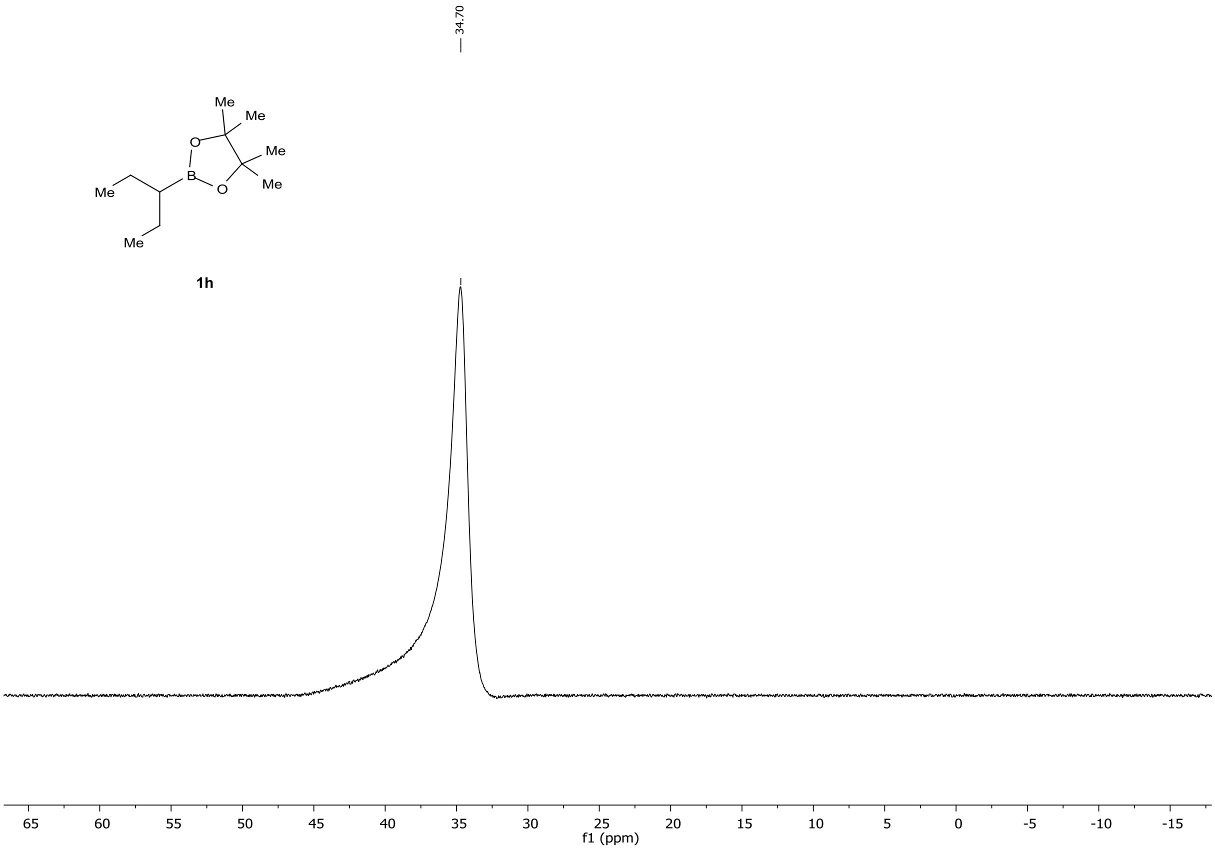

2b, ^{1}H NMR (400 MHz, $CDCl_3$)

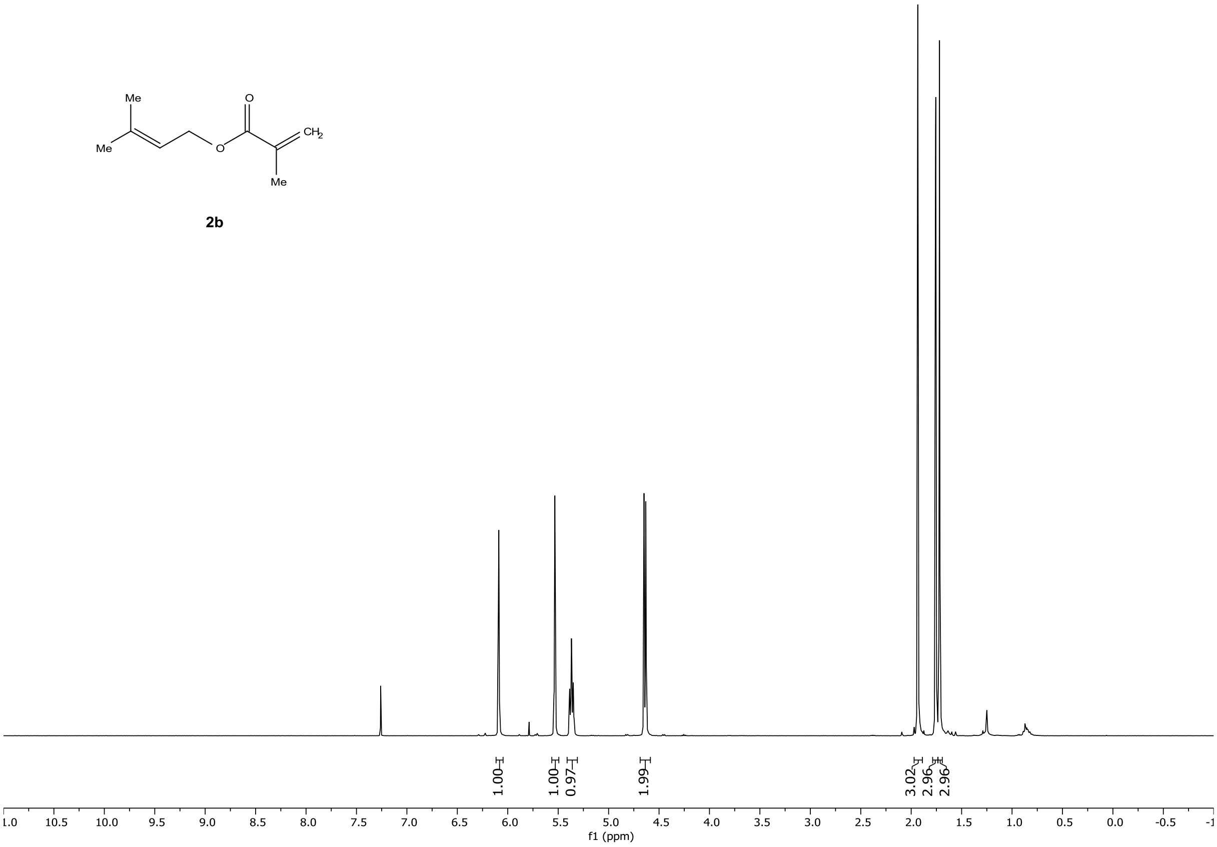

2b, ^{13}C NMR (101 MHz, $CDCl_3$)

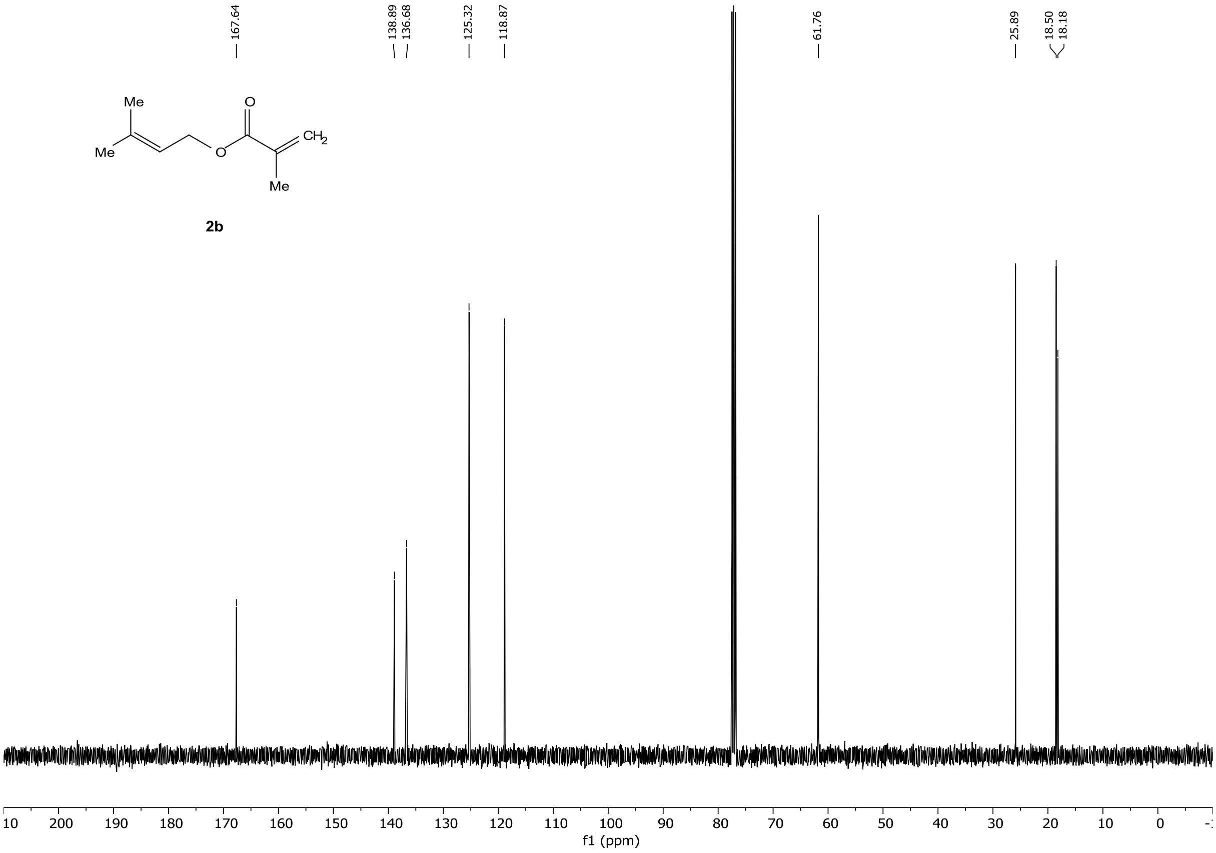

2c, ^{1}H NMR (400 MHz, $CDCl_3$)

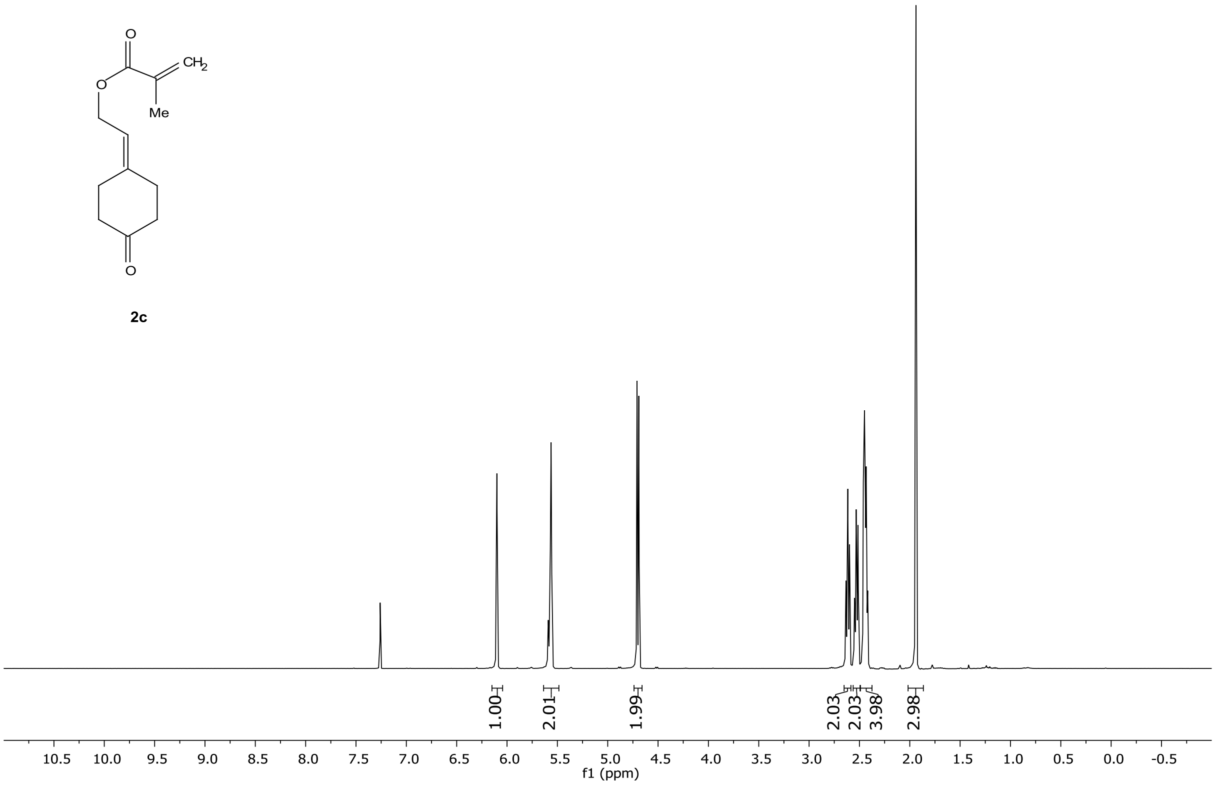

2c, ^{13}C NMR (101 MHz, $CDCl_3$)

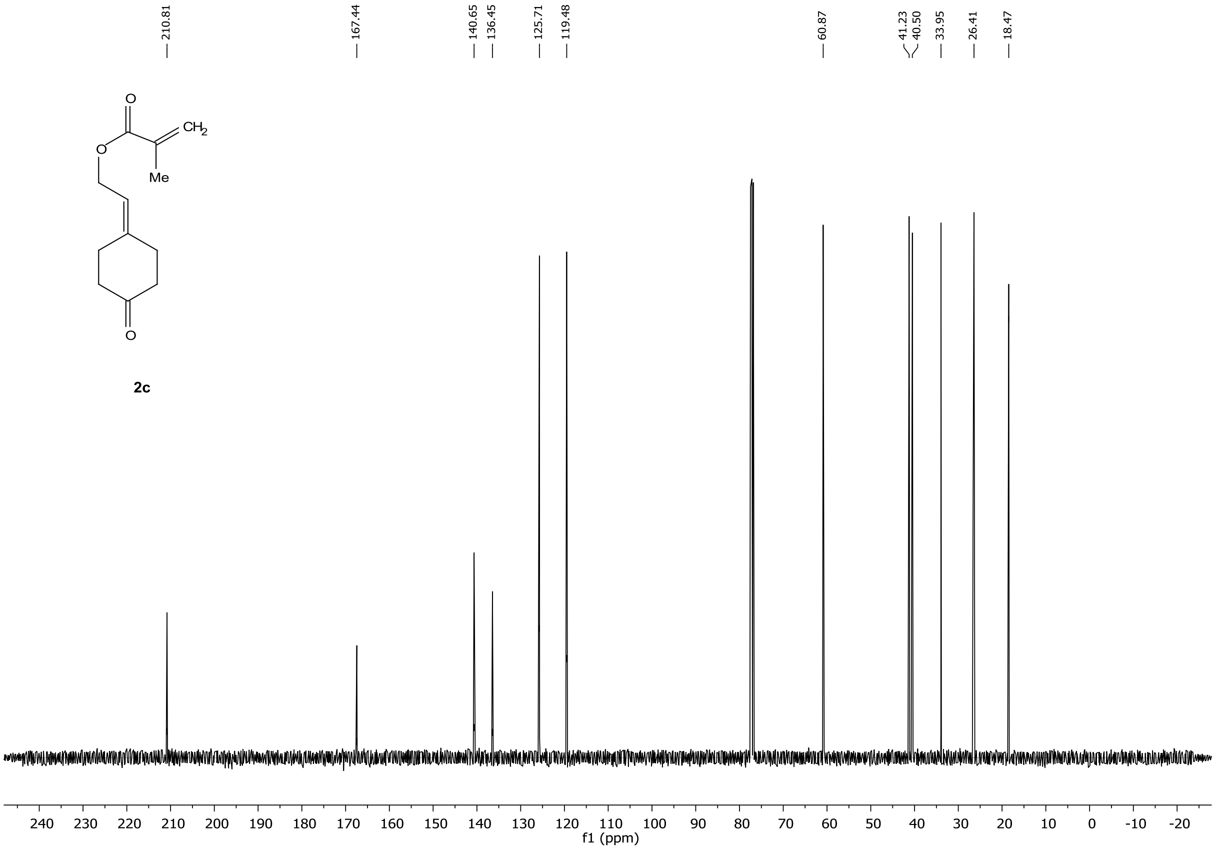

2d, ^{1}H NMR (400 MHz, $CDCl_3$)

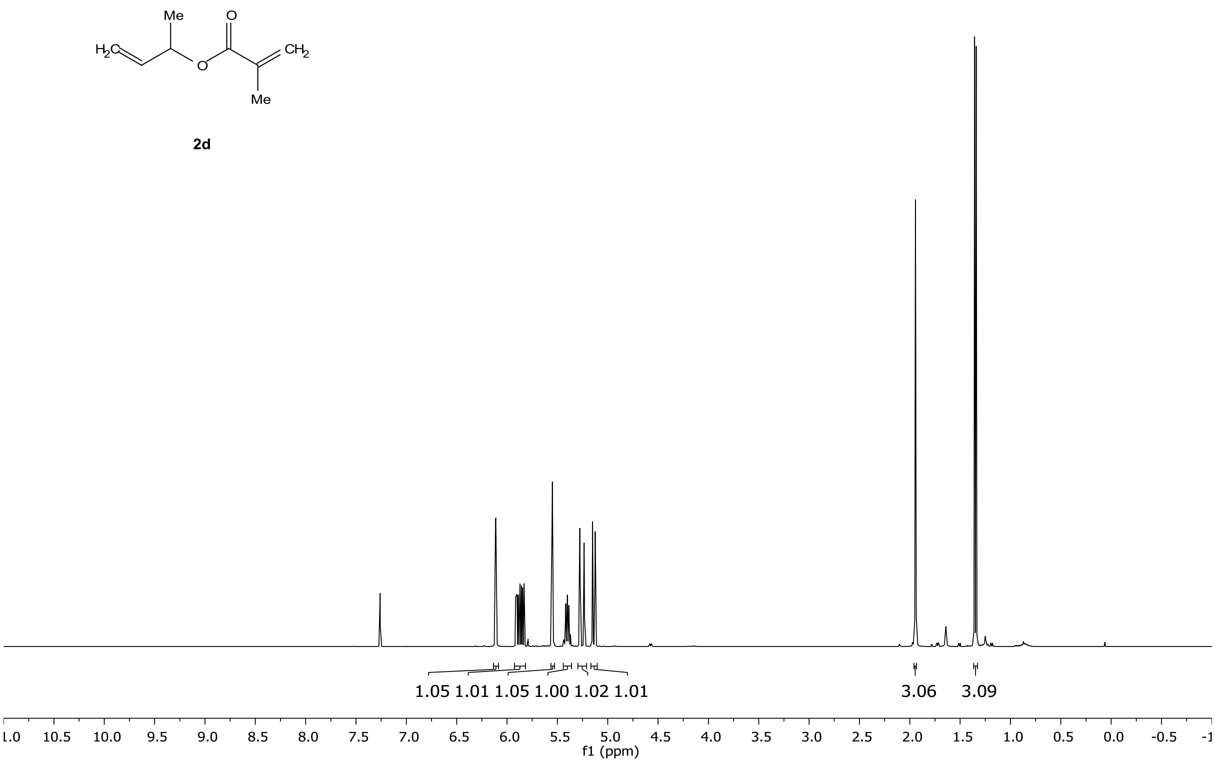

2d, ^{13}C NMR (101 MHz, $CDCl_3$)

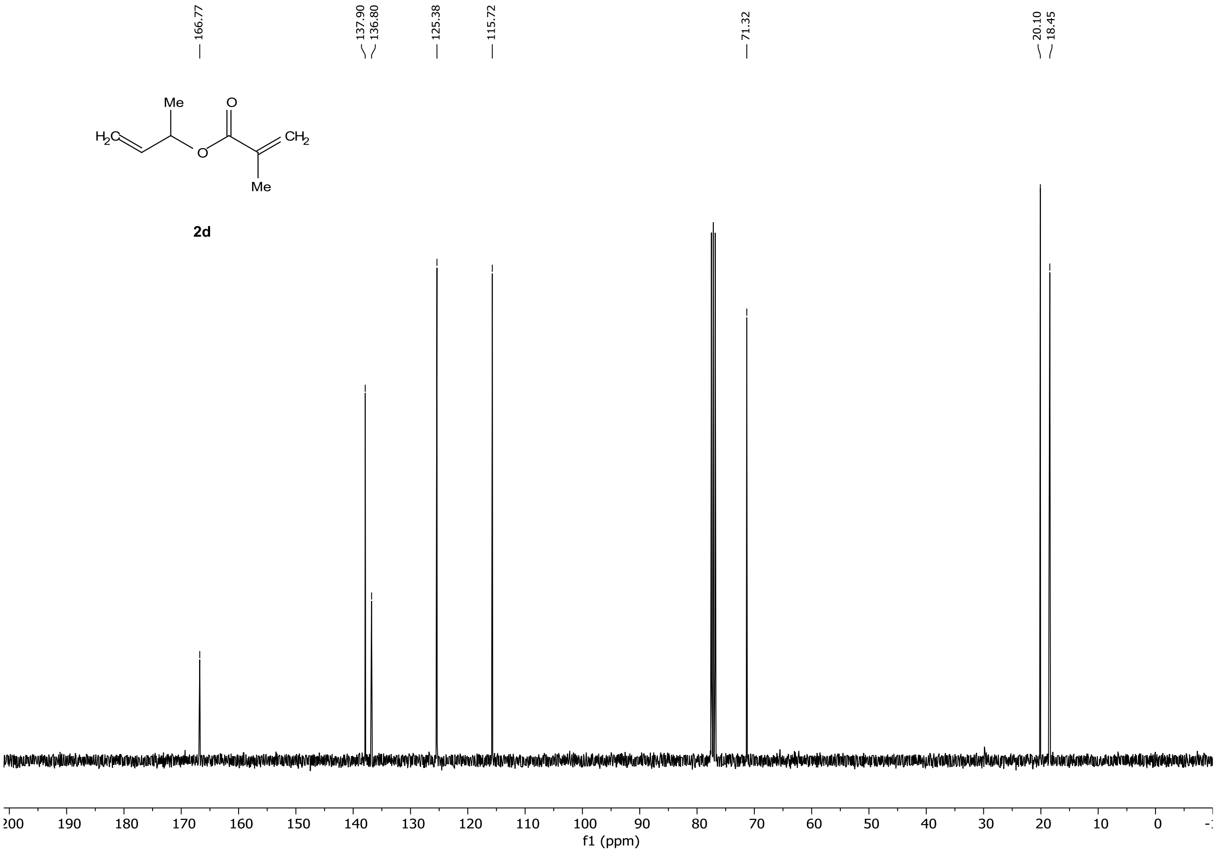

2e, ^{1}H NMR (400 MHz, $CDCl_3$)

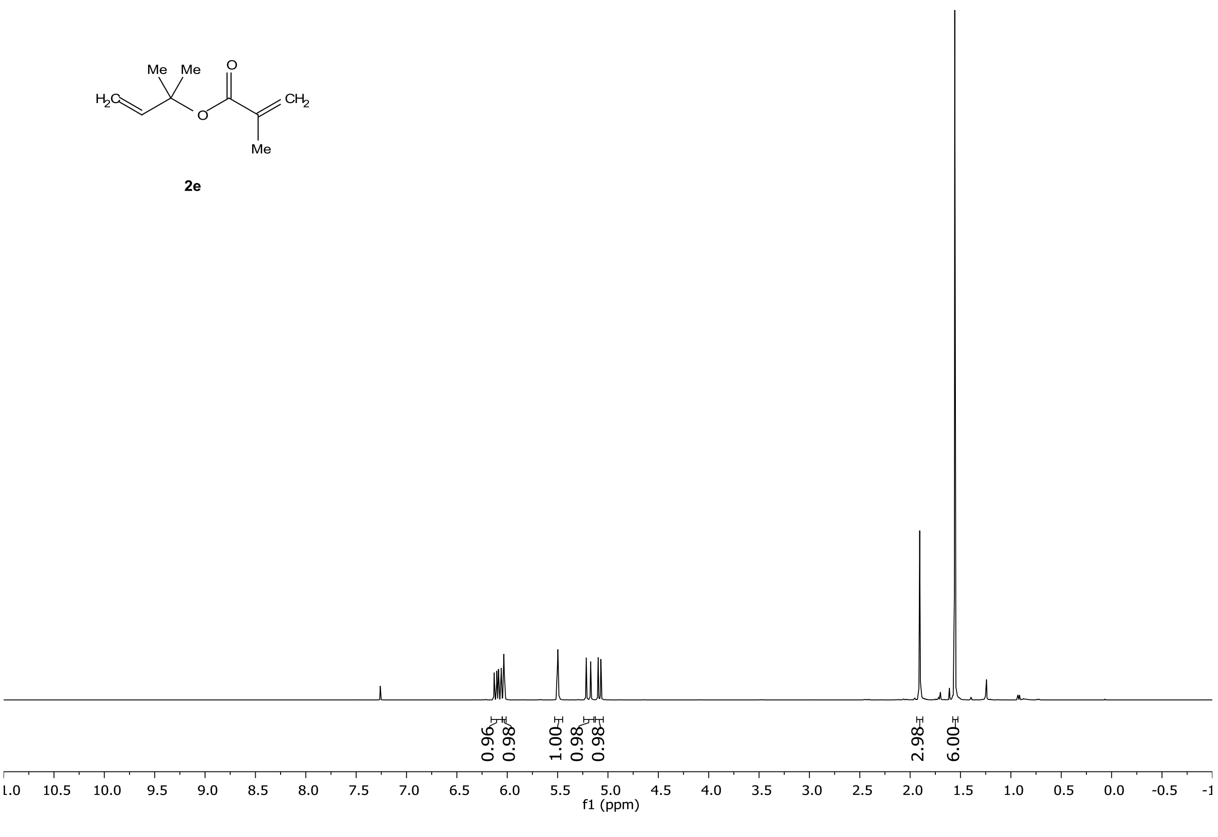

2e, ^{13}C NMR (101 MHz, $CDCl_3$)

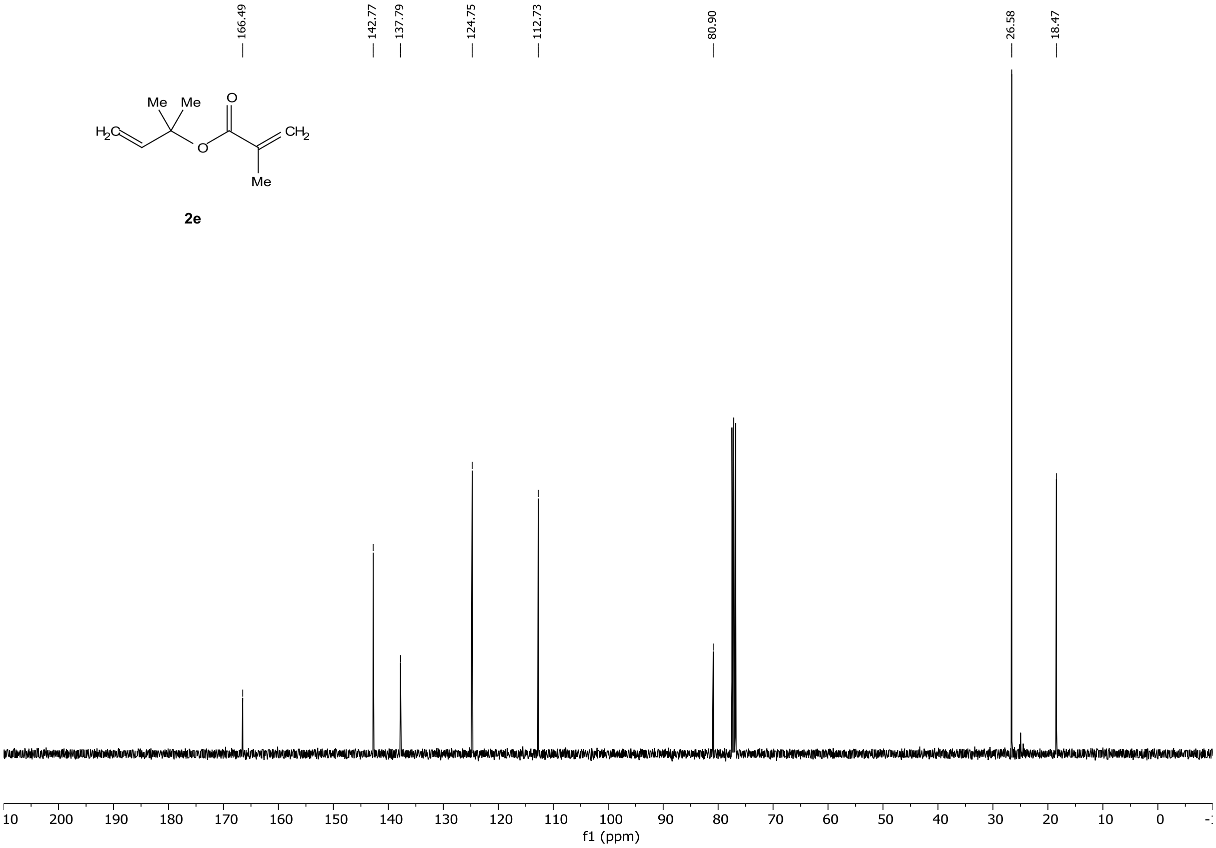

S2f, ^{1}H NMR (400 MHz, $CDCl_3$)

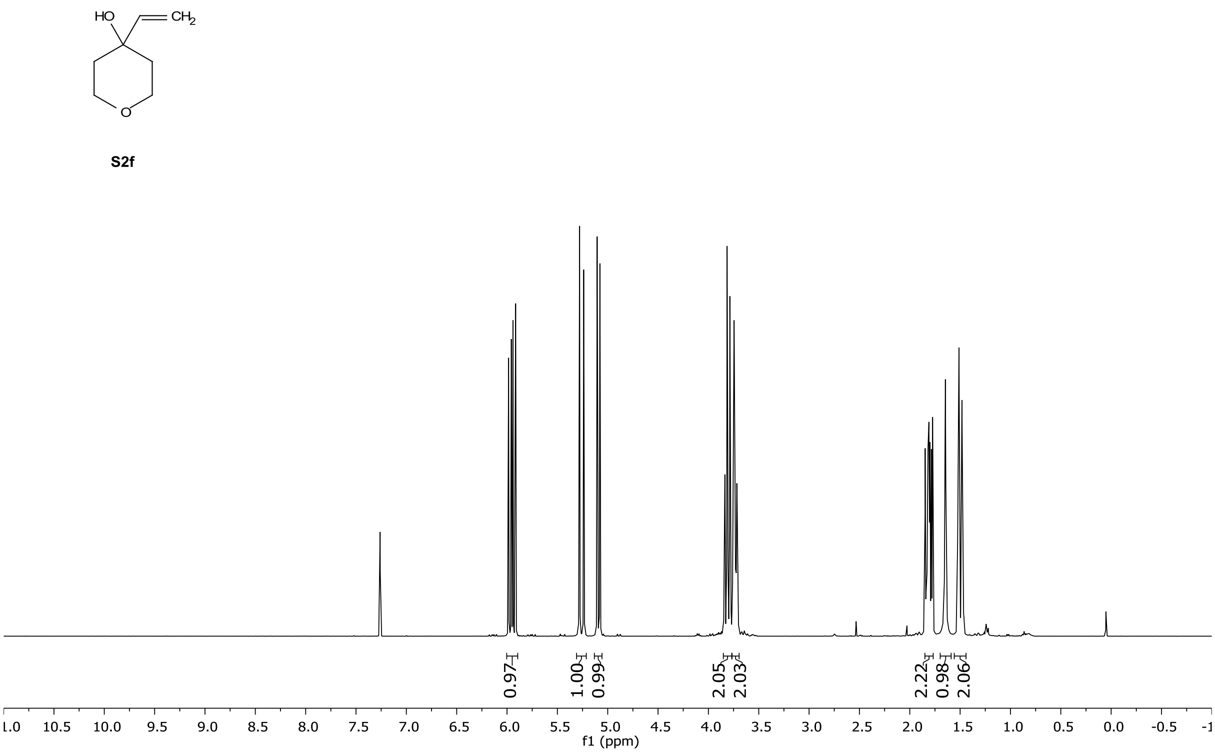

S2f, ^{13}C NMR (101 MHz, $CDCl_3$)

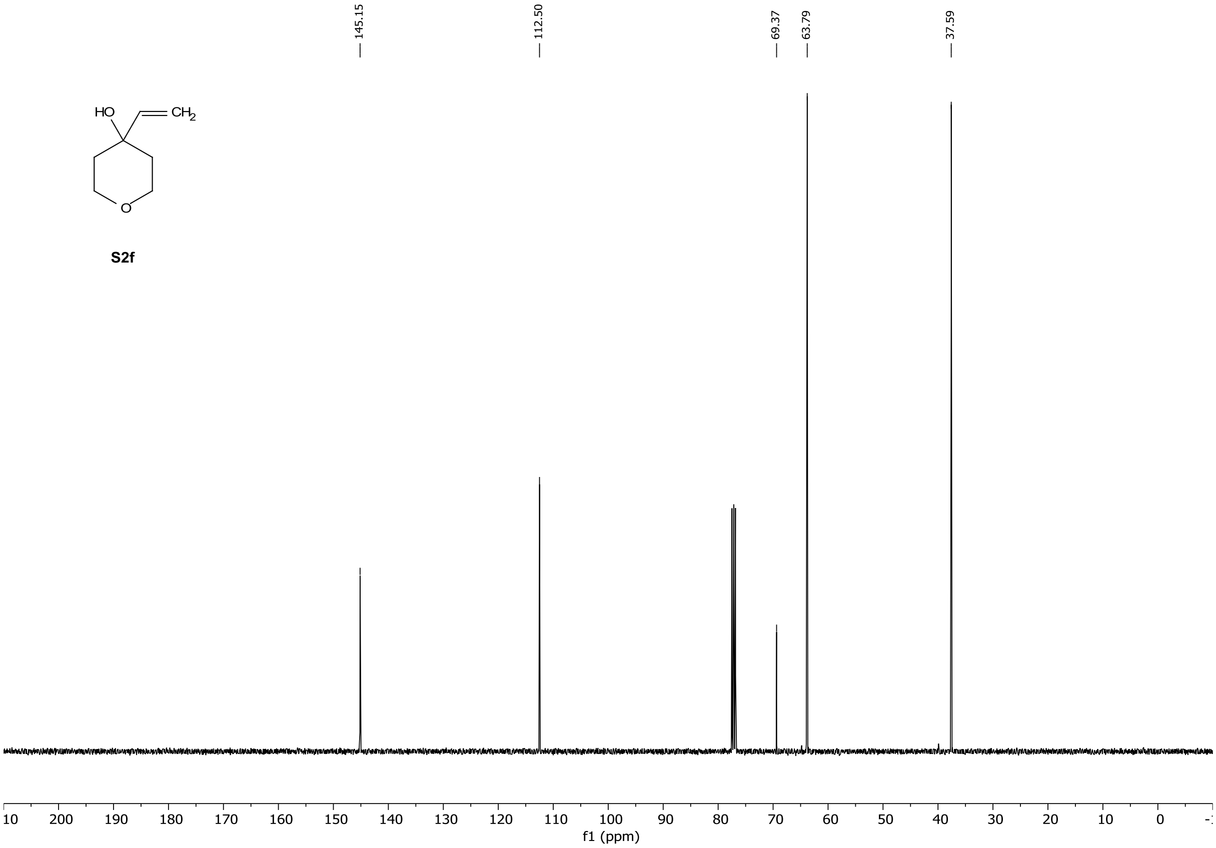

2f, ^{1}H NMR (400 MHz, $CDCl_3$)

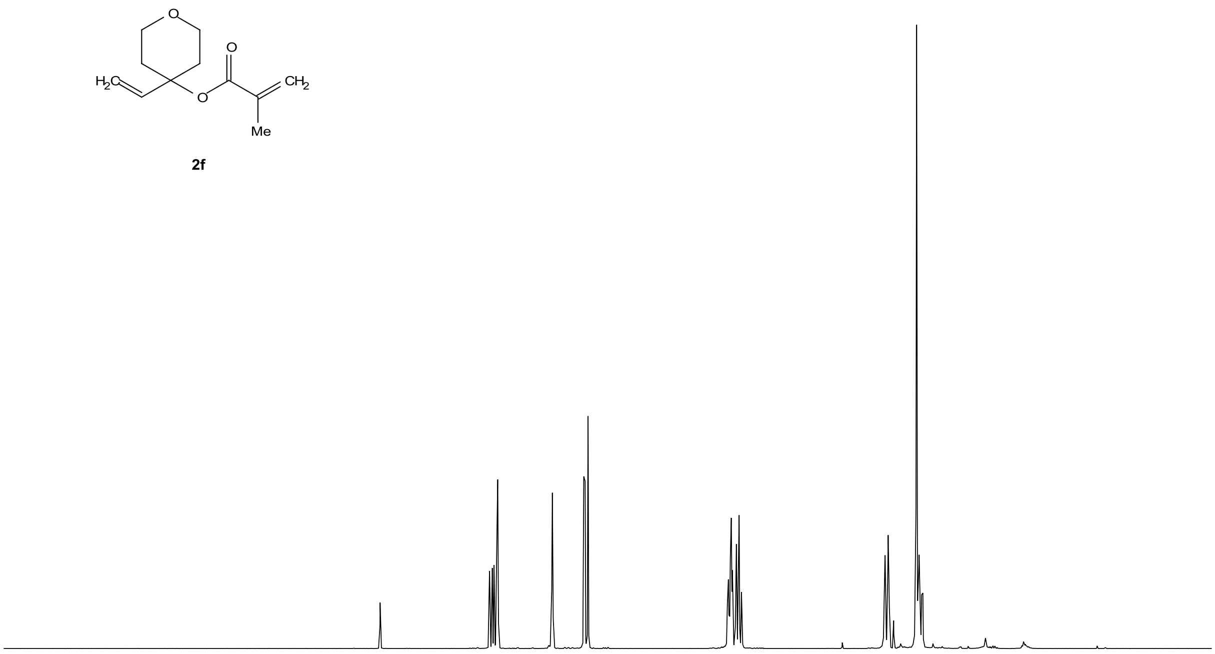

2f, ^{13}C NMR (101 MHz, $CDCl_3$)

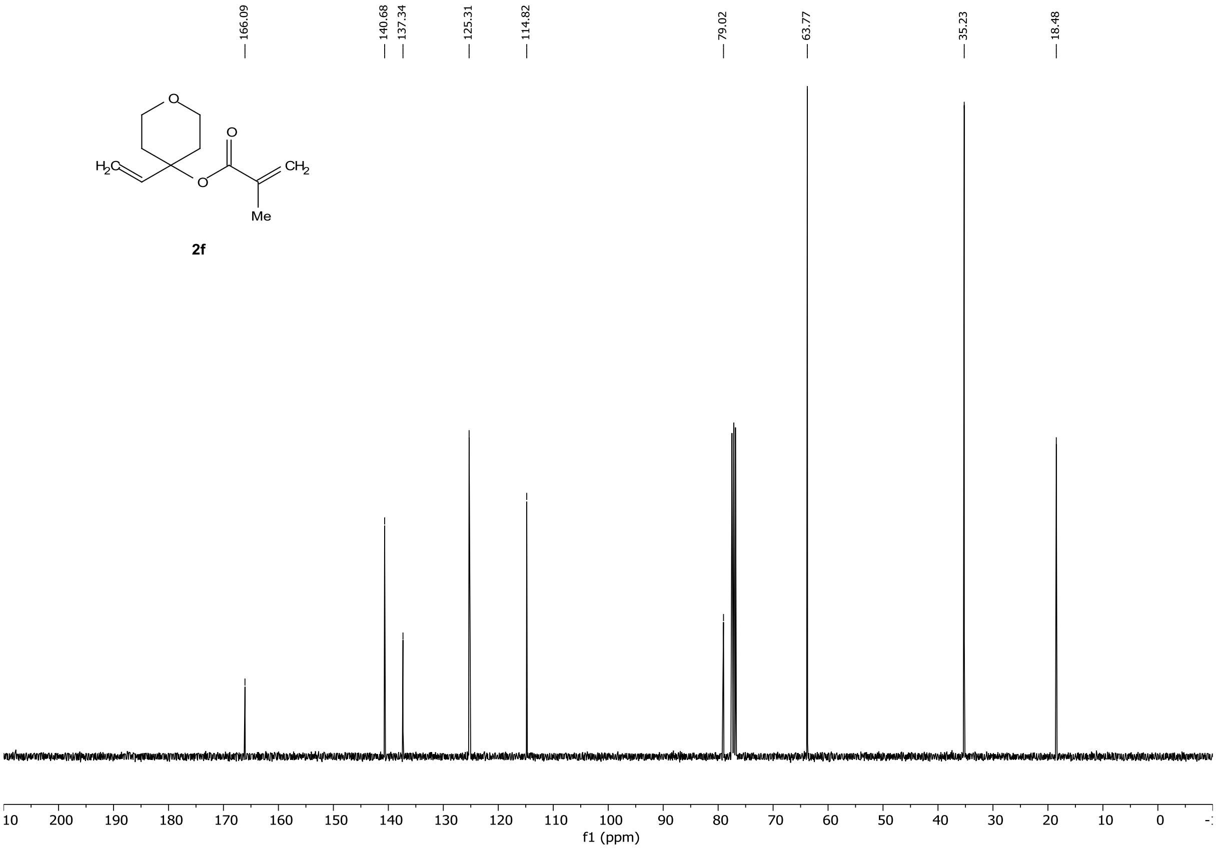

2g, ^{1}H NMR (400 MHz, $CDCl_3$)

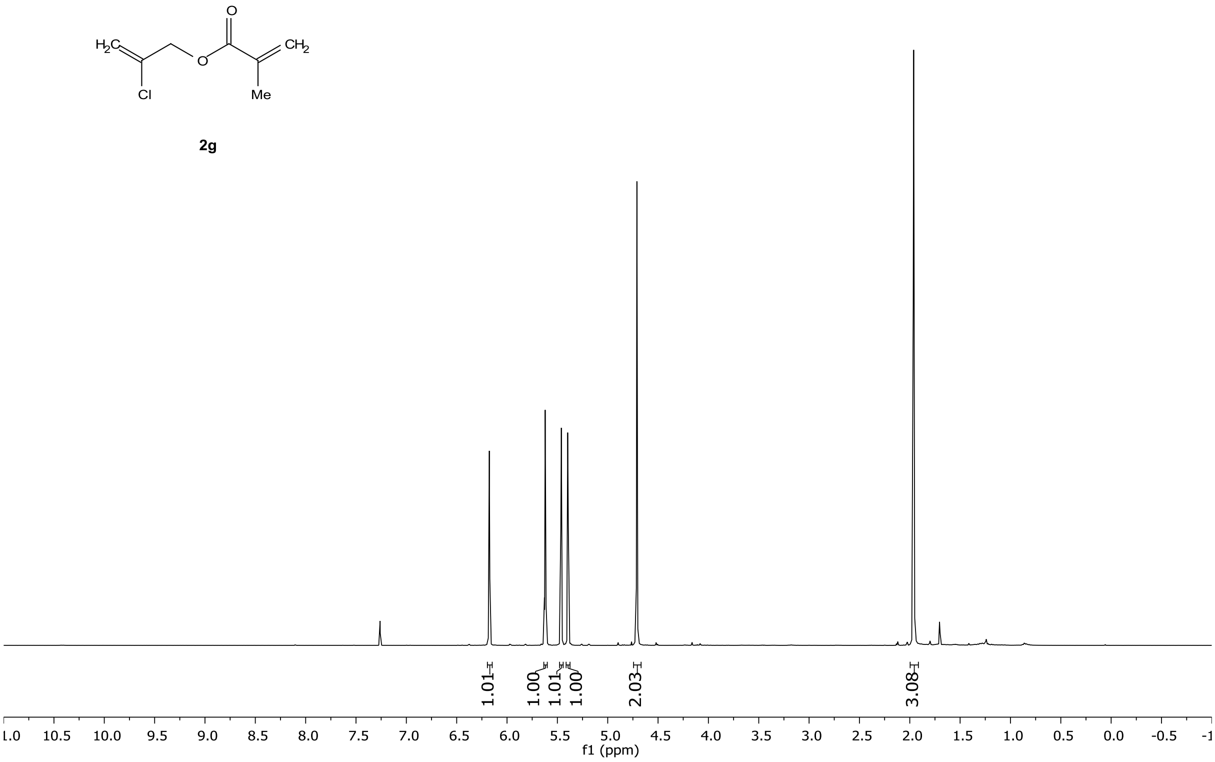

2g, ^{13}C NMR (101 MHz, $CDCl_3$)

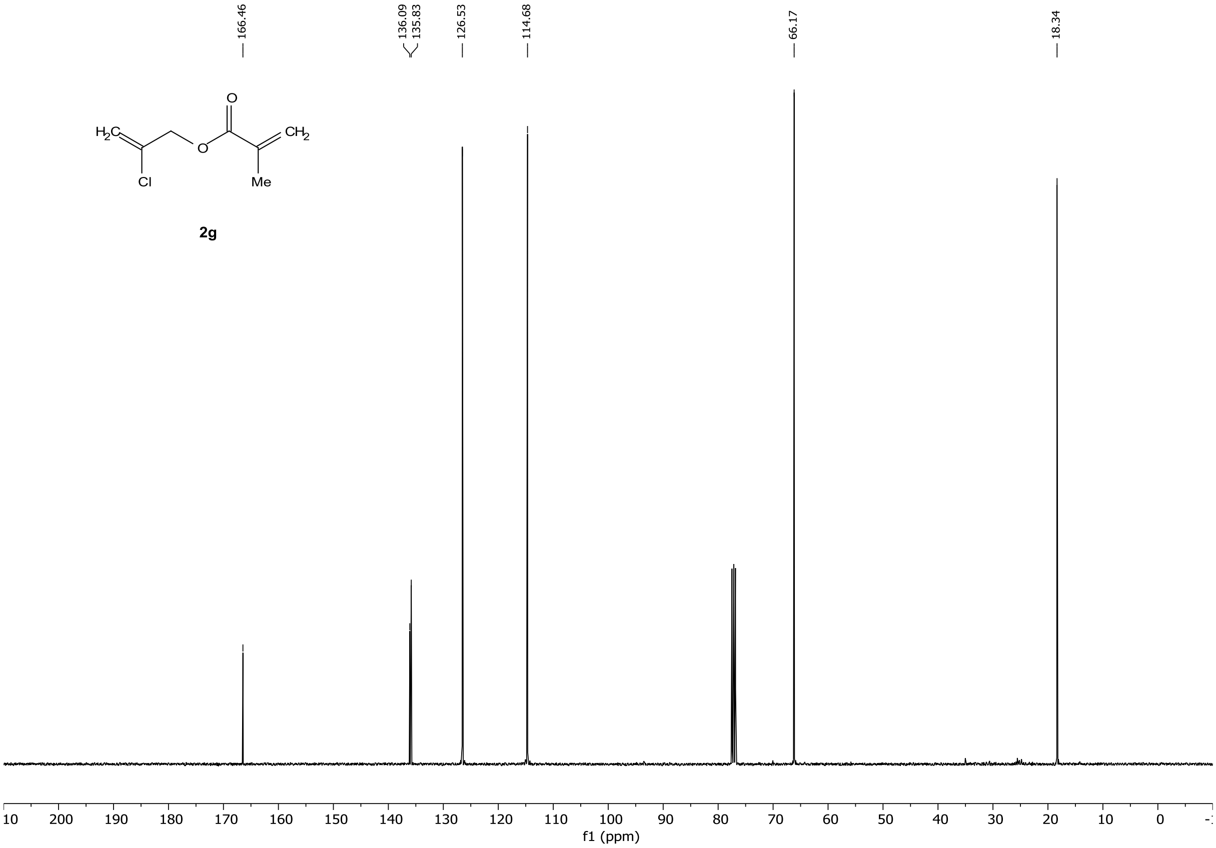

2h, ^{1}H NMR (400 MHz, $CDCl_3$)

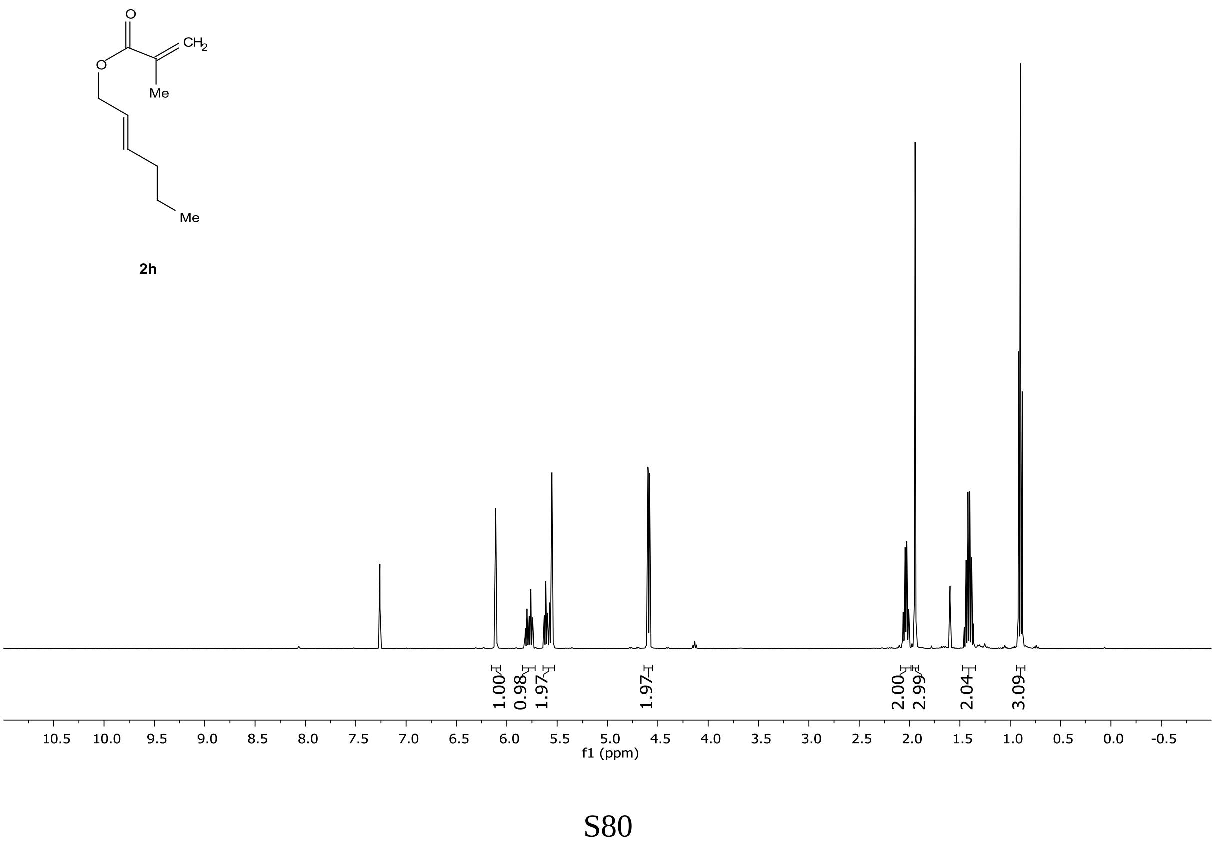

2h, ^{13}C NMR (101 MHz, $CDCl_3$)

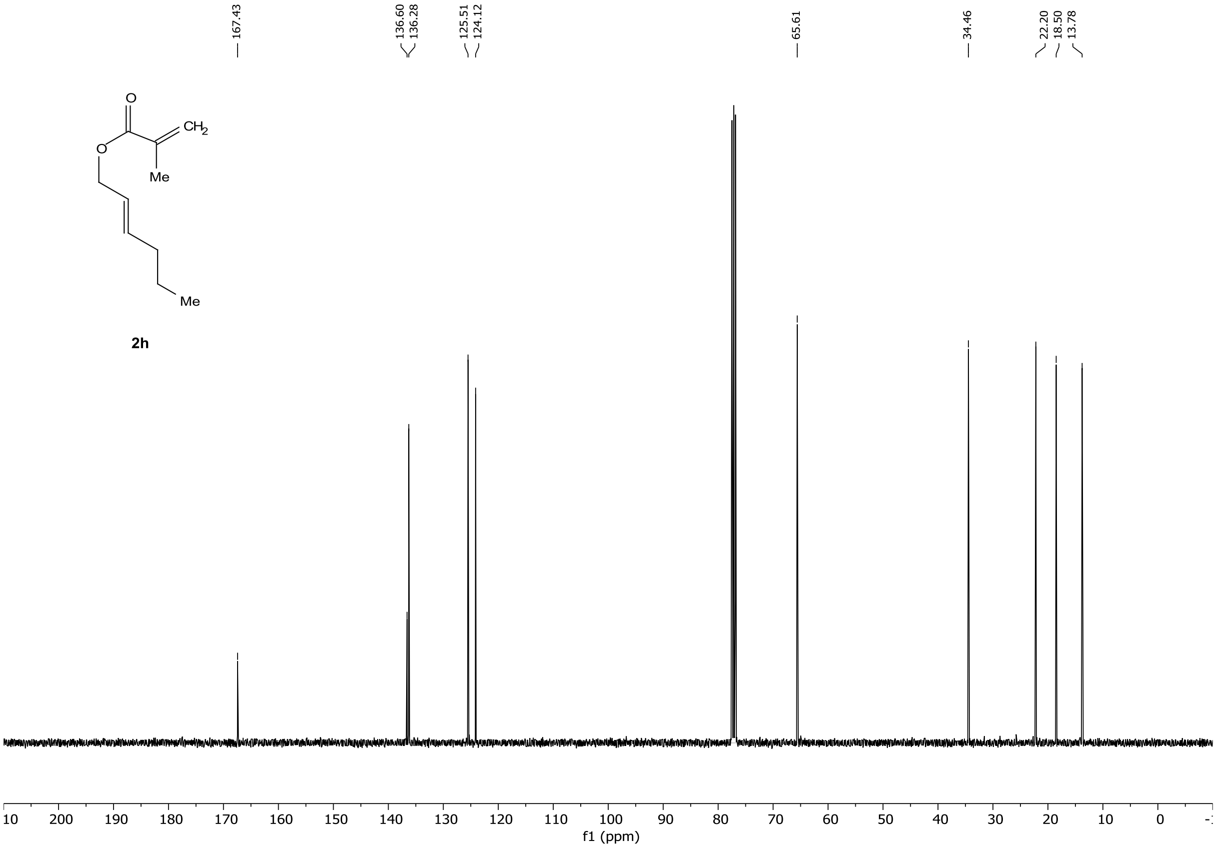

S2i, ^1H NMR (400 MHz, $CDCl_3$)

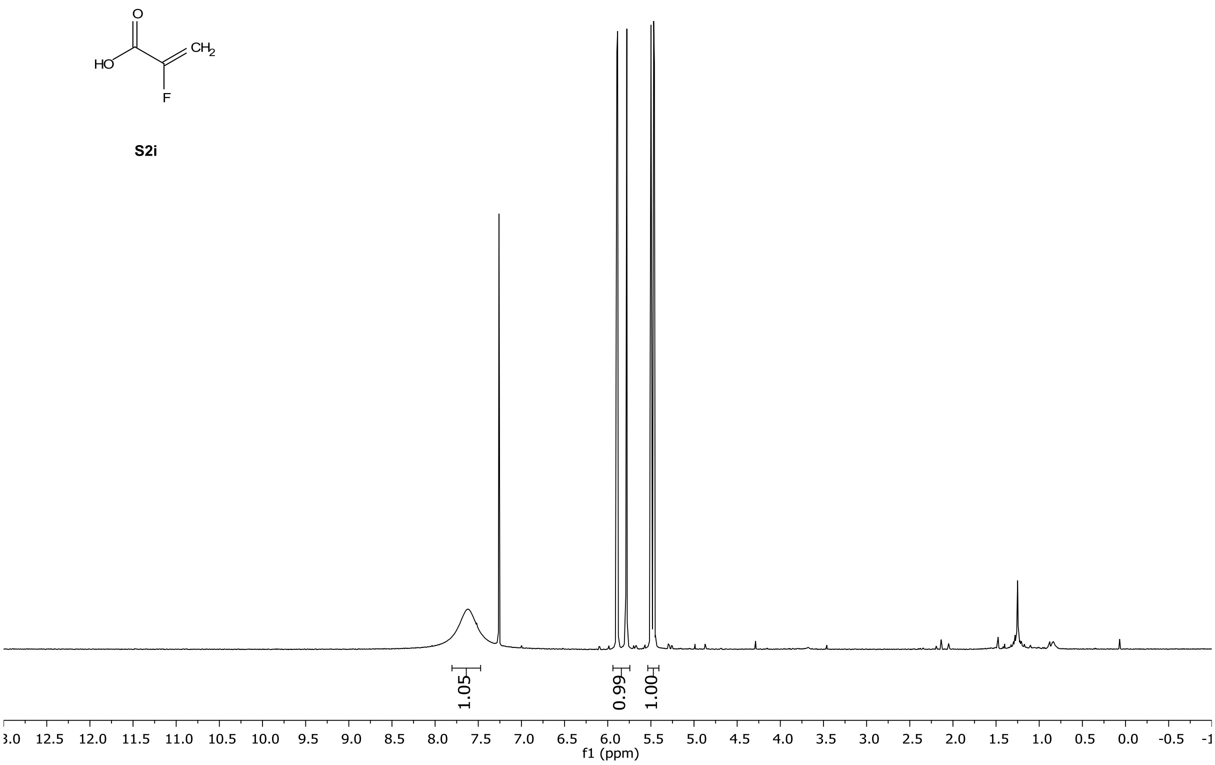

S2i, ^{13}C NMR (101 MHz, $CDCl_3$)

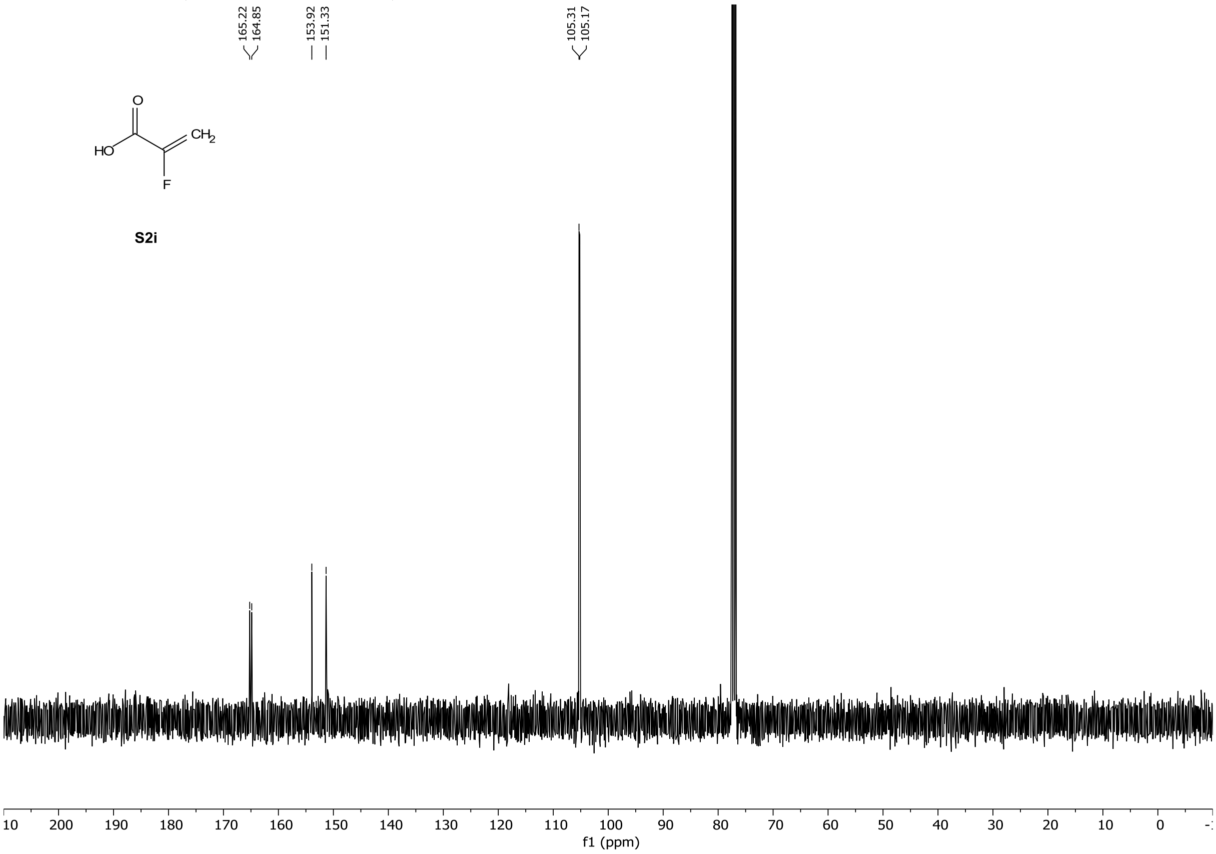

S2i, ^{19}F NMR (377 MHz, $CDCl_3$)

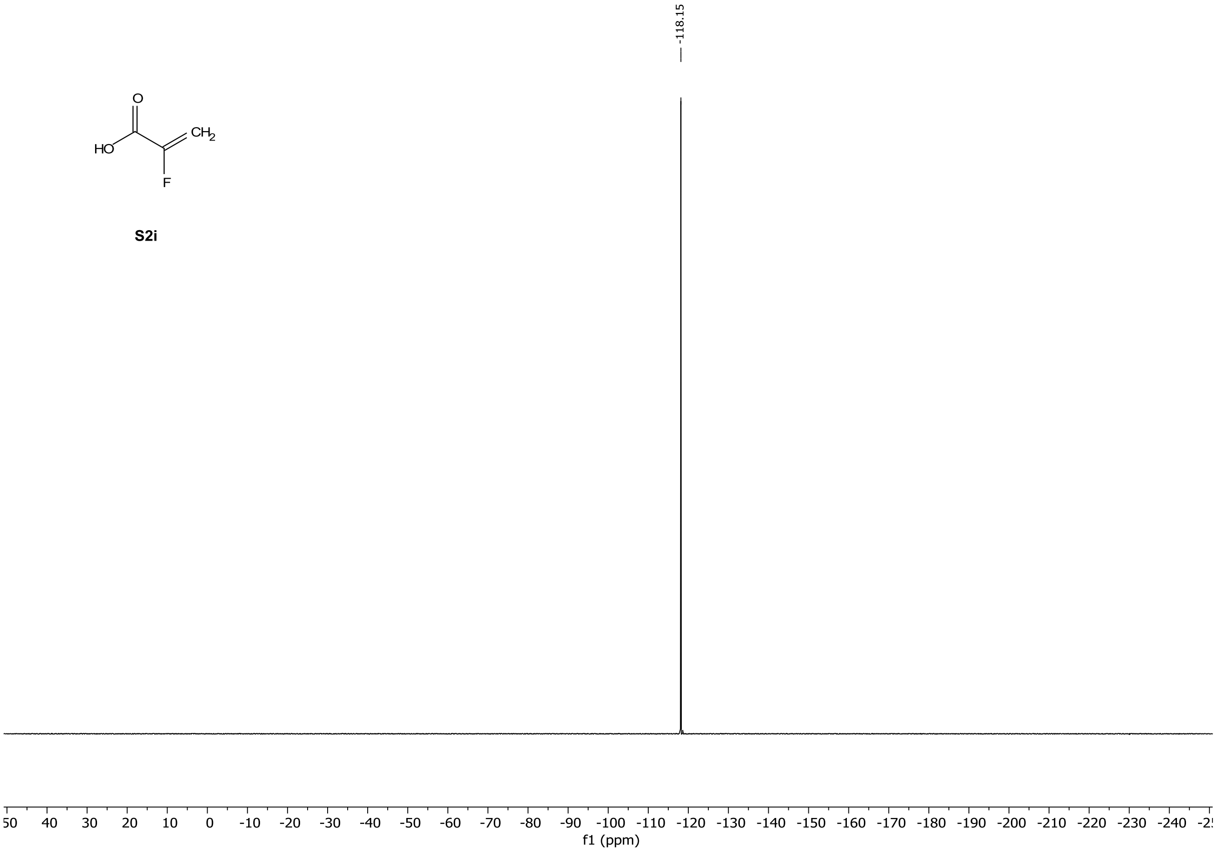

2i, ^{1}H NMR (400 MHz, $CDCl_3$)

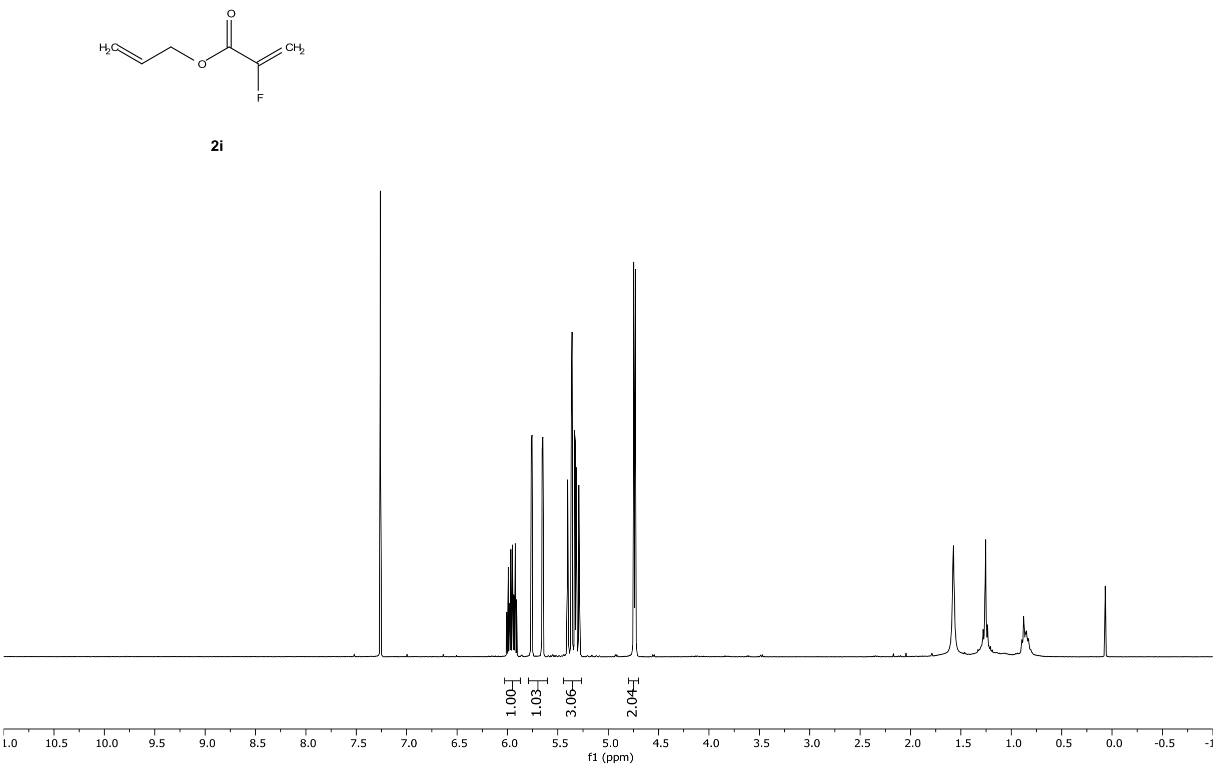

2i, ^{13}C NMR (101 MHz, $CDCl_3$)

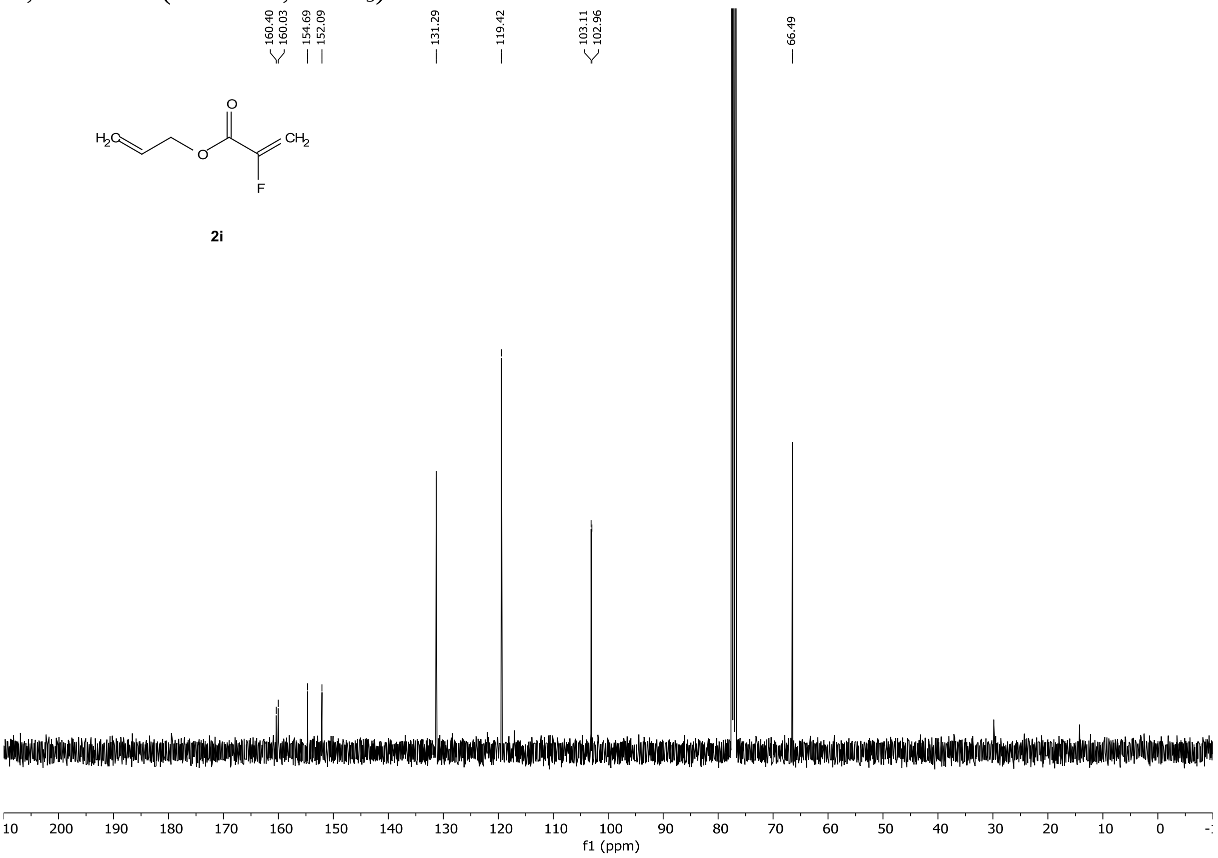

2i, ^{19}F NMR (377 MHz, $CDCl_3$)

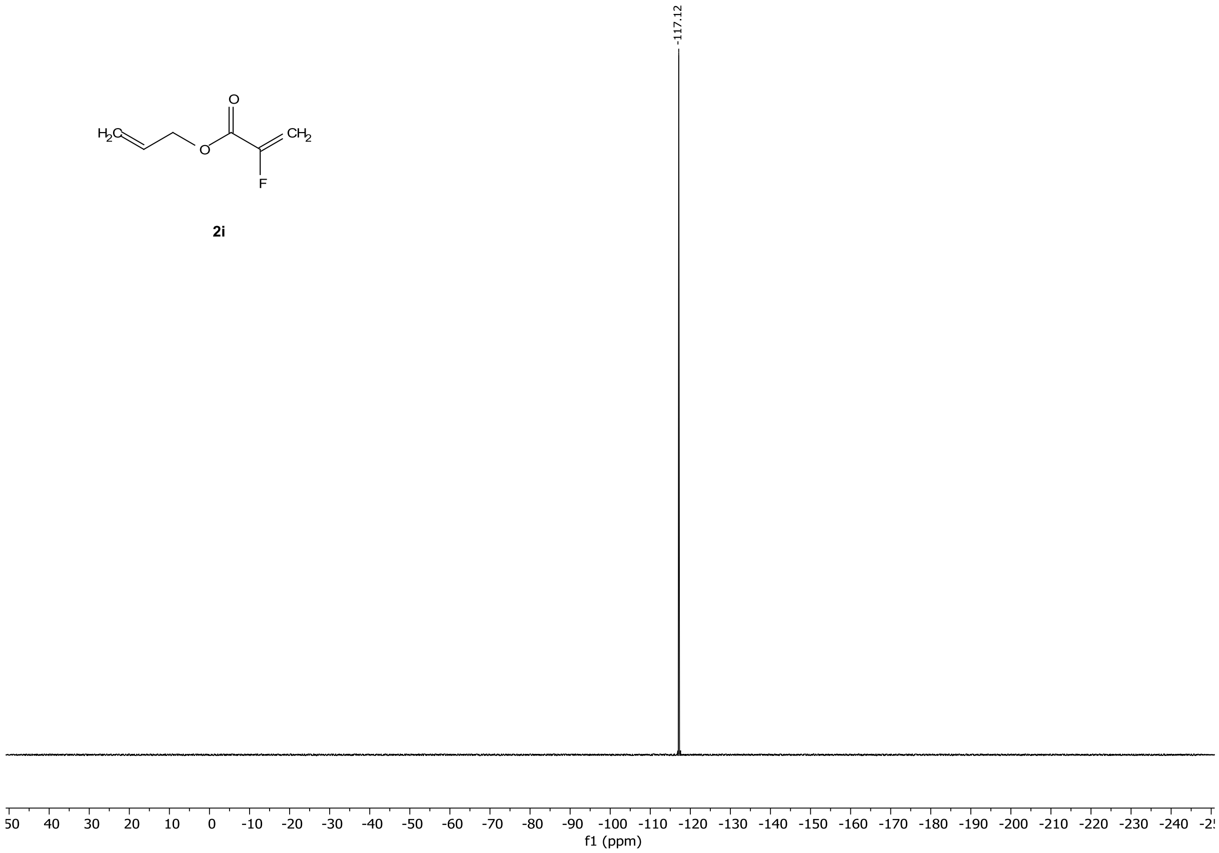

2j, ^{1}H NMR (400 MHz, $CDCl_3$)

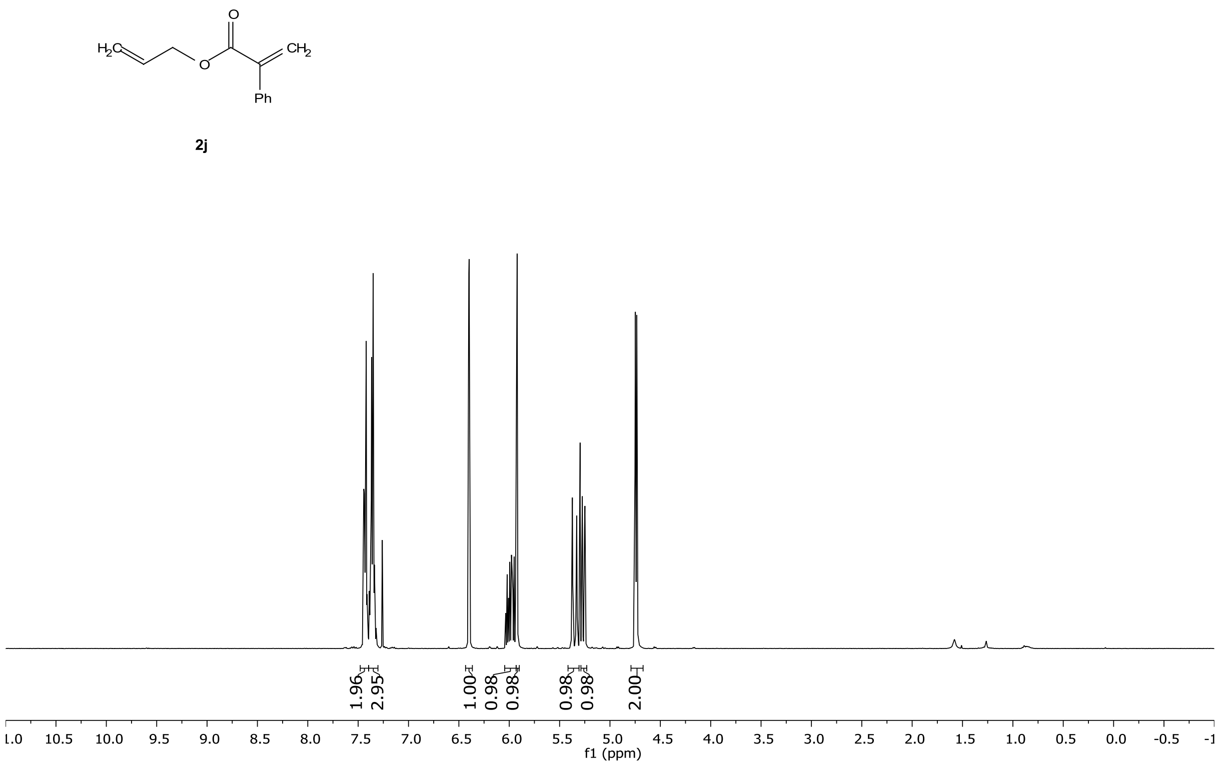

2j, ^{13}C NMR (101 MHz, $CDCl_3$)

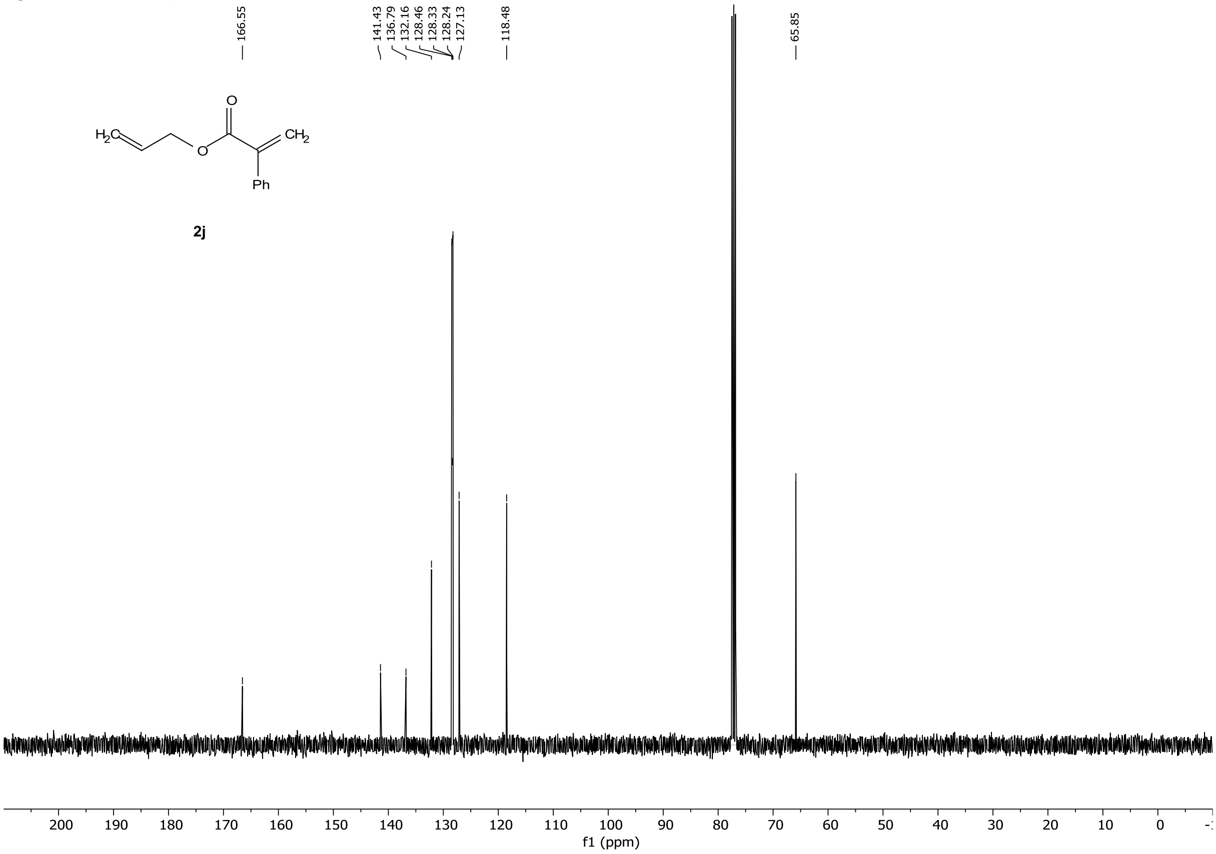

S2k, ^{1}H NMR (400 MHz, $CDCl_3$)

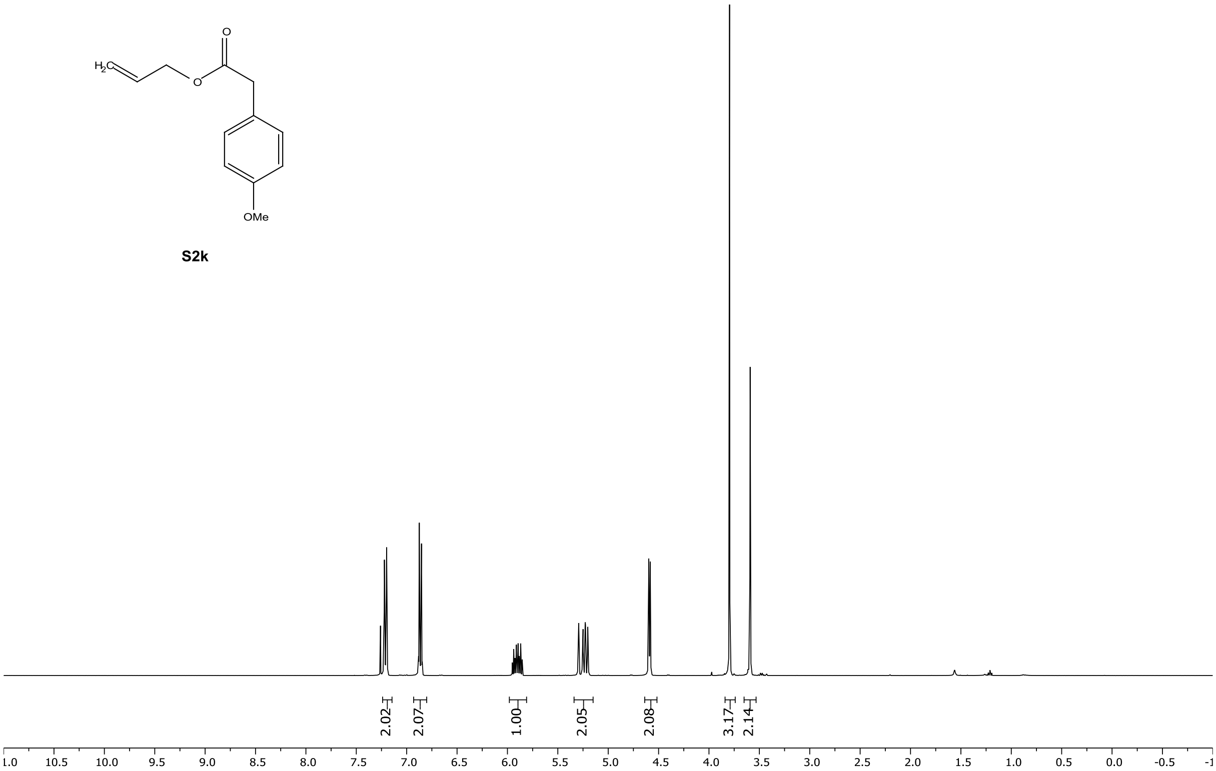

S2k, ^{13}C NMR (101 MHz, $CDCl_3$)

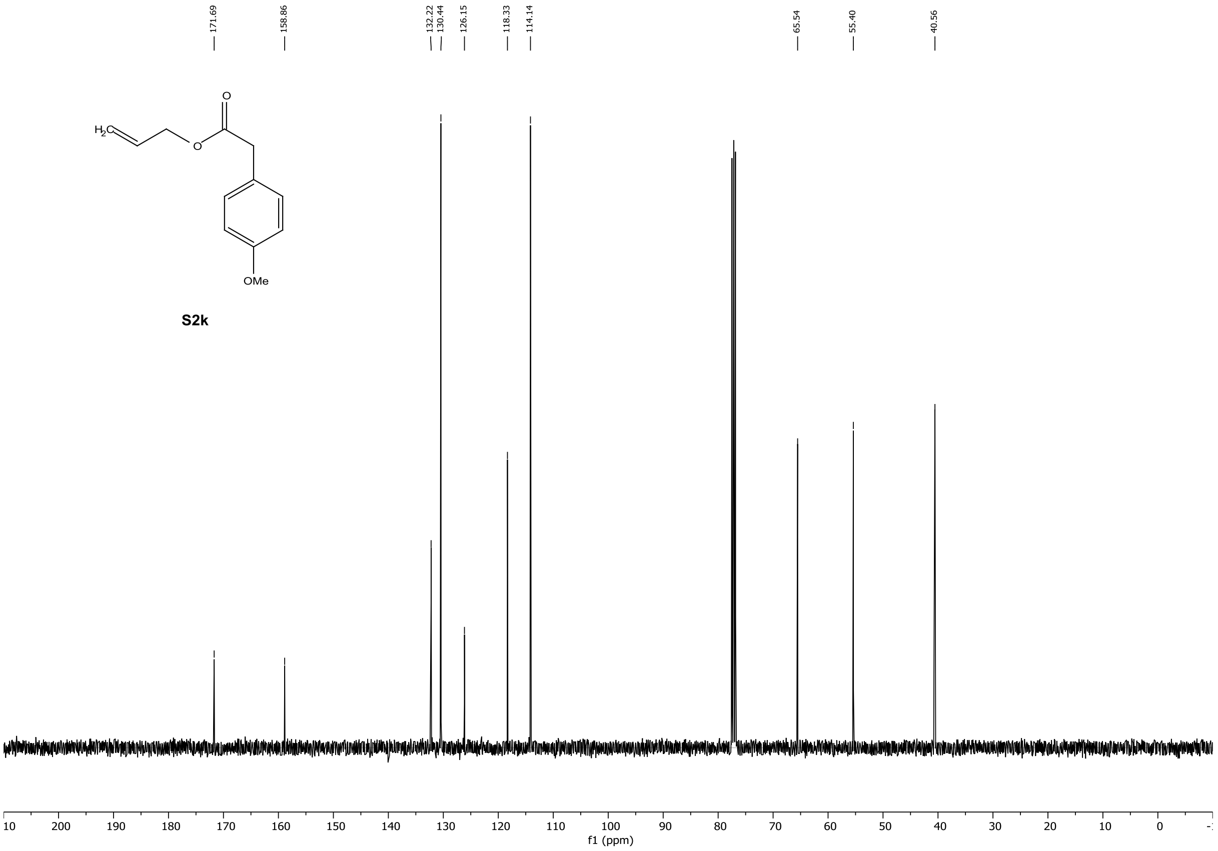

2k, ^{1}H NMR (400 MHz, $CDCl_3$)

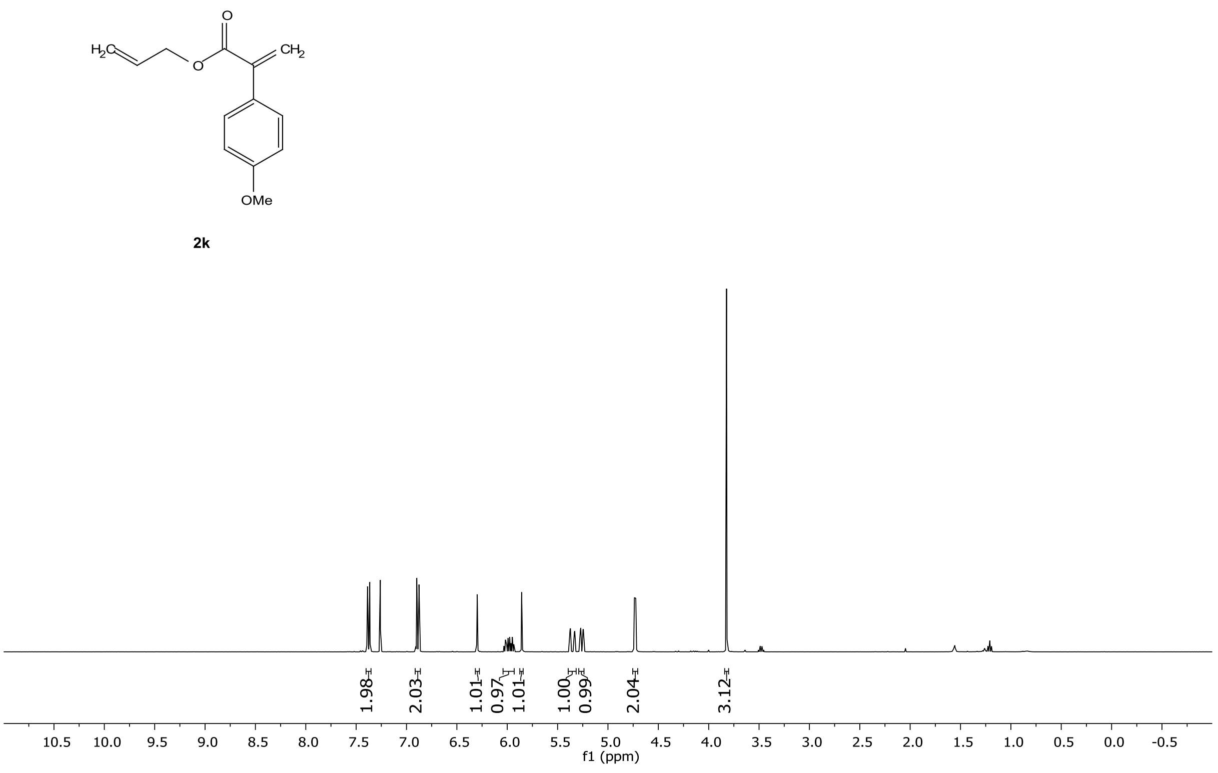

2k, ^{13}C NMR (101 MHz, $CDCl_3$)

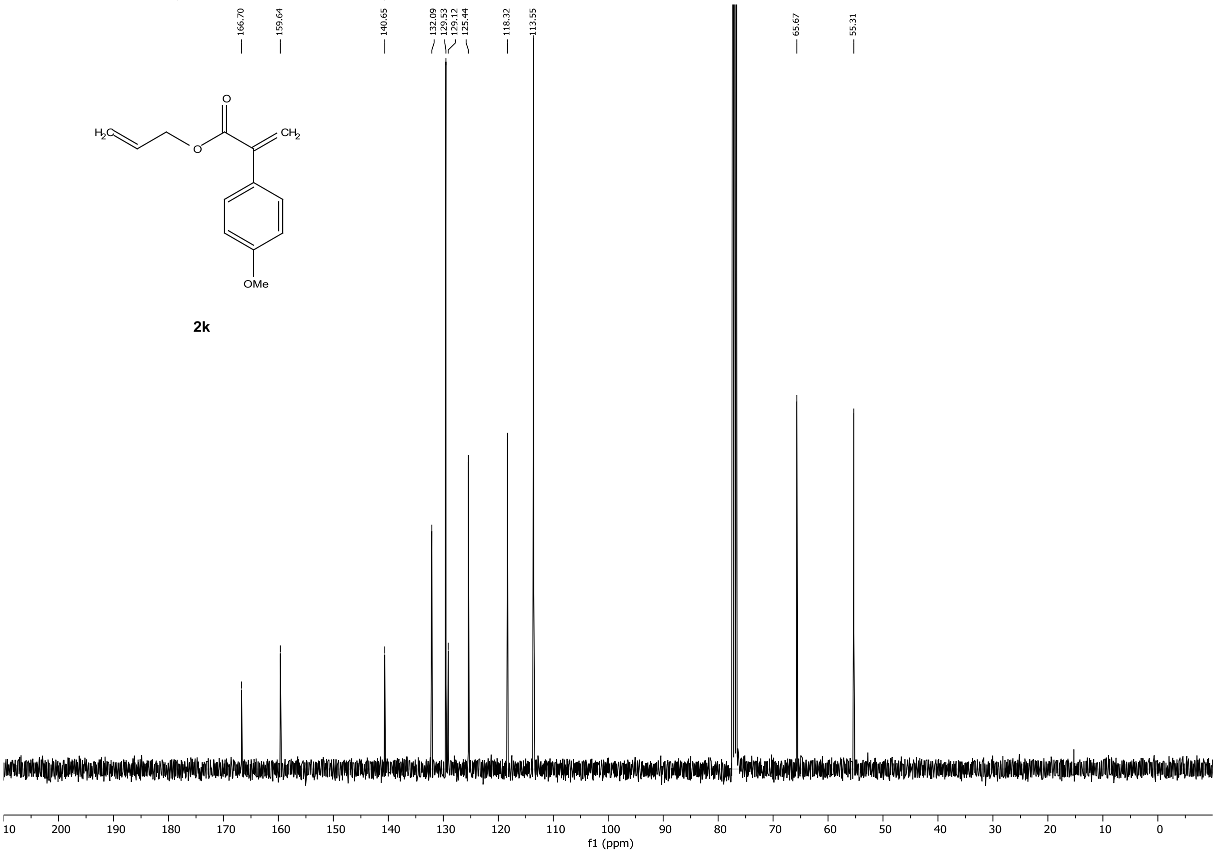

S2l, ^{1}H NMR (400 MHz, $CDCl_3$)

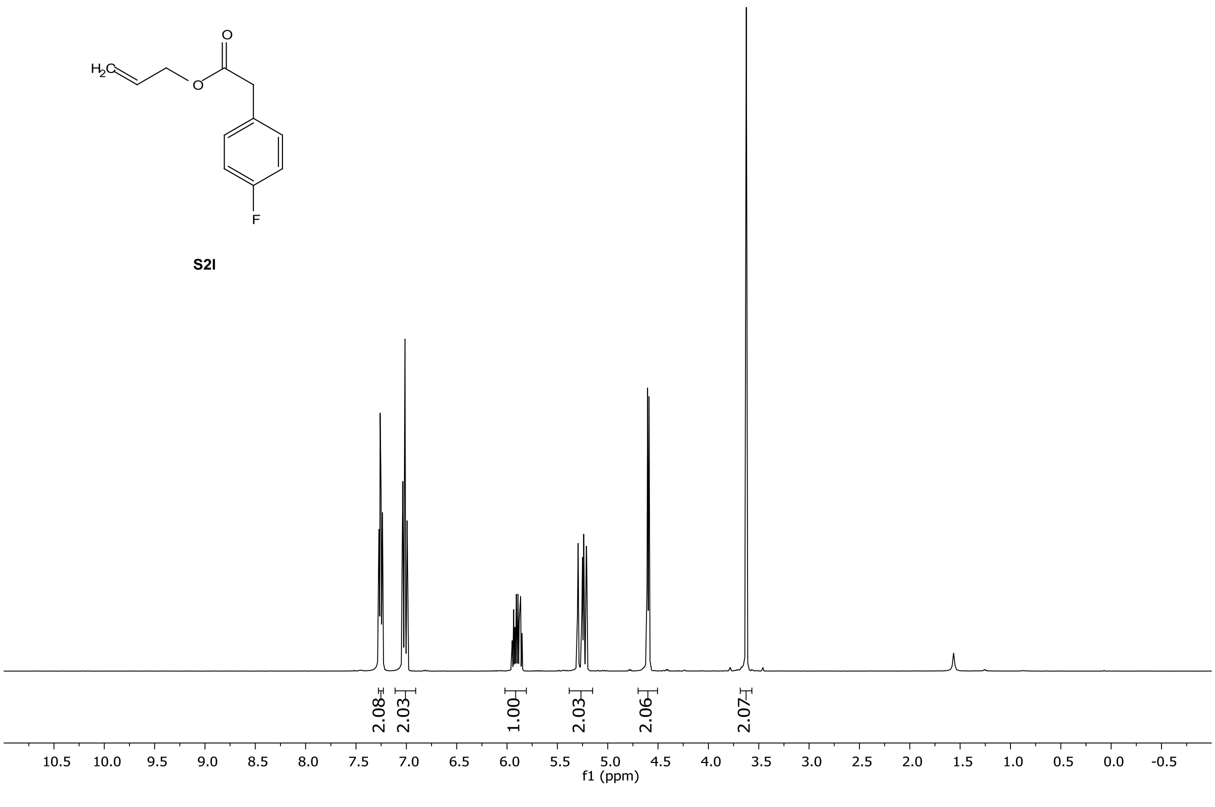

S2l, ^{13}C NMR (101 MHz, $CDCl_3$)

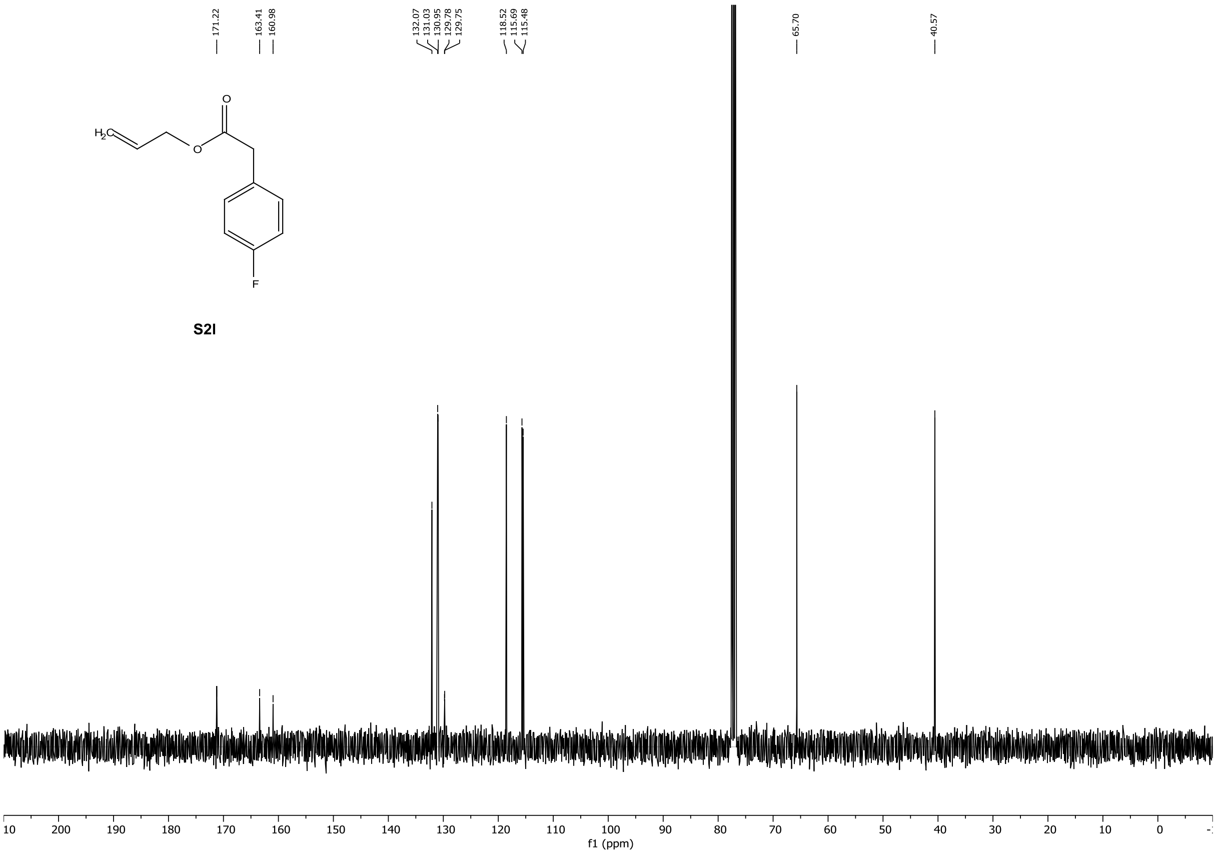

S2l, ^{19}F NMR (377 MHz, $CDCl_3$)

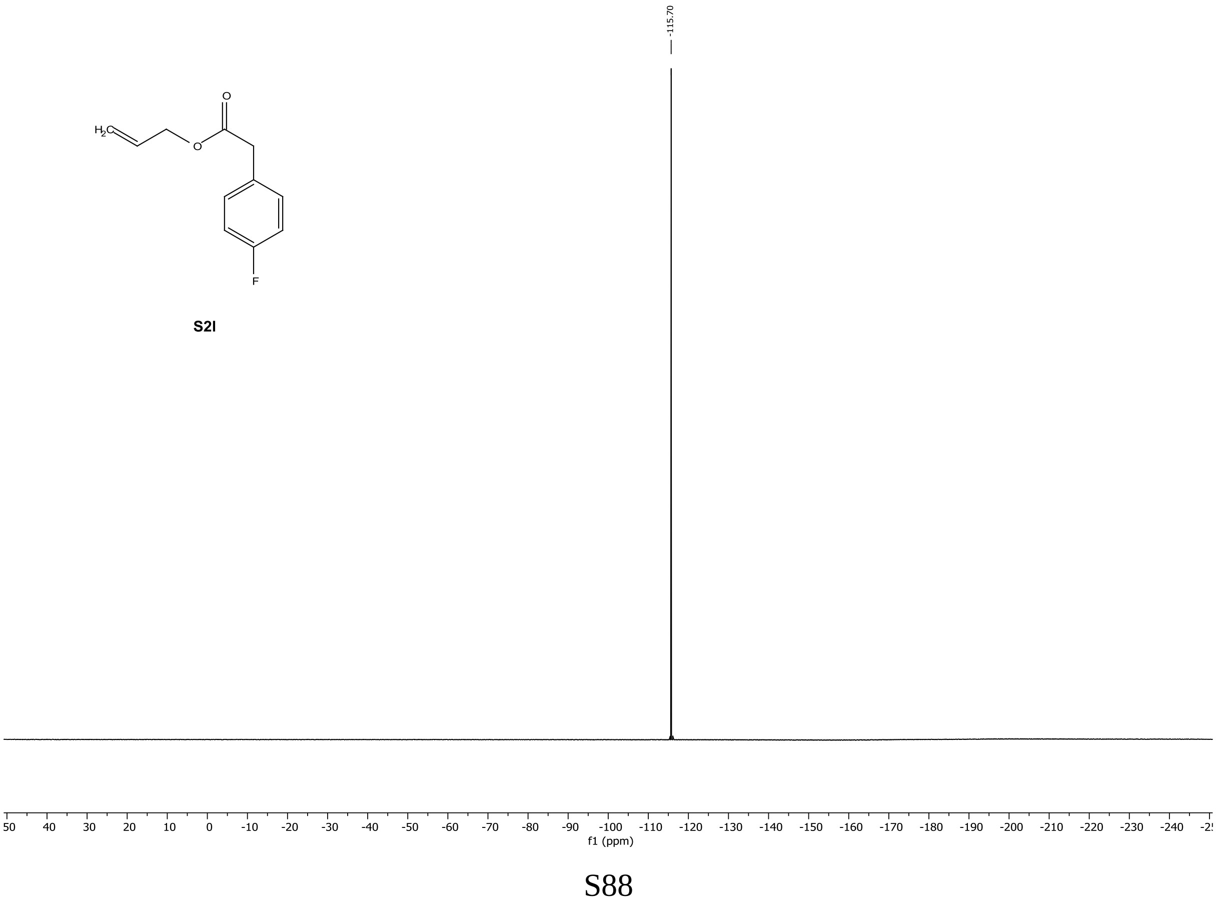

2l, ^{1}H NMR (400 MHz, $CDCl_3$)

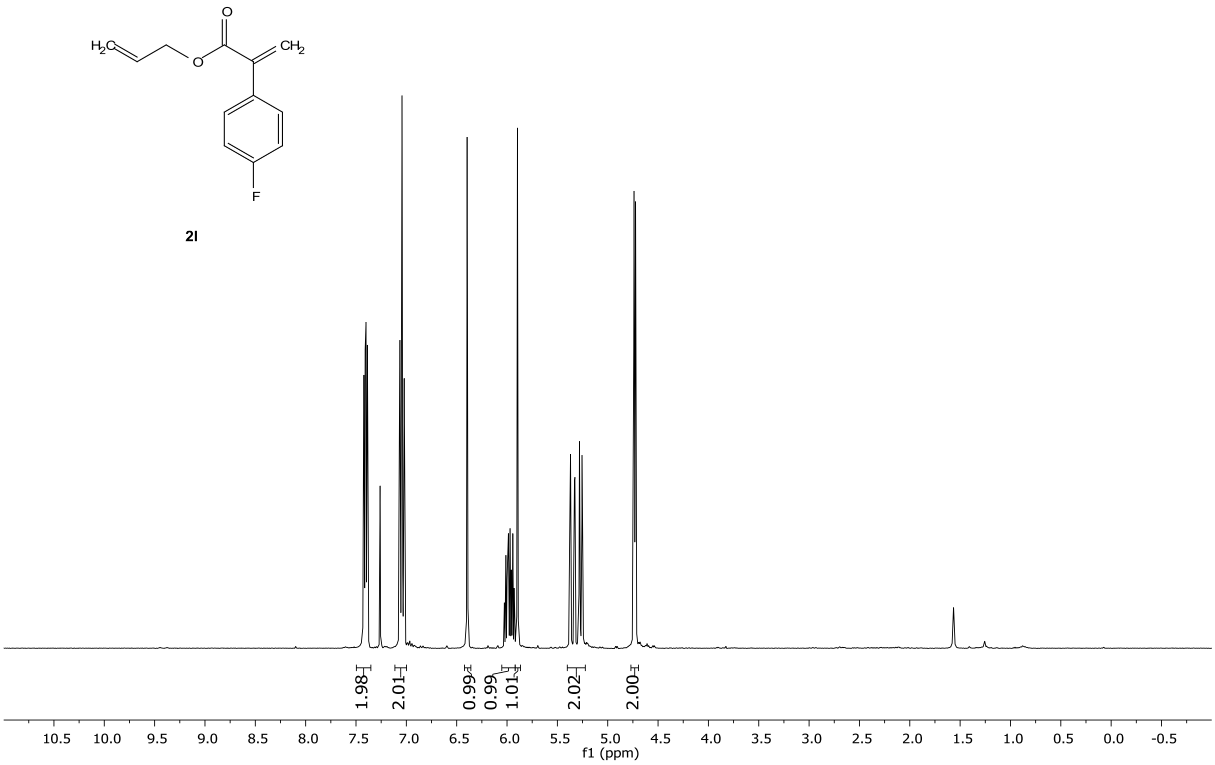

2l, ^{13}C NMR (101 MHz, $CDCl_3$)

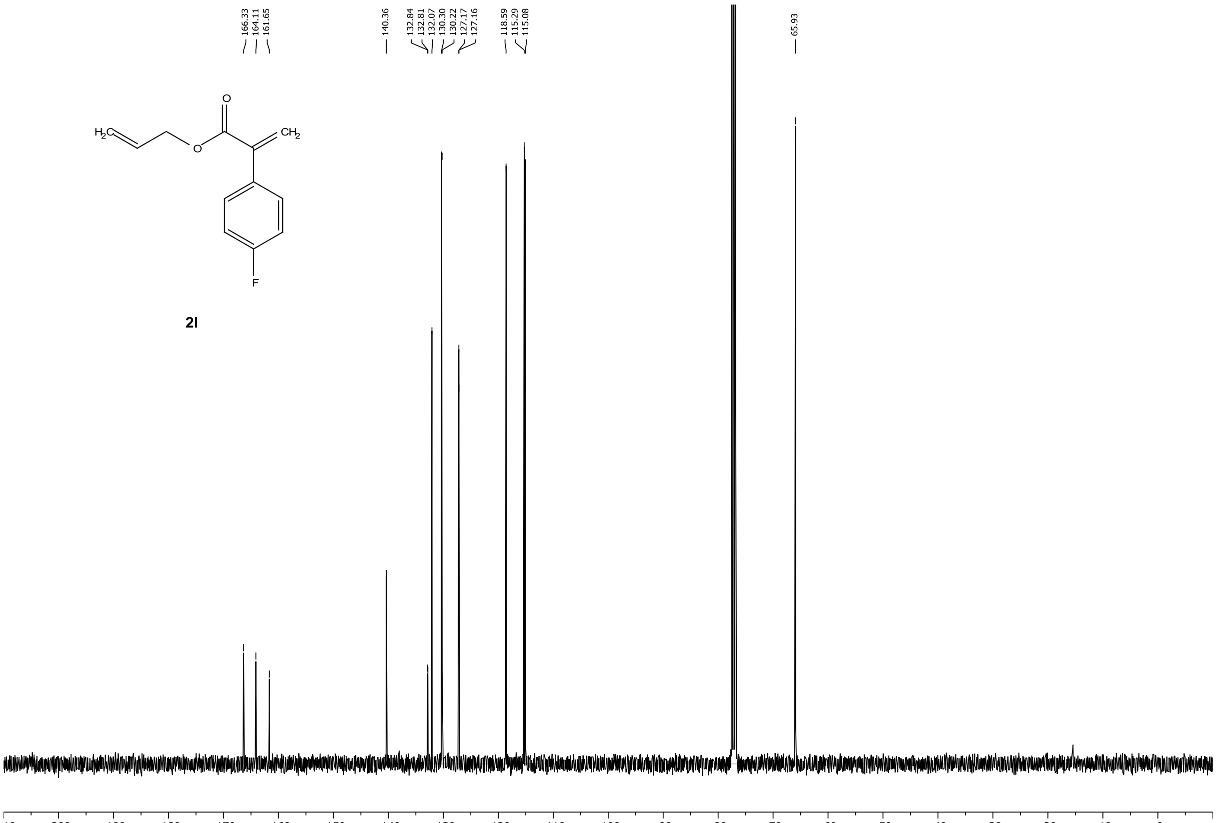

2l, ^{19}F NMR (377 MHz, $CDCl_3$)

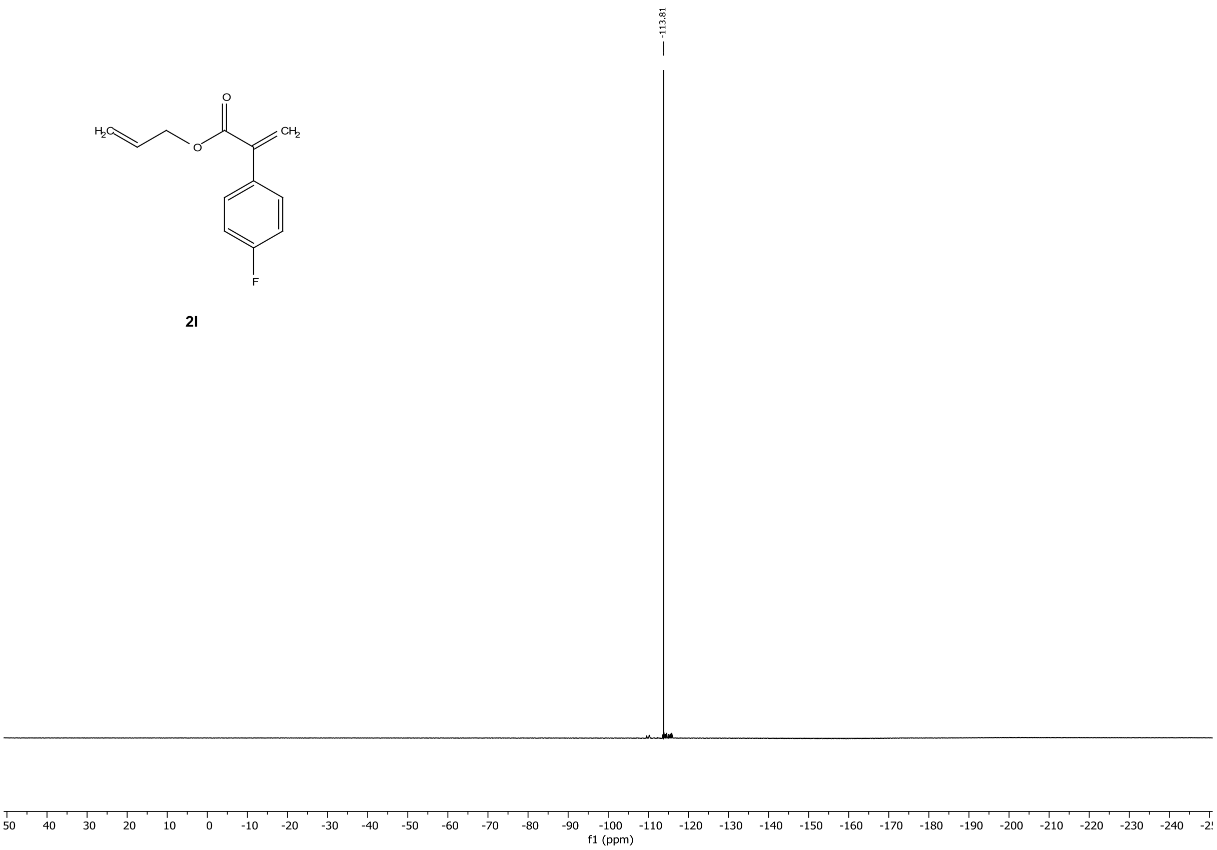

S2m, ^{1}H NMR (400 MHz, $CDCl_3$)

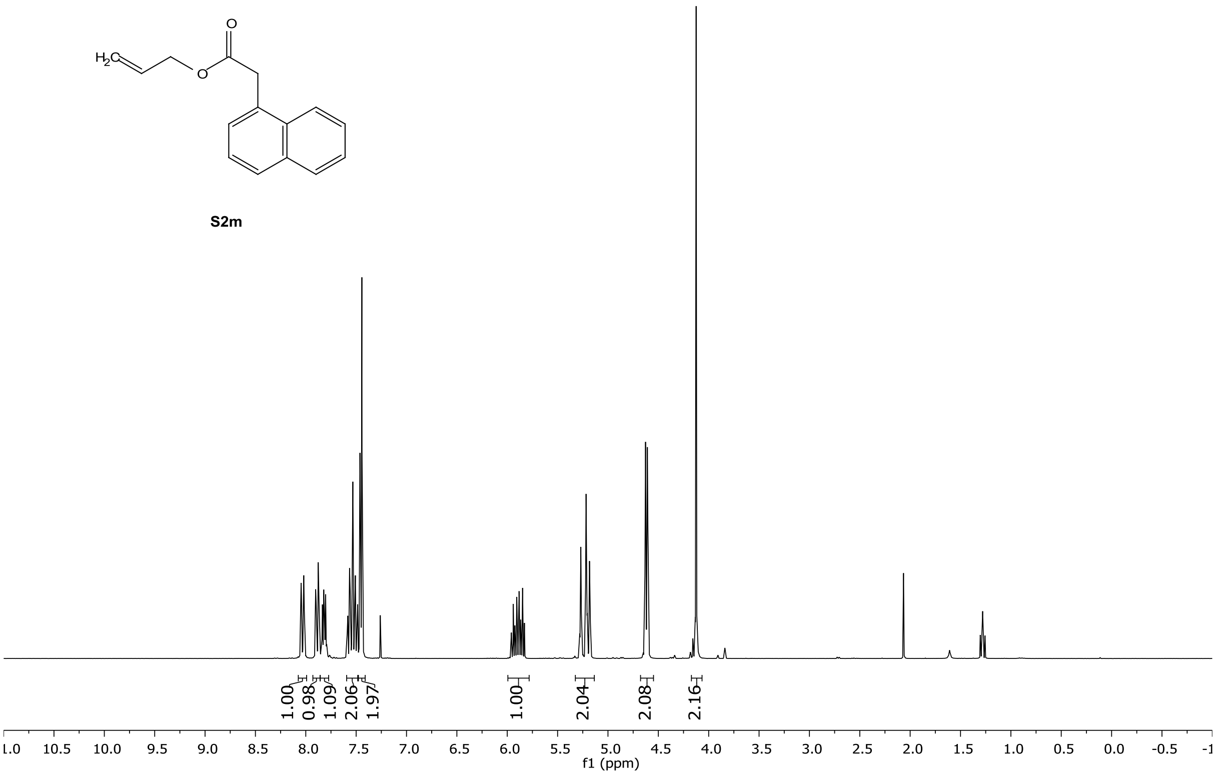

S2m, ^{13}C NMR (101 MHz, $CDCl_3$)

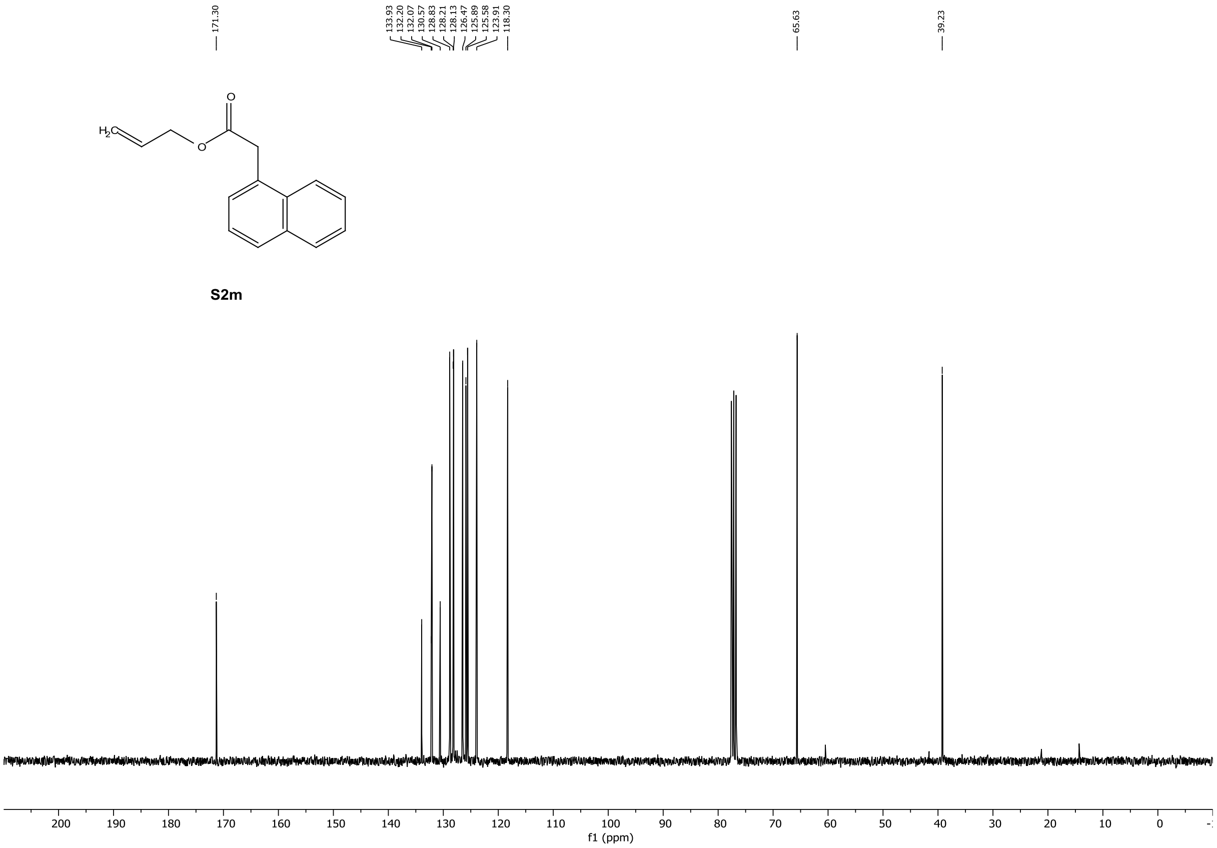

2m, ^{1}H NMR (400 MHz, $CDCl_3$)

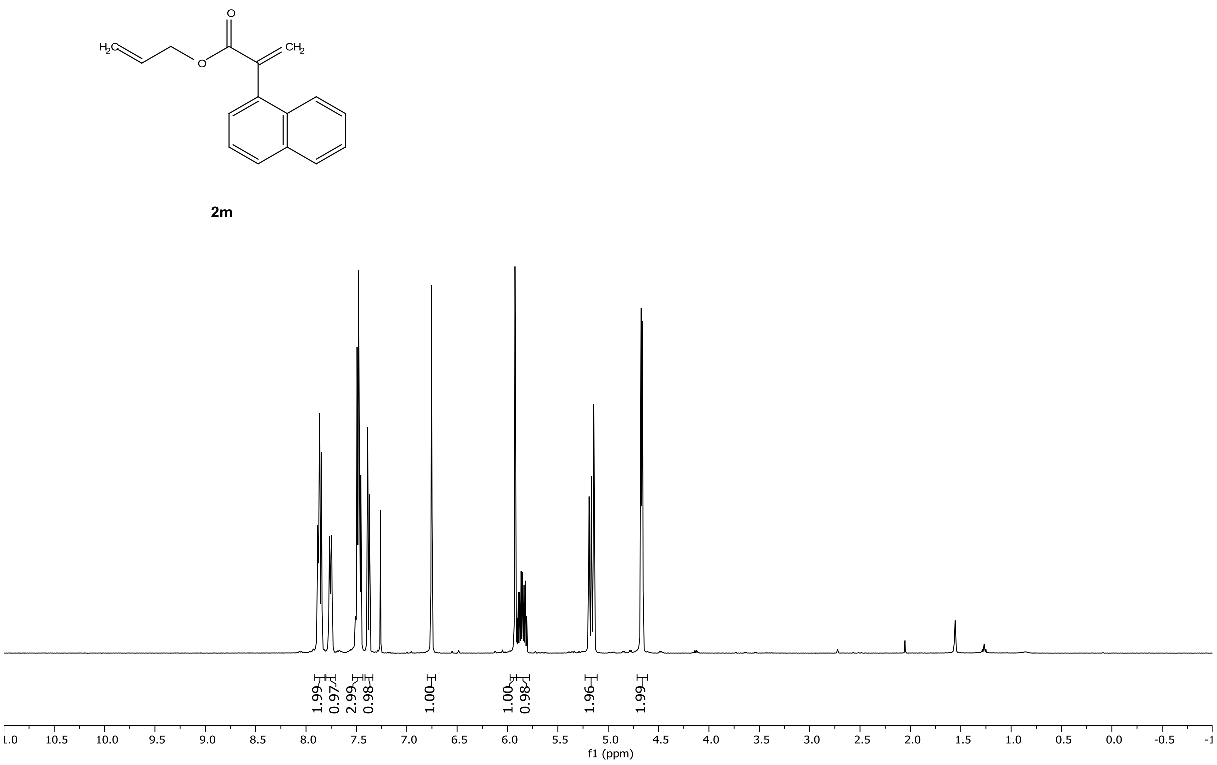

2m, ^{13}C NMR (101 MHz, $CDCl_3$)

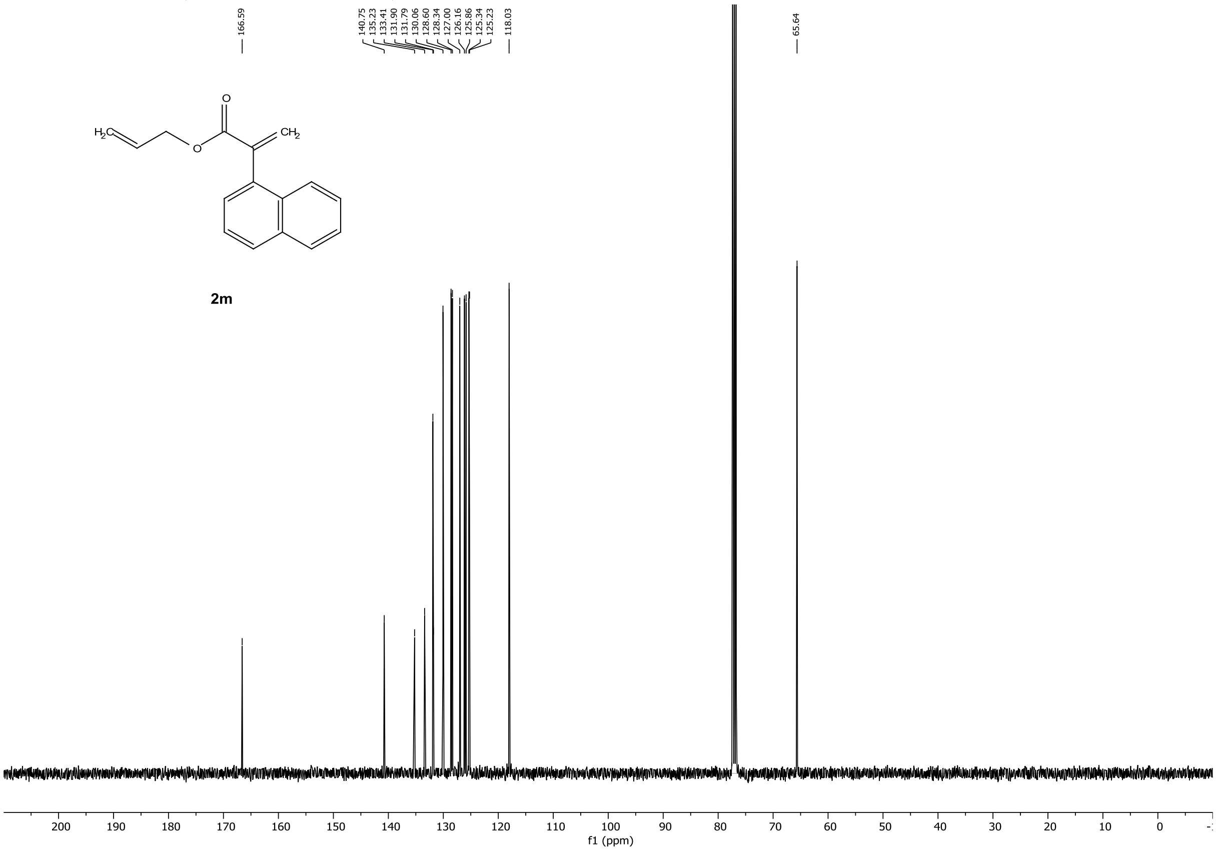

2n, ^{1}H NMR (400 MHz, $CDCl_3$)

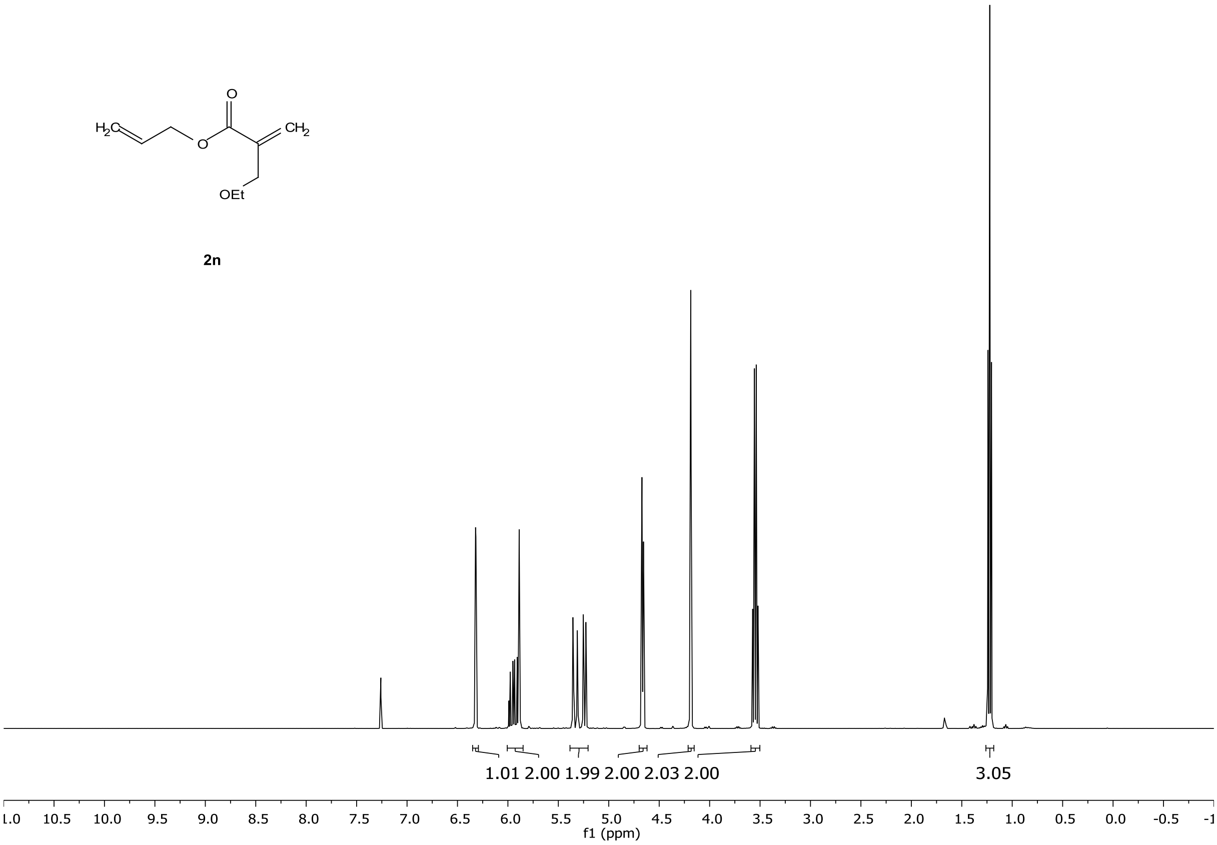

2n, ^{13}C NMR (101 MHz, $CDCl_3$)

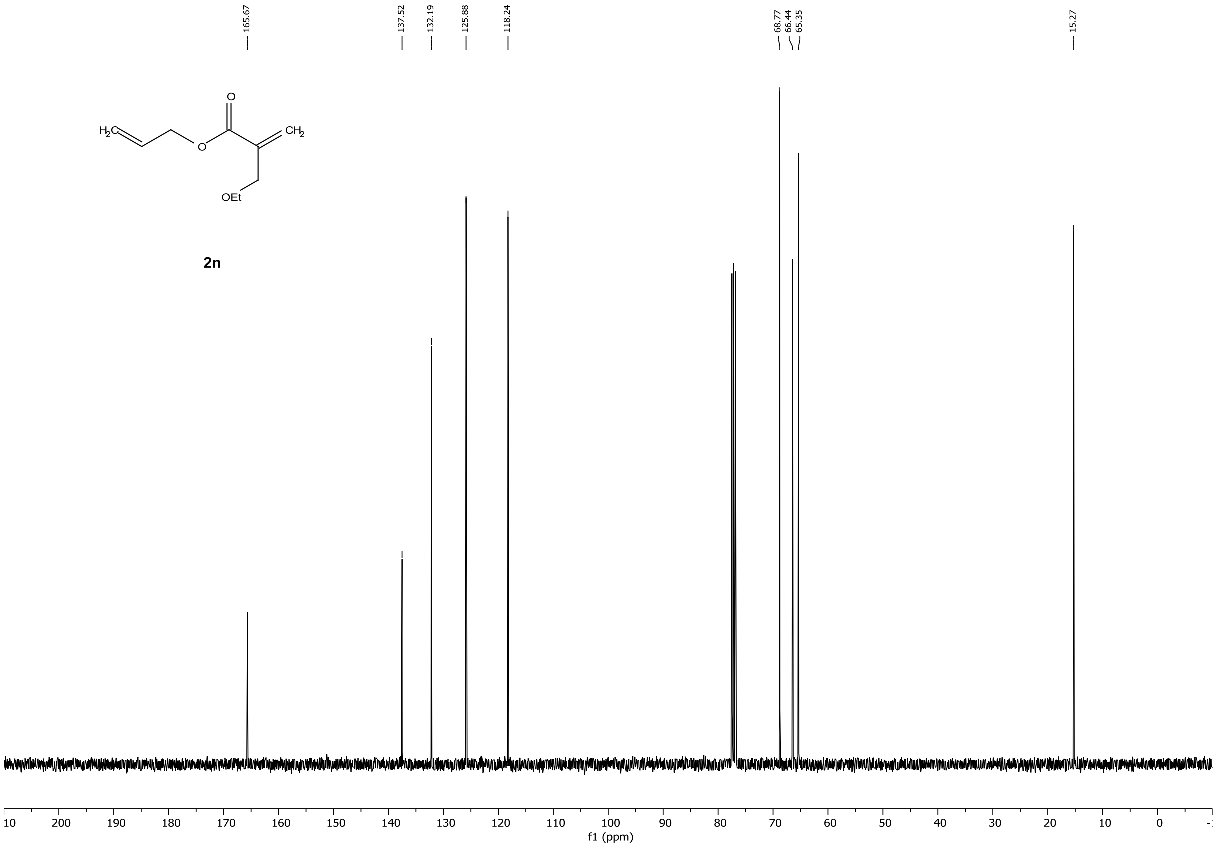

2o, ^{1}H NMR (400 MHz, $CDCl_3$)

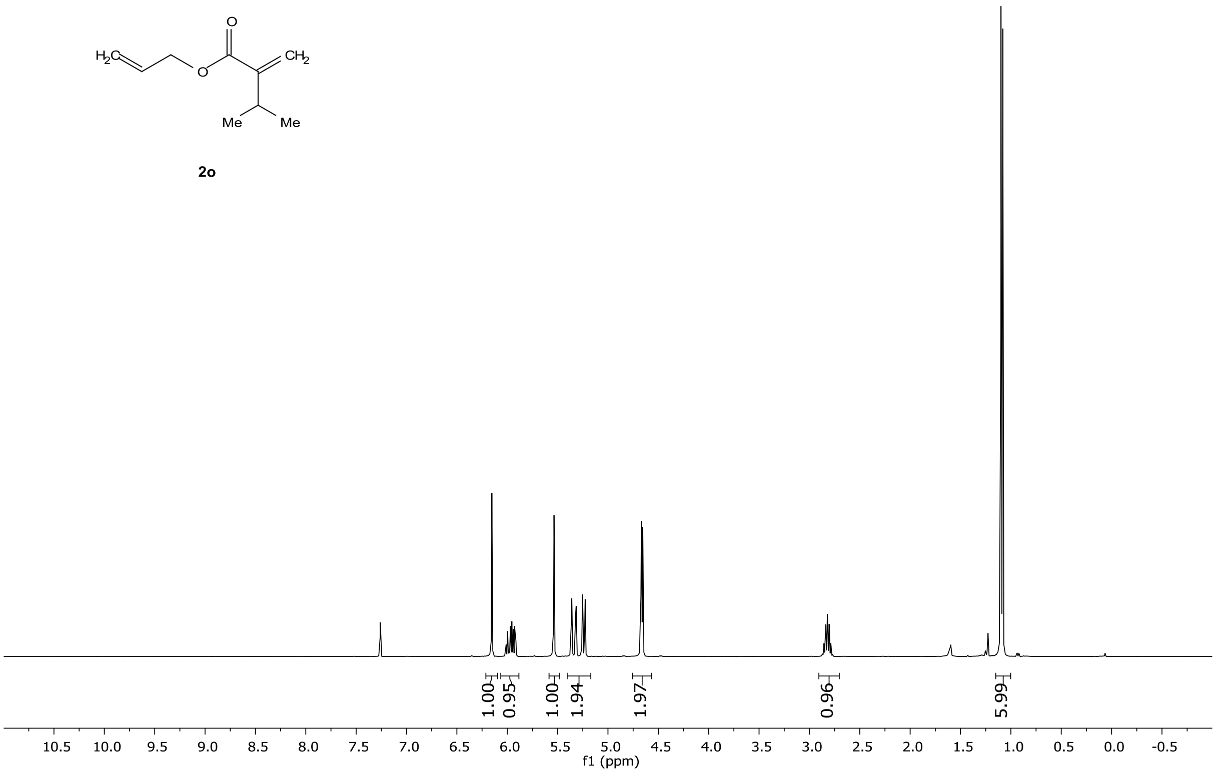

2o, ^{13}C NMR (101 MHz, $CDCl_3$)

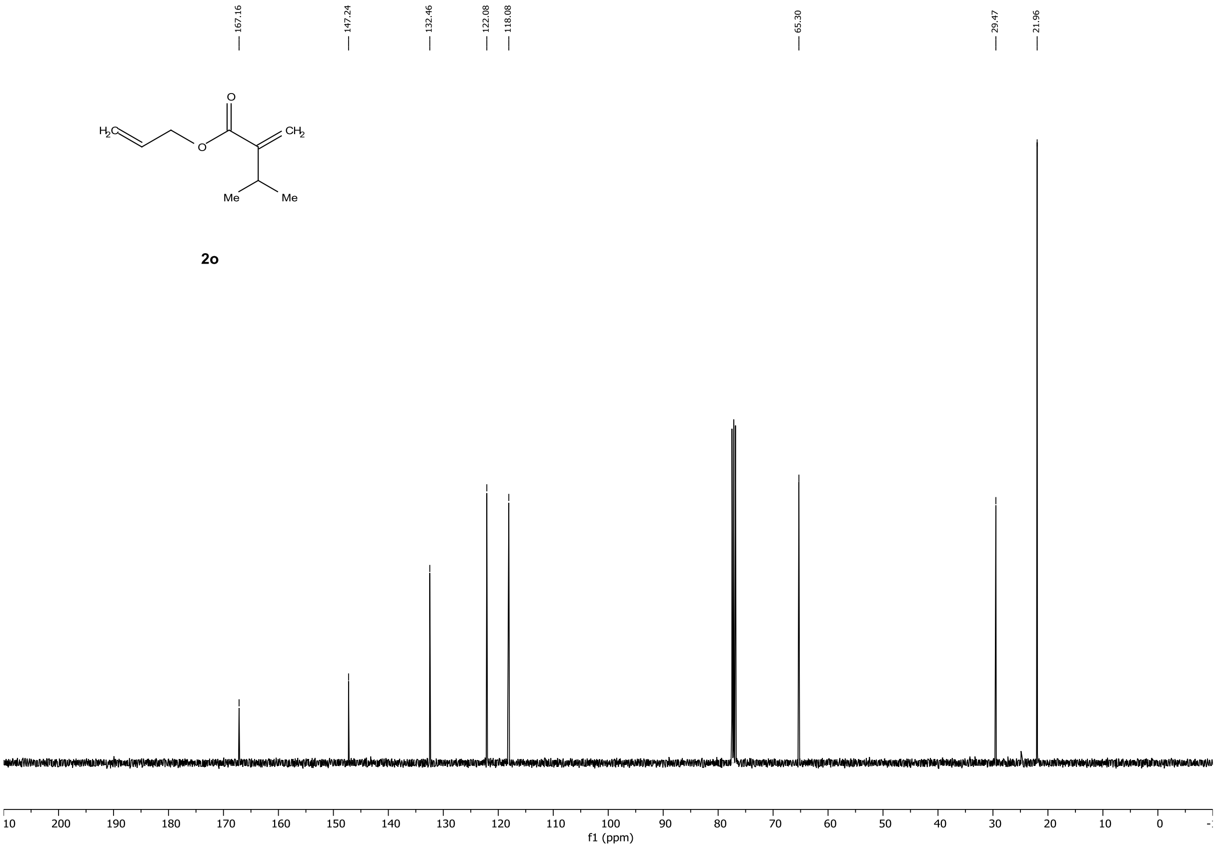

2p, ^{1}H NMR (400 MHz, $CDCl_3$)

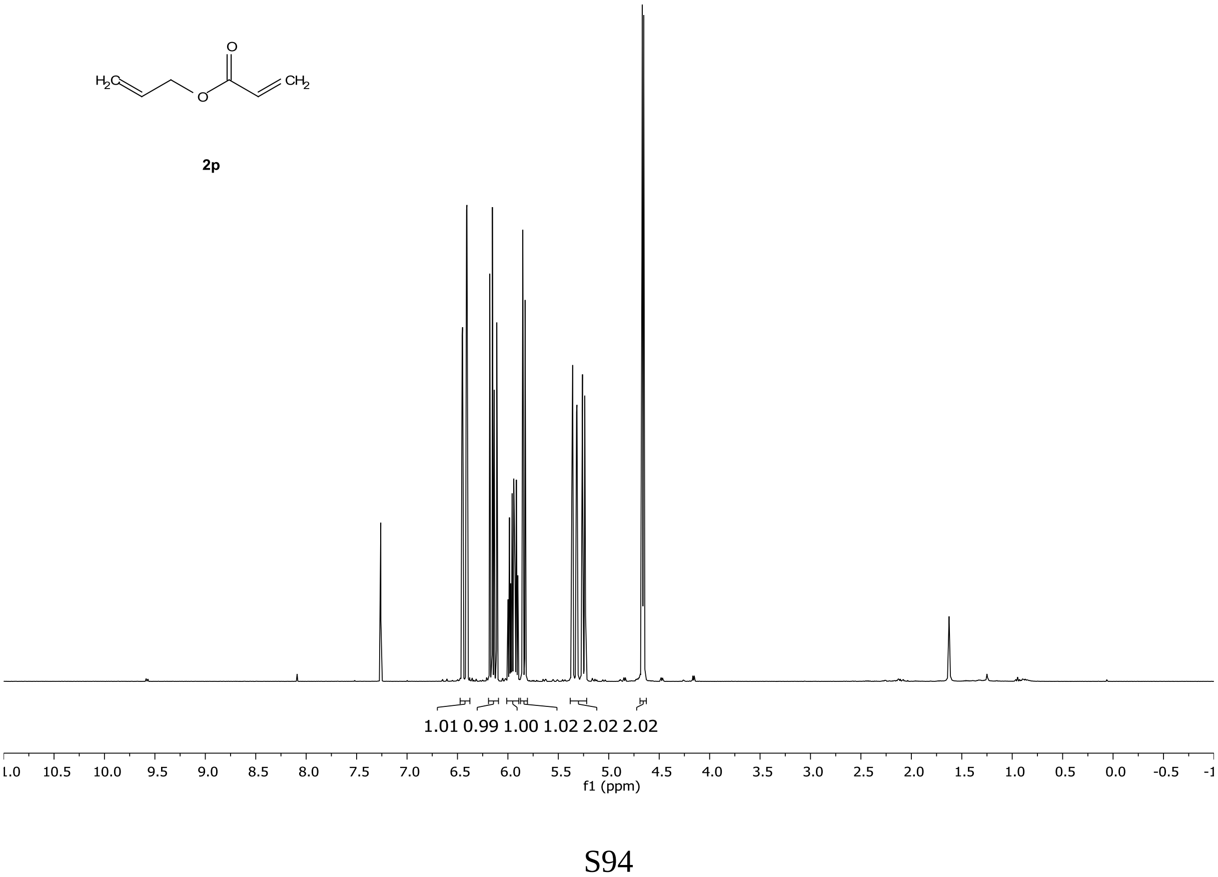

2p, ^{13}C NMR (101 MHz, $CDCl_3$)

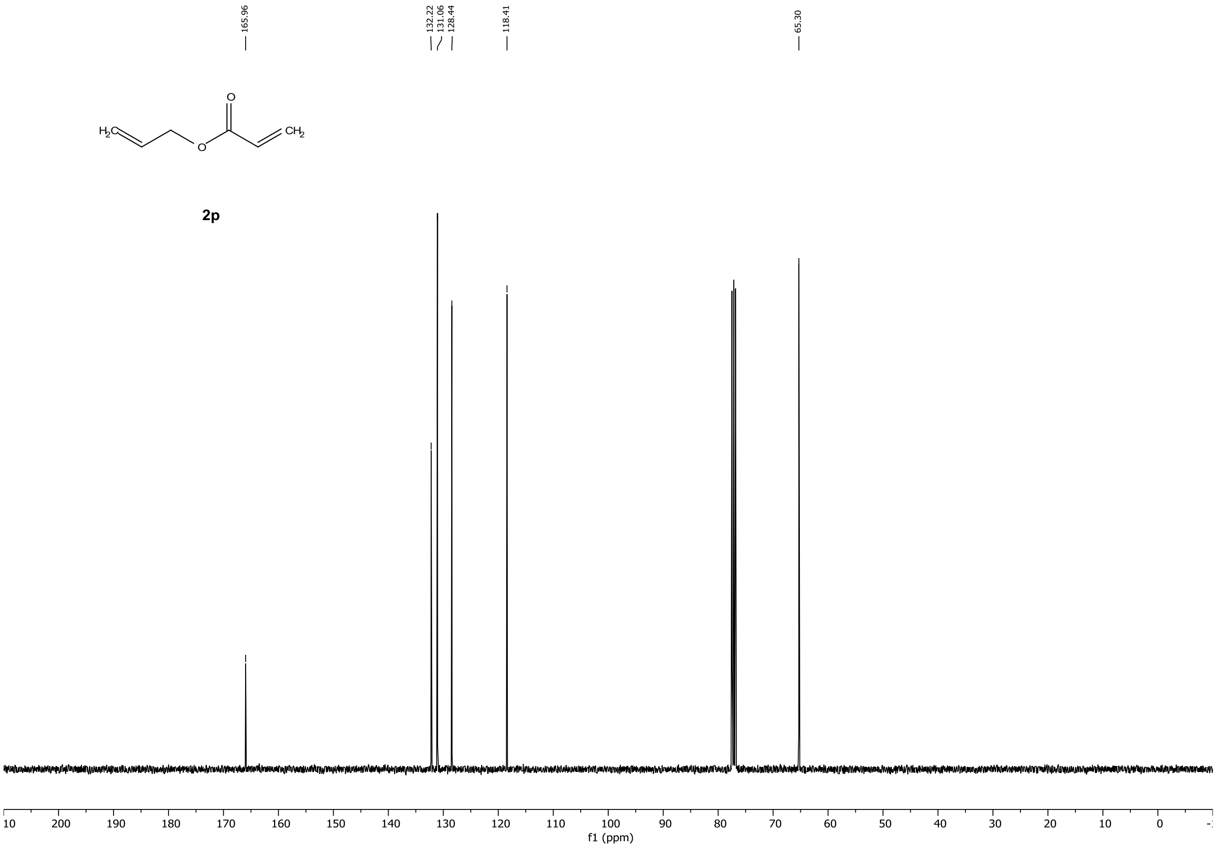

4g, ^{1}H NMR (300 MHz, $CDCl_3$)

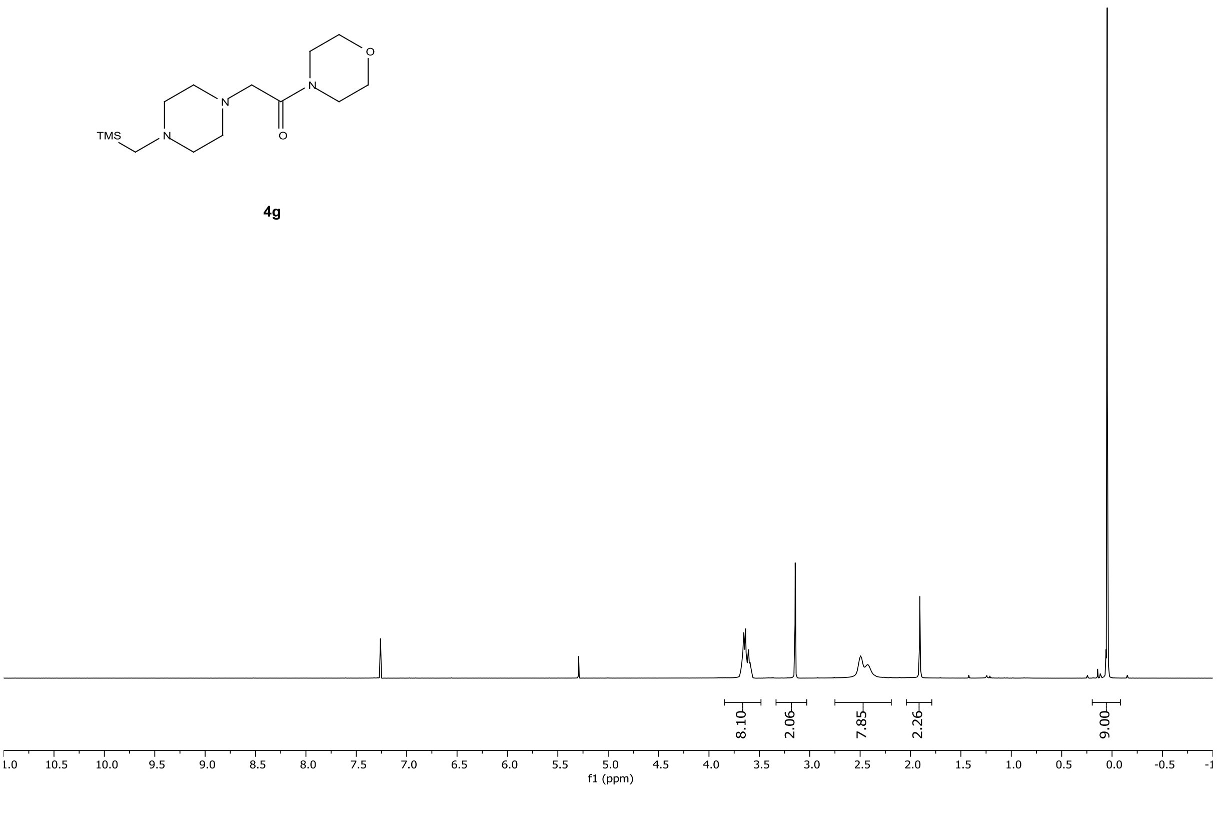

4g, ^{13}C NMR (75 MHz, $CDCl_3$)

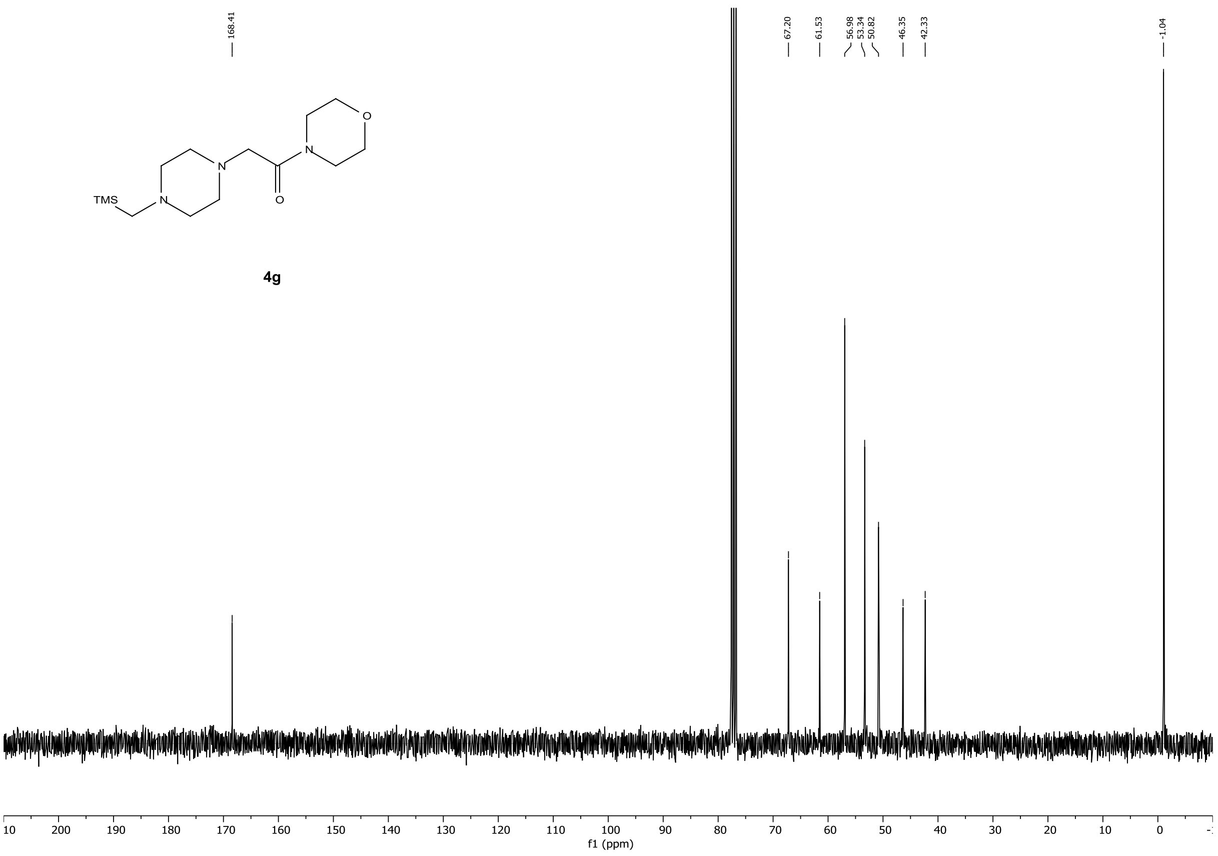

S1-4i, ^{1}H NMR (400 MHz, $CDCl_3$)

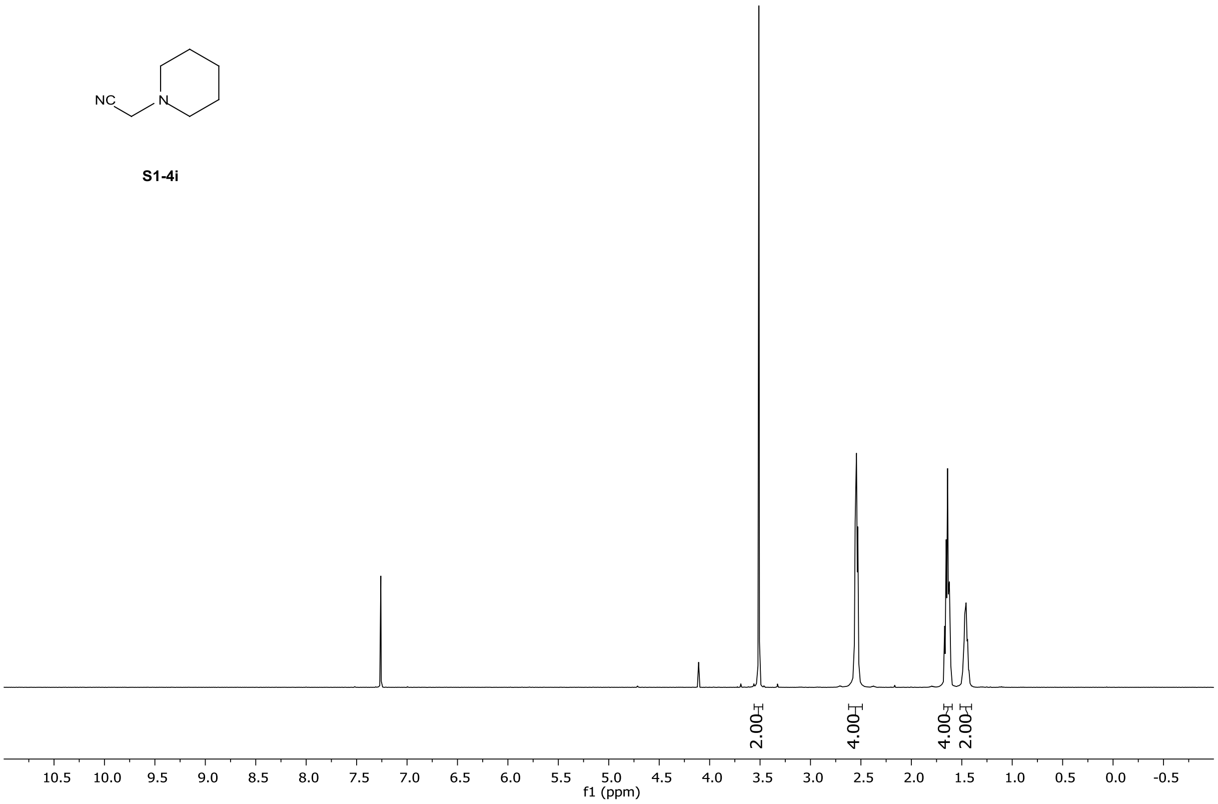

S1-4i, ^{13}C NMR (101 MHz, $CDCl_3$)

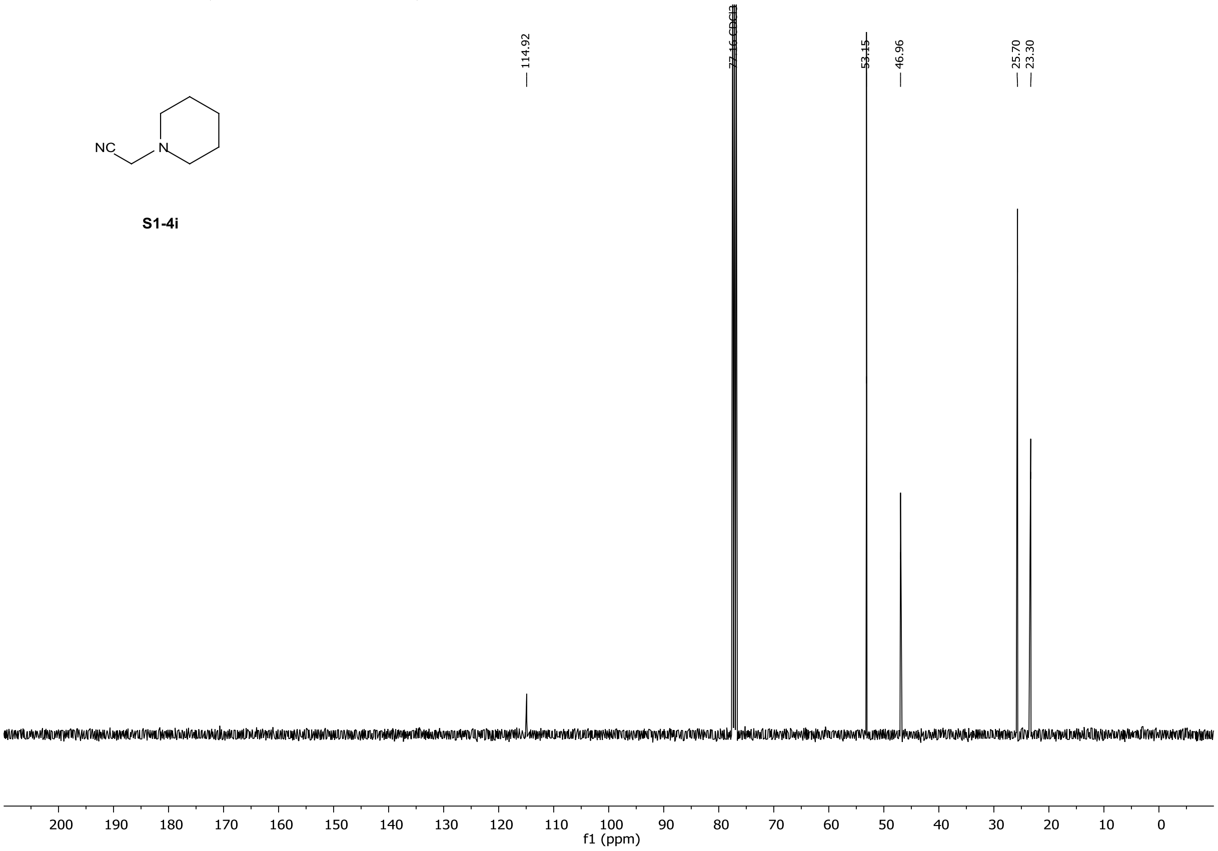

S2-4i, ^{1}H NMR (400 MHz, $CDCl_3$)

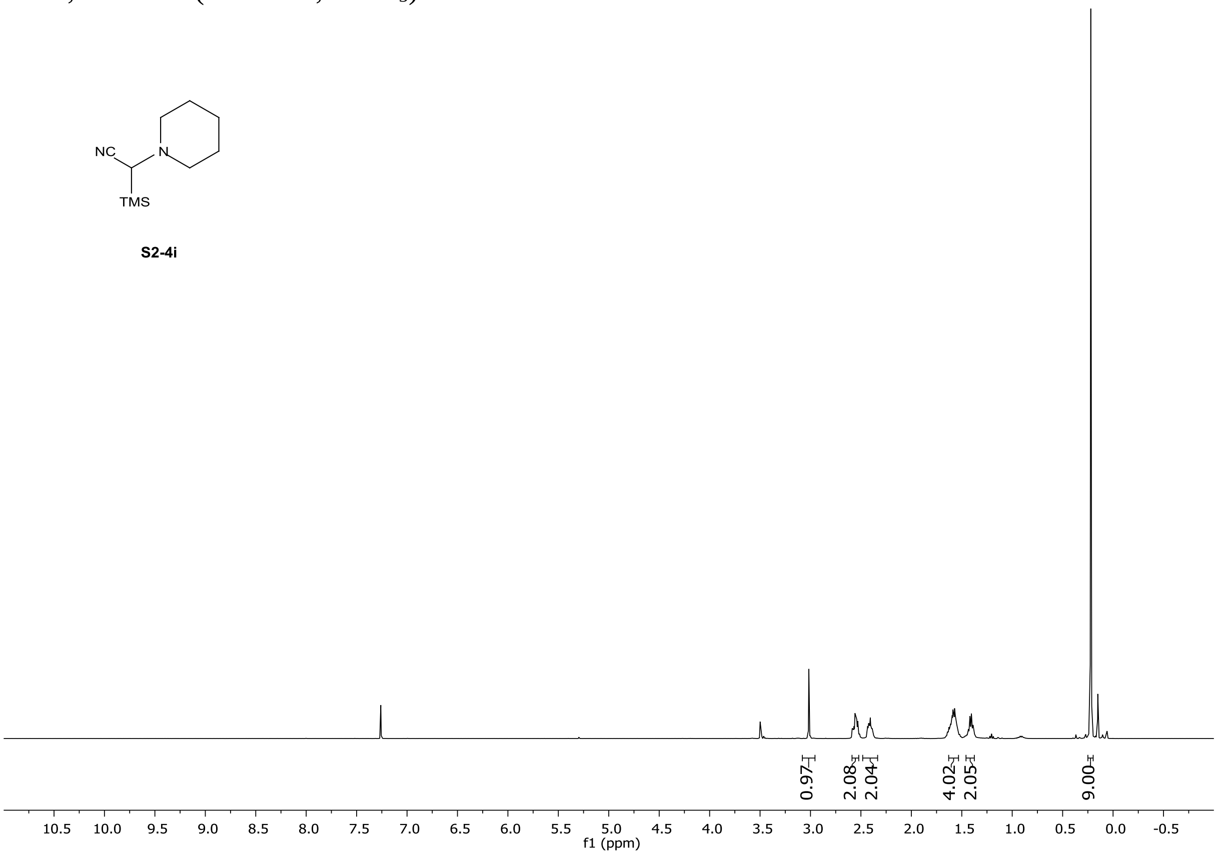

S2-4i, ^{13}C NMR (101 MHz, $CDCl_3$)

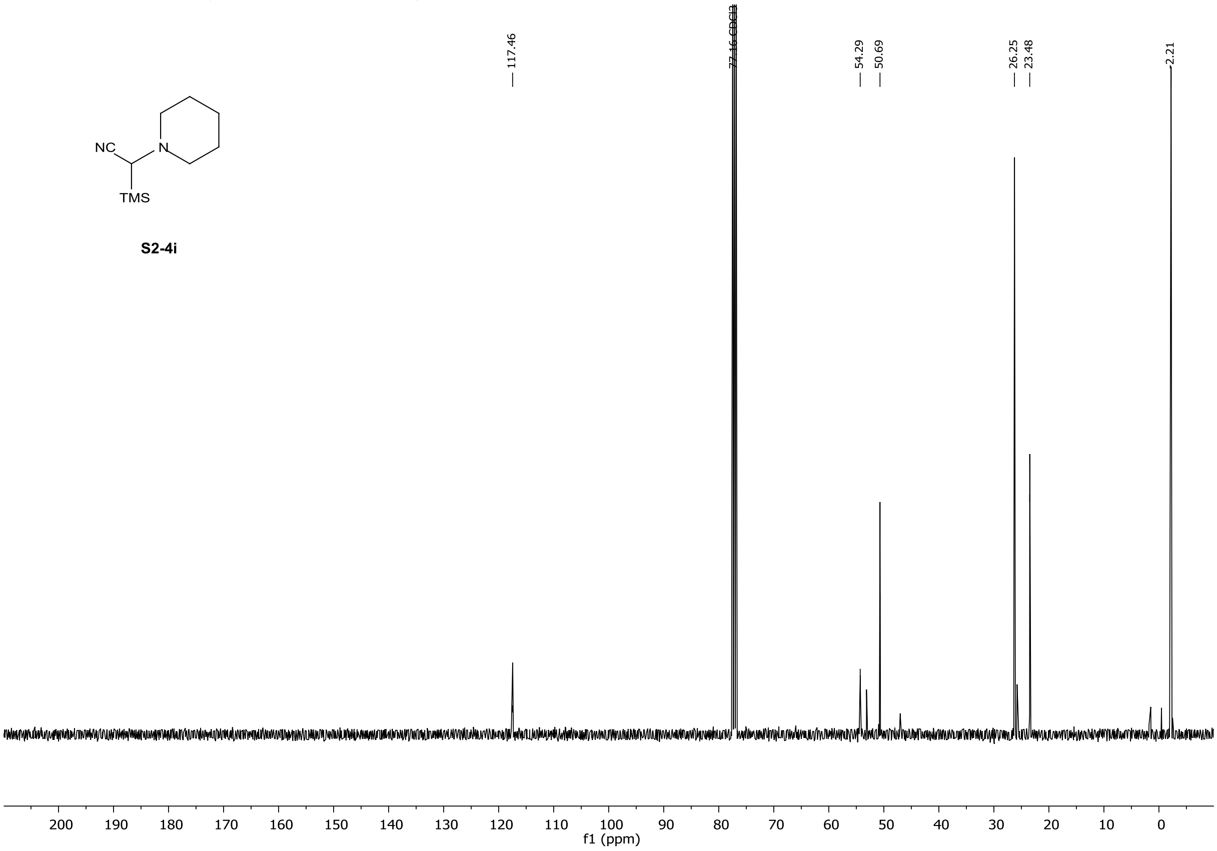

4i, ^{1}H NMR (400 MHz, $CDCl_3$)

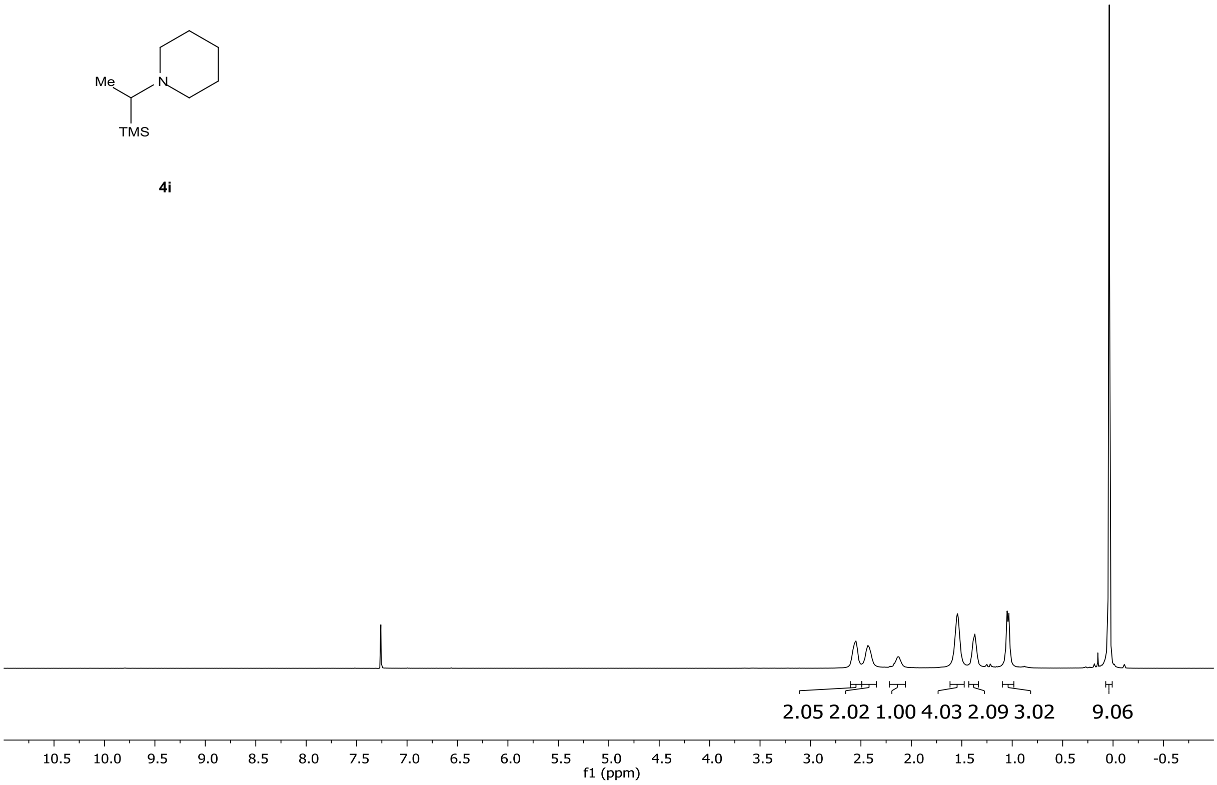

4i, ^{13}C NMR (101 MHz, $CDCl_3$)

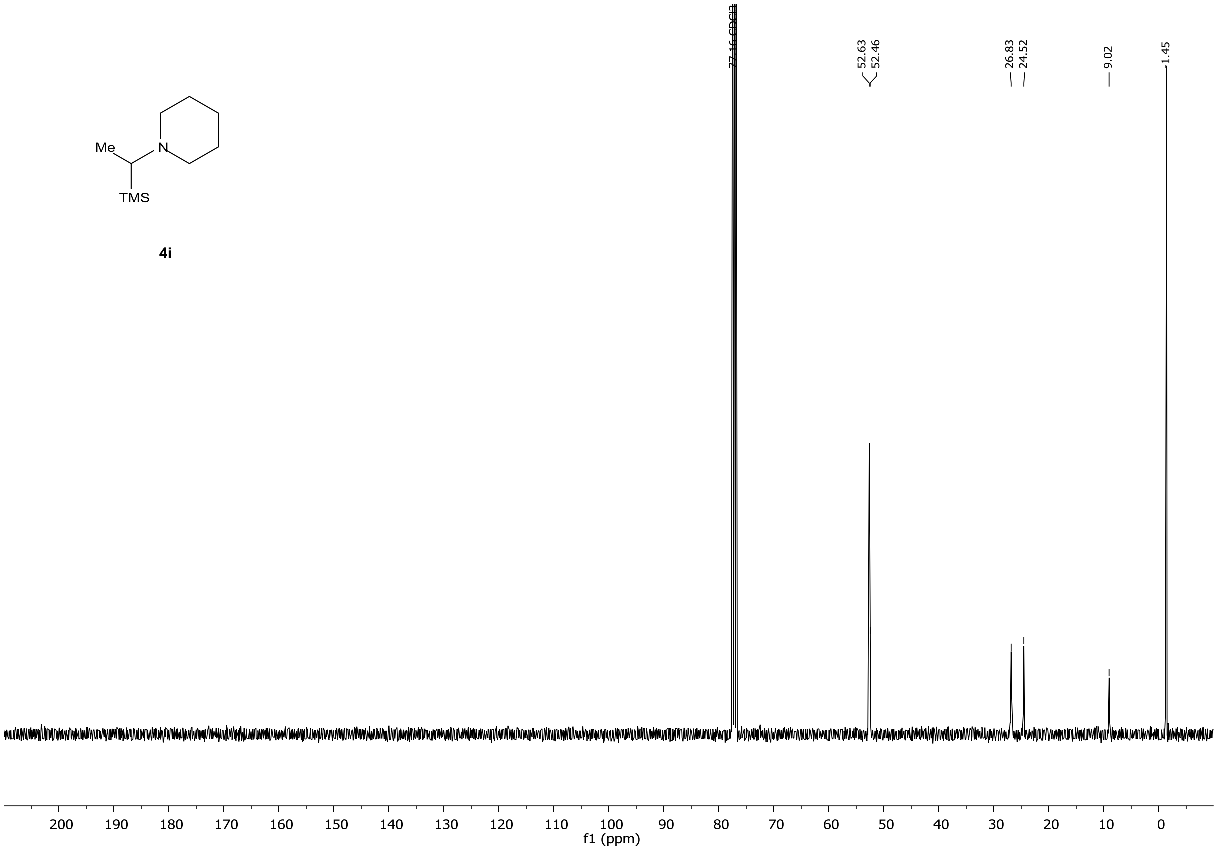

3a, ^{1}H NMR (400 MHz, $CDCl_3$)

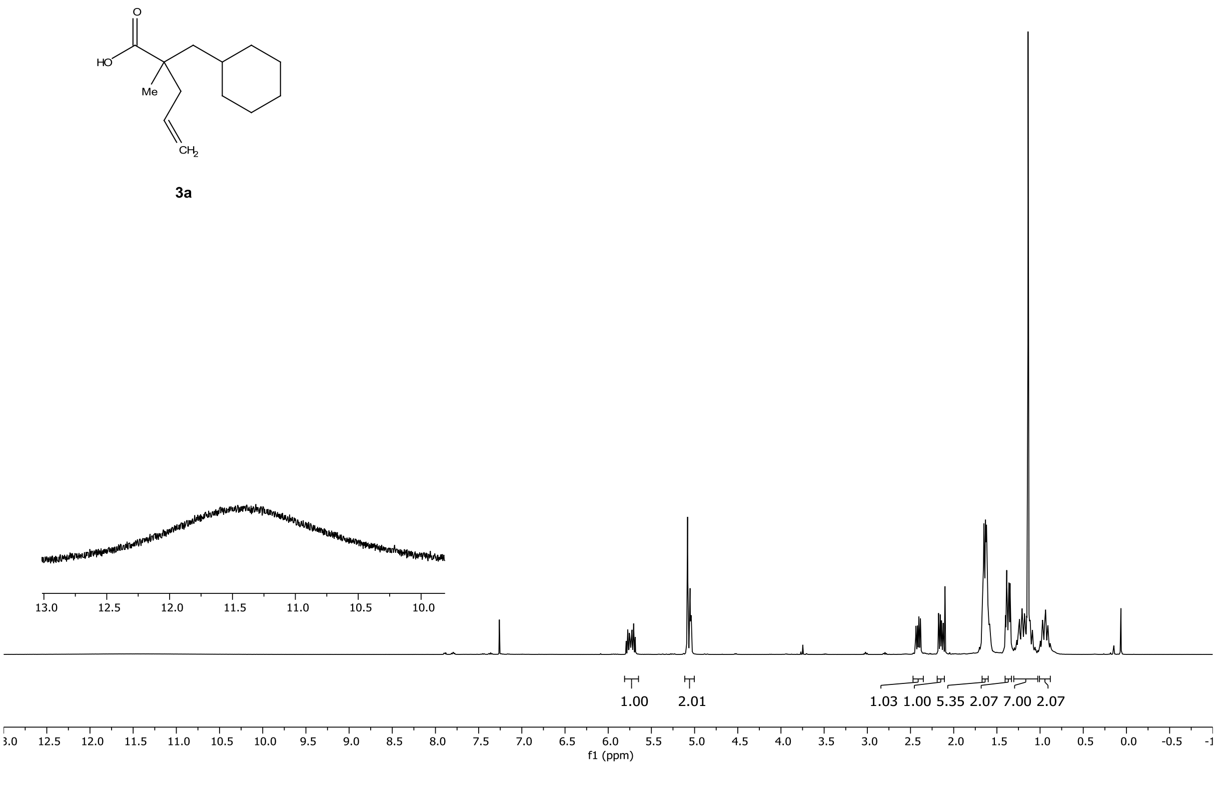

3a, ^{13}C NMR (126 MHz, $CDCl_3$)

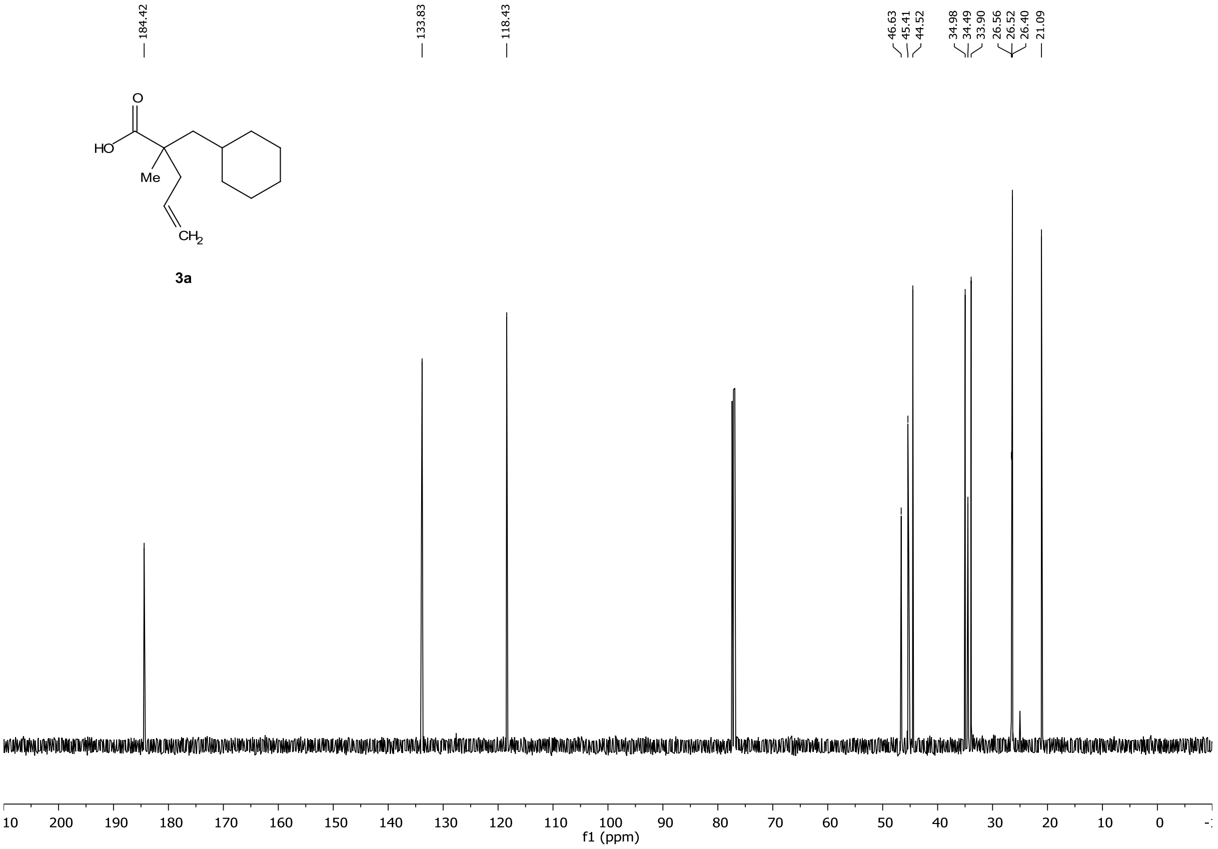

3b, ^{1}H NMR (400 MHz, $CDCl_3$)

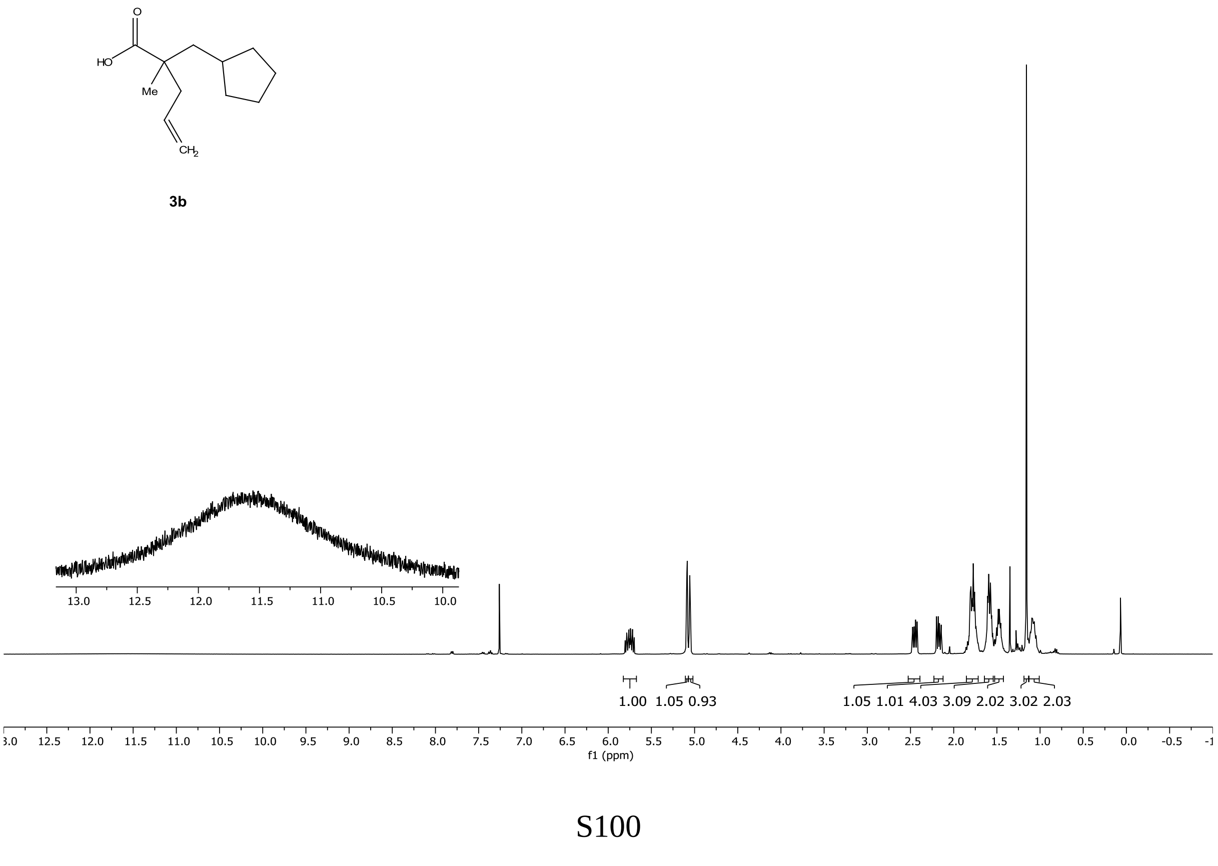

3b, ^{13}C NMR (101 MHz, $CDCl_3$)

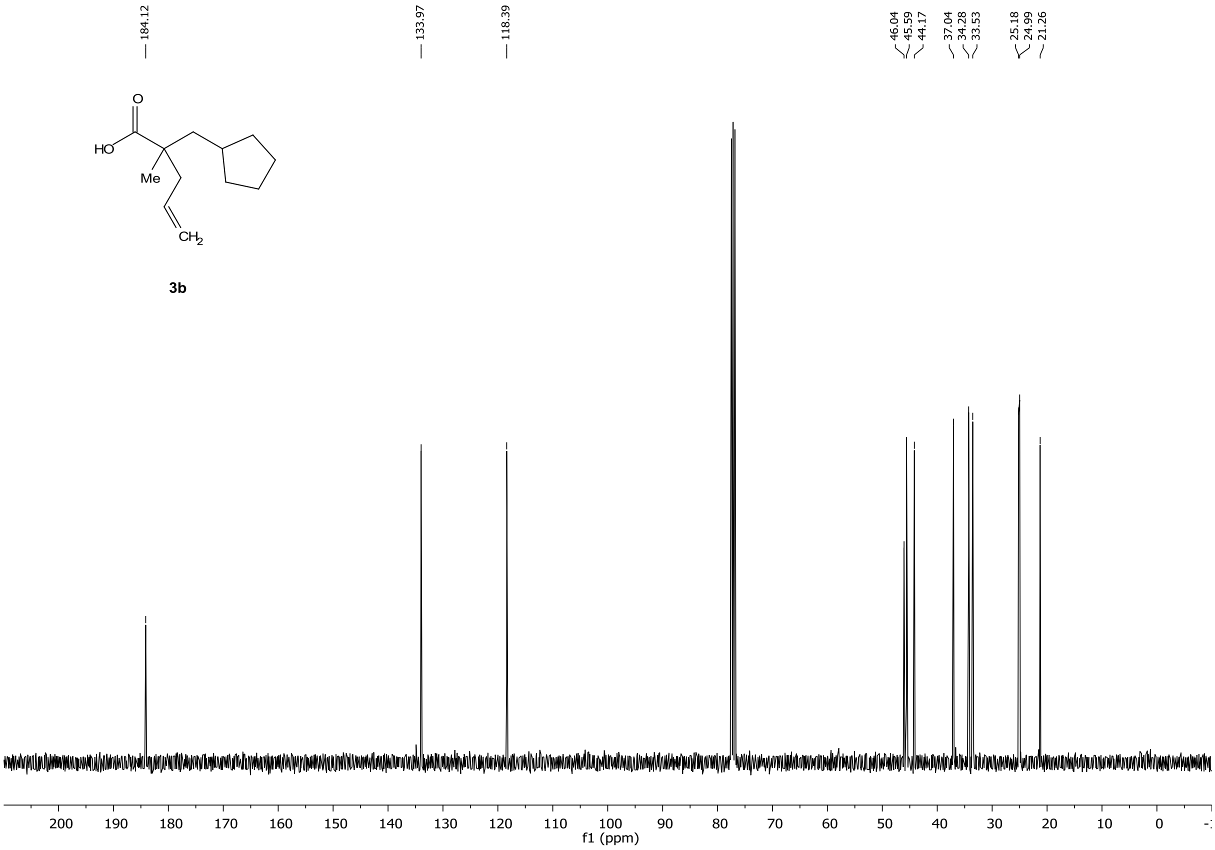

3c, ^{1}H NMR (400 MHz, $CDCl_3$)

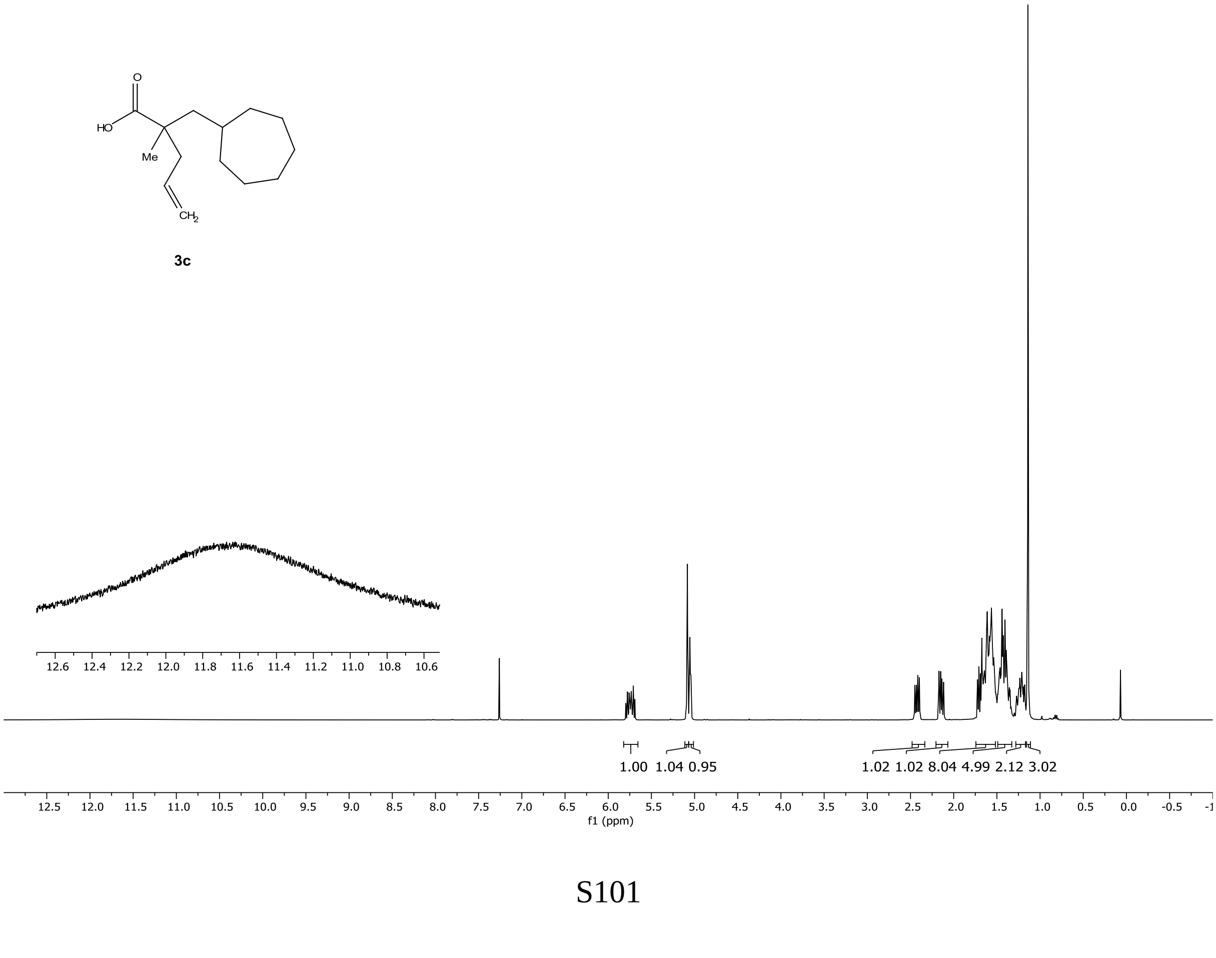

3c, ^{13}C NMR (101 MHz, $CDCl_3$)

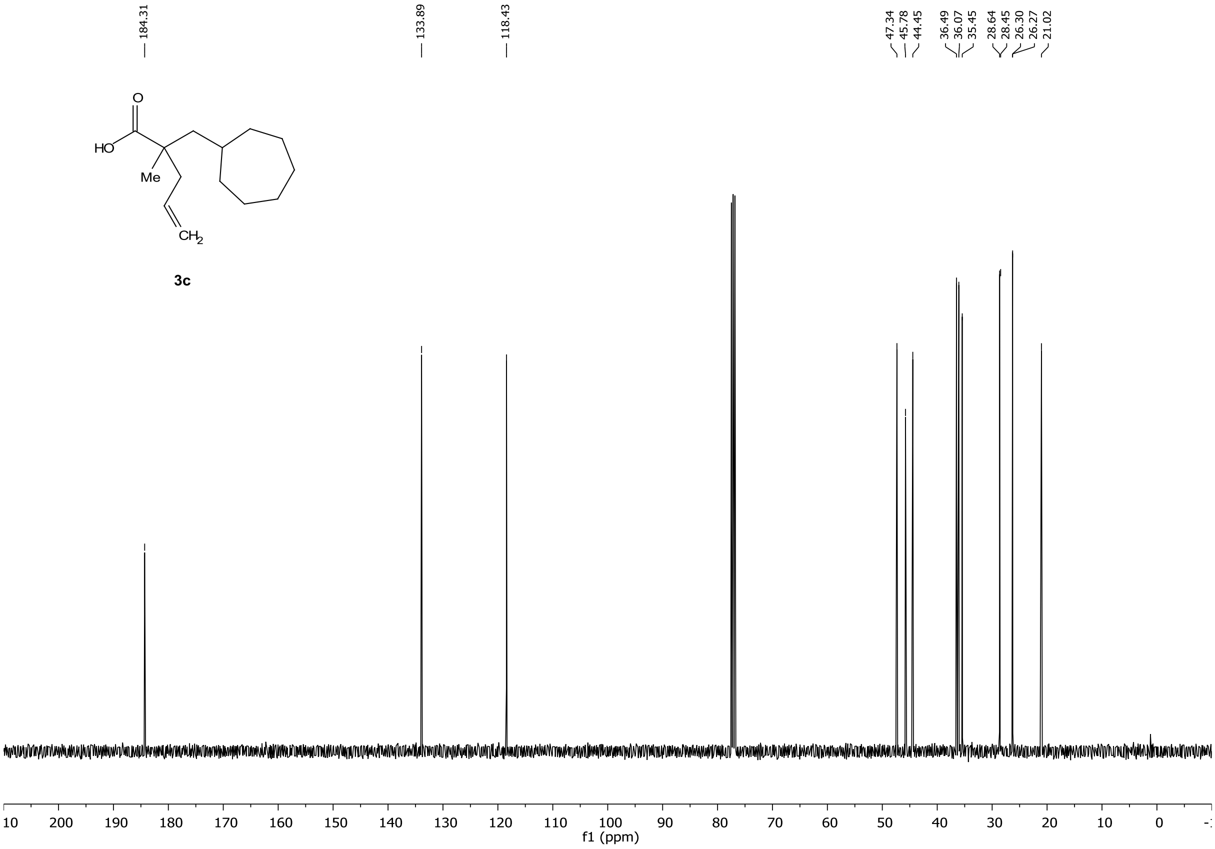

3d, ^{1}H NMR (400 MHz, $CDCl_3$)

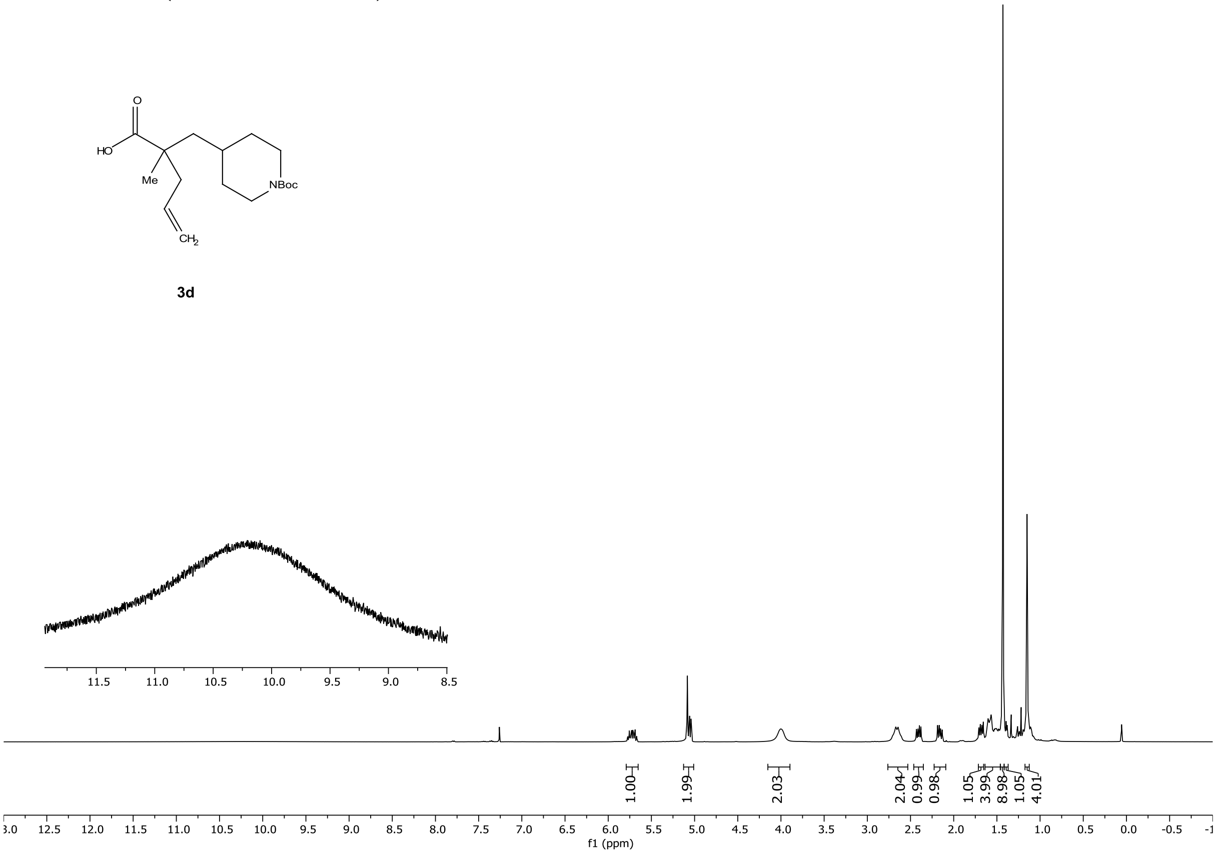

3d, ^{13}C NMR (101 MHz, $CDCl_3$)

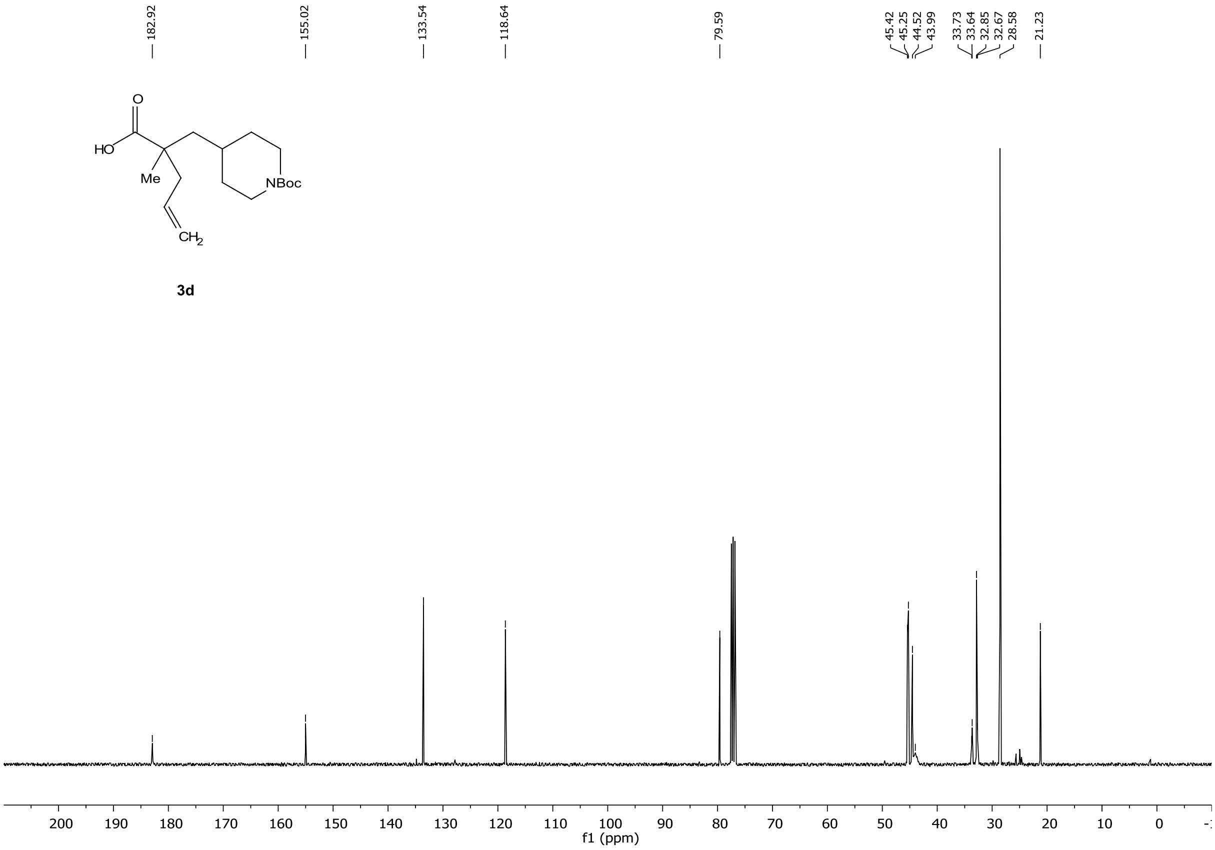

3e, ^{1}H NMR (400 MHz, $CDCl_3$)

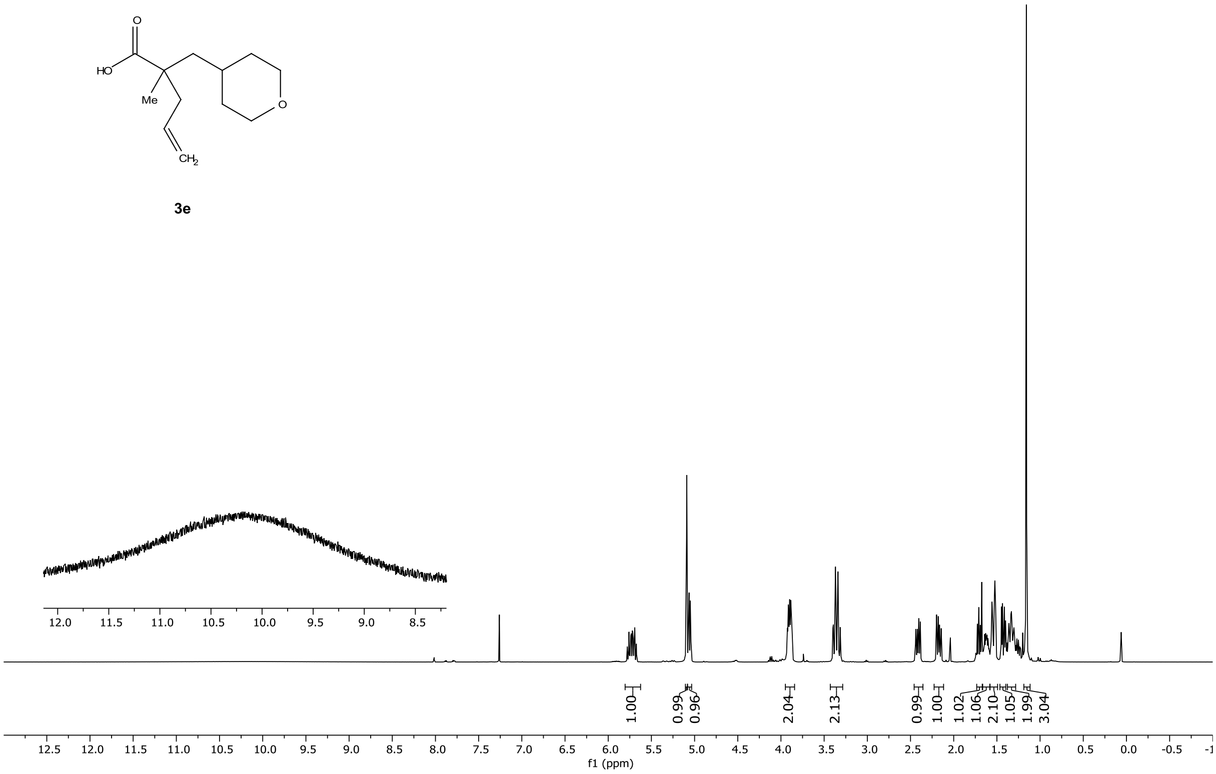

3e, ^{13}C NMR (101 MHz, $CDCl_3$)

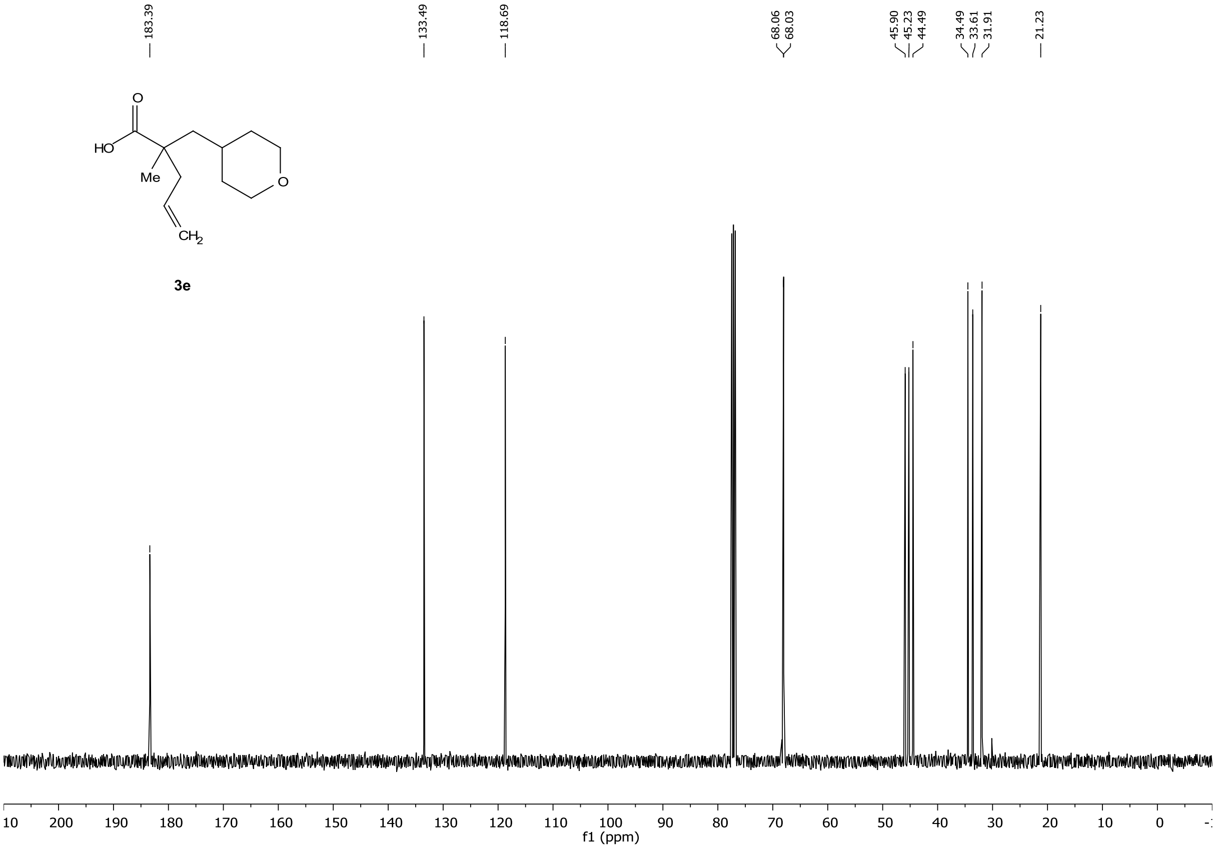

3f, ^{1}H NMR (400 MHz, $CDCl_3$)

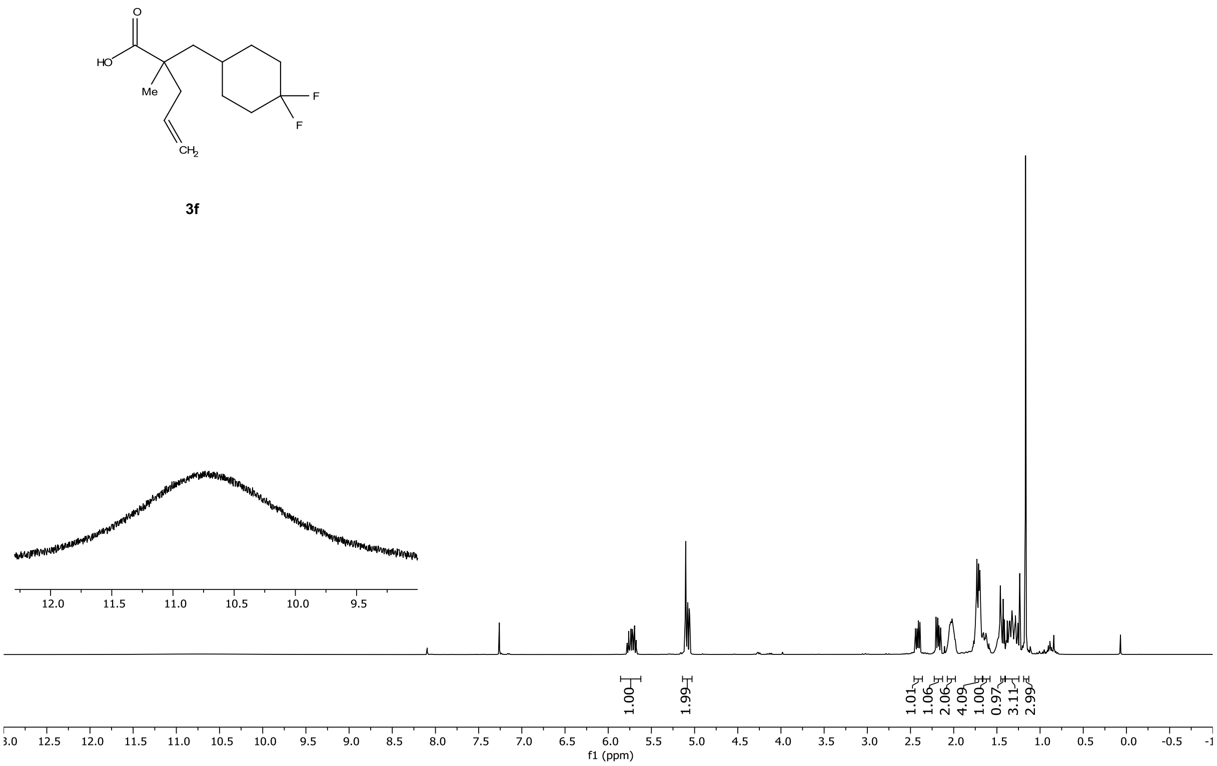

3f, ^{13}C NMR (101 MHz, $CDCl_3$)

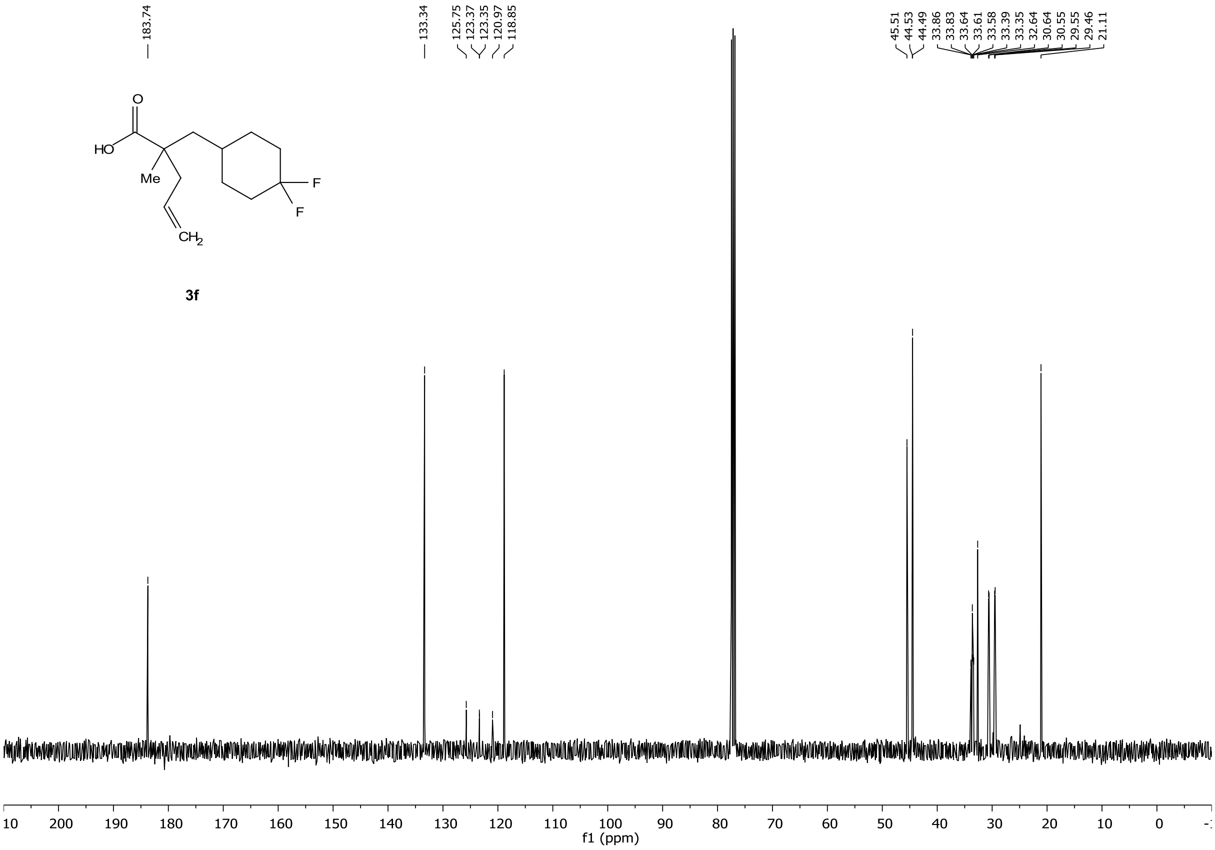

3f, ^{19}F NMR (376 MHz, CDCl3)

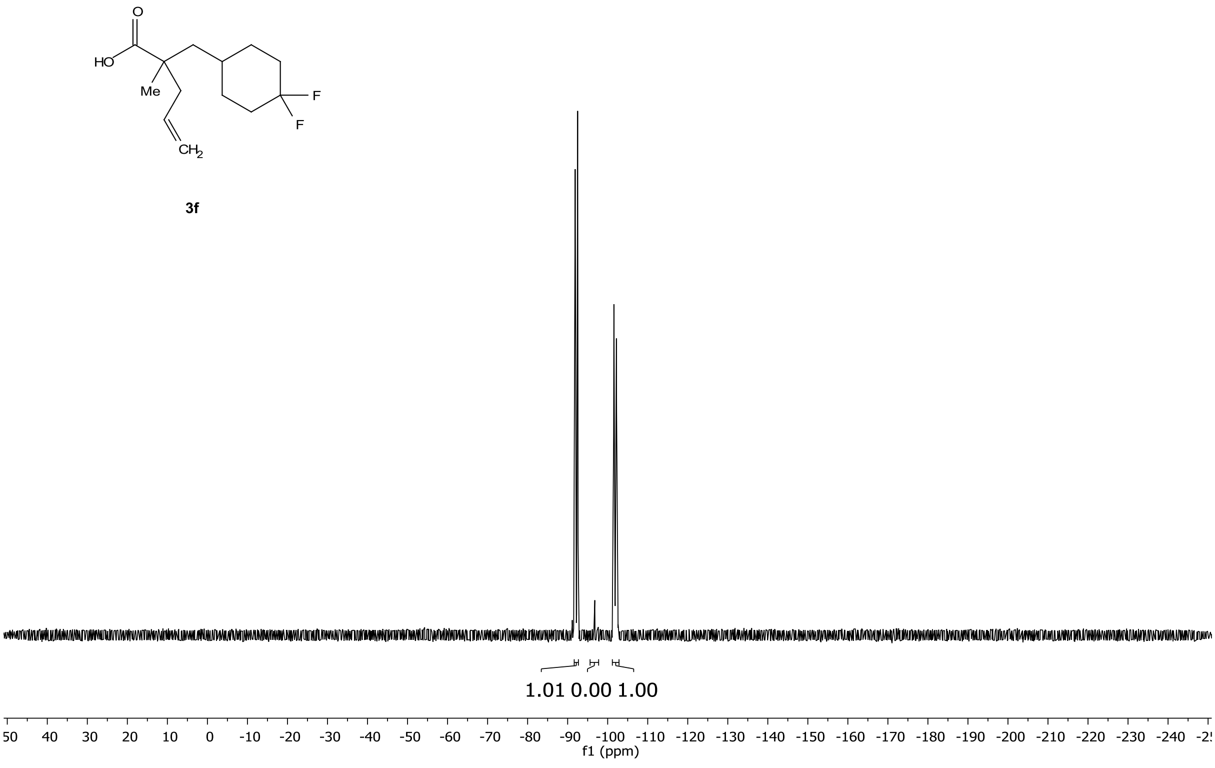

3g, ^{1}H NMR (400 MHz, $CDCl_3$)

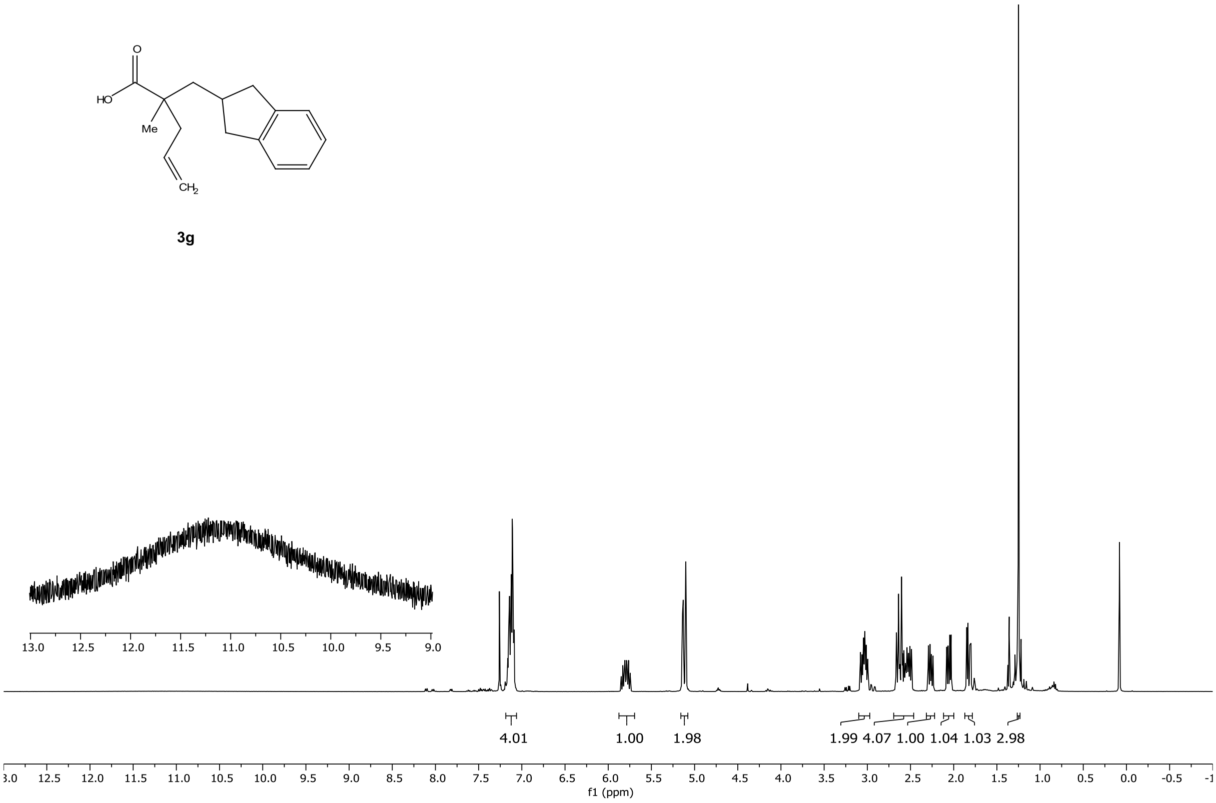

3g, ^{13}C NMR (101 MHz, $CDCl_3$)

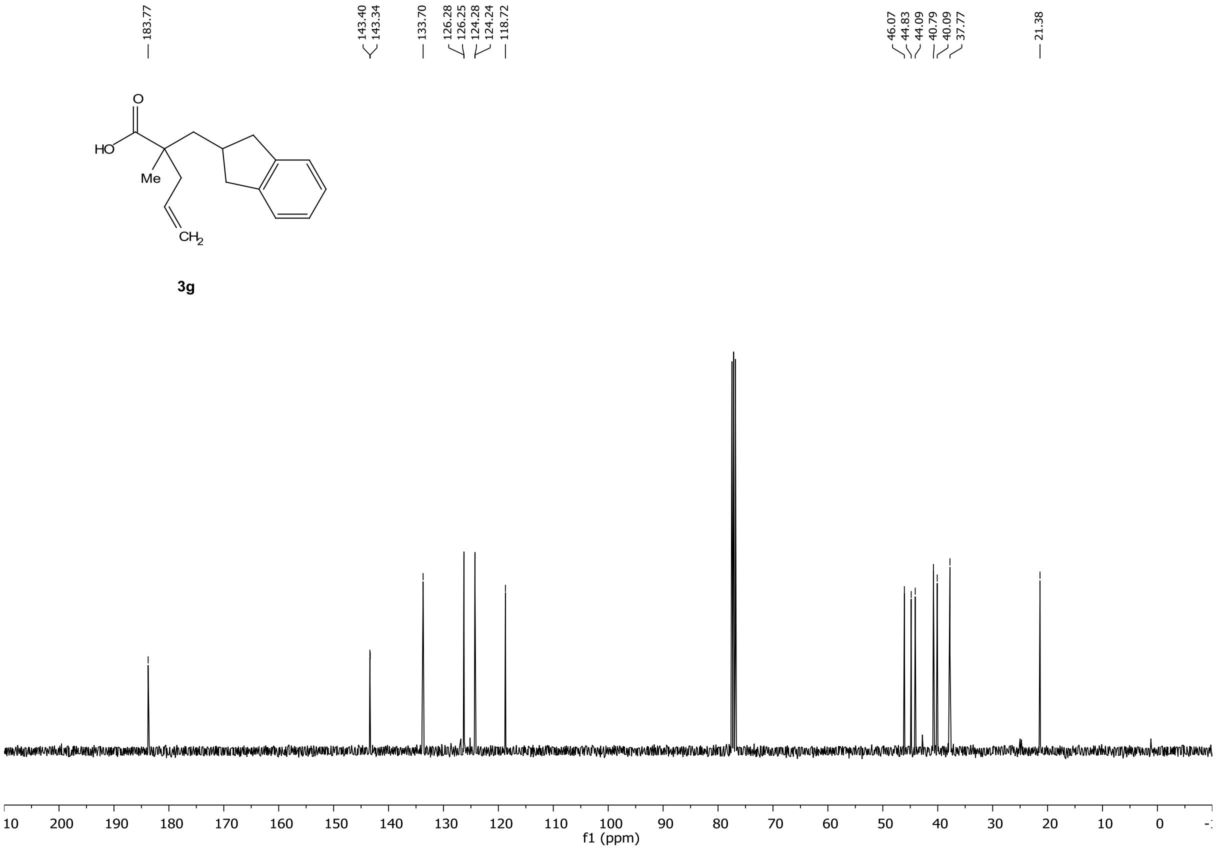

3h, ^{1}H NMR (300 MHz, $CDCl_3$)

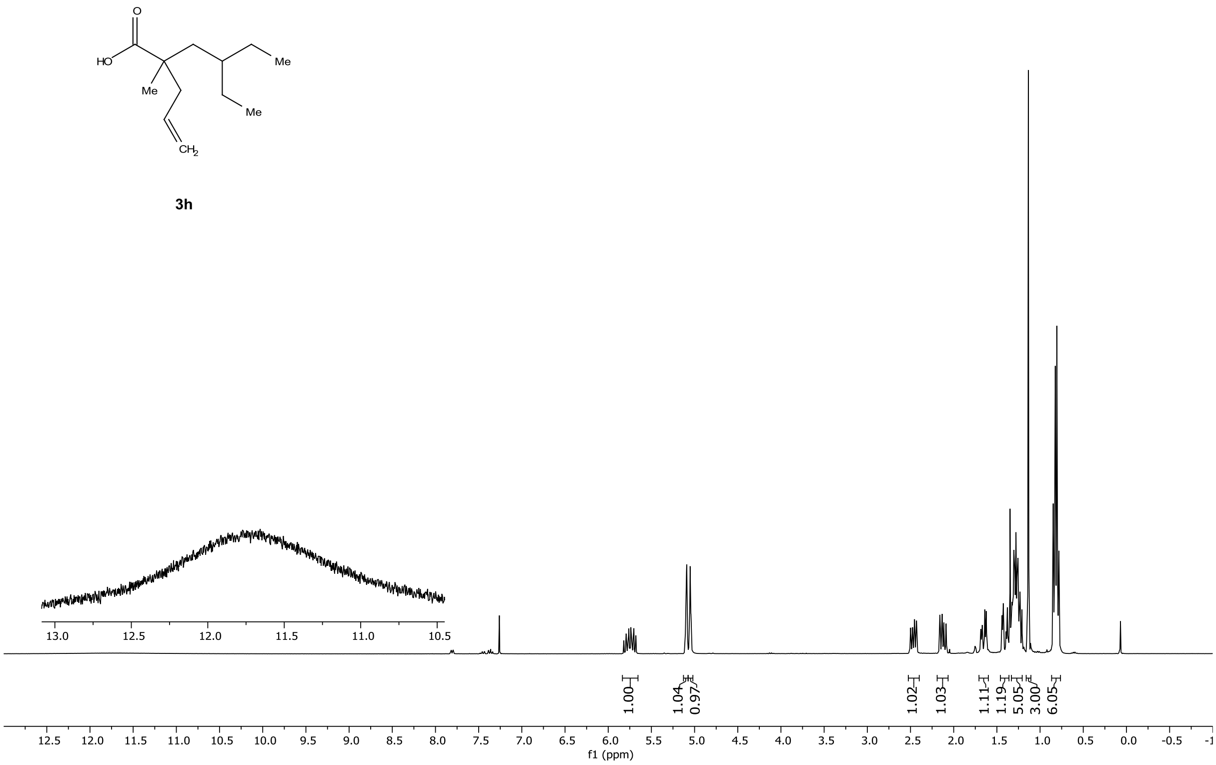

3h, ^{13}C NMR (76 MHz, $CDCl_3$)

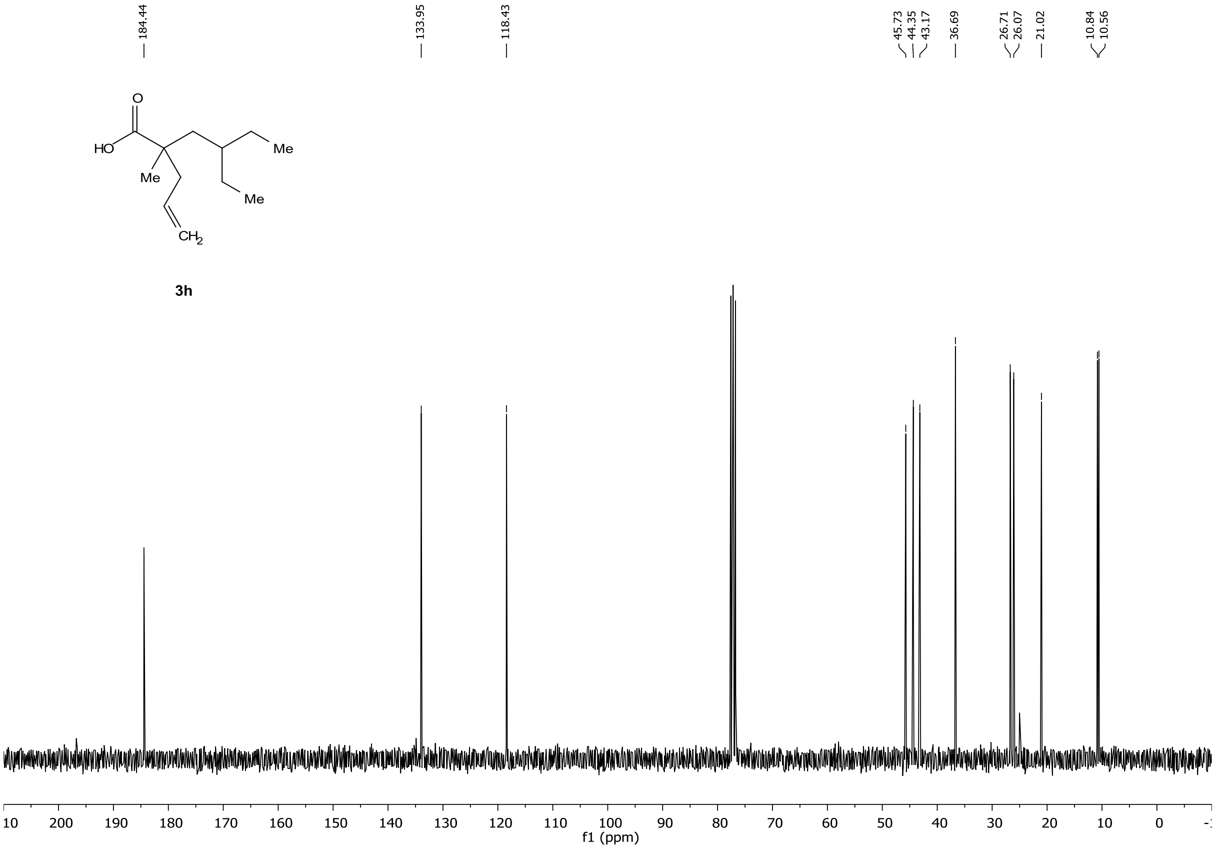

3i, ^{1}H NMR (400 MHz, $CDCl_3$)

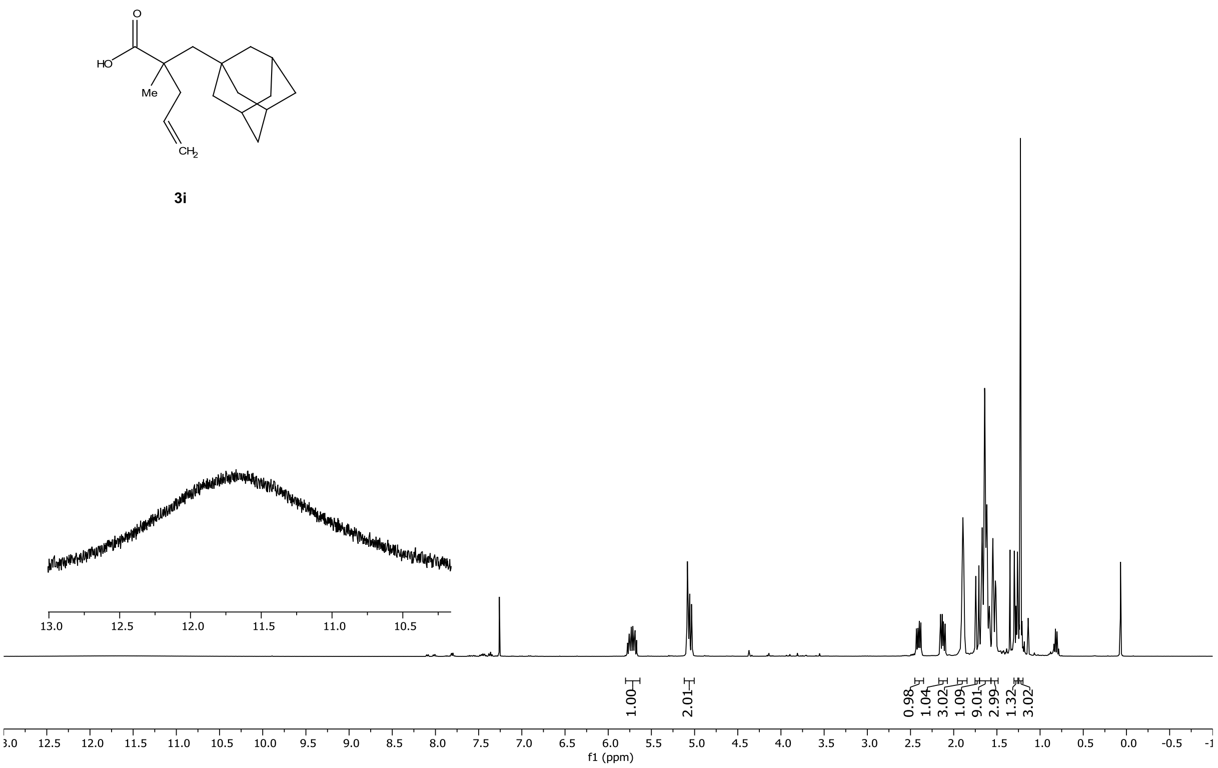

3i, ^{13}C NMR (76 MHz, $CDCl_3$)

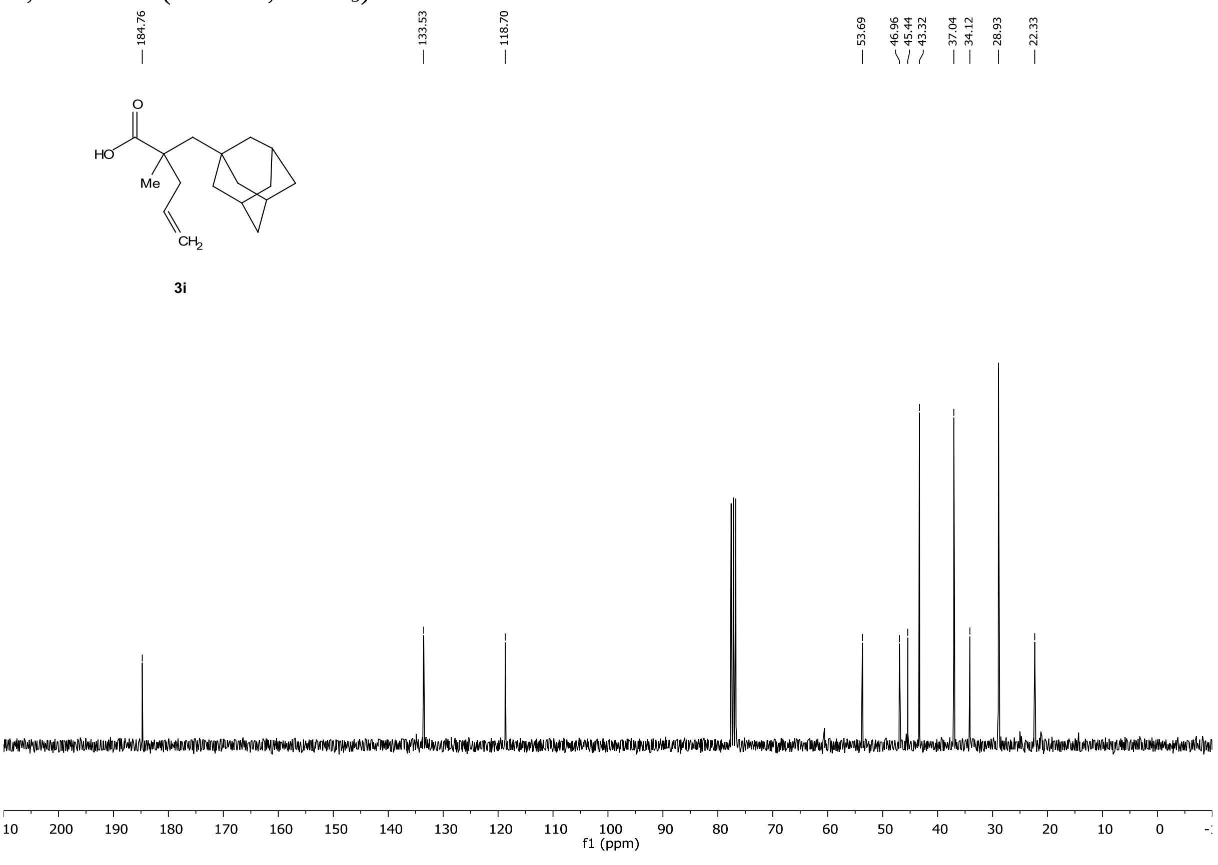

3j, ^{1}H NMR (400 MHz, $CDCl_3$)

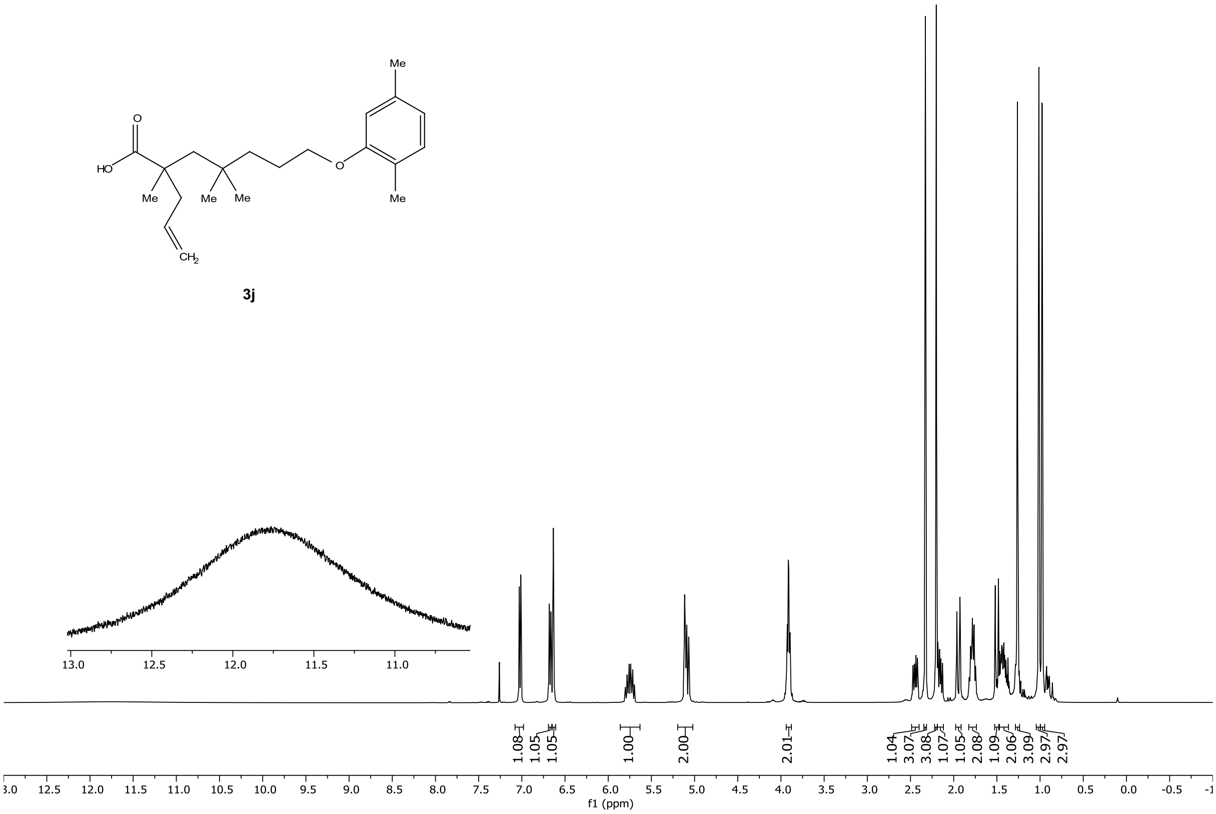

3j, ^{13}C NMR (101 MHz, $CDCl_3$)

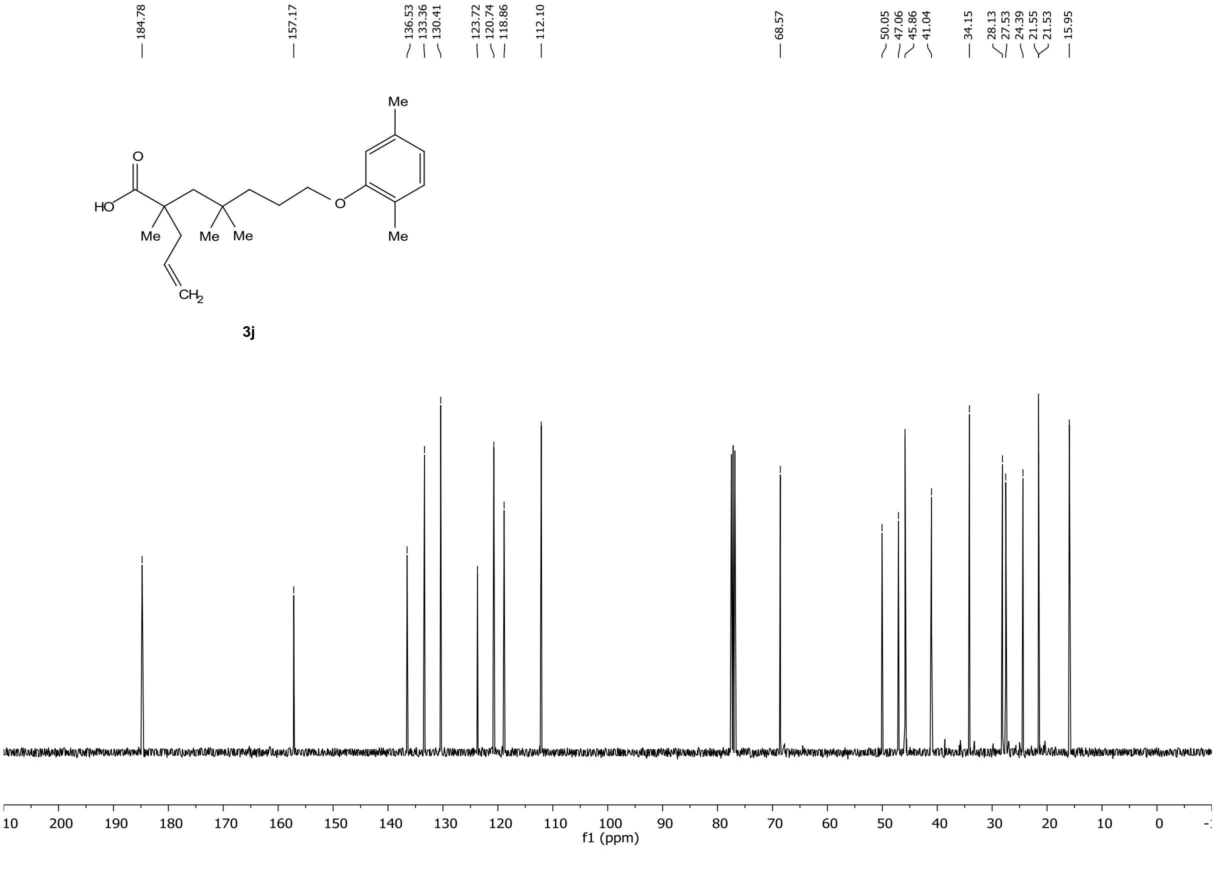

3k, ^{1}H NMR (400 MHz, $CDCl_3$)

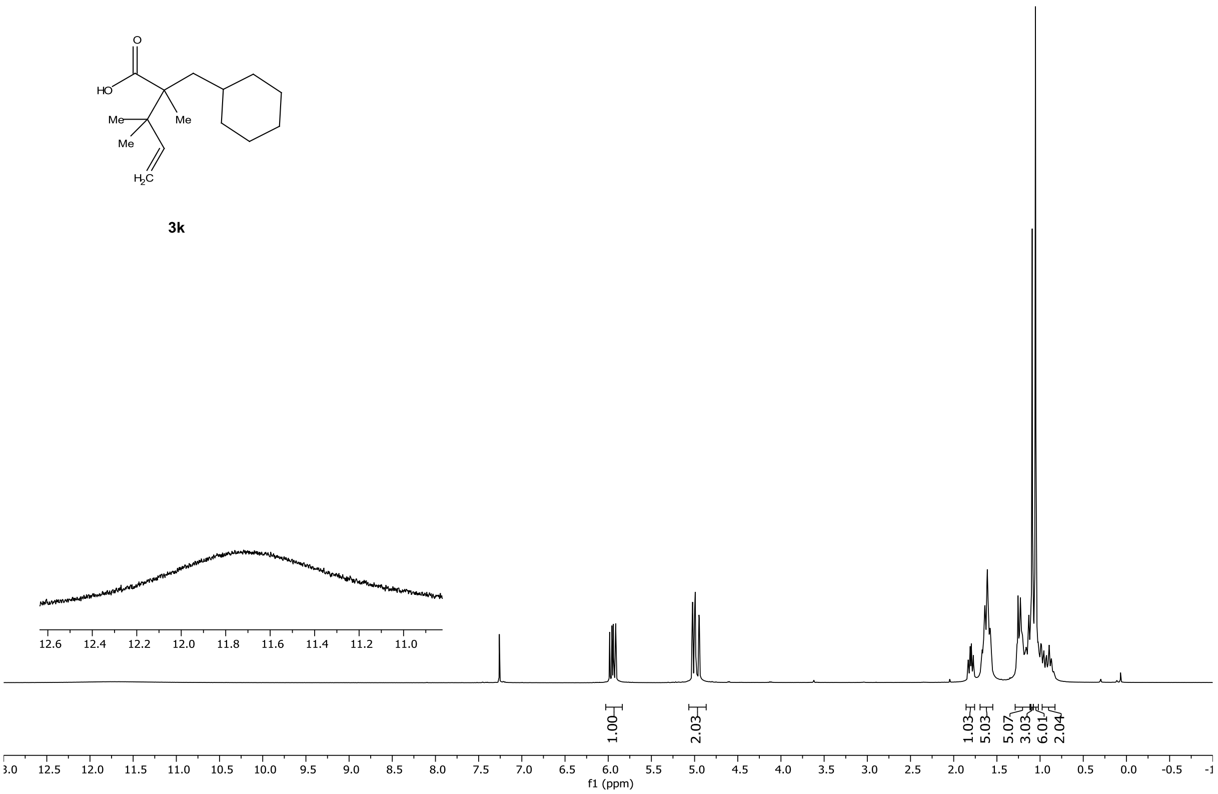

3k, ^{13}C NMR (101 MHz, $CDCl_3$)

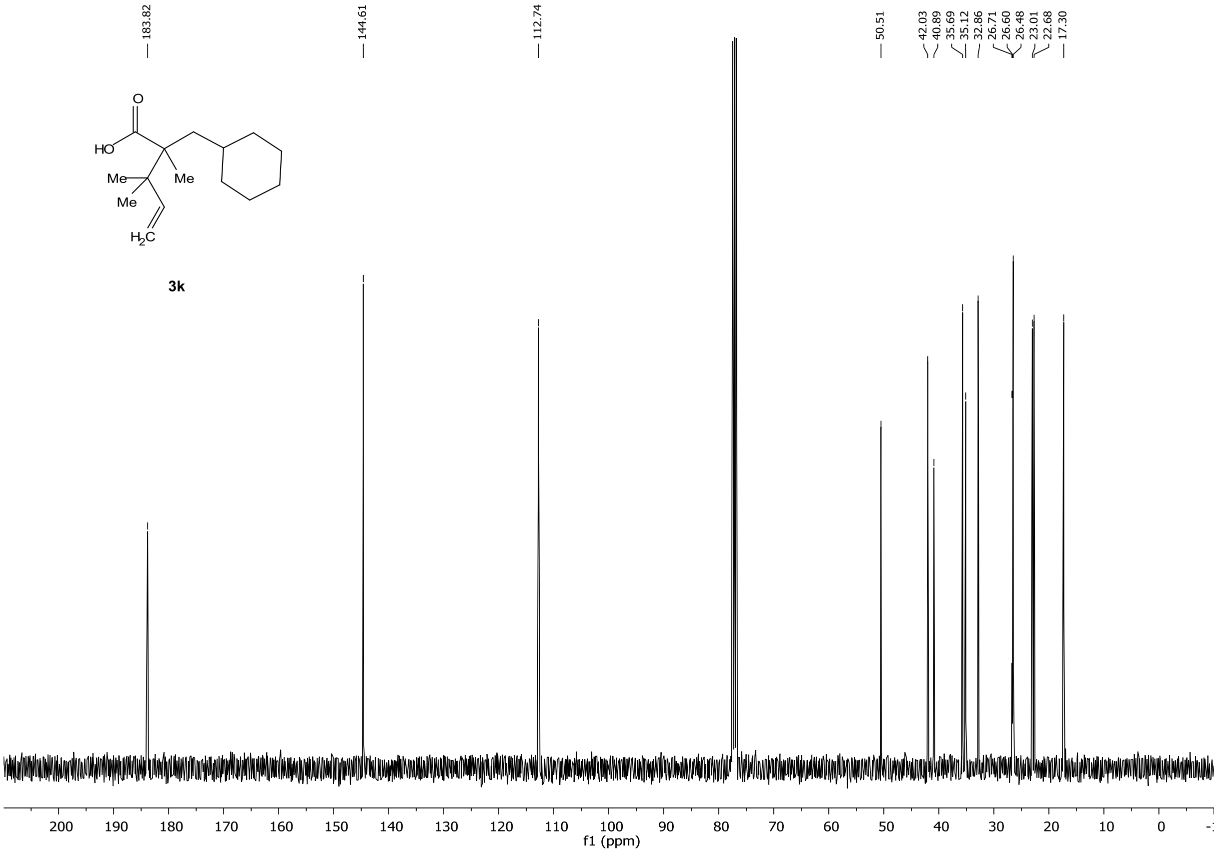

3l, ^{1}H NMR (400 MHz, $CDCl_3$)

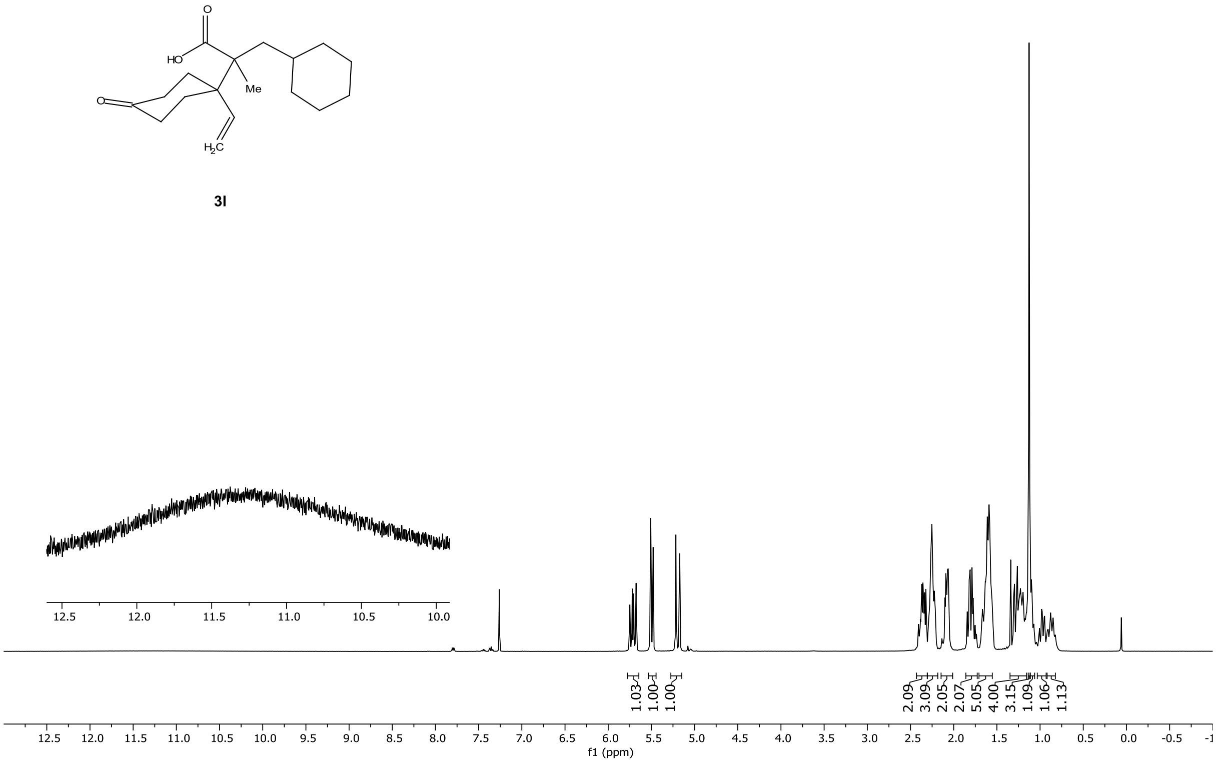

3l, ^{13}C NMR (101 MHz, $CDCl_3$)

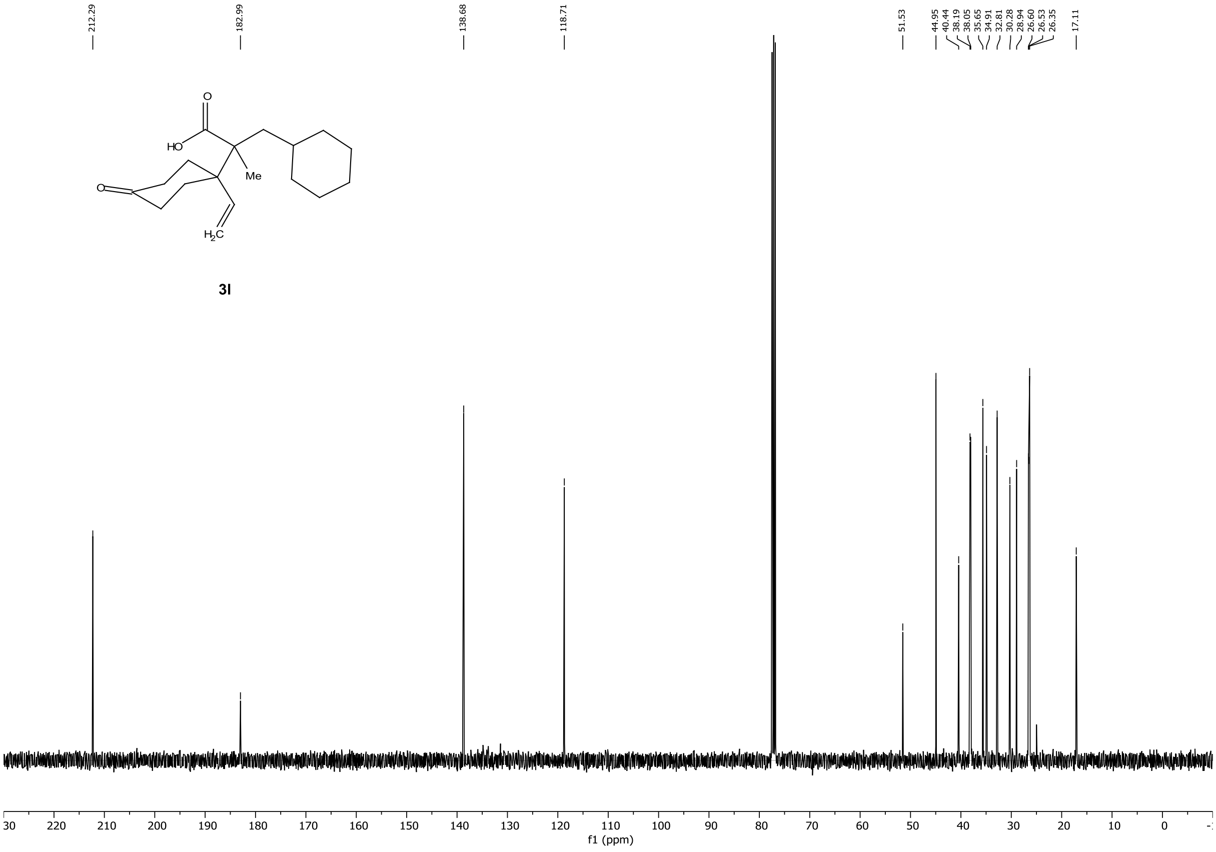

3m, ^{1}H NMR (400 MHz, $CDCl_3$)

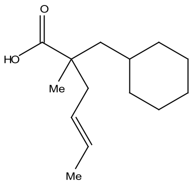

3m

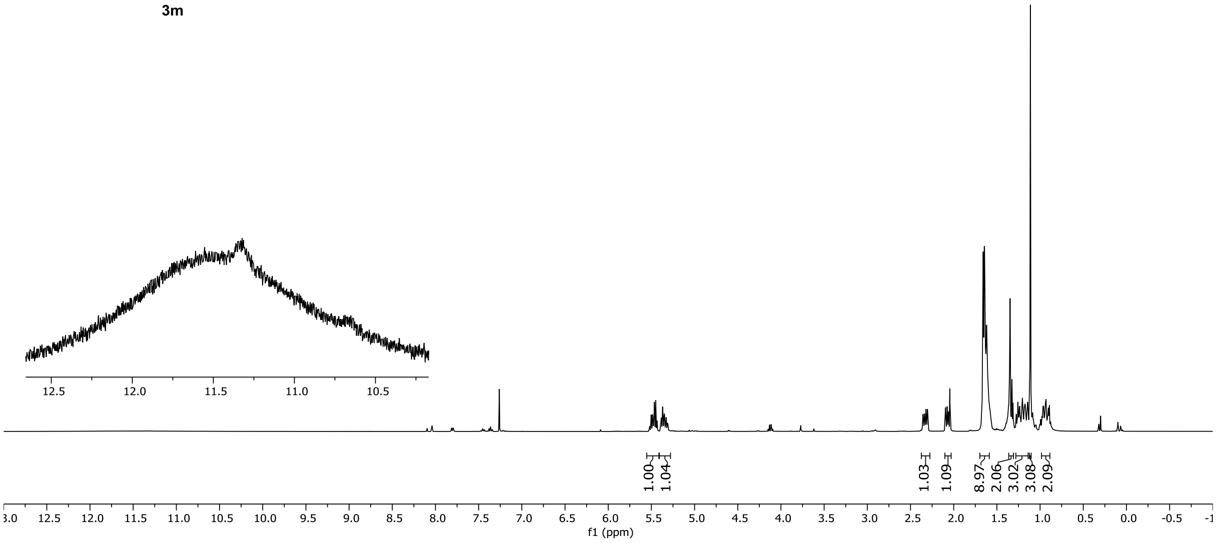

3m, ^{13}C NMR (101 MHz, $CDCl_3$)

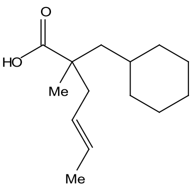

3m

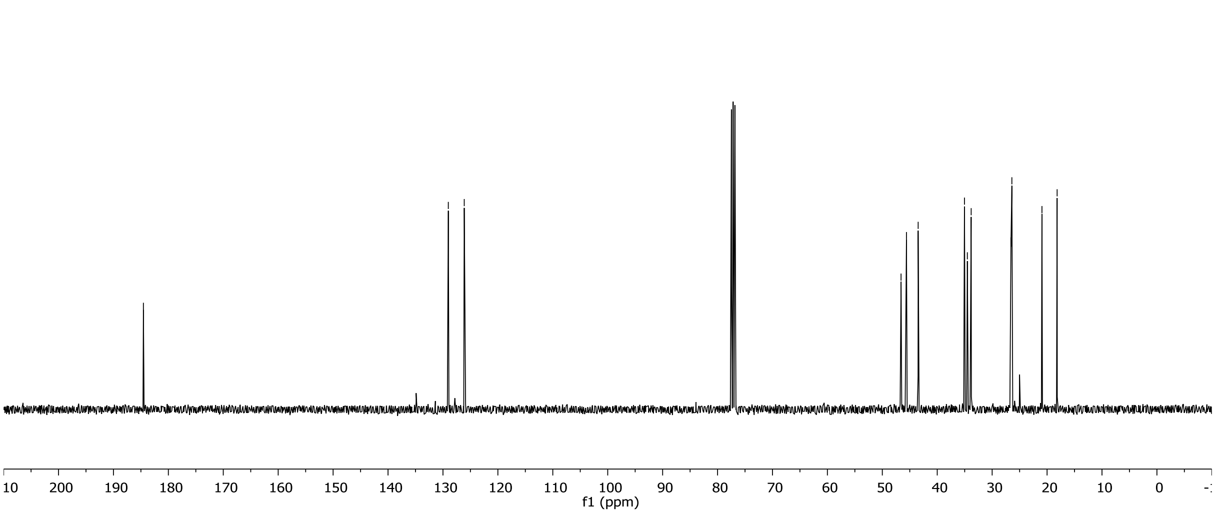

3n, ^{1}H NMR (300 MHz, $CDCl_3$)

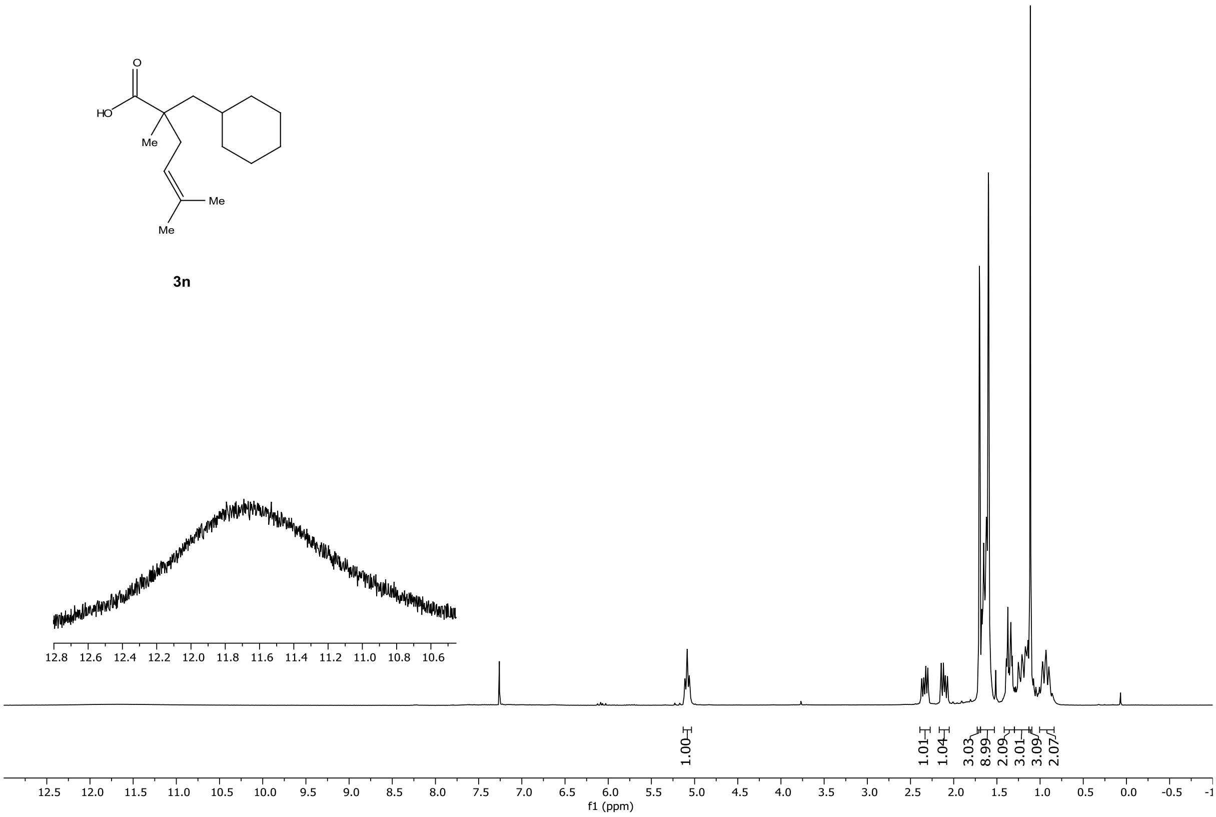

3n, ^{13}C NMR (75 MHz, $CDCl_3$)

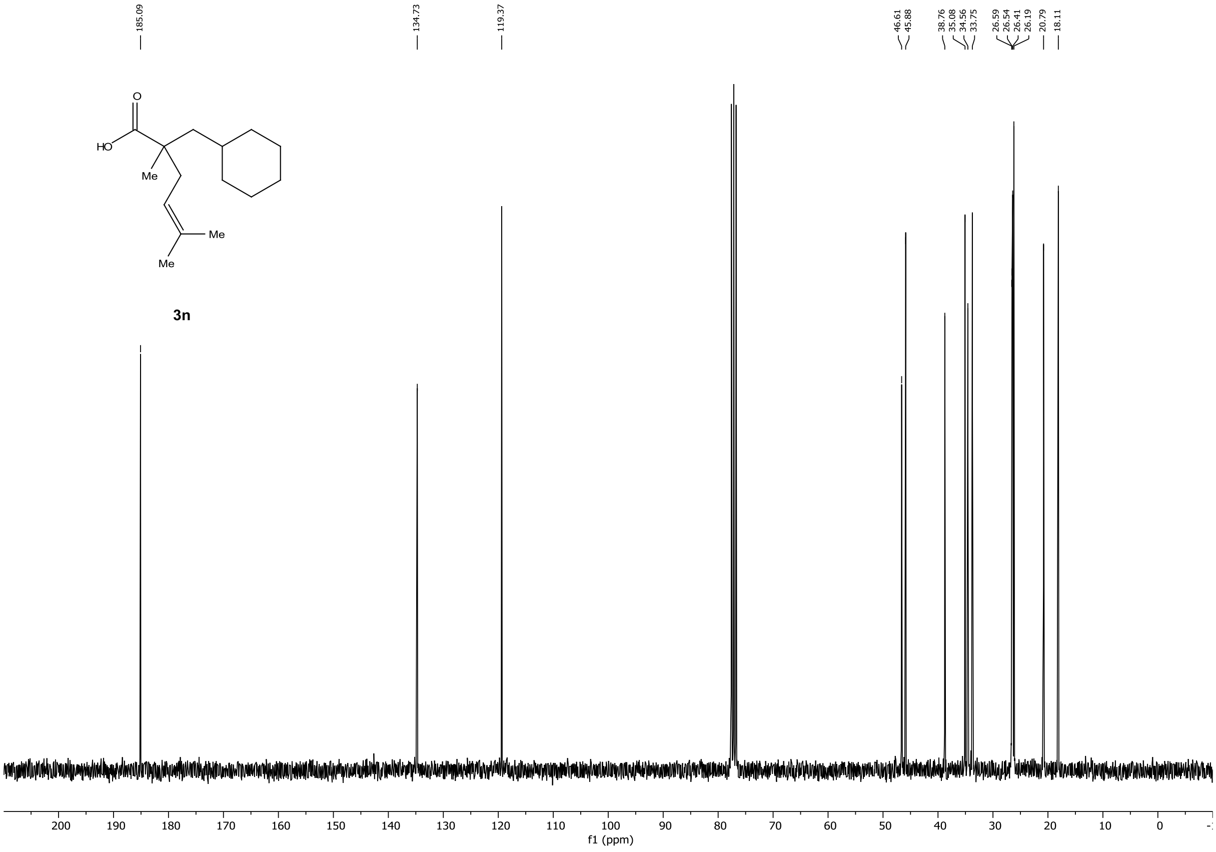

3o, ^{1}H NMR (400 MHz, $CDCl_3$)

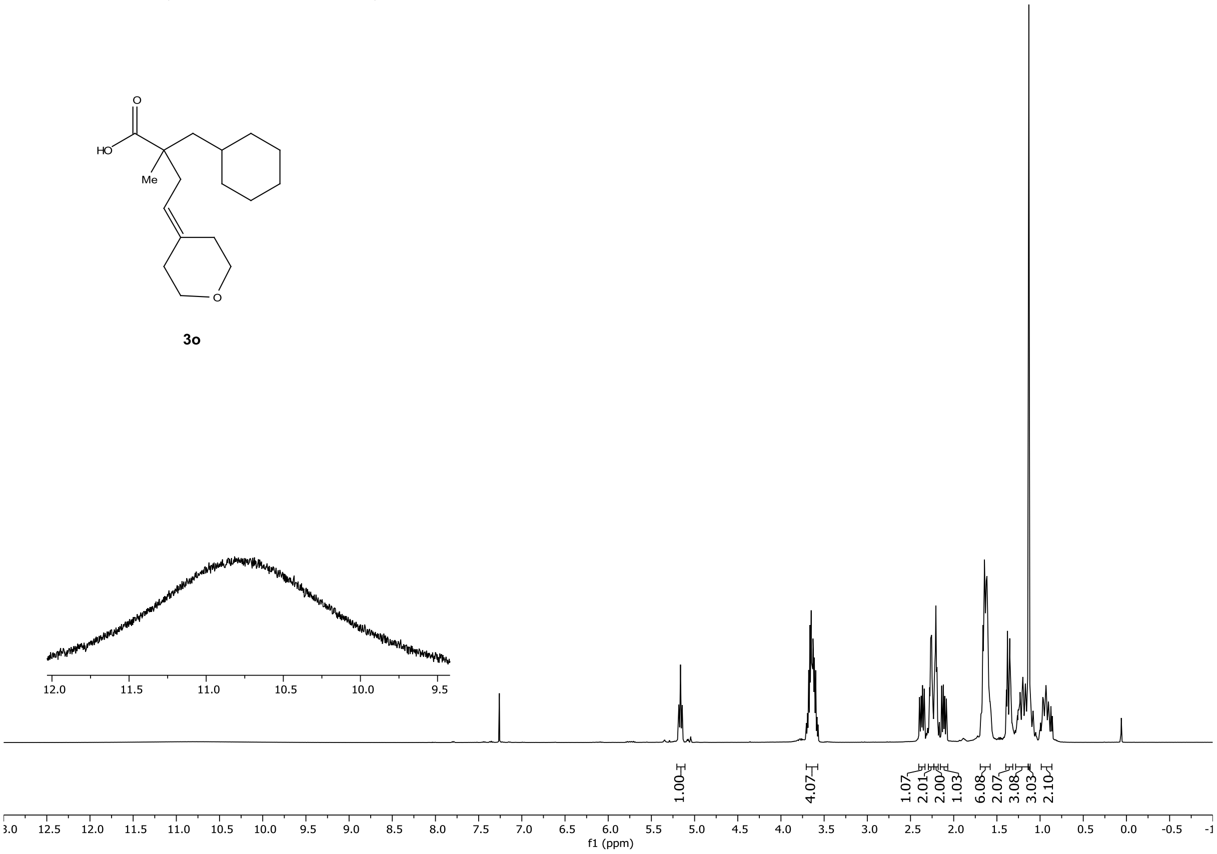

3o, ^{13}C NMR (101 MHz, $CDCl_3$)

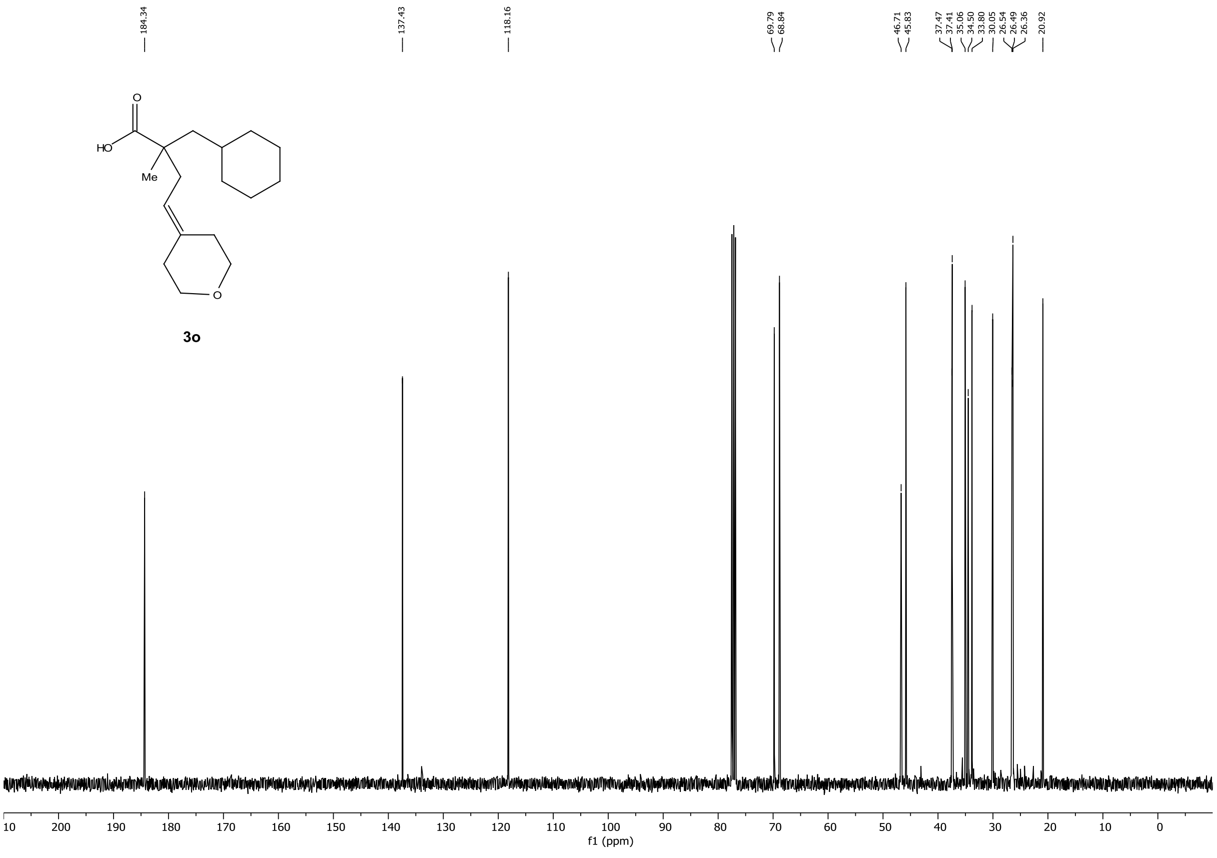

3p, ^{1}H NMR (400 MHz, $CDCl_3$)

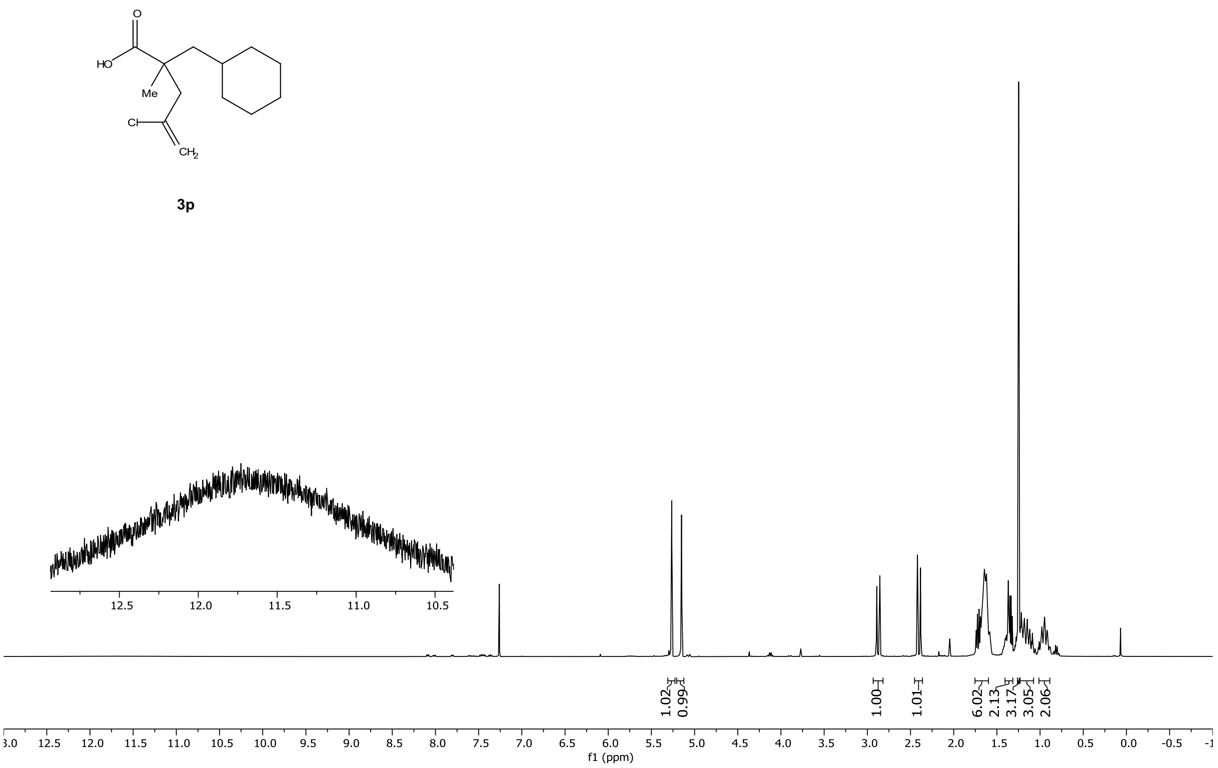

3p, ^{13}C NMR (101 MHz, $CDCl_3$)

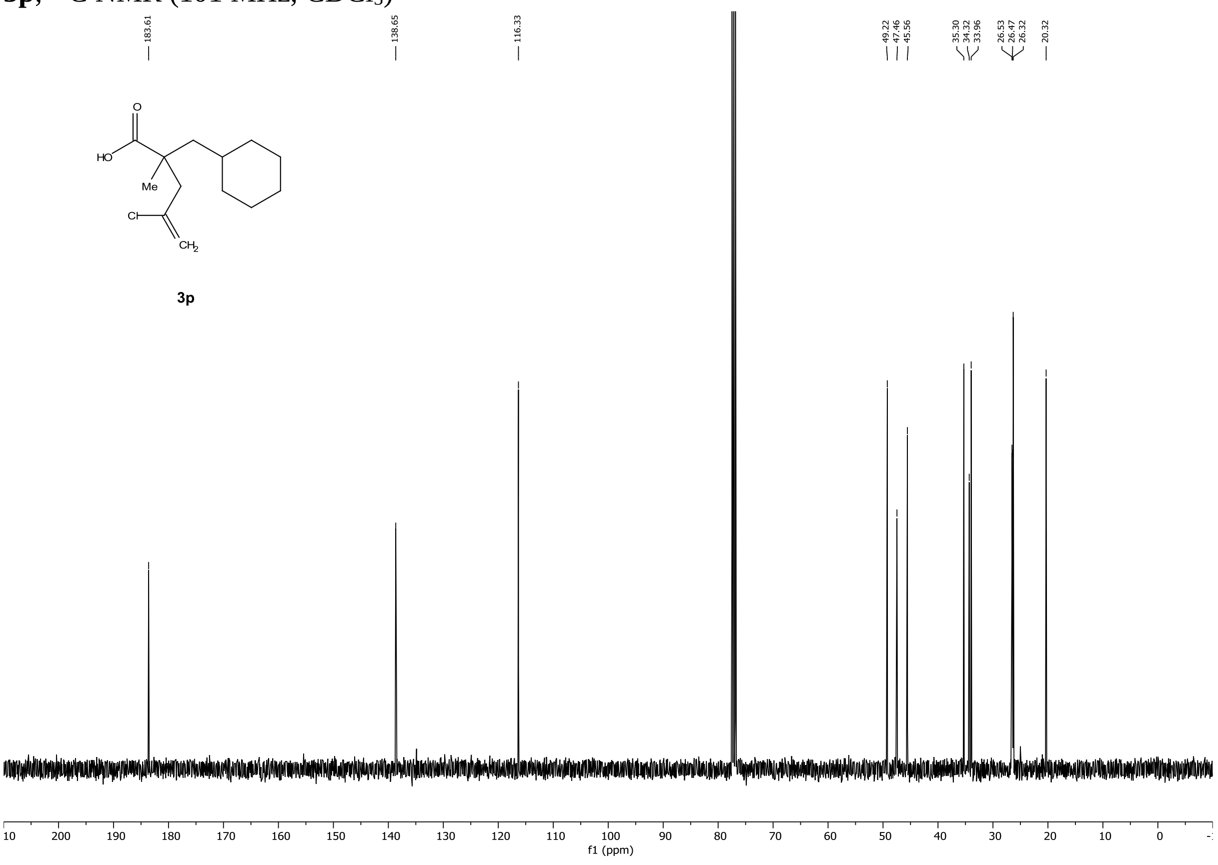

3q, ^{1}H NMR (400 MHz, $CDCl_3$, both diastereomers)

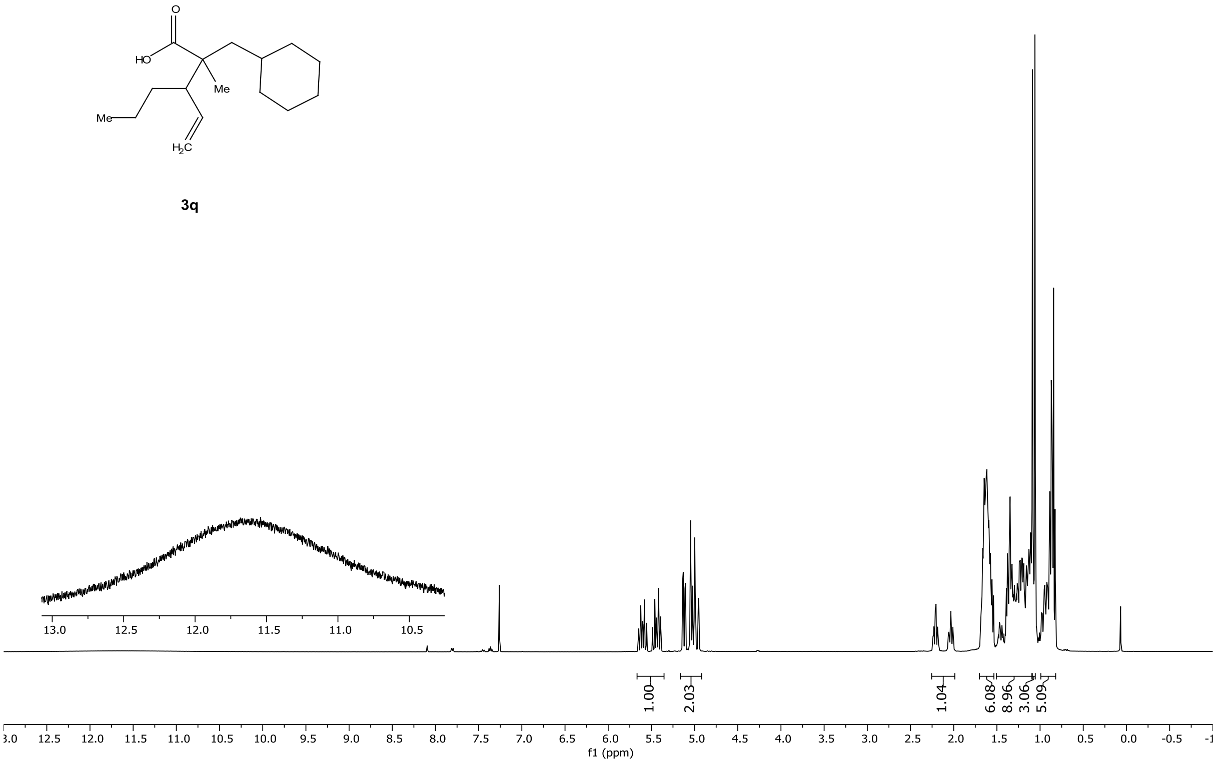

3q, ^{13}C NMR (101 MHz, $CDCl_3$, both diastereomers)

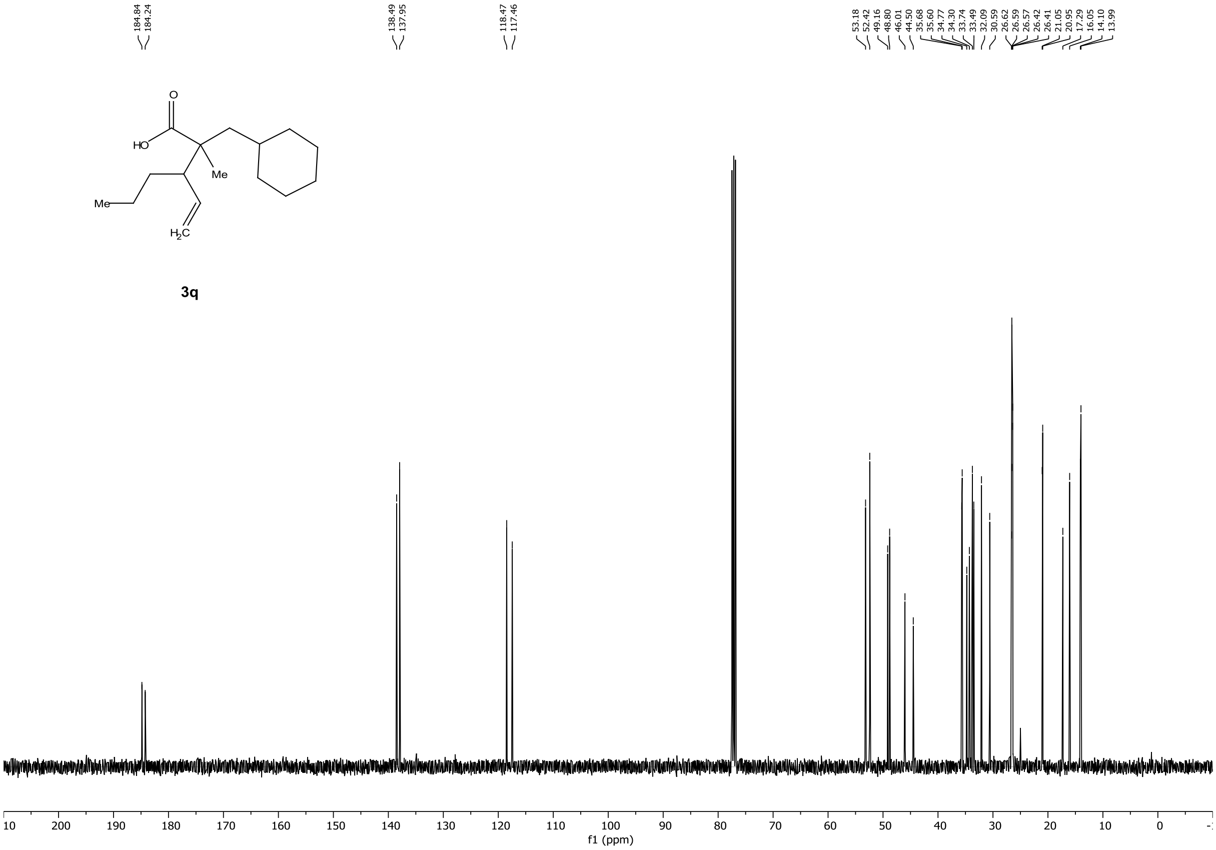

3r, ^{1}H NMR (400 MHz, $CDCl_3$)

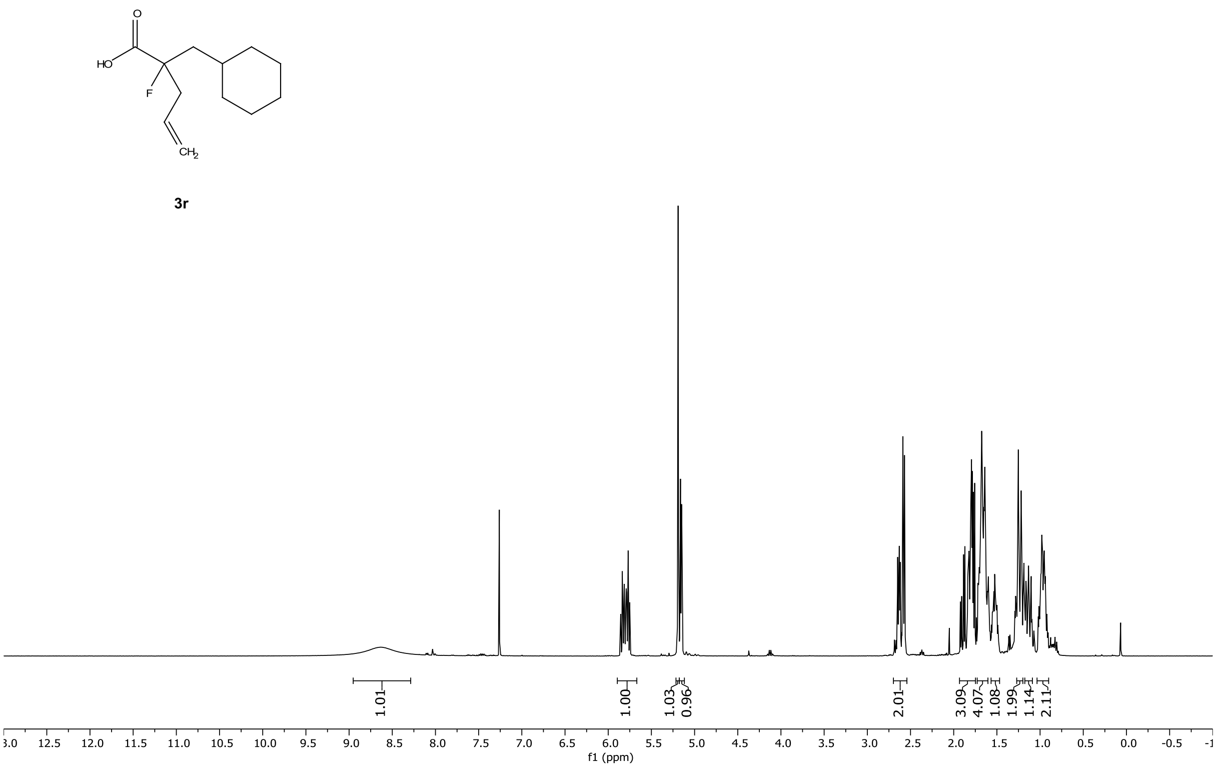

3r, ^{13}C NMR (101 MHz, $CDCl_3$)

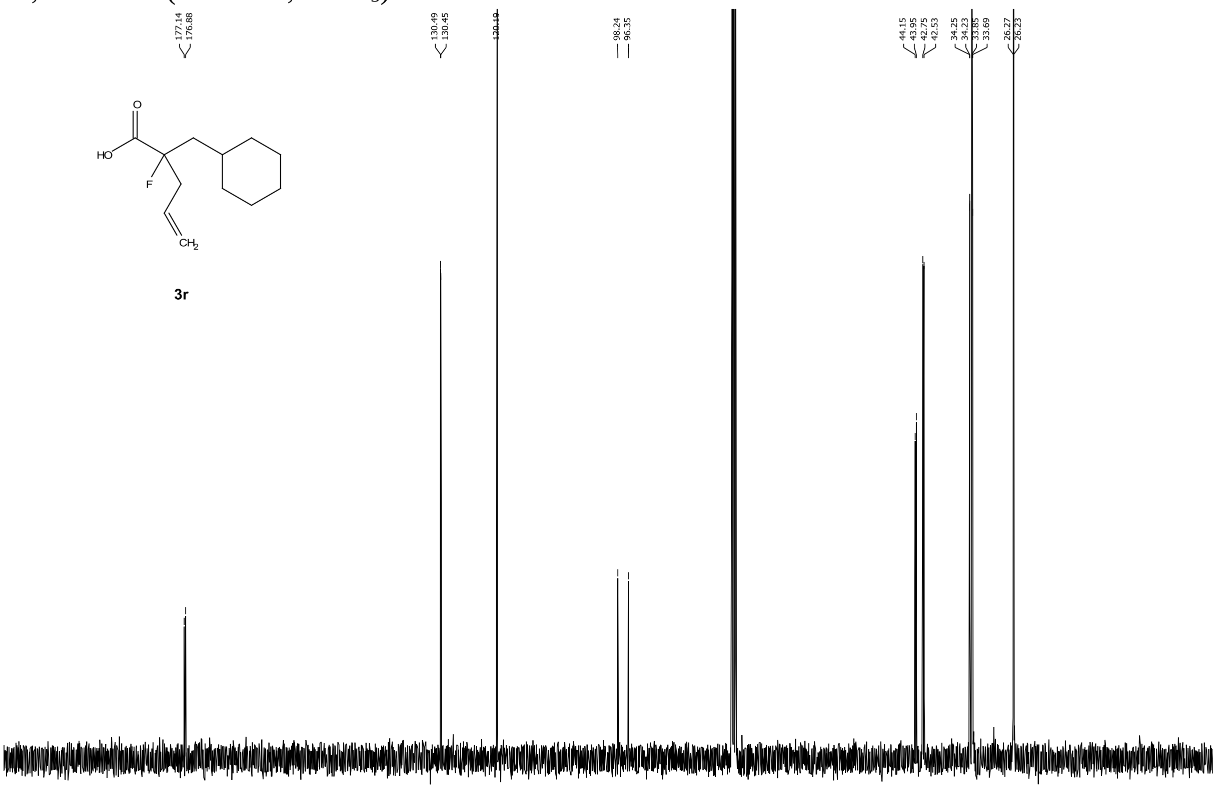

3r, ^{19}F NMR (377 MHz, $CDCl_3$)

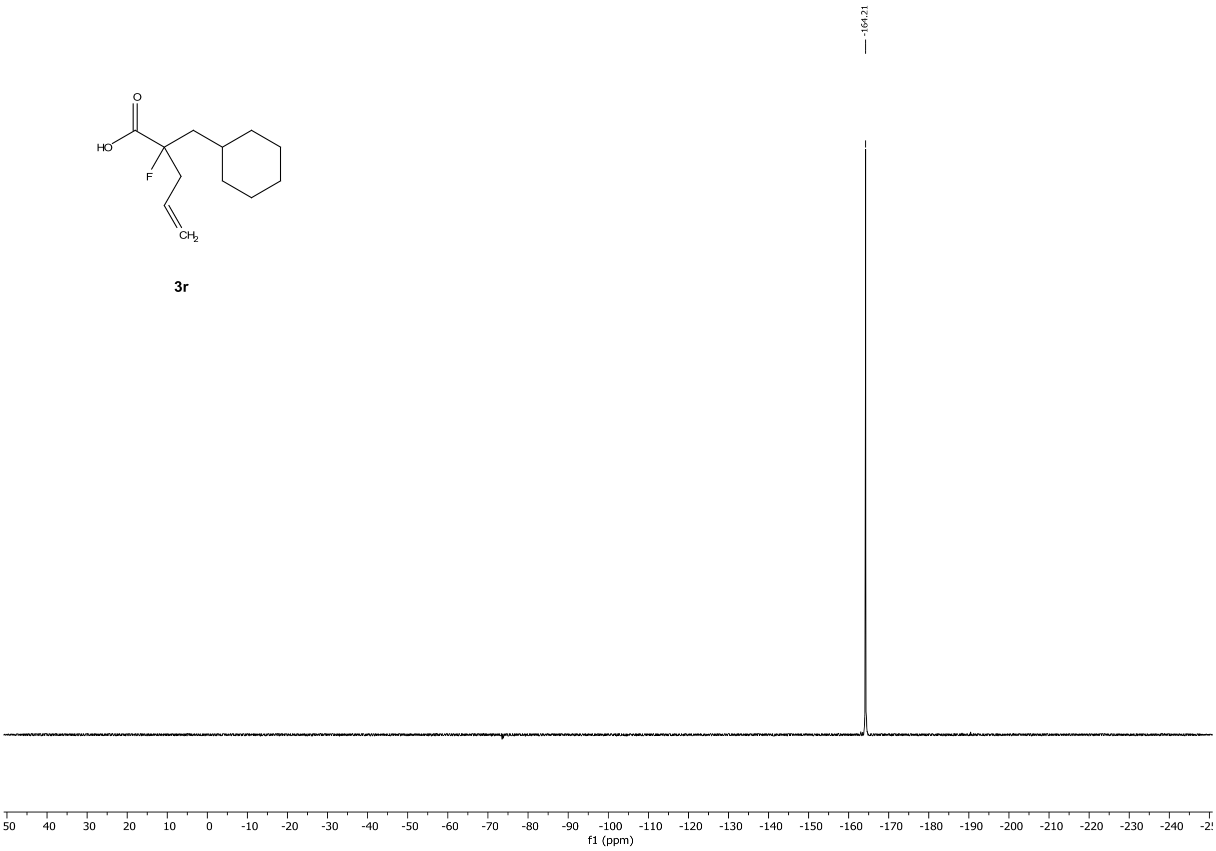

3s, ^{1}H NMR (400 MHz, $CDCl_3$)

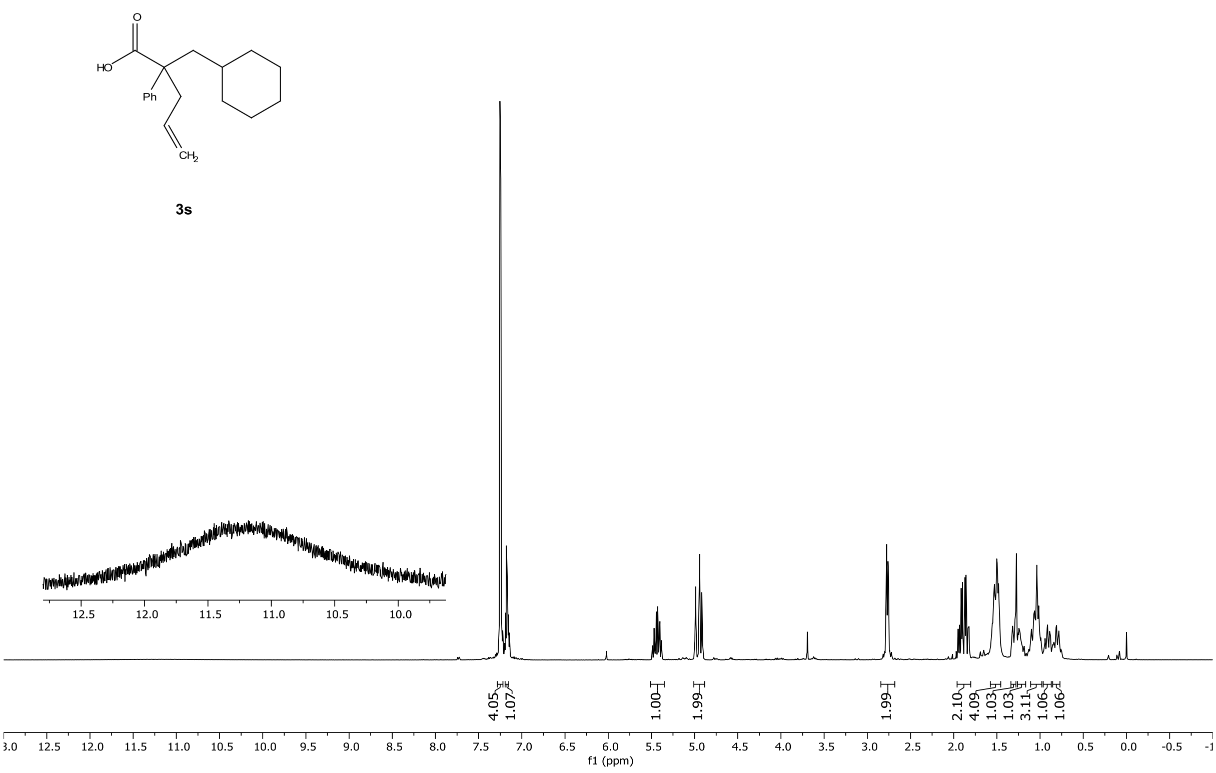

3s, ^{13}C NMR (101 MHz, $CDCl_3$)

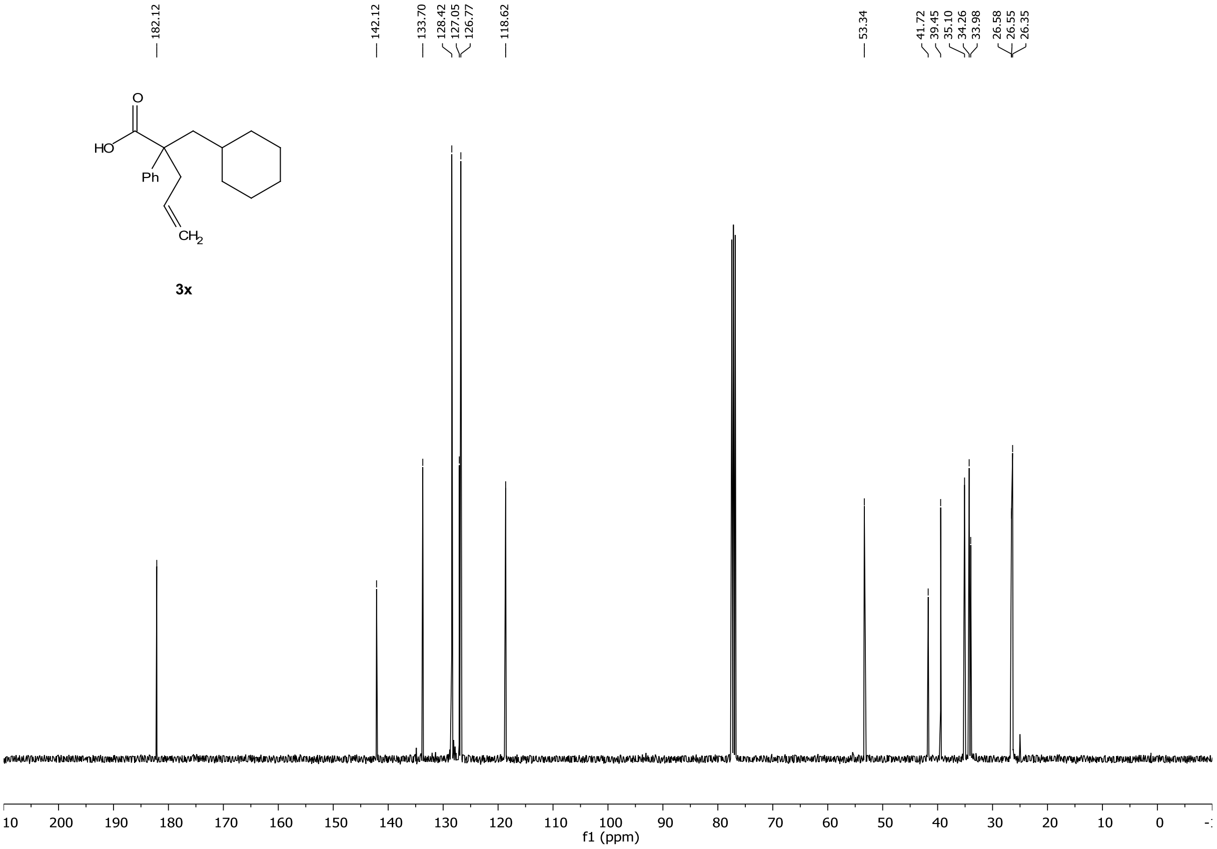

3t, ^{1}H NMR (400 MHz, $CDCl_3$)

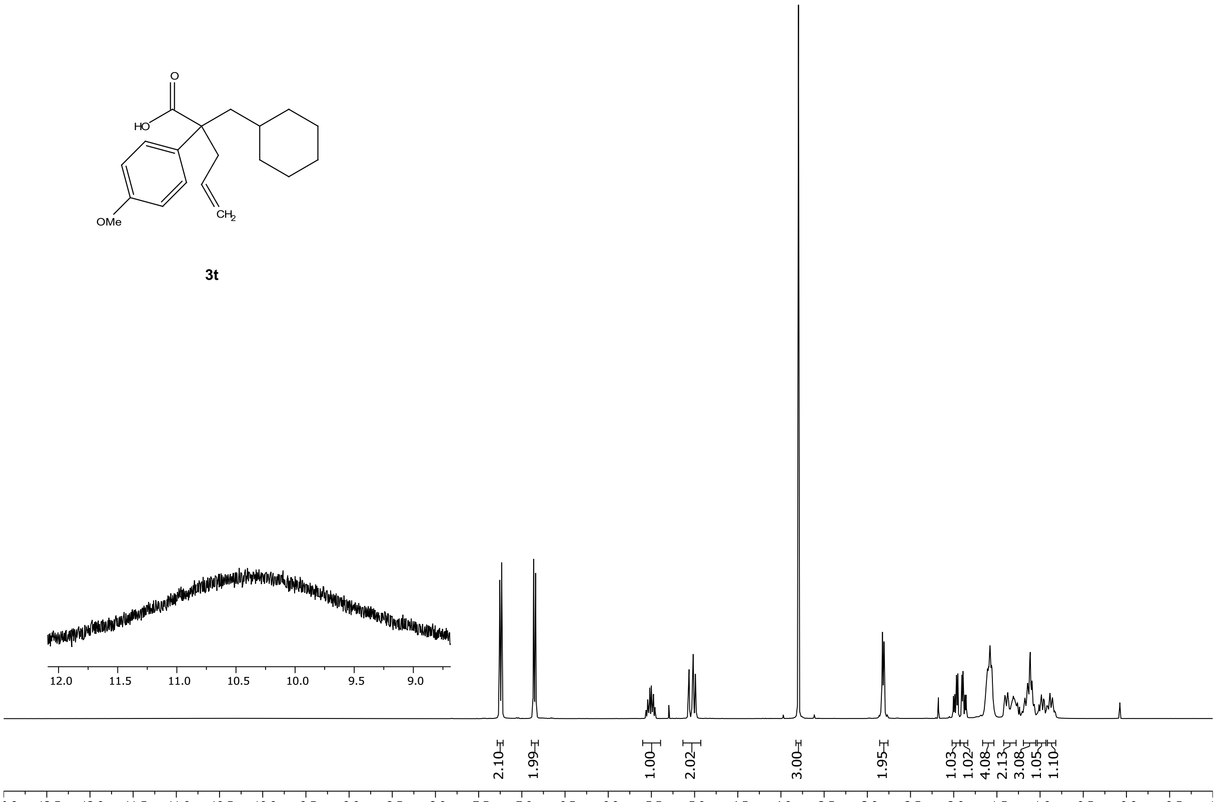

3t, ^{13}C NMR (101 MHz, CDCl$_3$)

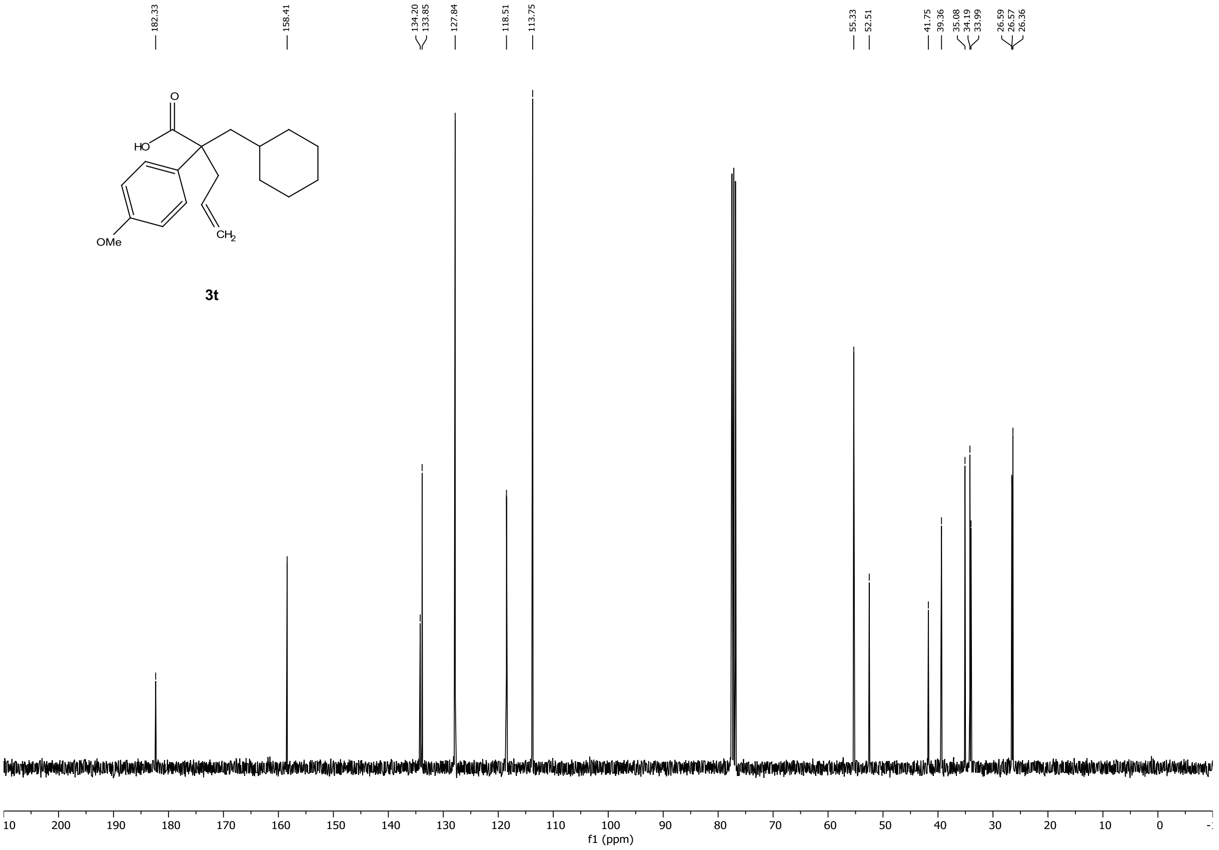

3u, ^{1}H NMR (400 MHz, CDCl$_3$)

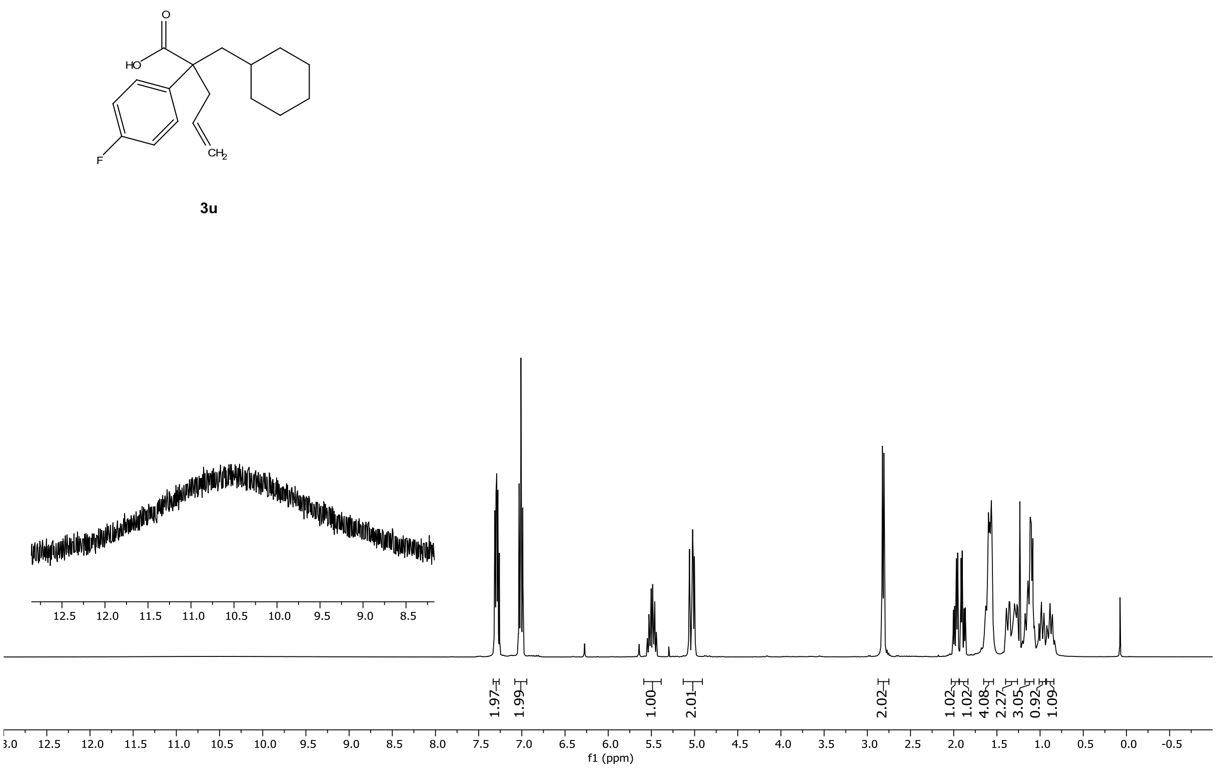

3u, ^{13}C NMR (101 MHz, $CDCl_3$)

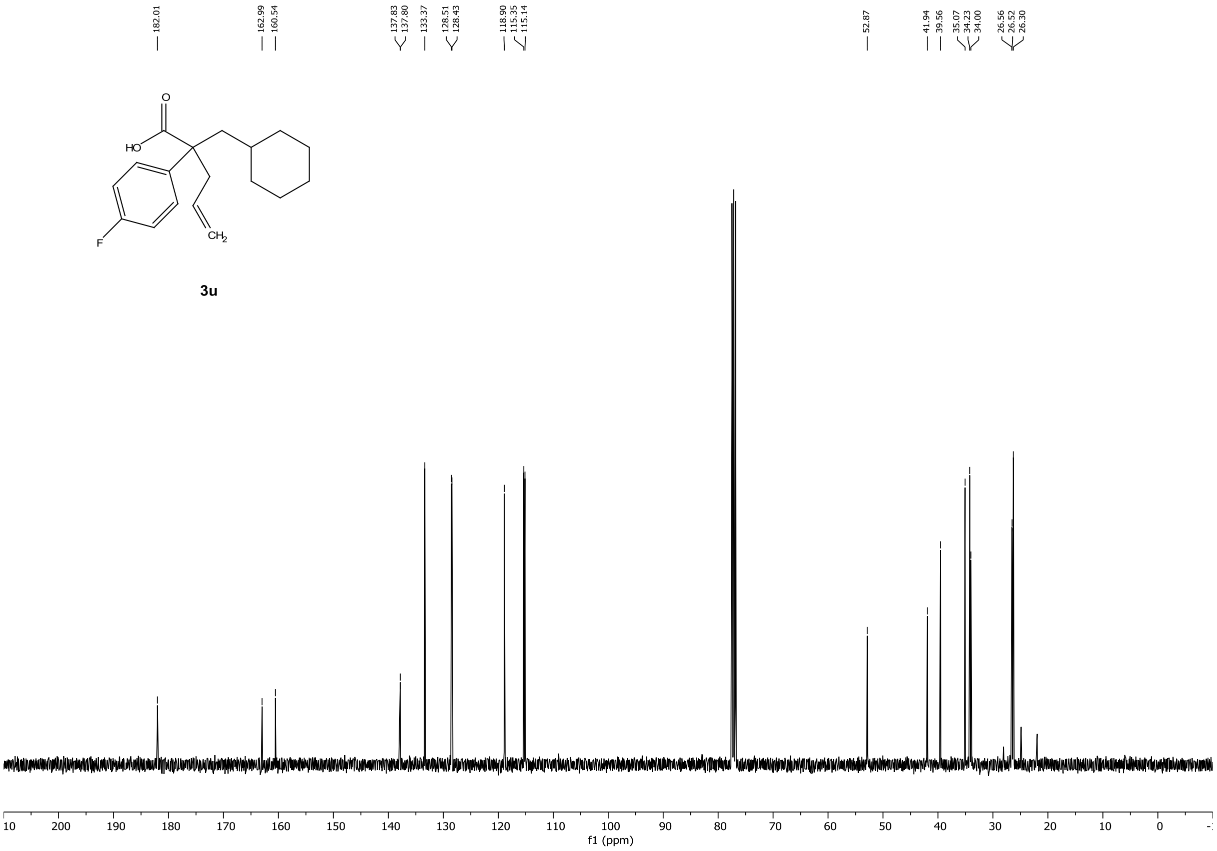

3u, ^{19}F NMR (376 MHz, $CDCl_3$)

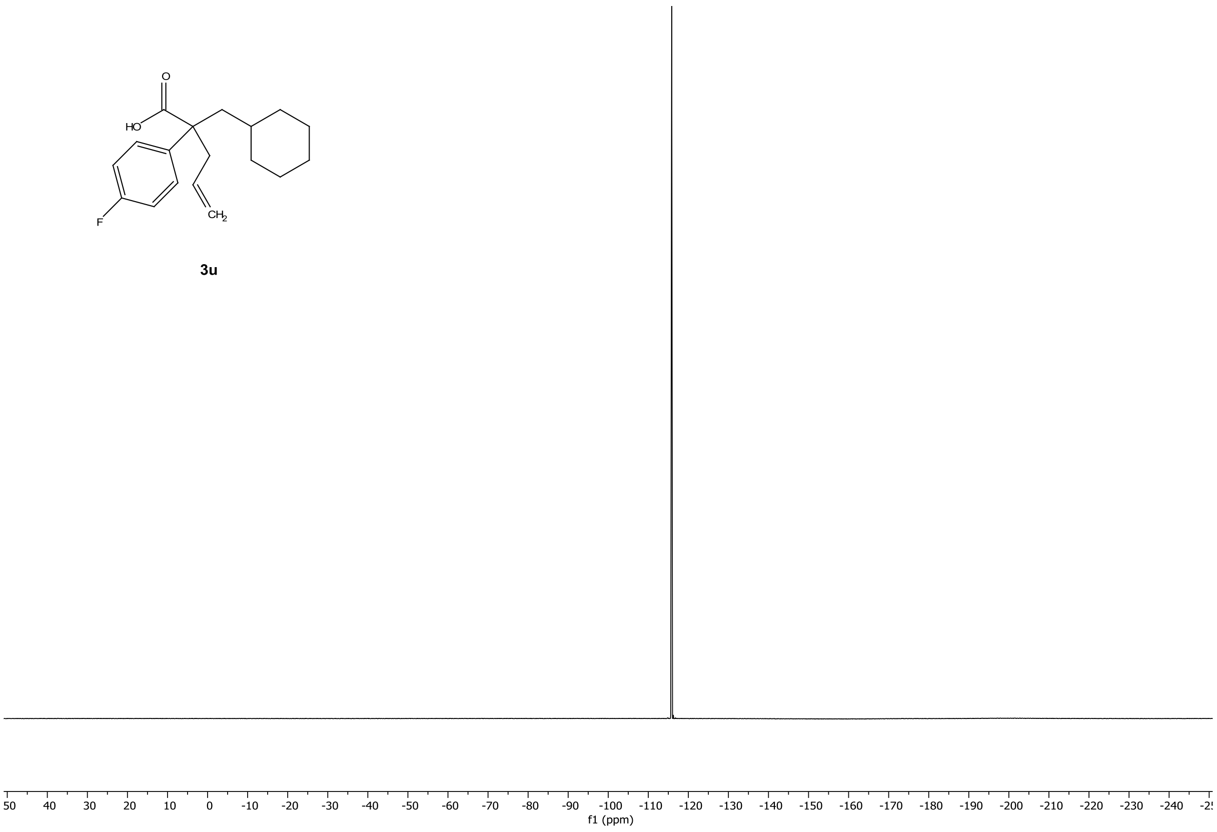

3v, ^{1}H NMR (400 MHz, $CDCl_3$)

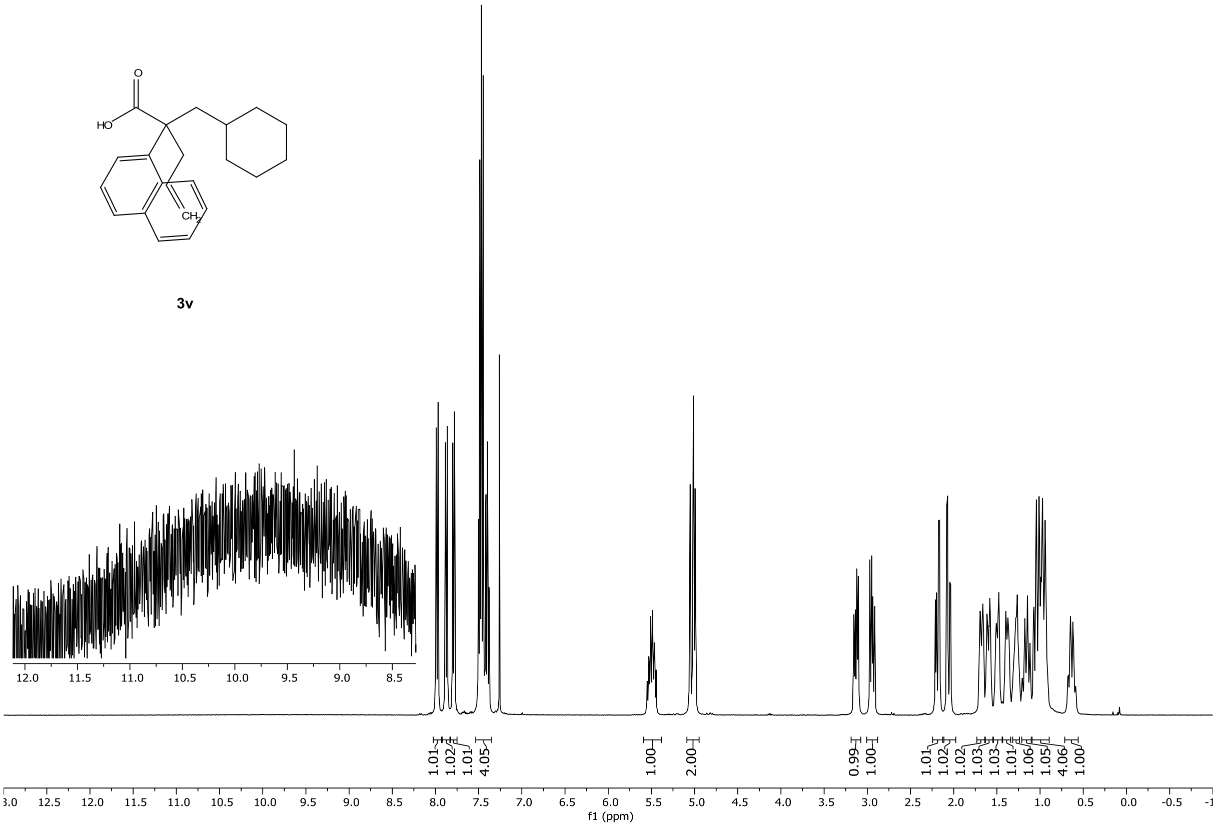

3v, ^{13}C NMR (101 MHz, $CDCl_3$)

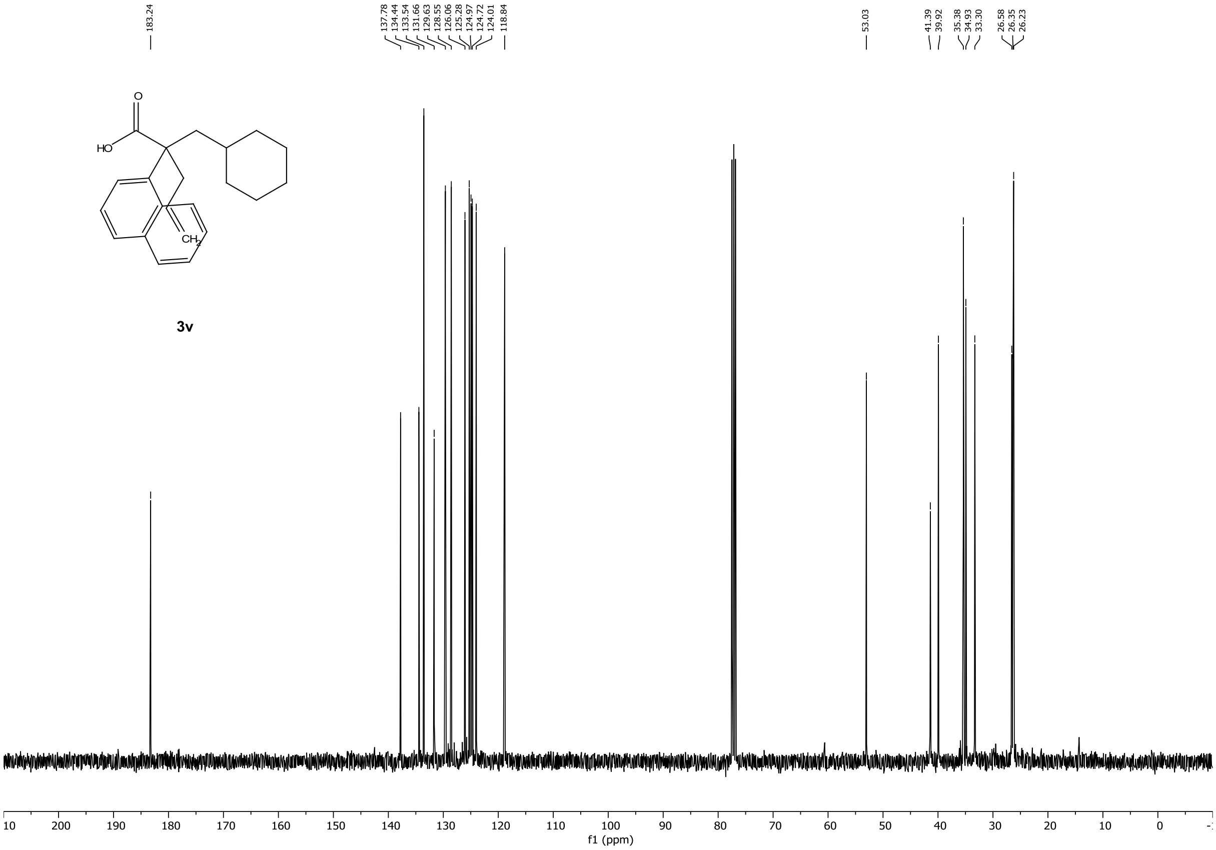

3w, ^{1}H NMR (400 MHz, $CDCl_3$)

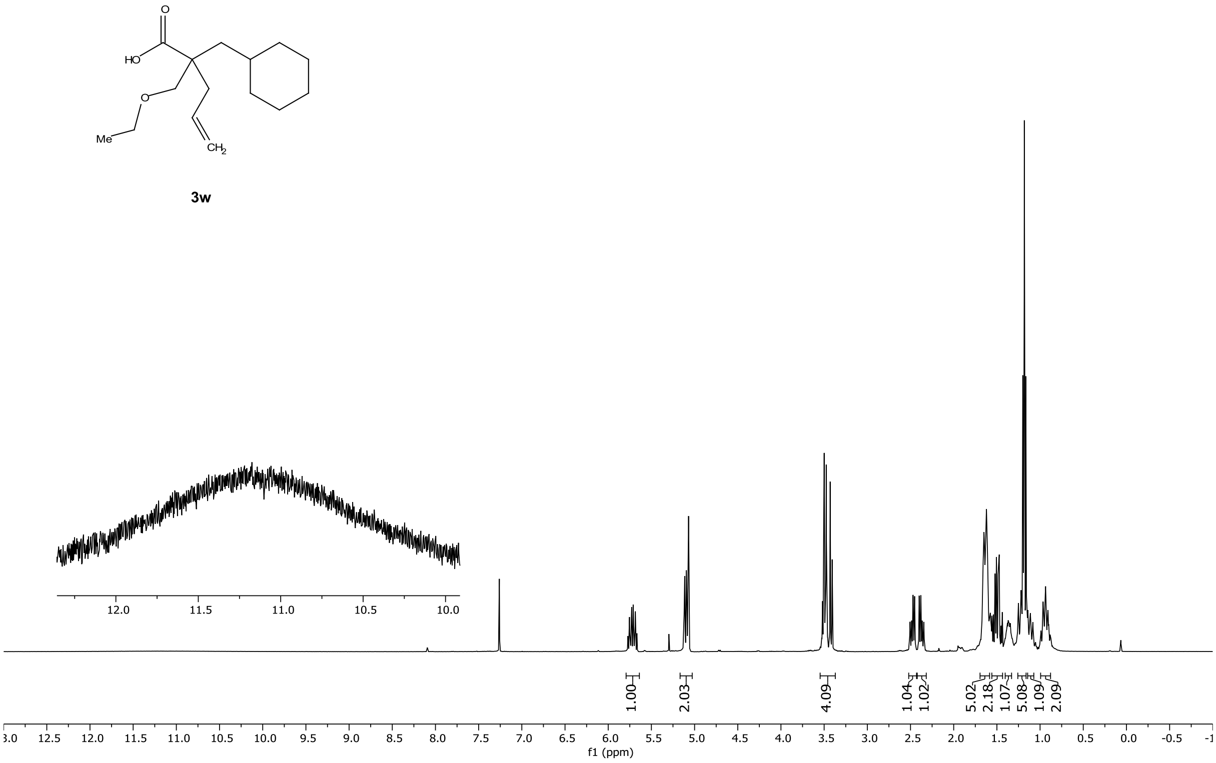

3w, ^{13}C NMR (101 MHz, $CDCl_3$)

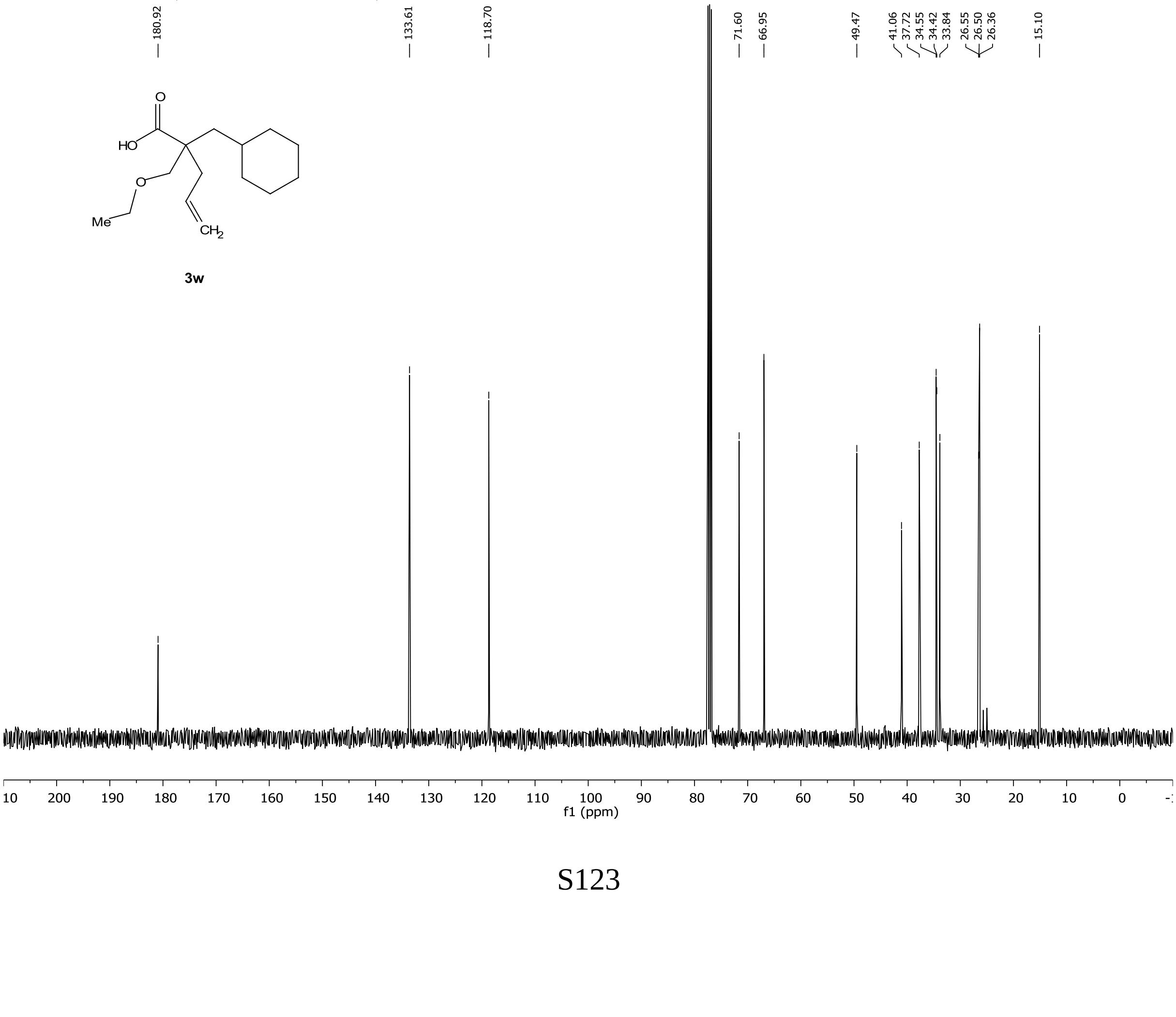

3x, ^{1}H NMR (400 MHz, $CDCl_3$)

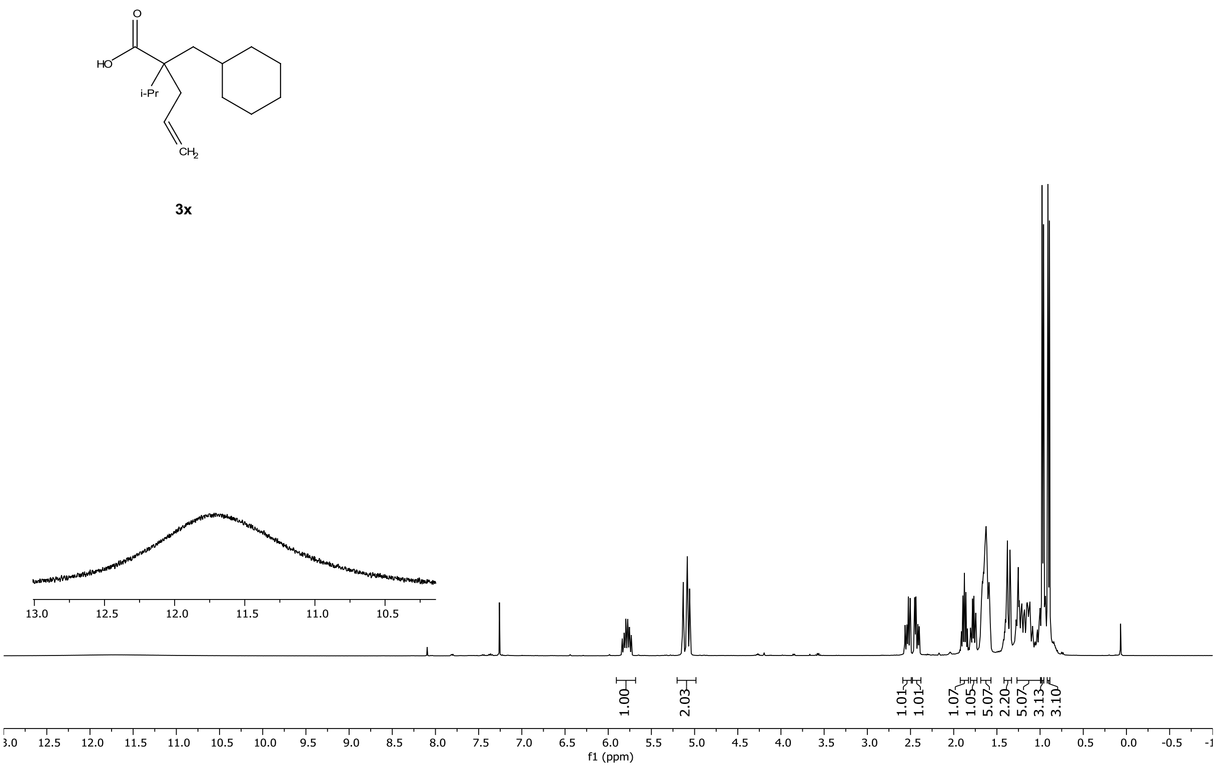

3x, ^{13}C NMR (101 MHz, $CDCl_3$)

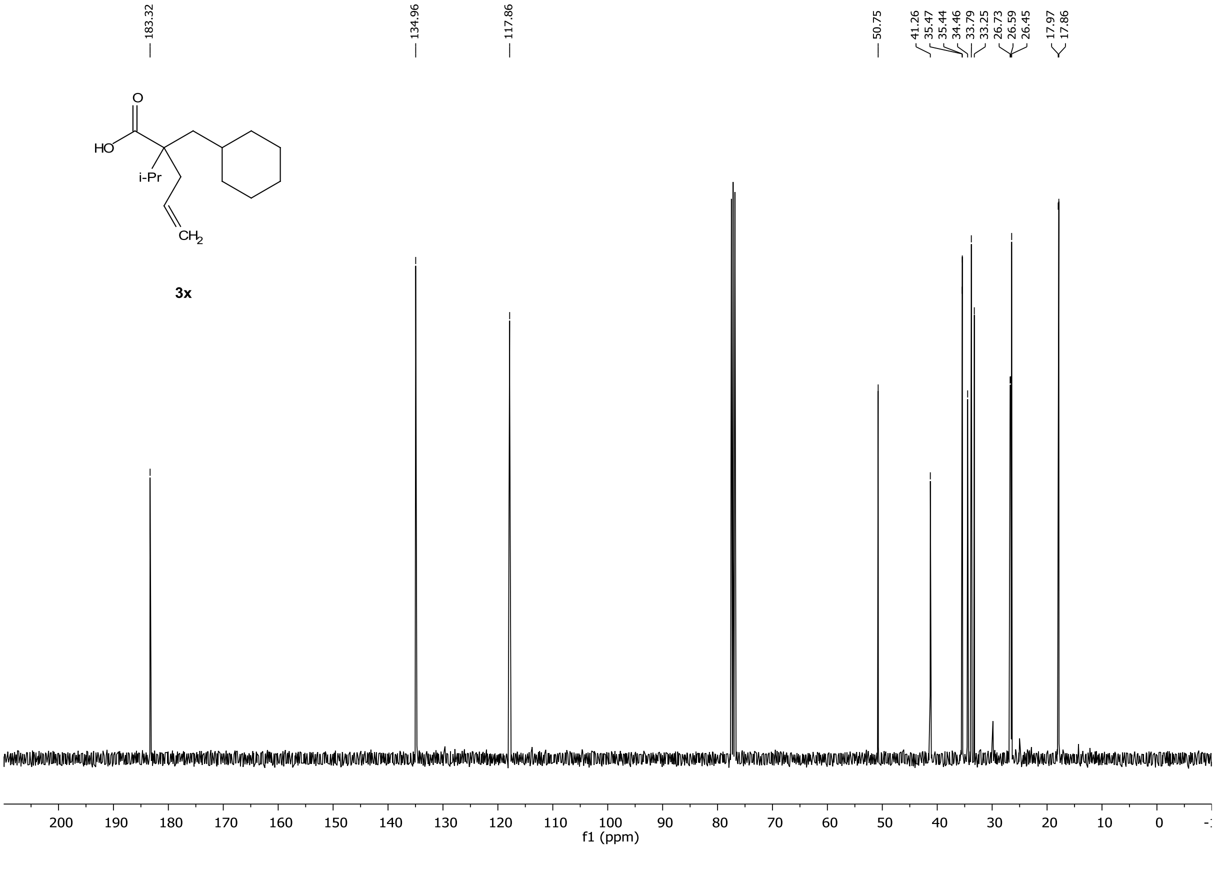

3y, ^{1}H NMR (400 MHz, $CDCl_3$)

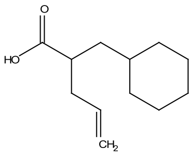

3y

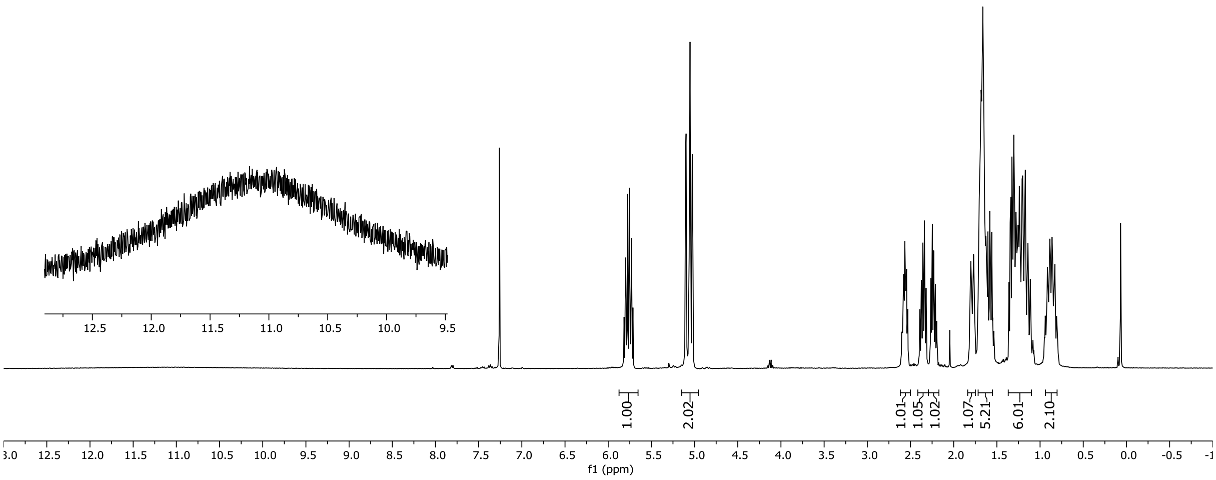

3y, ^{13}C NMR (101 MHz, $CDCl_3$)

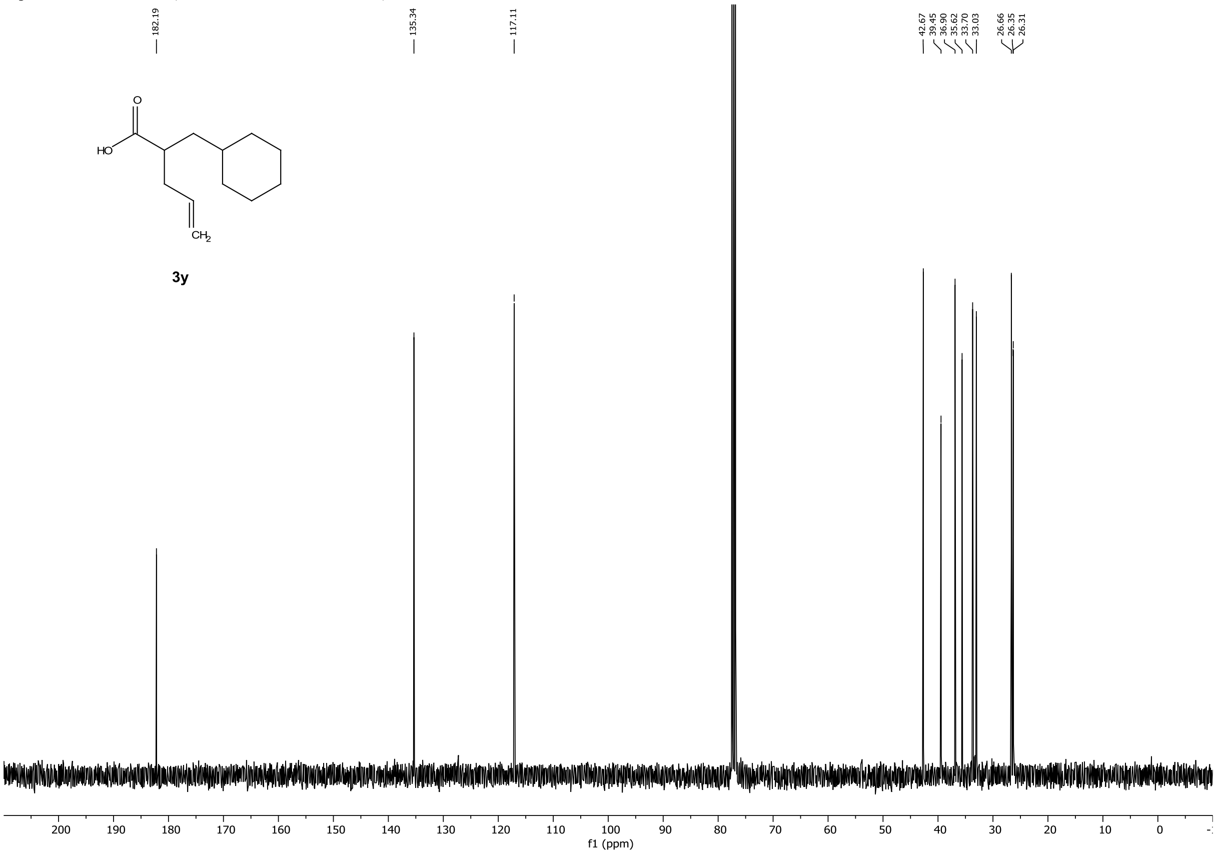

5a, ^{1}H NMR (300 MHz, $CDCl_3$)

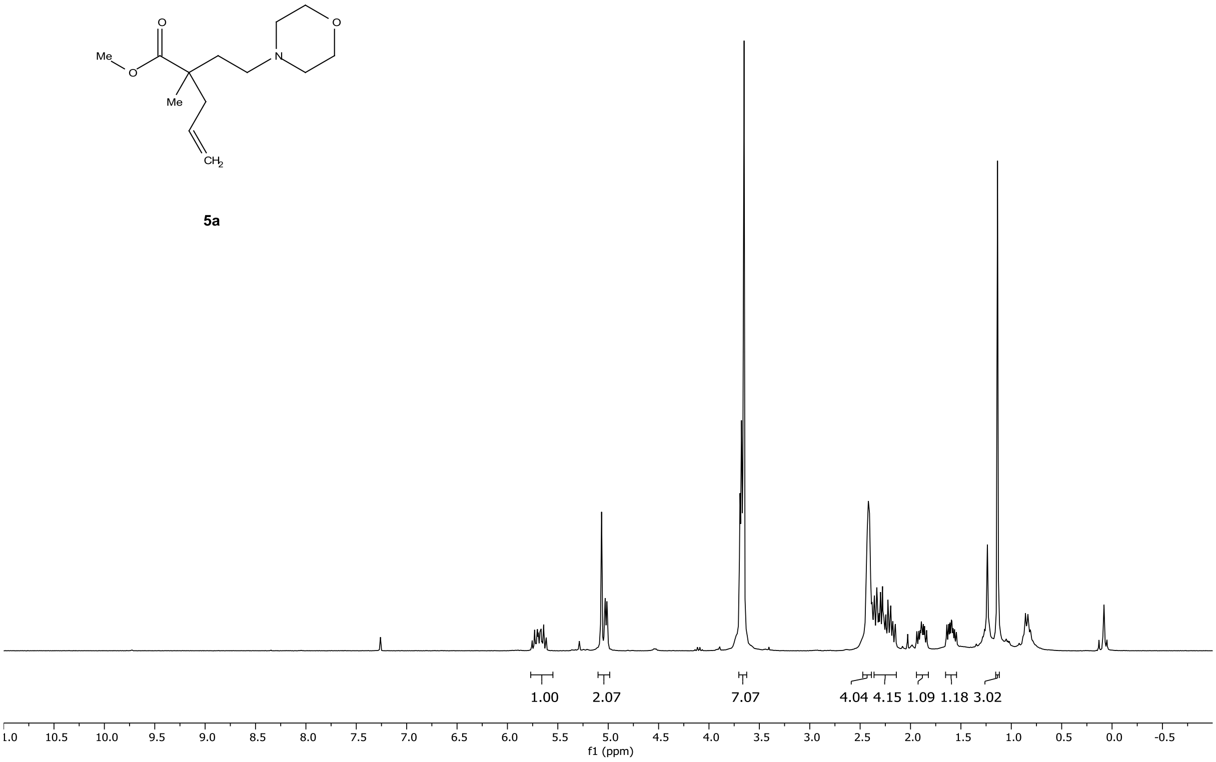

5a, ^{13}C NMR (75 MHz, $CDCl_3$)

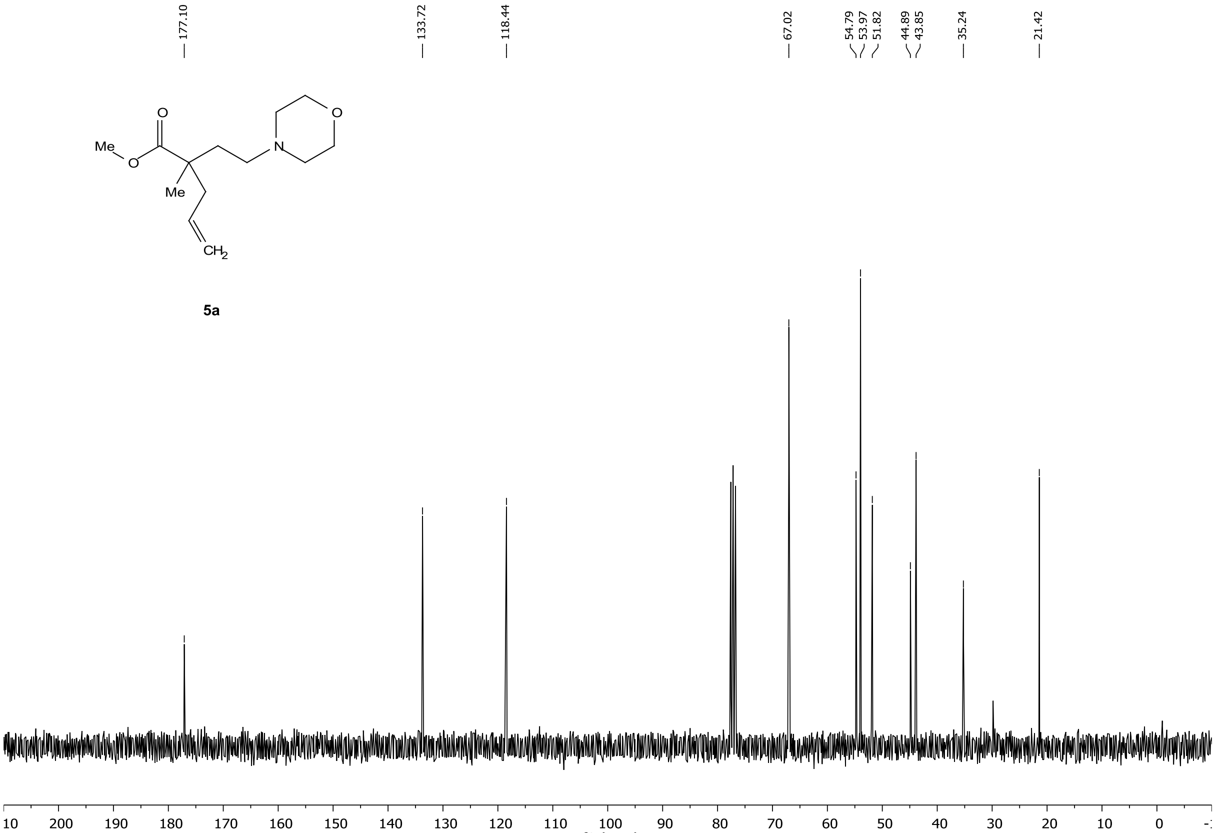

5b, ^{1}H NMR (400 MHz, $CDCl_3$)

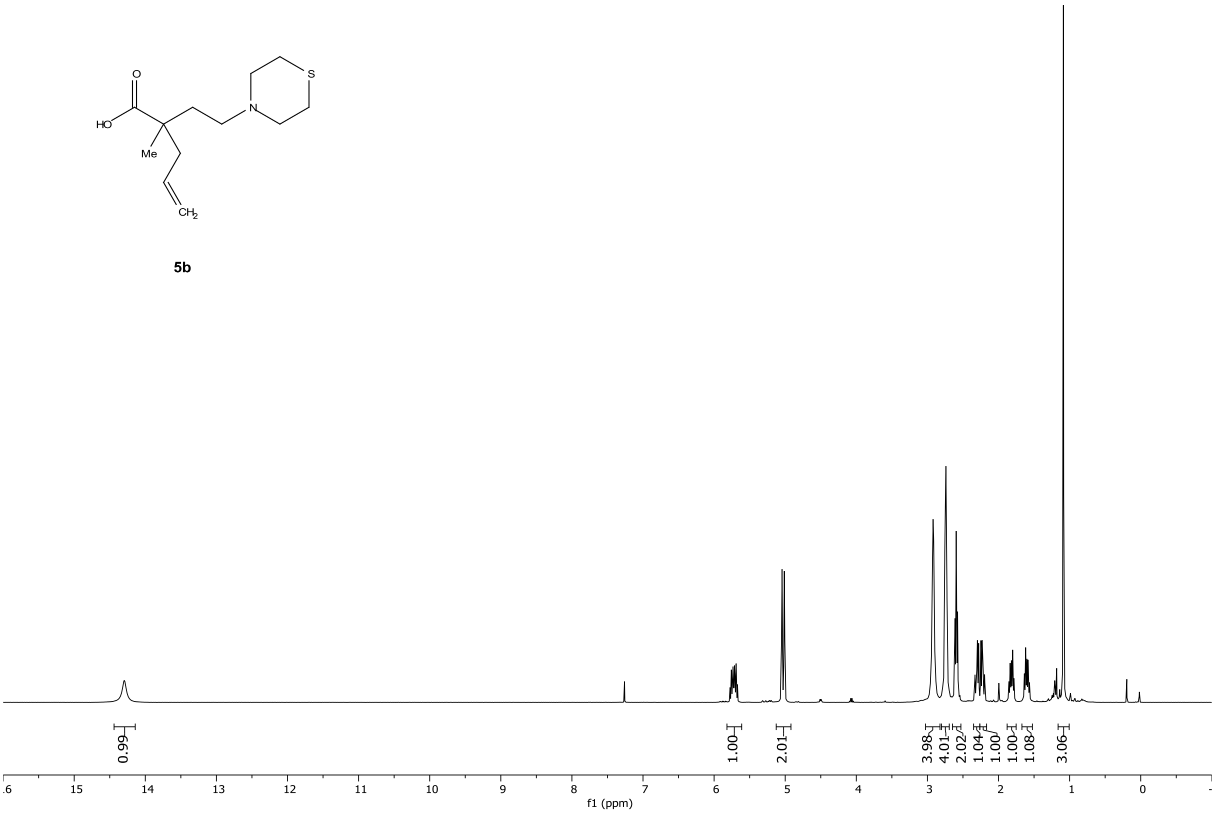

5b, ^{13}C NMR (101 MHz, $CDCl_3$)

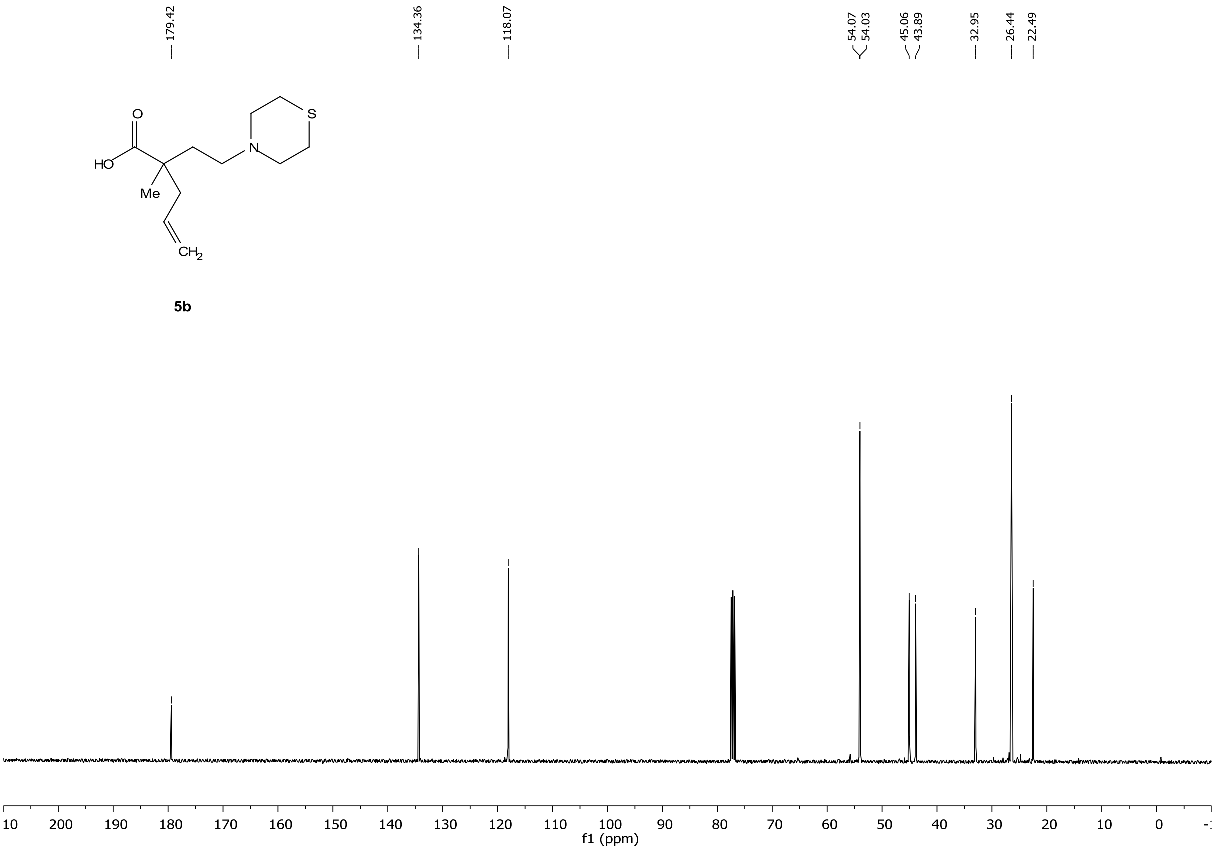

5c, ^{1}H NMR (400 MHz, $CDCl_3$)

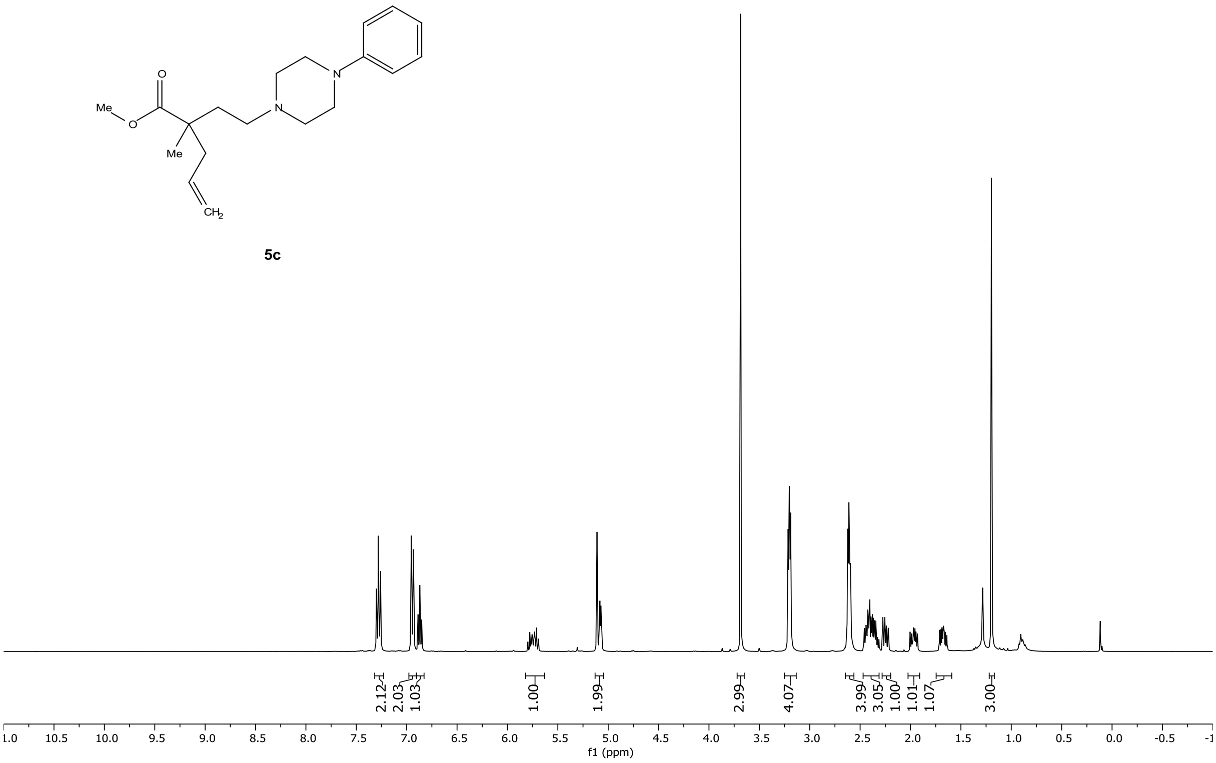

5c, ^{13}C NMR (101 MHz, $CDCl_3$)

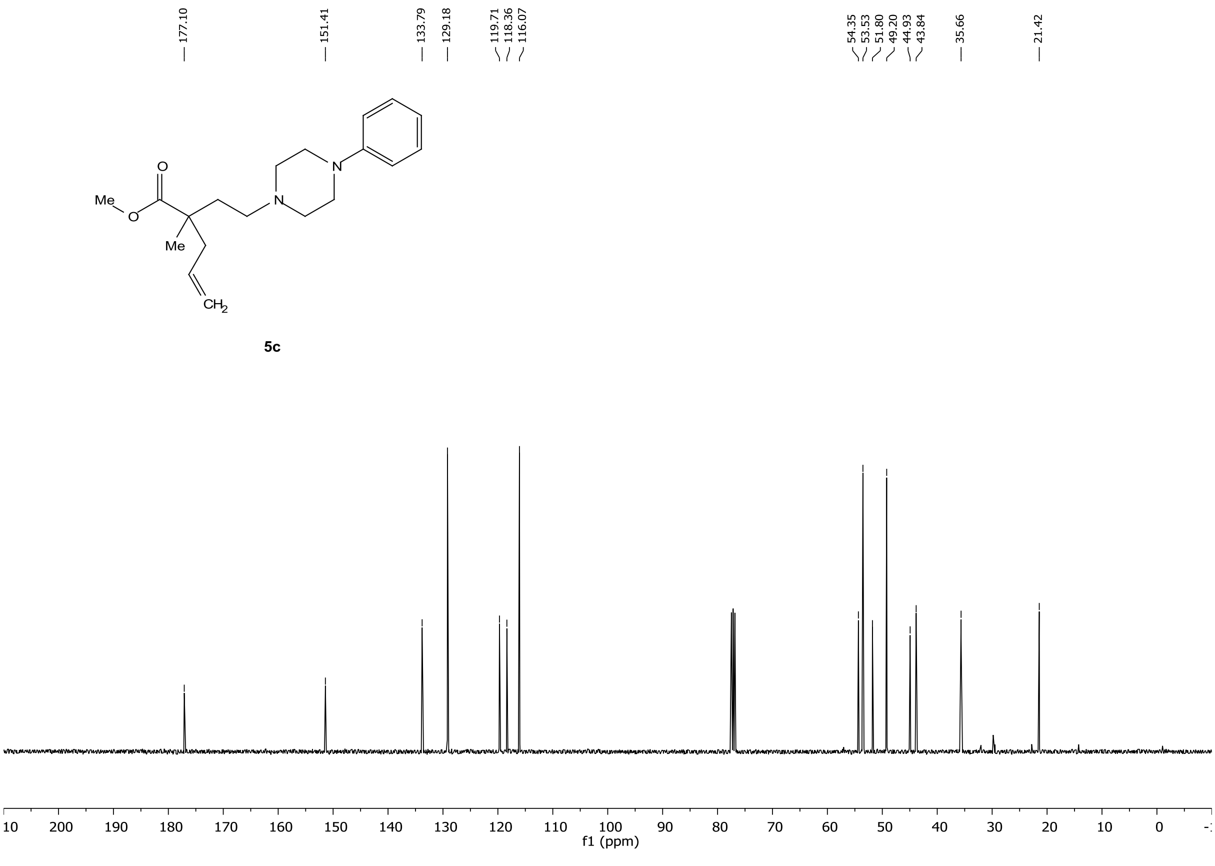

5d, ^{1}H NMR (400 MHz, $CDCl_3$)

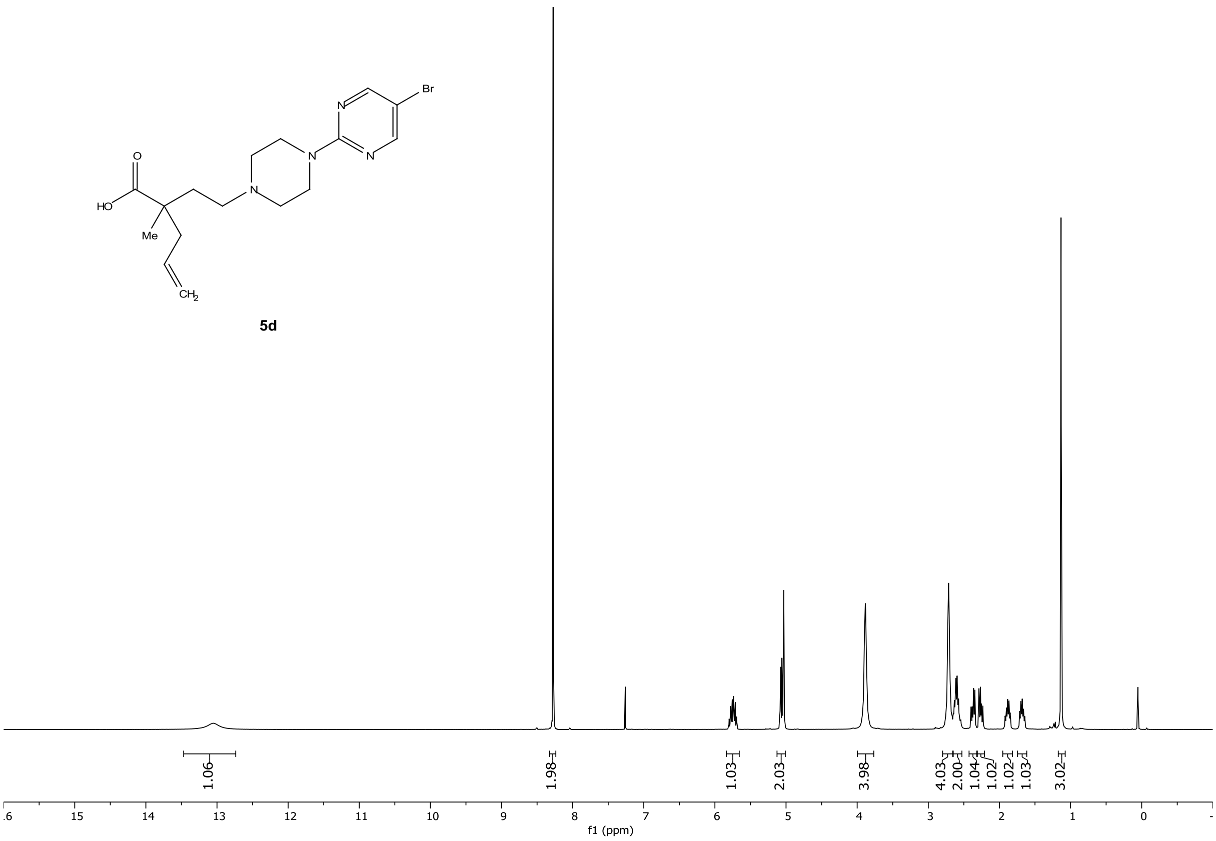

5d, ^{13}C NMR (101 MHz, $CDCl_3$)

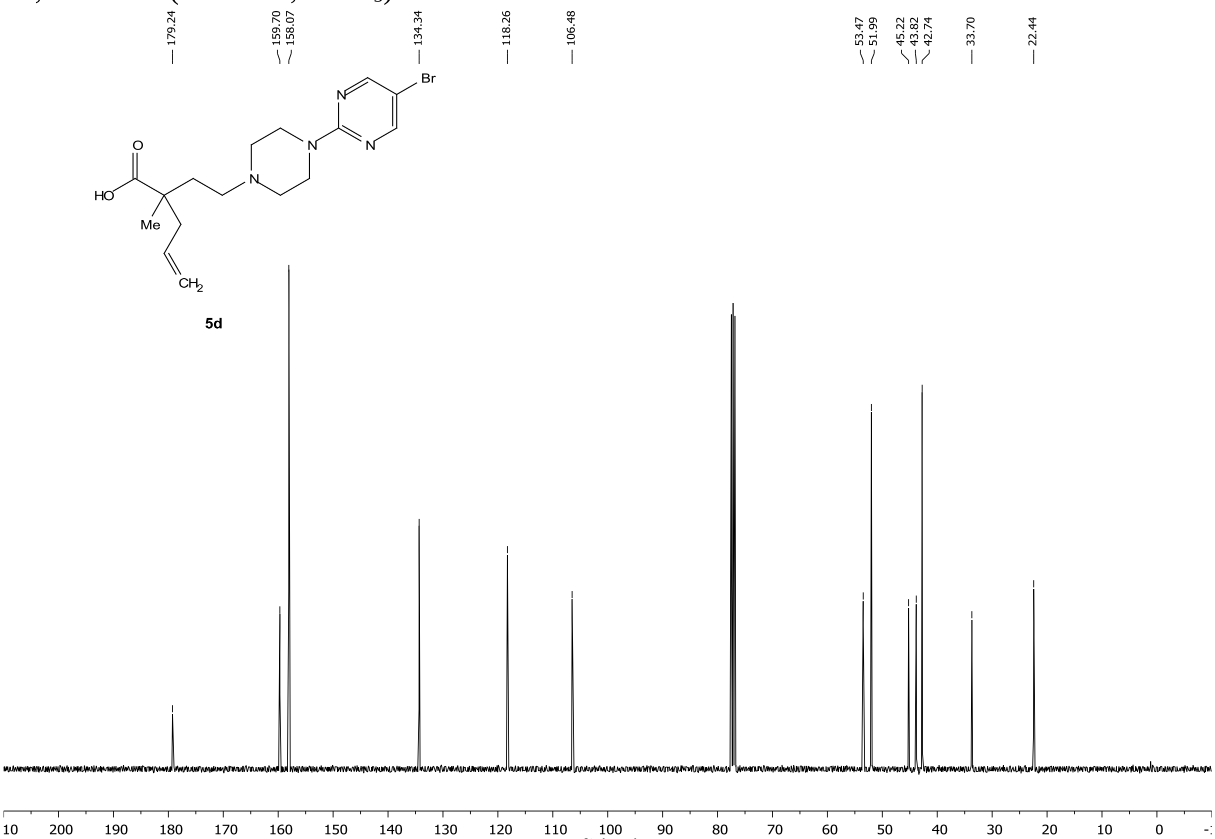

5e, ^{1}H NMR (400 MHz, $CDCl_3$)

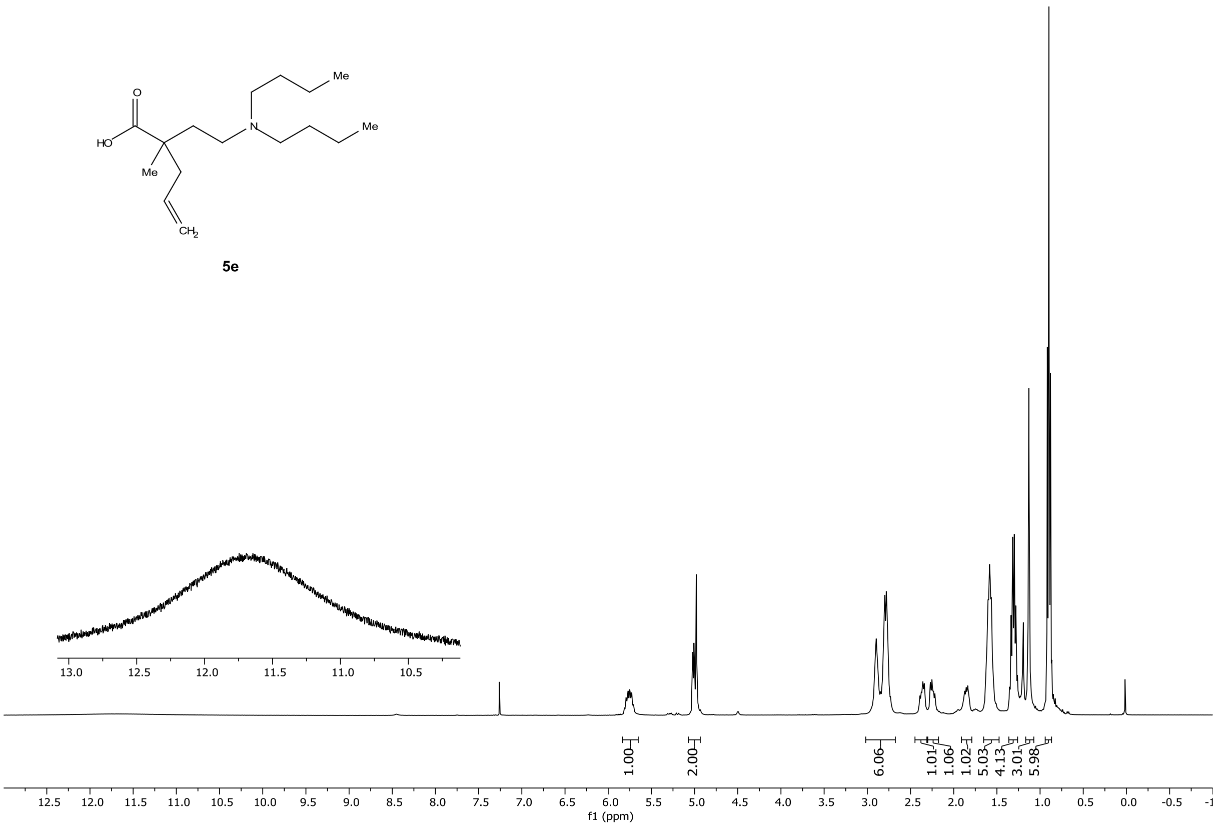

5e, ^{13}C NMR (101 MHz, $CDCl_3$)

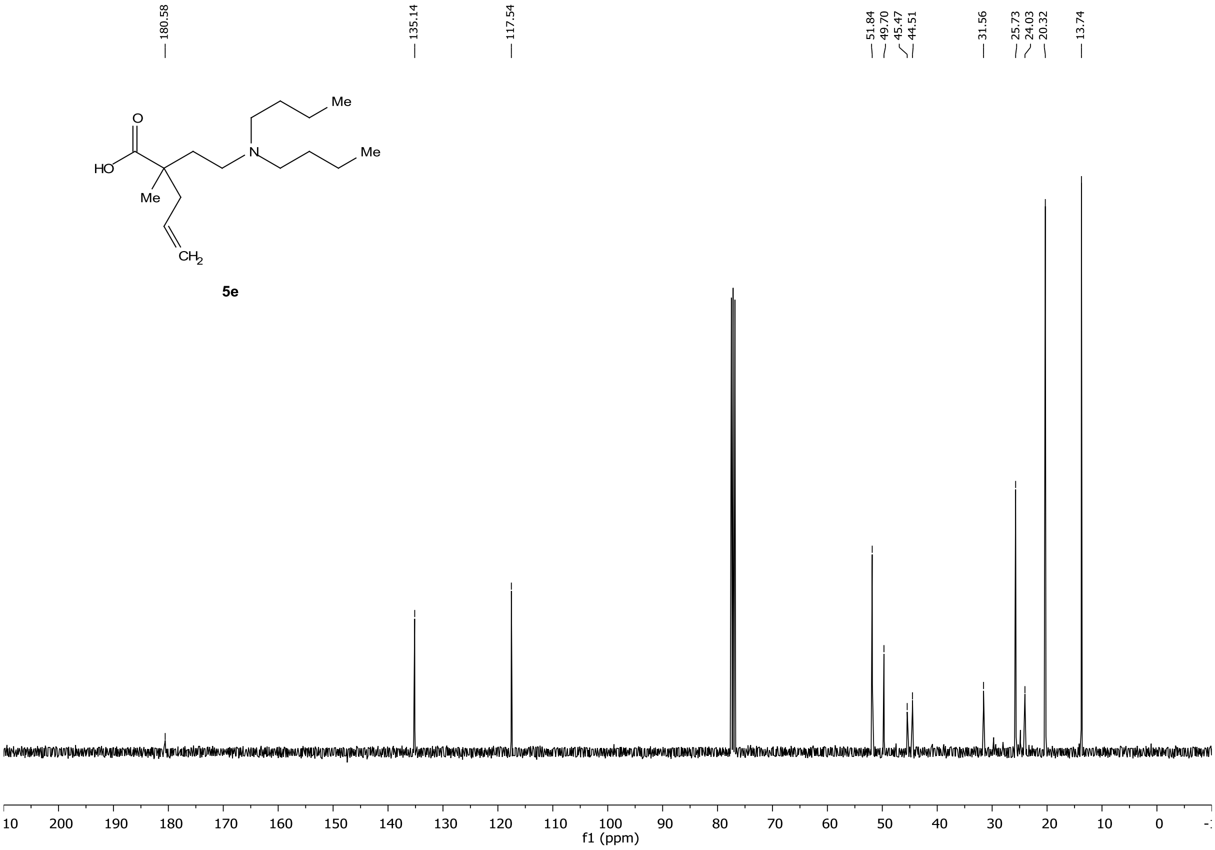

5f, ^{1}H NMR (400 MHz, $CDCl_3$)

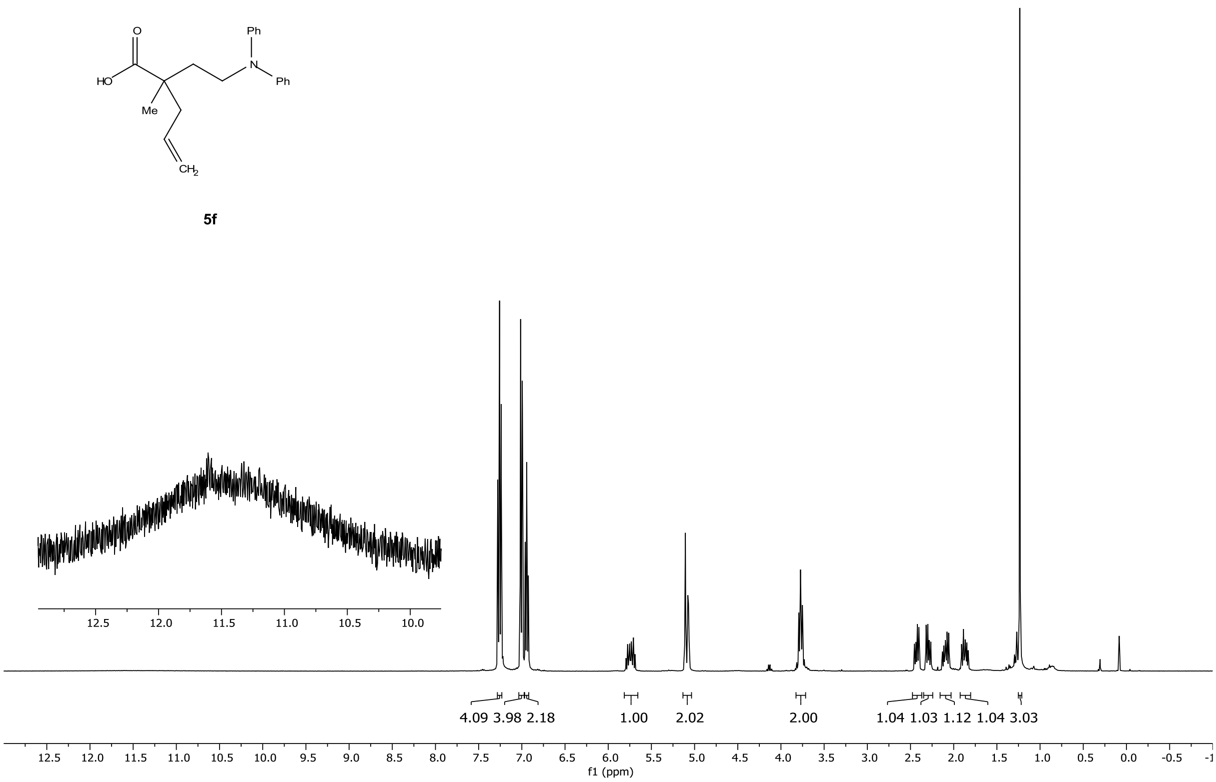

5f, ^{13}C NMR (126 MHz, $CDCl_3$)

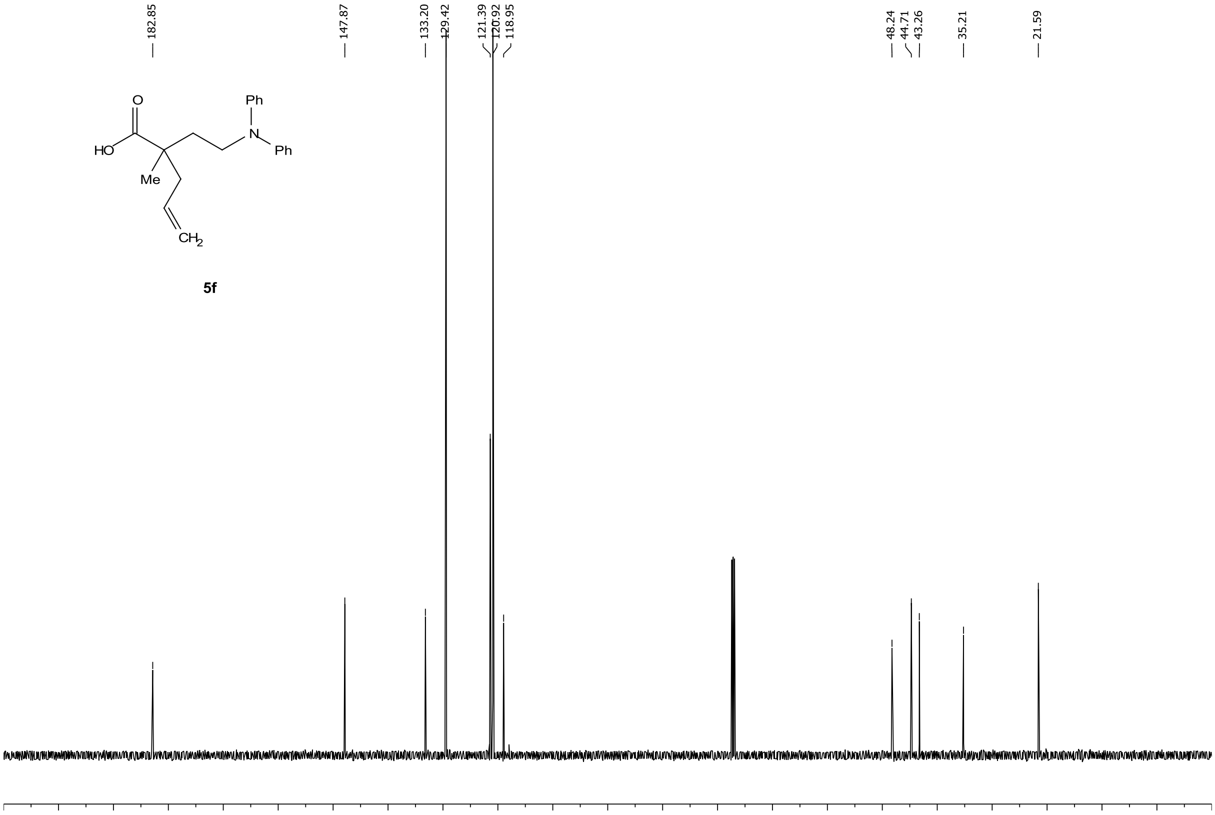

5g, ^{1}H NMR (400 MHz, $CDCl_3$)

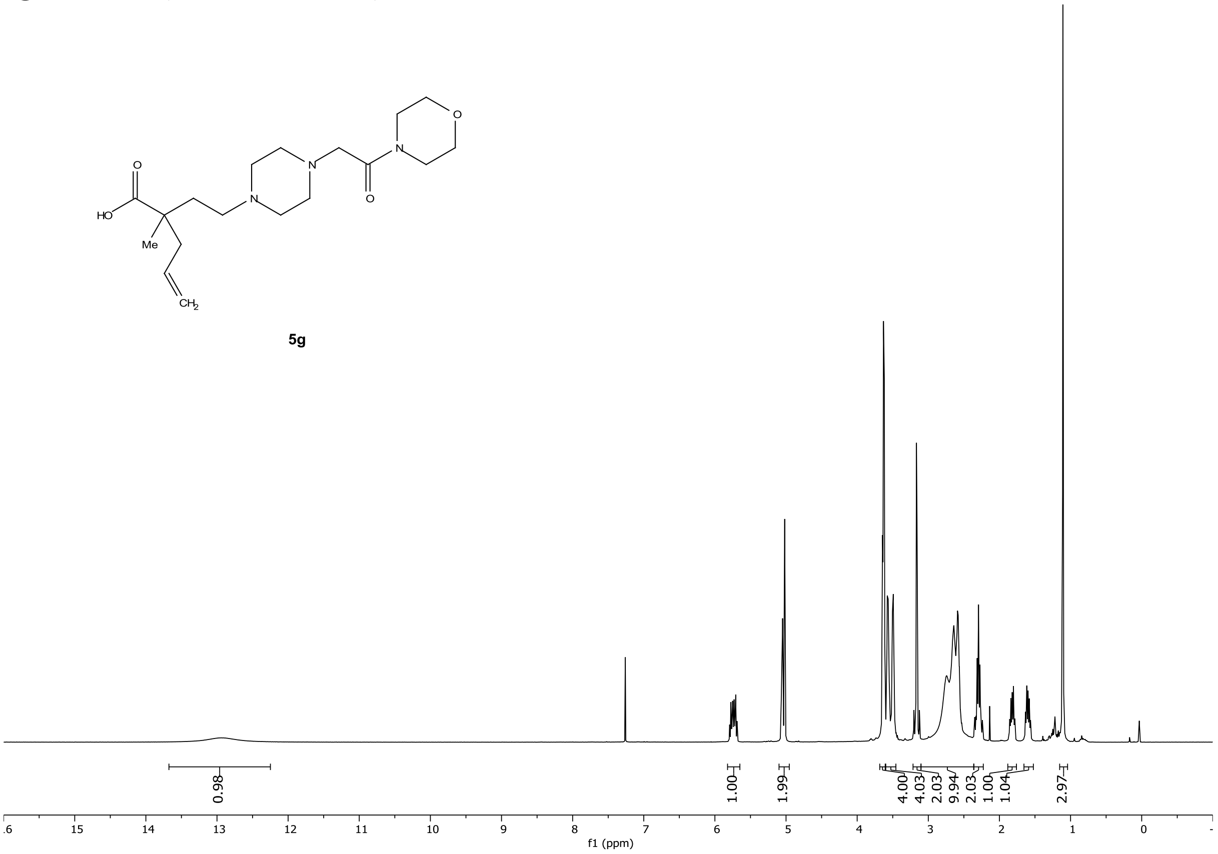

5g, ^{13}C NMR (101 MHz, $CDCl_3$)

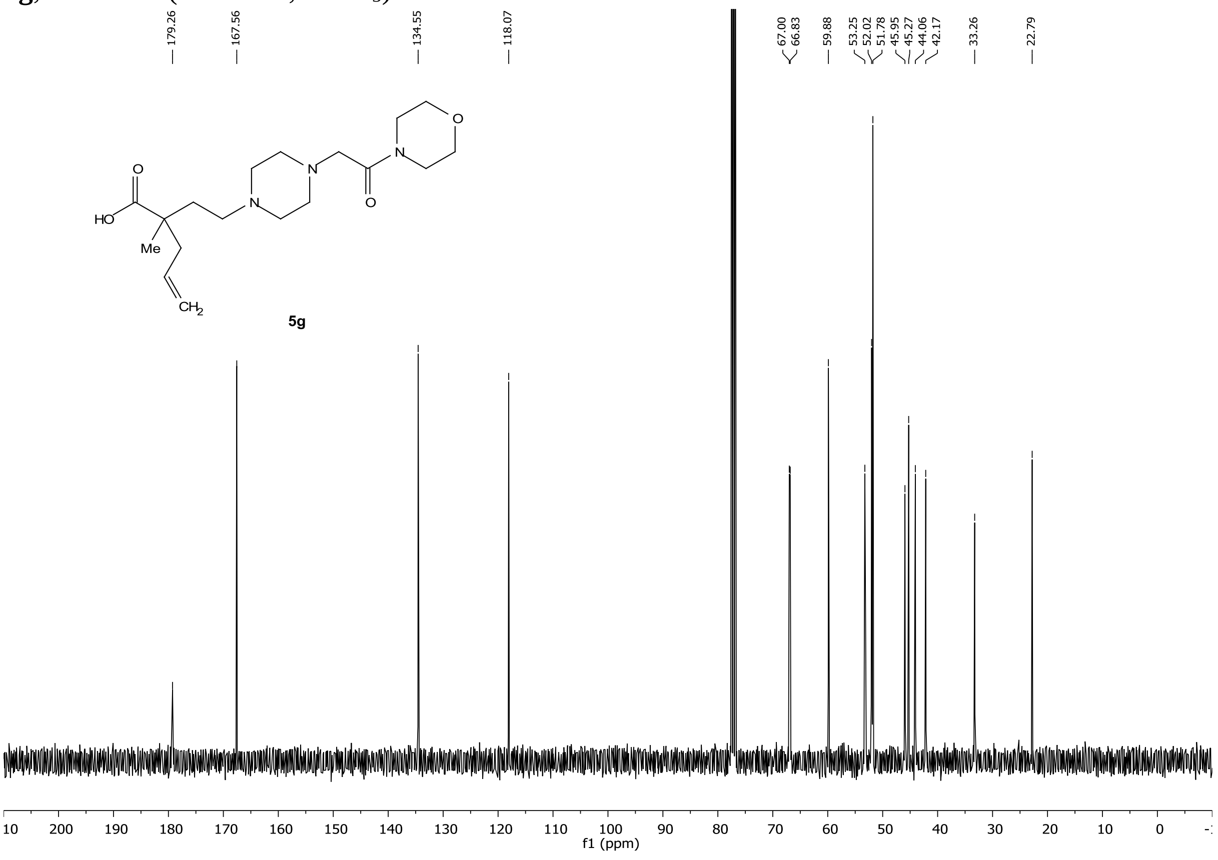

5h, ^{1}H NMR (400 MHz, $CDCl_3$, both diastereomers)

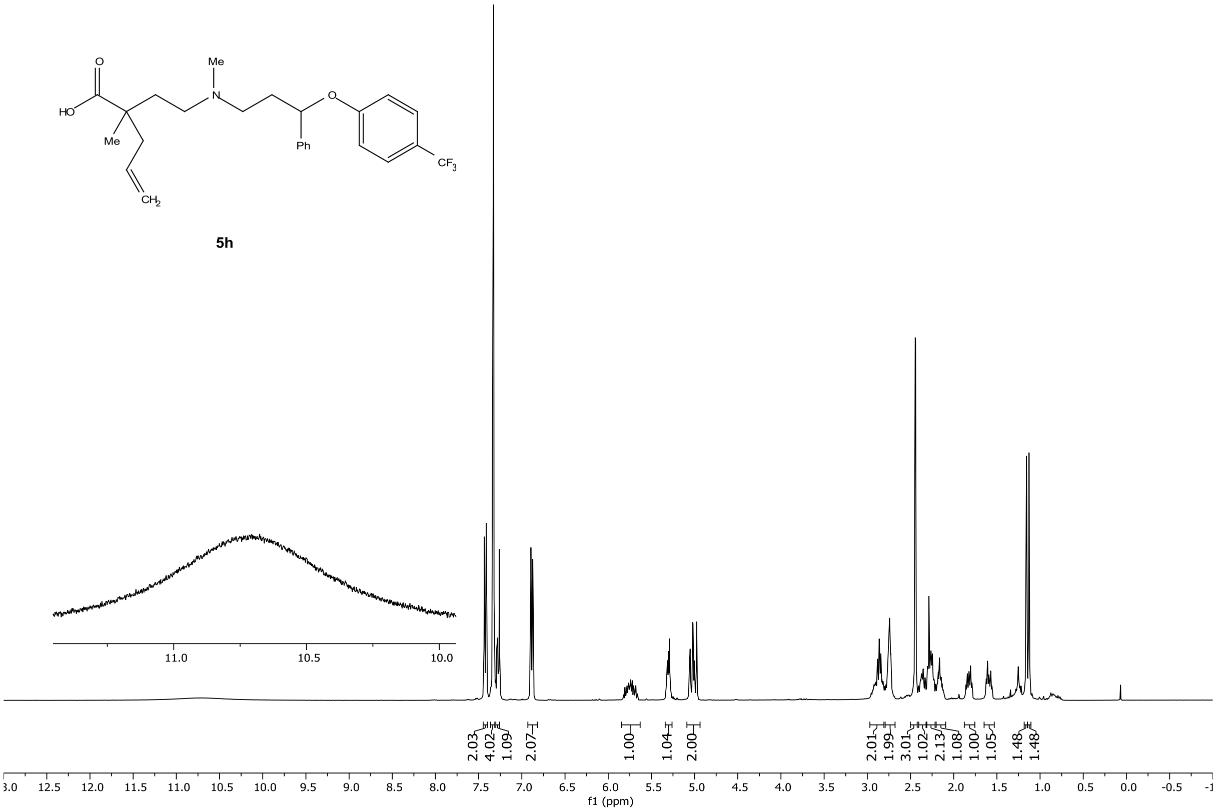

5h, ^{13}C NMR (101 MHz, $CDCl_3$, both diastereomers)

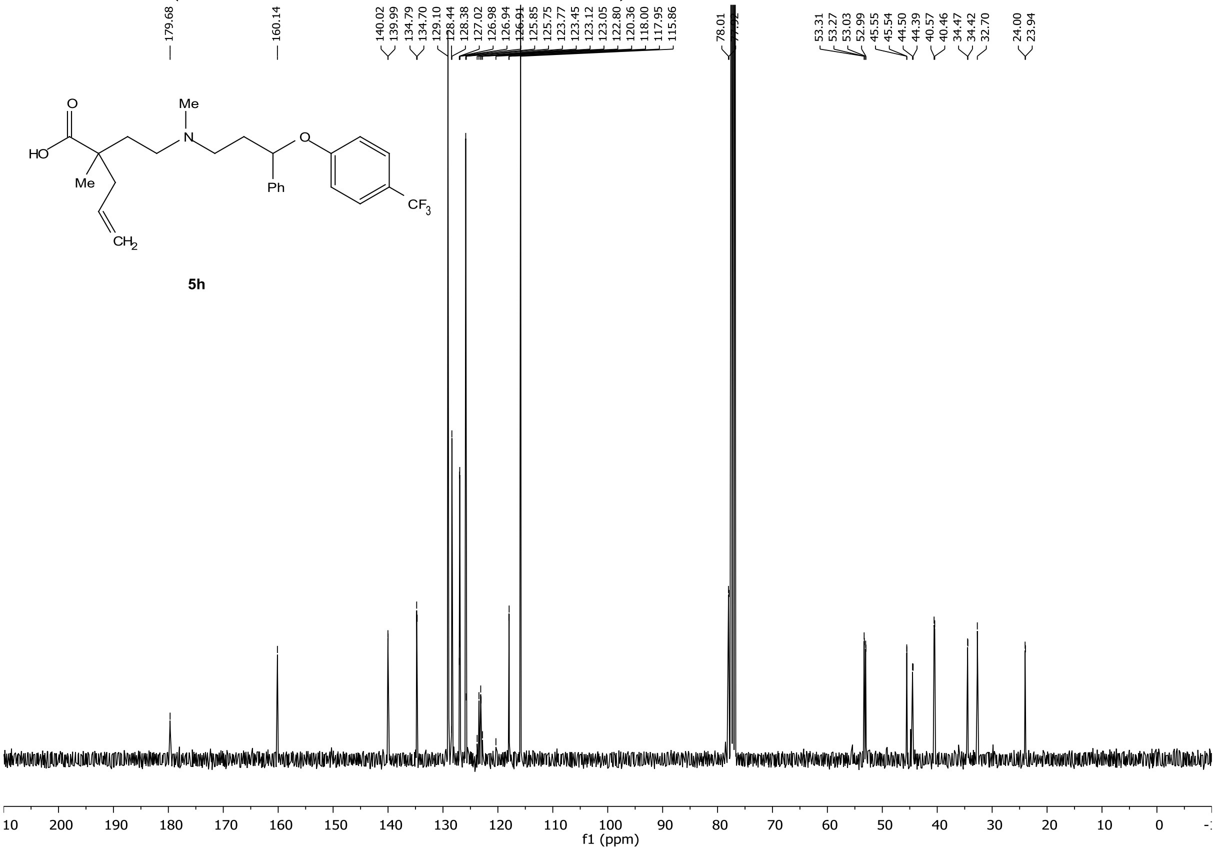

5h, ^{19}F NMR (376 MHz, CDCl$_3$, both diastereomers)

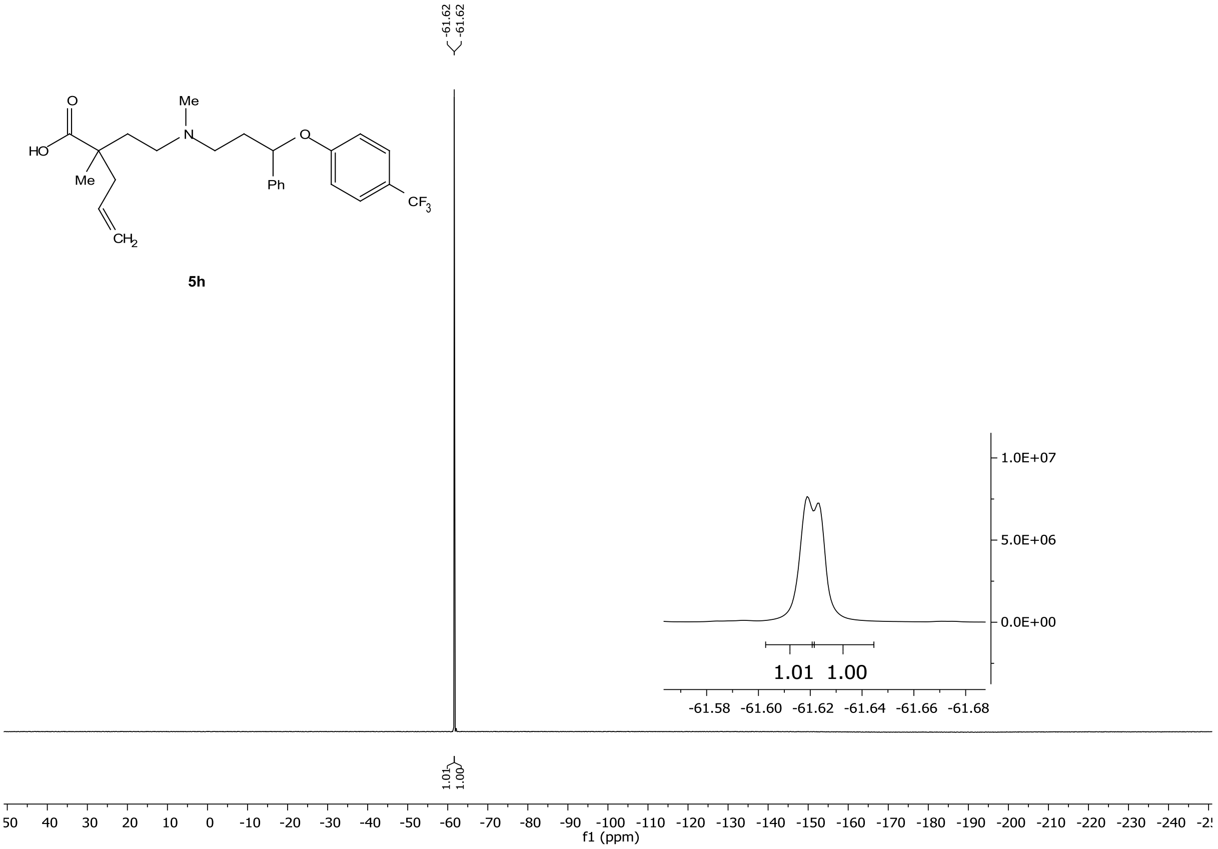

5i, ^{1}H NMR (400 MHz, CDCl$_3$, both diastereomers)

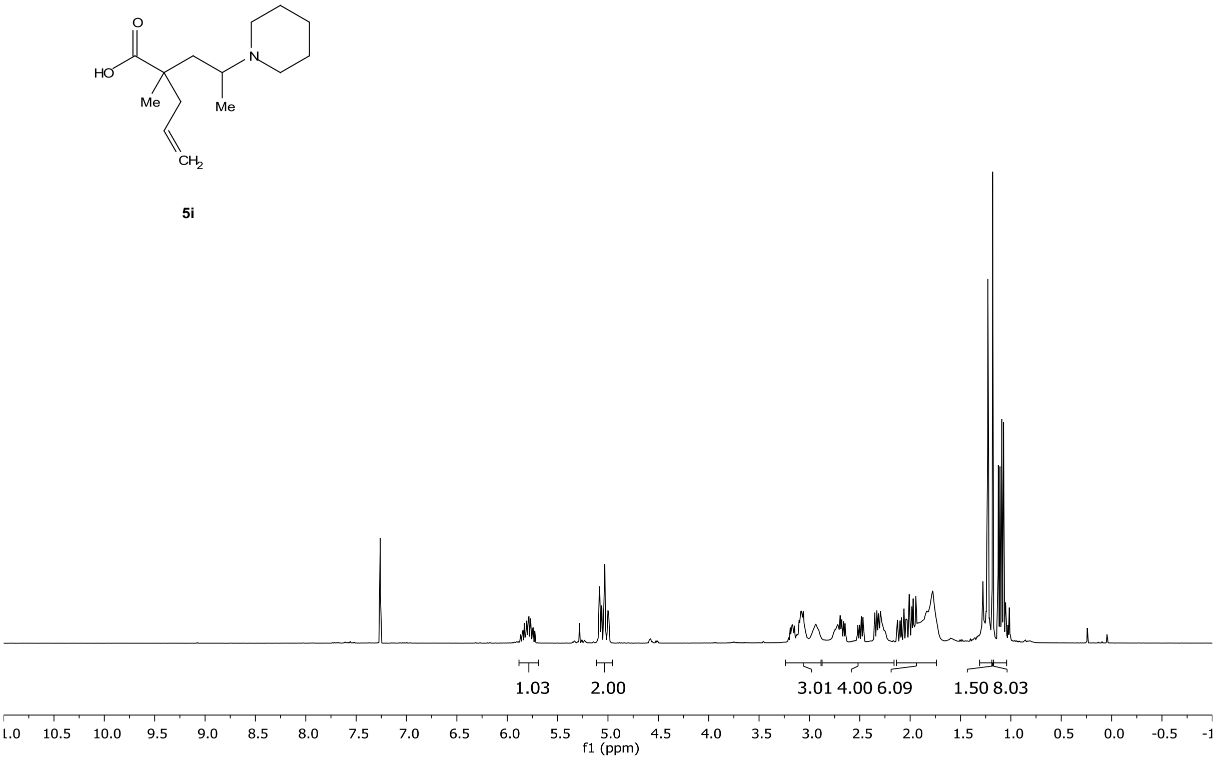

5i, ^{13}C NMR (101 MHz, $CDCl_3$, both diastereomers)

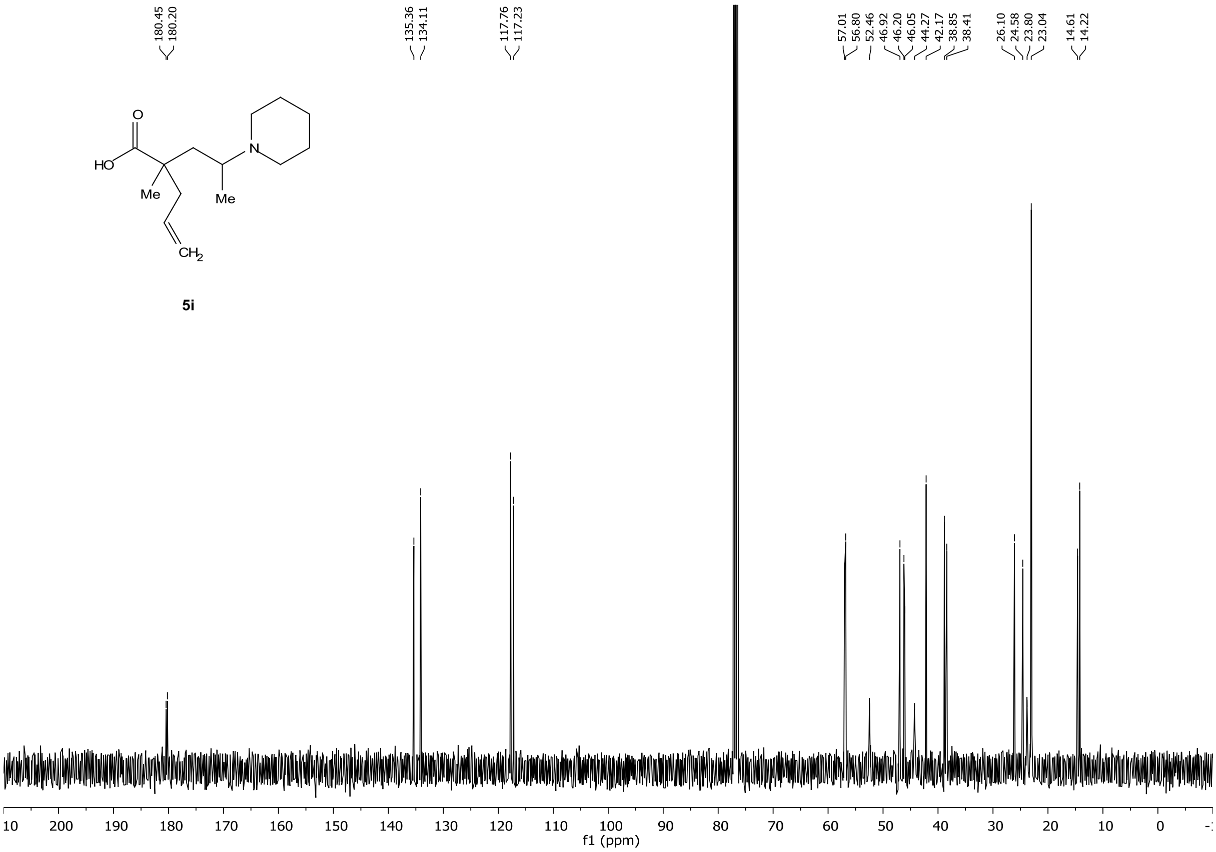

Supplement: SC-012-D0SC06385A-s001 [file SC-012-D0SC06385A-s001.pdf]
